# The Concise Guide to PHARMACOLOGY 2015/16: G protein‐coupled receptors

**DOI:** 10.1111/bph.13348

**Published:** 2015-12-09

**Authors:** Stephen PH Alexander, Anthony P Davenport, Eamonn Kelly, Neil Marrion, John A Peters, Helen E Benson, Elena Faccenda, Adam J Pawson, Joanna L Sharman, Christopher Southan, Jamie A Davies, CGTP Collaborators

**Affiliations:** ^1^ School of Biomedical Sciences University of Nottingham Medical School Nottingham NG7 2UH UK; ^2^ Clinical Pharmacology Unit University of Cambridge Cambridge CB2 0QQ UK; ^3^ School of Physiology and Pharmacology University of Bristol Bristol BS8 1TD UK; ^4^ Neuroscience Division, Medical Education Institute, Ninewells Hospital and Medical School University of Dundee Dundee DD1 9SY UK; ^5^ Centre for Integrative Physiology University of Edinburgh Edinburgh EH8 9XD UK

## Abstract

The Concise Guide to PHARMACOLOGY 2015/16 provides concise overviews of the key properties of over 1750 human drug targets with their pharmacology, plus links to an open access knowledgebase of drug targets and their ligands (www.guidetopharmacology.org), which provides more detailed views of target and ligand properties. The full contents can be found at http://onlinelibrary.wiley.com/doi/10.1111/bph.13348/full. G protein‐coupled receptors are one of the eight major pharmacological targets into which the Guide is divided, with the others being: ligand‐gated ion channels, voltage‐gated ion channels, other ion channels, nuclear hormone receptors, catalytic receptors, enzymes and transporters. These are presented with nomenclature guidance and summary information on the best available pharmacological tools, alongside key references and suggestions for further reading. The Concise Guide is published in landscape format in order to facilitate comparison of related targets. It is a condensed version of material contemporary to late 2015, which is presented in greater detail and constantly updated on the website www.guidetopharmacology.org, superseding data presented in the previous Guides to Receptors & Channels and the Concise Guide to PHARMACOLOGY 2013/14. It is produced in conjunction with NC‐IUPHAR and provides the official IUPHAR classification and nomenclature for human drug targets, where appropriate. It consolidates information previously curated and displayed separately in IUPHAR‐DB and GRAC and provides a permanent, citable, point‐in‐time record that will survive database updates.

## Conflict of interest

The authors state that there are no conflicts of interest to declare.

## Overview

G protein‐coupled receptors (GPCRs) are the largest class of membrane proteins in the human genome. The term "7TM receptor" is commonly used interchangeably with "GPCR", although there are some receptors with seven transmembrane domains that do not signal through G proteins. GPCRs share a common architecture, each consisting of a single polypeptide with an extracellular N‐terminus, an intracellular C‐terminus and seven hydrophobic transmembrane domains (TM1‐TM7) linked by three extracellular loops (ECL1‐ECL3) and three intracellular loops (ICL1‐ICL3). About 800 GPCRs have been identified in man, of which about half have sensory functions, mediating olfaction ( 400), taste (33), light perception (10) and pheromone signalling (5) [1309]. The remaining  350 non‐sensory GPCRs mediate intersignalling by ligands that range in size from small molecules to peptide to large proteins; they are the targets for the majority of drugs in clinical usage [1451, 1560], although only a minority of these receptors are exploited therapeutically. The first classification scheme to be proposed for GPCRs [984] divided them, on the basic of sequence homology, into six classes. These classes and their prototype members were as follows: **Class A**(rhodopsin‐like), **Class B** (secretin receptor family), **Class C** (metabotropic glutamate), **Class D**(fungal mating pheromone receptors), Class E (cyclic AMP receptors) and **Class F** (frizzled/smoothened). Of these, classes D and E are not found in vertebrates. An alternative classification scheme "GRAFS" [1666] divides vertebrate GPCRs into five classes, overlapping with the A‐F nomenclature, *viz*:

**Glutamate family (class C)**, which includes metabotropic glutamate receptors, a calcium‐sensing receptor and GABA_B_ receptors, as well as three taste type 1 receptors [class C list] and a family of pheromone receptors (V2 receptors) that are abundant in rodents but absent in man [1309].

**Rhodopsin family (class A),** which includes receptors for a wide variety of small molecules, neurotransmitters, peptides and hormones, together with olfactory receptors, visual pigments, taste type 2 receptors and five pheromone receptors (V1 receptors). [Class A list]

**Adhesion family** GPCRs are phylogenetically related to class B receptors, from which they differ by possessing large extracellular N‐termini that are autoproteolytically cleaved from their 7TM domains at a conserved "GPCR proteolysis site" (GPS) which lies within a much larger ( 320 residue) "GPCR autoproteolysis‐inducing" (GAIN) domain, an evolutionary ancient mofif also found in polycystic kidney disease 1 (PKD1)‐like proteins, which has been suggested to be both required and sufficient for autoproteolysis [1538]. [Adhesion family list].

**Frizzled family (class F)** consists of 10 Frizzled proteins (FZD(1‐10)) and Smoothened (SMO). [Frizzled family list]. The FZDs are activated by secreted lipoglycoproteins of the WNT family, whereas SMO is indirectly activated by the Hedgehog (HH) family of proteins acting on the transmembrane protein Patched (PTCH).

**Secretin family (class B),** encoded by 15 genes in humans. The ligands for receptors in this family are polypeptide hormones of 27‐141 amino‐acid residues; nine of the mammalian receptors respond to ligands that are structurally related to one another (glucagon, glucagon‐like peptides (GLP‐1, GLP‐2), glucose‐dependent insulinotropic polypeptide (GIP), secretin, vasoactive intestinal peptide (VIP), pituitary adenylate cyclase‐activating polypeptide (PACAP) and growth‐hormone‐releasing hormone (GHRH) [703].

## GPCR families

**Table:** 

**Family**	**Class A**	**Class B (Secretin)**	**Class C (Glutamate)**	**Adhesion**	**Frizzled**
**Receptors with known ligands**	197^a^	15	12	0	11
**Orphans**	87 (54)^a^	‐	8 (1)^a^	26 (6)^a^	0
**Sensory (olfaction)**	390^b,c^	‐	‐	‐	‐
**Sensory (vision)**	10^d^ opsins	‐	‐	‐	‐
**Sensory (taste)**	30^c^ taste 2	‐	3^c^ taste 1	‐	‐
**Sensory (pheromone)**	5^c^ vomeronasal 1	‐	‐	‐	‐
**Total**	719	15	22	33	11

^a^Numbers in brackets refer to orphan receptors for which an endogenous ligand has been proposed in at least one publication, see [396]; ^b^[1443]; ^c^[1309]; ^d^[1866].

Much of our current understanding of the structure and function of GPCRs is the result of pioneering work on the visual pigment rhodopsin and on the *β*_2_ adrenoceptor, the latter culminating in the award of the 2012 Nobel Prize in chemistry to Robert Lefkowitz and Brian Kobilka [975, 1073].

## Family structure

5746 Orphan and other 7TM receptors

5746 Class A Orphans

5756 Class C Orphans

5756 Taste 1 receptors

5757 Taste 2 receptors

5758 Other 7TM proteins

5759 5‐Hydroxytryptamine receptors

5764 Acetylcholine receptors (muscarinic)

5766 Adenosine receptors

5768 Adhesion Class GPCRs

5770 Adrenoceptors

5774 Angiotensin receptors

5775 Apelin receptor

5777 Bile acid receptor

5778 Bombesin receptors

5780 Bradykinin receptors

5781 Calcitonin receptors

5783 Calcium‐sensing receptors

5784 Cannabinoid receptors

5785 Chemerin receptor

5785 Chemokine receptors

5791 Cholecystokinin receptors

5792 Class Frizzled GPCRs

5793 Complement peptide receptors

5795 Corticotropin‐releasing factor receptors

5796 Dopamine receptors

5798 Endothelin receptors

5799 G protein‐coupled estrogen receptor

5800 Formylpeptide receptors

5801 Free fatty acid receptors

5803 GABA_B_ receptors

5805 Galanin receptors

5806 Ghrelin receptor

5807 Glucagon receptor family

5809 Glycoprotein hormone receptors

5810 Gonadotrophin‐releasing hormone receptors

5811 GPR18, GPR55 and GPR119

5812 Histamine receptors

5814 Hydroxycarboxylic acid receptors

5815 Kisspeptin receptor

5816 Leukotriene receptors

5818 Lysophospholipid (LPA) receptors

5819 Lysophospholipid (S1P) receptors

5820 Melanin‐concentrating hormone receptors

5821 Melanocortin receptors

5822 Melatonin receptors

5823 Metabotropic glutamate receptors

5826 Motilin receptor

5827 Neuromedin U receptors

5828 Neuropeptide FF/neuropeptide AF receptors

5829 Neuropeptide S receptor

5829 Neuropeptide W/neuropeptide B receptors

5830 Neuropeptide Y receptors

5832 Neurotensin receptors

5833 Opioid receptors

5835 Orexin receptors

5836 Oxoglutarate receptor

5836 P2Y receptors

5838 Parathyroid hormone receptors

5839 Platelet‐activating factor receptor

5840 Prokineticin receptors

5841 Prolactin‐releasing peptide receptor

5842 Prostanoid receptors

5844 Proteinase‐activated receptors

5846 QRFP receptor

5846 Relaxin family peptide receptors

5848 Somatostatin receptors

5850 Succinate receptor

5850 Tachykinin receptors

5852 Thyrotropin‐releasing hormone receptors

5852 Trace amine receptor

5854 Urotensin receptor

5854 Vasopressin and oxytocin receptors

5856 VIP and PACAP receptors

## Orphan and other 7TM receptors

## Class A Orphans

### Overview

Table 1 lists a number of putative GPCRs identified by NC‐IUPHAR[**530**], for which preliminary evidence for an endogenous ligand has been published, or for which there exists a potential link to a disease, or disorder. These GPCRs have recently been reviewed in detail [396]. The GPCRs in Table 1 are all Class A, rhodopsin‐like GPCRs. Class A orphan GPCRs not listed in Table 1 are putative GPCRs with as‐yet unidentified endogenous ligands.

**Table 1:** Class A orphan GPCRs with putative endogenous ligands

*GPR1*	*GPR3*	*GPR4*	*GPR6*	*GPR12*	*GPR15*	*GPR17*	*GPR20*
*GPR22*	*GPR26*	*GPR31*	*GPR34*	*GPR35*	*GPR37*	*GPR39*	*GPR50*
*GPR63*	*GRP65*	*GPR68*	*GPR75*	*GPR84*	*GPR87*	*GPR88*	*GPR132*
*GPR149*	*GPR161*	*GPR183*	*LGR4*	*LGR5*	*LGR6*	*MAS1*	*MRGPRD*
*MRGPRX1*	*MRGPRX2*	*P2RY10*	*TAAR2*				

In addition the orphan receptors *GPR18*, *GPR55* and *GPR119* which are reported to respond to endogenous agents analogous to the endogenous cannabinoid ligands have been grouped together (GPR18, GPR55 and GPR119).

**Table:** 

Nomenclature	*GPR1*	*GPR3*	*GPR4*	*GPR6*
HGNC, UniProt	*GPR1*, P46091	*GPR3*, P46089	*GPR4*, P46093	*GPR6*, P46095
Endogenous ligand	–	–	Protons	–
Endogenous agonists	chemerin (*RARRES2*, Q99969) (p*K*_d_ 8.3) [95]	–	–	–
Agonists	–	diphenyleneiodonium chloride (pEC_50_ 6) [2091]	–	–
Comments	Reported to act as a co‐receptor for HIV [1724]. See review [396] for discussion of pairing with chemerin.	sphingosine 1‐phosphate was reported to be an endogenous agonist [1921], but this finding was not replicated in subsequent studies [2093]. Reported to activate adenylyl cyclase constitutively through G_s_ [466]. Gene disruption results in premature ovarian ageing [1063], reduced *β*‐amyloid deposition [1868] and hypersensitivity to thermal pain [1615] in mice. First small molecule inverse agonist [860] and agonists identified [2091].	An initial report suggesting activation by lysophosphatidylcholine and sphingosylphosphorylcholine [2131] has been retracted [2148]. GPR4, GPR65, GPR68 and GPR132 are now thought to function as proton‐sensing receptors detecting acidic pH [396, 1704]. Gene disruption is associated with increased perinatal mortality and impaired vascular proliferation [2085]. Negative allosteric modulators of GPR4 have been reported [1889].	An initial report that sphingosine 1‐phosphate (S1P) was a high‐affinity ligand (EC_50_ value of 39nM) [815, 1921] was not repeated by arrestin PathHunter[TM] assays [1785, 2093]. Reported to activate adenylyl cyclase constitutively through G_s_ and to be located intracellularly [1453]. GPR6‐deficient mice showed reduced striatal cyclic AMP production *in vitro* and selected alterations in instrumental conditioning *in vivo*. [1134].

**Table:** 

Nomenclature	*GPR12*	*GPR15*	*GPR17*	*GPR19*
HGNC, UniProt	*GPR12*, P47775	*GPR15*, P49685	*GPR17*, Q13304	*GPR19*, Q15760
Endogenous agonists	–	–	UDP‐glucose (pEC_50_ 5.9–9.5) [130, 344], LTC_4_ (pEC_50_ 7.8–9.5) [344], UDP‐galactose (pEC_50_ 6–8.9) [130, 344], uridine diphosphate (pEC_50_ 6–8.8) [130, 344], LTD_4_ (pEC_50_ 8.1–8.4) [344]	–
Comments	Reports that sphingosine 1‐phosphate is a ligand of GPR12 [814, 1921] have not been replicated in arrestin‐based assays [1785, 2093]. Gene disruption results in dyslipidemia and obesity [154].	Reported to act as a co‐receptor for HIV [462]. In an infection‐induced colitis model, *Gpr15* knockout mice were more prone to tissue damage and inflammatory cytokine expression [945].	Reported to be a dual leukotriene and uridine diphosphate receptor [344]. Another group instead proposed that GPR17 functions as a negative regulator of the CysLT_1_ receptor response to leukotriene D_4_ (LTD_4_). For further discussion, see [396]. Reported to antagonize CysLT_1_ receptor signalling *in vivo*and *in vitro* [1175]. See reviews [250] and [396].	–

**Table:** 

Nomenclature	*GPR20*	*GPR21*	*GPR22*	*GPR25*	*GPR26*
HGNC, UniProt	*GPR20*, Q99678	*GPR21*, Q99679	*GPR22*, Q99680	*GPR25*, O00155	*GPR26*, Q8NDV2
Comments	Reported to inhibit adenylyl cyclase constitutively through G_i/o_ [708]. GPR20 deficient mice exhibit hyperactivity characterised by increased total distance travelled in an open field test [207].	*Gpr21* knockout mice were resistant to diet‐induced obesity, exhibiting an increase in glucose tolerance and insulin sensitivity, as well as a modest lean phenotype [1448].	Gene disruption results in increased severity of functional decompensation following aortic banding [10]. Identified as a susceptibility locus for osteoarthritis [494, 929, 1935].	–	Has been reported to activate adenylyl cyclase constitutively through G_s_ [880]. *Gpr26* knockout mice show increased levels of anxiety and depression‐like behaviours [2117].

**Table:** 

Nomenclature	*GPR27*	*GPR31*	*GPR32*	*GPR33*	*GPR34*
HGNC, UniProt	*GPR27*, Q9NS67	*GPR31*, O00270	*GPR32*, O75388	*GPR33*, Q49SQ1	*GPR34*, Q9UPC5
Rank order of potency	–	–	resolvin D1>LXA_4_	–	–
Endogenous agonists	–	12S‐HETE (Selective) (pEC_50_ 9.6) [665] – Mouse	resolvin D1 (pEC_50_ 11.1) [1006], LXA_4_ (pEC_50_ 9.7) [1006]	–	lysophosphatidylserine (Selective) (pEC_50_ 6.6–6.9) [960, 1817]
Labelled ligands	–	–	[^3^H]resolvin D1 (Agonist) (p*K*_d_ 9.7) [1006]	–	–
Comments	Knockdown of Gpr27 reduces endogenous mouse insulin promotor activity and glucose‐stimulated insulin secretion [1012].	See [396] for discussion of pairing.	resolvin D1 has been demonstrated to activate GPR32 in two publications [316, 1006]. The pairing was not replicated in a recent study based on arrestin recruitment [1785]. *GPR32* is a pseudogene in mice and rats. See reviews [250] and [396].	*GPR33* is a pseudogene in most individuals, containing a premature stop codon within the coding sequence of the second intracellular loop [1621].	Lysophosphatidylserine has been reported to be a ligand of GPR34 in several publications, but the pairing was not replicated in a recent study based on arrestin recruitment [1785]. Fails to respond to a variety of lipid‐derived agents [2093]. Gene disruption results in an enhanced immune response [1102]. Characterization of agonists at this receptor is discussed in [819] and [396].

**Table:** 

Nomenclature	*GPR35*	*GPR37*	*GPR37L1*	*GPR39*	*GPR42*
HGNC, UniProt	*GPR35*, Q9HC97	*GPR37*, O15354	*GPR37L1*, O60883	*GPR39*, O43194	*GPR42*, O15529
Endogenous agonists	2‐oleoyl‐LPA (pEC_50_ 7.3–7.5) [1436], kynurenic acid (pEC_50_ 3.9–4.4) [1785, 1980]	–	–	Zn^2+^ [775]	–
Agonists	–	neuropeptide head activator (pEC_50_ 8–8.5) [1578]	–	compound 1 [PMID: 24900608] (pEC_50_ 4.9–7.2) [166]	–
Comments	Several studies have shown that kynurenic acid is an agonist of GPR35 but it remains controversial whether the proposed endogenous ligand reaches sufficient tissue concentrations to activate the receptor [1015]. 2‐oleoyl‐LPA has also been proposed as an endogenous ligand [1436] but these results were not replicated in an arrestin assay [1785]. The phosphodiesterase inhibitor zaprinast [1863] has become widely used as a surrogate agonist to investigate GPR35 pharmacology and signalling [1863]. GPR35 is also activated by the pharmaceutical adjunct pamoic acid [2124]. See reviews [396] and [429].	Reported to associate and regulate the dopamine transporter [1207] and to be a substrate for parkin [1205]. Gene disruption results in altered striatal signalling [1206]. The peptides prosaptide and prosaposin are proposed as endogenous ligands for GPR37 and GPR37L1 [1264].	The peptides prosaptide and prosaposin are proposed as endogenous ligands for GPR37 and GPR37L1 [1264].	Zn^2+^ has been reported to be a potent and efficacious agonist of human, mouse and rat GPR39 [2089]. obestatin (*GHRL*, Q9UBU3), a fragment from the ghrelin precursor, was reported initially as an endogenous ligand, but subsequent studies failed to reproduce these findings. *GPR39* has been reported to be down‐regulated in adipose tissue in obesity‐related diabetes [273]. Gene disruption results in obesity and altered adipocyte metabolism [1497]. Reviewed in [396].	–

**Table:** 

Nomenclature	*GPR45*	*GPR50*	*GPR52*	*GPR61*
HGNC, UniProt	*GPR45*, Q9Y5Y3	*GPR50*, Q13585	*GPR52*, Q9Y2T5	*GPR61*, Q9BZJ8
Comments	–	GPR50 is structurally related to MT_1_ and MT_2_ melatonin receptors, with which it heterodimerises constitutively and specifically [1089]. *Gpr50* knockout mice display abnormal thermoregulation and are much more likely than wild‐type mice to enter fasting‐induced torpor [111].	First small molecule agonist reported [1703].	GPR61 deficient mice exhibit obesity associated with hyperphagia [1363]. Although no endogenous ligands have been identified, 5‐(nonyloxy)tryptamine has been reported to be a low affinity inverse agonist [1852].

**Table:** 

Nomenclature	*GPR62*	*GPR63*	*GPR65*	*GPR68*	*GPR75*
HGNC, UniProt	*GPR62*, Q9BZJ7	*GPR63*, Q9BZJ6	*GPR65*, Q8IYL9	*GPR68*, Q15743	*GPR75*, O95800
Endogenous ligand	–	–	Protons	Protons	–
Comments	–	sphingosine 1‐phosphate and dioleoylphosphatidic acid have been reported to be low affinity agonists for GPR63 [1394] but this finding was not replicated in an arrestin‐based assay [2093].	GPR4, GPR65, GPR68 and GPR132 are now thought to function as proton‐sensing receptors detecting acidic pH [396, 1704]. Reported to activate adenylyl cyclase; gene disruption leads to reduced eosinophilia in models of allergic airway disease [1000].	GPR68 was previously identified as a receptor for sphingosylphosphorylcholine (SPC) [2068], but the original publication has been retracted [2067]. GPR4, GPR65, GPR68 and GPR132 are now thought to function as proton‐sensing receptors detecting acidic pH [396, 1704]. A family of 3,5‐disubstituted isoxazoles were identified as agonists of GPR68 [1617].	CCL5 (*CCL5*, P13501) was reported to be an agonist of GPR75 [816], but the pairing could not be repeated in an arrestin assay [1785].

**Table:** 

Nomenclature	*GPR78*	*GPR79*	*GPR82*	*GPR83*	*GPR84*
HGNC, UniProt	*GPR78*, Q96P69	*GPR79*, –	*GPR82*, Q96P67	*GPR83*, Q9NYM4	*GPR84*, Q9NQS5
Agonists	–	–	–	Zn^2+^ (pEC_50_ 5) [1351] – Mouse	decanoic acid (pEC_50_ 5–5.4) [1785, 1981], undecanoic acid (pEC_50_ 5.1) [1981], lauric acid (pEC_50_ 5) [1981]
Comments	GPR78 has been reported to be constitutively active, coupled to elevated cAMP production [880].	–	Mice with *Gpr82* knockout have a lower body weight and body fat content associated with reduced food intake, decreased serum triglyceride levels, as well as higher insulin sensitivity and glucose tolerance [479].	One isoform has been implicated in the induction of CD4(+) CD25(+) regulatory T cells (Tregs) during inflammatory immune responses [696]. The extracellular N‐terminal domain is reported as an intramolecular inverse agonist [1352].	Medium chain free fatty acids with carbon chain lengths of 9‐14 activate GPR84 [1828, 1981]. A surrogate ligand for GPR84, 6‐n‐octylaminouracil has also been proposed [1828]. See review [396] for discussion of classification. Mutational analysis and molecular modelling of GPR84 has been reported [1397].

**Table:** 

Nomenclature	*GPR85*	*GPR87*	*GPR88*	*GPR101*
HGNC, UniProt	*GPR85*, P60893	*GPR87*, Q9BY21	*GPR88*, Q9GZN0	*GPR101*, Q96P66
Endogenous agonists	–	LPA (pEC_50_ 7.4) [1344, 1836]	–	–
Agonists	–	–	compound 2 [PMID: 24793972] (pEC_50_ 6.2) [868]	–
Comments	Proposed to regulate hippocampal neurogenesis in the adult, as well as neurogenesis‐dependent learning and memory [303].	–	Gene disruption results in altered striatal signalling [1137]. Small molecule agonists have been reported [147].	Mutations in GPR101 have been linked to gigantism and acromegaly [1906].

**Table:** 

Nomenclature	*GPR132*	*GPR135*	*GPR139*	*GPR141*	*GPR142*
HGNC, UniProt	*GPR132*, Q9UNW8	*GPR135*, Q8IZ08	*GPR139*, Q6DWJ6	*GPR141*, Q7Z602	*GPR142*, Q7Z601
Endogenous ligand	Protons	–	–	–	–
Agonists	–	–	compound 1a [PMID: 24900311] (pEC_50_ 7.4) [1721]	–	–
Comments	GPR4, GPR65, GPR68 and GPR132 are now thought to function as proton‐sensing receptors detecting acidic pH [396, 1704]. Reported to respond to lysophosphatidylcholine [891], but later retracted [2038].	–	Peptide agonists have been reported [828].	–	Small molecule agonists have been reported [1890, 2106].

**Table:** 

Nomenclature	*GPR146*	*GPR148*	*GPR149*	*GPR150*	*GPR151*
HGNC, UniProt	*GPR146*, Q96CH1	*GPR148*, Q8TDV2	*GPR149*, Q86SP6	*GPR150*, Q8NGU9	*GPR151*, Q8TDV0
Comments	Yosten *et al*. demonstrated inhibition of proinsulin C‐peptide (*INS*, P01308)‐induced stimulation of cFos expression folllowing knockdown of GPR146 in KATO III cells, suggesting proinsulin C‐peptide as an endogenous ligand of the receptor [2103].	–	*Gpr149* knockout mice displayed increased fertility and enhanced ovulation, with increased levels of FSH receptor and cyclin D2 mRNA levels [463].	–	GPR151 responded to galanin with an EC_50_ value of 2 *μ*M, suggesting that the endogenous ligand shares structural features with galanin (*GAL*, P22466) [813].

**Table:** 

Nomenclature	*GPR152*	*GPR153*	*GPR160*	*GPR161*	*GPR162*
HGNC, UniProt	*GPR152*, Q8TDT2	*GPR153*, Q6NV75	*GPR160*, Q9UJ42	*GPR161*, Q8N6U8	*GPR162*, Q16538
Comments	–	–	–	A C‐terminal truncation (deletion) mutation in Gpr161 causes congenital cataracts and neural tube defects in the vacuolated lens (vl) mouse mutant [1226]. The mutated receptor is associated with cataract, spina bifida and white belly spot phenotypes in mice [994]. Gene disruption is associated with a failure of asymmetric embryonic development in zebrafish [1085].	–

**Table:** 

Nomenclature	*GPR171*	*GPR173*	*GPR174*	*GPR176*	*GPR182*
HGNC, UniProt	*GPR171*, O14626	*GPR173*, Q9NS66	*GPR174*, Q9BXC1	*GPR176*, Q14439	*GPR182*, O15218
Endogenous agonists	–	–	lysophosphatidylserine (pEC_50_ 7.1) [825]	–	–
Comments	GPR171 has been shown to be activated by the endogenous peptide BigLEN {Mouse}. This receptor‐peptide interaction is believed to be involved in regulating feeding and metabolism responses [621].	–	See [819] which discusses characterization of agonists at this receptor.	–	Rat GPR182 was first proposed as the adrenomedullin receptor [904]. However, it was later reported that rat and human GPR182 did not respond to adrenomedullin [927] and GPR182 is not currently considered to be a genuine adrenomedullin receptor [722].

**Table:** 

Nomenclature	*GPR183*	*LGR4*	*LGR5*	*LGR6*	*MAS1*
HGNC, UniProt	*GPR183*, P32249	*LGR4*, Q9BXB1	*LGR5*, O75473	*LGR6*, Q9HBX8	*MAS1*, P04201
Endogenous agonists	7*α*,25‐dihydroxycholesterol (Selective) (pEC_50_ 8.1–9.8) [694, 1125], 7*α*,27‐dihydroxycholesterol (Selective) (pEC_50_ 8.9) [1125], 7*β*, 25‐dihydroxycholesterol (Selective) (pEC_50_ 8.7) [1125], 7*β*, 27‐dihydroxycholesterol (Selective) (pEC_50_ 7.3) [1125]	R‐spondin‐2 (*RSPO2*, Q6UXX9) (pEC_50_ 12.5) [266], R‐spondin‐1 (*RSPO1*, Q2MKA7) (pEC_50_ 10.7) [266], R‐spondin‐3 (*RSPO3*, Q9BXY4) (pEC_50_ 10.7) [266], R‐spondin‐4 (*RSPO4*, Q2I0M5) (pEC_50_ 10.1) [266]	R‐spondin‐2 (*RSPO2*, Q6UXX9) (pEC_50_ 12) [266], R‐spondin‐1 (*RSPO1*, Q2MKA7) (pEC_50_ 11.1) [266], R‐spondin‐3 (*RSPO3*, Q9BXY4) (pEC_50_ 11) [266], R‐spondin‐4 (*RSPO4*, Q2I0M5) (pEC_50_ 9.4) [266]	R‐spondin‐1 (*RSPO1*, Q2MKA7) [266, 2140], R‐spondin‐2 (*RSPO2*, Q6UXX9) [266, 2140], R‐spondin‐3 (*RSPO3*, Q9BXY4) [266, 2140], R‐spondin‐4 (*RSPO4*, Q2I0M5) [266, 2140]	–
Agonists	–	–	–	–	angiotensin‐(1‐7) (*AGT*, P01019) (p*K*_i_ 7.3) [612] – Mouse
Comments	Two independent publications have shown that 7*α*,25‐dihydroxycholesterol is an agonist of GPR183 and have demonstrated by mass spectrometry that this oxysterol is present endogenously in tissues [694, 1125]. Gpr183‐deficient mice show a reduction in the early antibody response to a T‐dependent antigen. GPR183‐deficient B cells fail to migrate to the outer follicle and instead stay in the follicle centre [923, 1488].	LGR4 does not couple to heterotrimeric G proteins or recruit arrestins when stimulated by the R‐spondins, indicating a unique mechanism of action. R‐spondins bind to LGR4, which specifically associates with Frizzled and LDL receptor‐related proteins (LRPs) that are activated by the extracellular Wnt molecules and then trigger canonical Wnt signalling to increase gene expression [266, 1612, 2140]. Gene disruption leads to multiple developmental disorders [869, 1154, 1781, 2005].	The four R‐spondins can bind to LGR4, LGR5, and LGR6, which specifically associate with Frizzled and LDL receptor‐related proteins (LRPs), proteins that are activated by extracellular Wnt molecules and which then trigger canonical Wnt signalling to increase gene expression [266, 2140].	–	–

**Table:** 

Nomenclature	*MAS1L*	*MRGPRD*	*MRGPRE*	*MRGPRF*	*MRGPRG*
HGNC, UniProt	*MAS1L*, P35410	*MRGPRD*, Q8TDS7	*MRGPRE*, Q86SM8	*MRGPRF*, Q96AM1	*MRGPRG*, Q86SM5
Endogenous agonists	–	*β*‐alanine (pEC_50_ 4.8) [1729, 1785]	–	–	–
Comments	–	An endogenous peptide with a high degree of sequence similarity to angiotensin‐(1‐7) (*AGT*, P01019), alamandine (*AGT*), was shown to promote NO release in MRGPRD‐transfected cells. The binding of alamandine to MRGPRD to was shown to be blocked by D‐Pro^7^‐angiotensin‐(1‐7), *β*‐alanine and PD123319 [1045]. Genetic ablation of MRGPRD+ neurons of adult mice decreased behavioural sensitivity to mechanical stimuli but not to thermal stimuli [278]. See reviews [396] and [1779].	See reviews [396] and [1779].	MRGPRF has been reported to respond to stimulation by angiotensin metabolites [589]. See reviews [396] and [1779].	See reviews [396] and [1779].

**Table:** 

Nomenclature	*MRGPRX1*	*MRGPRX2*	*MRGPRX3*	*MRGPRX4*
HGNC, UniProt	*MRGPRX1*, Q96LB2	*MRGPRX2*, Q96LB1	*MRGPRX3*, Q96LB0	*MRGPRX4*, Q96LA9
Endogenous agonists	bovine adrenal medulla peptide 8‐22 (*PENK*, P01210) (Selective) (pEC_50_ 5.3–7.8) [299, 1080, 1785]	PAMP‐20 (*ADM*, P35318) (Selective) [899]	–	–
Agonists	–	cortistatin‐14 {Mouse, Rat} (pEC_50_ 6.9–7.6) [899, 1594, 1785]	–	–
Selective agonists	–	PAMP‐12 (human) (pEC_50_ 7.2–7.7) [899]	–	–
Comments	Reported to mediate the sensation of itch [1131, 1739]. Reports that bovine adrenal medulla peptide 8‐22 (*PENK*, P01210) was the most potent of a series of proenkephalin A‐derived peptides as an agonist of MRGPRX1 in assays of calcium mobilisation and radioligand binding [1080] were replicated in an independent study using an arrestin recruitment assay [1785]. See reviews [396] and [1779].	A diverse range of substances has been reported to be agonists of MRGPRX2, with cortistatin 14 the highest potency agonist in assays of calcium mobilisation [1594], also confirmed in an independent study using an arrestin recruitment assay [1785]. See reviews [396] and [1779].	–	See reviews [396] and [1779].

**Table:** 

Nomenclature	*OPN3*	*OPN4*	*OPN5*	*P2RY8*
HGNC, UniProt	*OPN3*, Q9H1Y3	*OPN4*, Q9UHM6	*OPN5*, Q6U736	*P2RY8*, Q86VZ1
Comments	–	–	Evidence indicates OPN5 triggers a UV‐sensitive G_i_‐mediated signalling pathway in mammalian tissues [982].	–

**Table:** 

Nomenclature	*P2RY10*	*TAAR2*	*TAAR3*	*TAAR4P*
HGNC, UniProt	*P2RY10*, O00398	*TAAR2*, Q9P1P5	*TAAR3*, Q9P1P4	*TAAR4P*, –
Rank order of potency	–	*β*‐phenylethylamine>tryptamine [185]	–	–
Endogenous agonists	sphingosine 1‐phosphate (Selective) (pEC_50_ 7.3) [1344], LPA (Selective) (pEC_50_ 6.9) [1344]	–	–	–
Comments	–	Probable pseudogene in 10‐15% of Asians due to a polymorphism (rs8192646) producing a premature stop codon at amino acid 168 [396].	*TAAR3* is thought to be a pseudogene in man though functional in rodents [396].	Pseudogene in man but functional in rodents [396].

**Table:** 

Nomenclature	*TAAR5*	*TAAR6*	*TAAR8*	*TAAR9*
HGNC, UniProt	*TAAR5*, O14804	*TAAR6*, Q96RI8	*TAAR8*, Q969N4	*TAAR9*, Q96RI9
Comments	Trimethylamine is reported as an agonist [1974] and 3‐iodothyronamine an inverse agonist [426].	–	–	*TAAR9* appears to be functional in most individuals but has a polymorphic premature stop codon at amino acid 61 (rs2842899) with an allele frequency of 10‐30% in different populations [1944].

## Class C Orphans

**Table:** 

Nomenclature	*GPR156*	*GPR158*	*GPR179*	*GPRC5A*	*GPRC5B*	*GPRC5C*	*GPRC5D*
HGNC, UniProt	*GPR156*, Q8NFN8	*GPR158*, Q5T848	*GPR179*, Q6PRD1	*GPRC5A*, Q8NFJ5	*GPRC5B*, Q9NZH0	*GPRC5C*, Q9NQ84	*GPRC5D*, Q9NZD1

## Taste 1 receptors

### Overview

Whilst the taste of acid and salty foods appear to be sensed by regulation of ion channel activity, bitter, sweet and umami tastes are sensed by specialised GPCR. Two classes of taste GPCR have been identified, T1R and T2R, which are similar in sequence and structure to Class C and Class A GPCR, respectively. Activation of taste receptors appears to involve gustducin‐ (G*α*t3) and G*α*14‐mediated signalling, although the precise mechanisms remain obscure. Gene disruption studies suggest the involvement of PLC*β*2 [2122], TRPM5 [2122] and IP3 [764] receptors in post‐receptor signalling of taste receptors. Although predominantly associated with the oral cavity, taste receptors are also located elsewhere, including further down the gastrointestinal system, in the lungs and in the brain.

### **Sweet/Umami**

T1R3 acts as an obligate partner in T1R1/T1R3 and T1R2/T1R3 heterodimers, which sense umami or sweet, respectively. T1R1/T1R3 heterodimers respond to L‐glutamic acid and may be positively allosterically modulated by 5'‐nucleoside monophosphates, such as 5'‐GMP[1096]. T1R2/T1R3 heterodimers respond to sugars, such as sucrose, and artificial sweeteners, such as saccharin[1376].

**Table:** 

Nomenclature	*TAS1R1*	*TAS1R2*	*TAS1R3*
HGNC, UniProt	*TAS1R1*, Q7RTX1	*TAS1R2*, Q8TE23	*TAS1R3*, Q7RTX0

## Taste 2 receptors

### **Bitter**

The composition and stoichiometry of bitter taste receptors is not yet established. Bitter receptors appear to separate into two groups, with very restricted ligand specificity or much broader responsiveness. For example, T2R5 responded to cycloheximide, but not 10 other bitter compounds [287], while T2R14 responded to at least eight different bitter tastants, including (‐)‐*α*‐thujone and picrotoxinin[119].

Specialist database BitterDB contains additional information on bitter compounds and receptors [2023].

**Table:** 

Nomenclature	*TAS2R1*	*TAS2R3*	*TAS2R4*	*TAS2R5*	*TAS2R7*	*TAS2R8*
HGNC, UniProt	*TAS2R1*, Q9NYW7	*TAS2R3*, Q9NYW6	*TAS2R4*, Q9NYW5	*TAS2R5*, Q9NYW4	*TAS2R7*, Q9NYW3	*TAS2R8*, Q9NYW2

**Table:** 

Nomenclature	*TAS2R9*	*TAS2R10*	*TAS2R13*	*TAS2R14*	*TAS2R16*	*TAS2R19*
HGNC, UniProt	*TAS2R9*, Q9NYW1	*TAS2R10*, Q9NYW0	*TAS2R13*, Q9NYV9	*TAS2R14*, Q9NYV8	*TAS2R16*, Q9NYV7	*TAS2R19*, P59542

**Table:** 

Nomenclature	*TAS2R20*	*TAS2R30*	*TAS2R31*	*TAS2R38*	*TAS2R39*	*TAS2R40*
HGNC, UniProt	*TAS2R20*, P59543	*TAS2R30*, P59541	*TAS2R31*, P59538	*TAS2R38*, P59533	*TAS2R39*, P59534	*TAS2R40*, P59535

**Table:** 

Nomenclature	*TAS2R41*	*TAS2R42*	*TAS2R43*	*TAS2R45*	*TAS2R46*	*TAS2R50*	*TAS2R60*
HGNC, UniProt	*TAS2R41*, P59536	*TAS2R42*, Q7RTR8	*TAS2R43*, P59537	*TAS2R45*, P59539	*TAS2R46*, P59540	*TAS2R50*, P59544	*TAS2R60*, P59551

## Other 7TM proteins

**Table:** 

Nomenclature	*GPR107*	*GPR137*	*OR51E1*	*TPRA1*	*GPR143*	*GPR157*
HGNC, UniProt	*GPR107*, Q5VW38	*GPR137*, Q96N19	*OR51E1*, Q8TCB6	*TPRA1*, Q86W33	*GPR143*, P51810	*GPR157*, Q5UAW9
Endogenous agonists	–	–	–	–	levodopa [1141]	–
Comments	GPR107 is a member of the LUSTR family of proteins found in both plants and animals, having similar topology to Gprotein‐coupled receptors [461]	–	OR51E1 is a putative olfactory receptor.	TPRA1 shows no homology to known G protein‐coupled receptors.	Loss‐of‐function mutations underlie ocular albinism type 1 [103].	GPR157 has ambiguous sequence similarities to several different GPCR families (class A, class B and the slime mould cyclic AMP receptor). Because of its distant relationship to other GPCRs, it cannot be readily classified.

## 5‐Hydroxytryptamine receptors

### Overview

5‐HT receptors (**nomenclature as agreed by the NC‐IUPHAR Subcommittee on 5‐HT receptors [** **789** **] and subsequently revised [** **707** **]**) are, with the exception of the ionotropic 5‐HT_3_ class, GPCR receptors where the endogenous agonist is 5‐hydroxytryptamine. The diversity of metabotropic 5‐HT receptors is increased by alternative splicing that produces isoforms of the 5‐HT_2A_ (non‐functional), 5‐HT_2C_ (non‐functional), 5‐HT_4_, 5‐HT_6_ (non‐functional) and 5‐HT_7_ receptors. Unique amongst the GPCRs, RNA editing produces 5‐HT_2C_ receptor isoforms that differ in function, such as efficiency and specificity of coupling to G_q/11_ and also pharmacology [164, 2011]. Most 5‐HT receptors (except 5‐ht _1e_ and 5‐ht _5a/5b_) play specific roles mediating functional responses in different tissues (reviewed by [1554, 1957].

**Table:** 

Nomenclature	5‐HT_1A_ receptor	5‐HT_1B_ receptor	5‐HT_1D_ receptor	5‐ht_1e_ receptor	5‐HT_1F_ receptor
HGNC, UniProt	*HTR1A*, P08908	*HTR1B*, P28222	*HTR1D*, P28221	*HTR1E*, P28566	*HTR1F*, P30939
Agonists	U92016A (p*K*_i_ 9.7) [1240], vilazodone (Partial agonist) (p*K*_i_ 9.7) [402], vortioxetine (Partial agonist) (p*K*_i_ 7.8) [90]	L‐694,247 (p*K*_i_ 9.2) [637], naratriptan (Partial agonist) (p*K*_i_ 8.1) [1365], eletriptan (p*K*_i_ 8) [1365], frovatriptan (p*K*_i_ 8) [2069], zolmitriptan (Partial agonist) (p*K*_i_ 7.7) [1365], vortioxetine (Partial agonist) (p*K*_i_ 7.5) [90], rizatriptan (Partial agonist) (p*K*_i_ 6.9) [1365]	dihydroergotamine (p*K*_i_ 9.2–9.9) [684, 1084, 1091], ergotamine (p*K*_i_ 9.1) [616], L‐694,247 (p*K*_i_ 9) [2052], naratriptan (p*K*_i_ 8.4–9) [432, 1365, 1577], zolmitriptan (p*K*_i_ 8.9) [1365], frovatriptan (p*K*_i_ 8.4) [2069], rizatriptan (p*K*_i_ 7.9) [1365]	BRL‐54443 (p*K*_i_ 8.7) [227]	BRL‐54443 (p*K*_i_ 8.9) [227], eletriptan (p*K*_i_ 8) [1365], sumatriptan (p*K*_i_ 7.2–7.9) [11, 12, 1365, 1968]
Selective agonists	8‐OH‐DPAT (p*K*_i_ 8.4–9.4) [406, 685, 896, 1079, 1280, 1386, 1388, 1389], NLX‐101 (p*K*_i_ 8.6) [1387]	CP94253 (p*K*_i_ 8.7) [976]	PNU109291 (p*K*_i_ 9.1) [483] – Gorilla, eletriptan (p*K*_i_ 8.9) [1365]	–	lasmiditan (p*K*_i_ 8.7) [1375], LY334370 (p*K*_i_ 8.7) [1968], 5‐BODMT (p*K*_i_ 8.4) [966], LY344864 (p*K*_i_ 8.2) [1502]
Antagonists	(S)‐UH 301 (p*K*_i_ 7.9) [1386]	–	–	–	–
Selective antagonists	WAY‐100635 (p*K*_i_ 7.9–9.2) [1386, 1388], robalzotan (p*K*_i_ 9.2) [872]	SB 224289 (Inverse agonist) (p*K*_i_ 8.2–8.6) [583, 1384, 1696], SB236057 (Inverse agonist) (p*K*_i_ 8.2) [1272], GR‐55562 (p*K*_B_ 7.4) [791]	SB 714786 (p*K*_i_ 9.1) [1987]	–	–
Nomenclature	5‐HT_1A_ receptor	5‐HT_1B_ receptor	5‐HT_1D_ receptor	5‐ht_1e_ receptor	5‐HT_1F_ receptor
Labelled ligands	[^3^H]robalzotan (Antagonist) (p*K*_d_ 9.8) [861], [^3^H]WAY100635 (Antagonist) (p*K*_d_ 9.5) [933], [^3^H]8‐OH‐DPAT (Agonist) (p*K*_d_ 6–9.4) [156, 896, 1385, 1388], [^3^H]NLX‐112 (Agonist) (p*K*_d_ 8.9) [748], [^11^C]WAY100635 (Antagonist) [1915], p‐[^18^F]MPPF (Antagonist) [368]	[^3^H]N‐methyl‐AZ10419369 (Agonist, Partial agonist) (p*K*_d_ 9.4) [1182], [^3^H]GR 125,743 (Selective Antagonist) (p*K*_d_ 8.6–9.2) [637, 2061], [^3^H]alniditan (Agonist) (p*K*_d_ 8.6–9) [1084], [^125^I]GTI (Agonist) (p*K*_d_ 8.9) [193, 232] – Rat, [^3^H]eletriptan (Agonist, Partial agonist) (p*K*_d_ 8.5) [1365], [^3^H]sumatriptan (Agonist, Partial agonist) (p*K*_d_ 8) [1365], [^11^C]AZ10419369 (Agonist, Partial agonist) [1950]	[^3^H]eletriptan (Agonist) (p*K*_d_ 9.1) [1365], [^3^H]alniditan (Agonist) (p*K*_d_ 8.8–8.9) [1084], [^125^I]GTI (Selective Agonist) (p*K*_d_ 8.9) [193, 232] – Rat, [^3^H]GR 125,743 (Selective Antagonist) (p*K*_d_ 8.6) [2061], [^3^H]sumatriptan (Agonist) (p*K*_d_ 8.2) [1365]	[^3^H]5‐HT (Agonist) (p*K*_d_ 8.1–8.2) [1237, 1463]	[^3^H]LY334370 (Agonist) (p*K*_d_ 9.4) [1968], [^125^I]LSD (Agonist) (p*K*_d_ 9) [44] – Mouse
Comments	–	Wang *et al*. (2013) report X‐ray structures which reveal the binding modality of ergotamine and dihydroergotamine to the 5‐HT_1B_ receptor in comparison with the structure of the 5‐HT_2B_ receptor [1978].	–	–	–

**Table:** 

Nomenclature	5‐HT_2A_ receptor	5‐HT_2B_ receptor	5‐HT_2C_ receptor	5‐HT_4_ receptor
HGNC, UniProt	*HTR2A*, P28223	*HTR2B*, P41595	*HTR2C*, P28335	*HTR4*, Q13639
Agonists	DOI (p*K*_i_ 7.4–9.2) [204, 1374, 1755]	methysergide (Partial agonist) (p*K*_i_ 8–9.4) [970, 1605, 1969], DOI (p*K*_i_ 7.6–7.7) [1025, 1374, 1659]	DOI (p*K*_i_ 7.2–8.6) [465, 1374, 1659], Ro 60‐0175 (p*K*_i_ 7.7–8.2) [953, 970]	cisapride (Partial agonist) (p*K*_i_ 6.4–7.4) [77, 128, 597, 1266, 1267, 1941]
Selective agonists	–	BW723C86 (p*K*_i_ 7.3–8.6) [108, 970, 1659], Ro 60‐0175 (p*K*_i_ 8.3) [970]	WAY‐163909 (p*K*_i_ 6.7–8) [454], lorcaserin (p*K*_i_ 7.8) [1878]	TD‐8954 (p*K*_i_ 9.4) [1250], ML 10302 (Partial agonist) (p*K*_i_ 7.9–9) [136, 160, 1266, 1267, 1268], RS67506 (pEC_50_ 8.8) [731] – Rat, relenopride (Partial agonist) (p*K*_i_ 8.3) [607], velusetrag (p*K*_i_ 7.7) [1139, 1763], BIMU 8 (p*K*_i_ 7.3) [347]
Antagonists	risperidone (Inverse agonist) (p*K*_i_ 9.3–10) [986, 1008, 1675], mianserin (p*K*_i_ 7.7–9.6) [970, 1001, 1280], ziprasidone (p*K*_i_ 8.8–9.5) [986, 1008, 1675, 1711], volinanserin (pIC_50_ 6.5–9.3) [970, 1142, 1568], blonanserin (p*K*_i_ 9.1) [1421], clozapine (Inverse agonist) (p*K*_i_ 7.6–9) [970, 1008, 1277, 1675, 1943], olanzapine (p*K*_i_ 8.6–8.9) [986, 1008, 1675, 1711], nefazodone (p*K*_i_ 8.2) [1698], chlorpromazine (Inverse agonist) (p*K*_i_ 8.1) [1008], loxapine (Inverse agonist) (p*K*_i_ 8.1) [1008], trifluoperazine (p*K*_i_ 7.9) [1008], pimozide (p*K*_i_ 7.1–7.7) [986, 1008], trazodone (p*K*_i_ 7.4) [970], haloperidol (p*K*_i_ 6.7–7.3) [1008, 1277, 1675, 1711, 1943], mesoridazine (p*K*_i_ 7.3) [326], mirtazapine (p*K*_i_ 7.2) [513], mirtazapine (p*K*_i_ 7.2) [513], quetiapine (p*K*_i_ 6.4–7) [986, 1008], molindone (p*K*_i_ 6.5) [1008]	mianserin (p*K*_i_ 7.9–8.8) [180, 970, 1969]	mianserin (Inverse agonist) (p*K*_i_ 8.3–9.2) [524, 970, 1280], methysergide (p*K*_i_ 8.6–9.1) [465, 970], ziprasidone (Inverse agonist) (p*K*_i_ 7.9–9) [743, 1008, 1711], olanzapine (Inverse agonist) (p*K*_i_ 8.1–8.4) [743, 1008, 1711], loxapine (Inverse agonist) (p*K*_i_ 7.8–8) [743, 1008], mirtazapine (p*K*_i_ 7.4) [513], mirtazapine (p*K*_i_ 7.4) [513], trazodone (p*K*_i_ 6.6) [970], trifluoperazine (p*K*_i_ 6.4) [1008], agomelatine (p*K*_i_ 6.2) [1276]	–
Selective antagonists	ketanserin (p*K*_i_ 8.1–9.7) [234, 970, 1559], pimavanserin (Inverse agonist) (p*K*_i_ 9.3) [572, 1943]	BF‐1 (p*K*_i_ 10.1) [1671], RS‐127445 (p*K*_i_ 9–9.5) [180, 970], EGIS‐7625 (p*K*_i_ 9) [1001]	FR260010 (p*K*_i_ 9) [700], SB 242084 (p*K*_i_ 8.2–9) [928, 970], RS‐102221 (p*K*_i_ 8.3–8.4) [181, 970]	RS 100235 (p*K*_i_ 8.7–12.2) [347, 1589], SB 204070 (p*K*_i_ 9.8–10.4) [128, 1266, 1267, 1941], GR 113808 (p*K*_i_ 9.3–10.3) [77, 128, 160, 347, 1267, 1589, 1941]
Labelled ligands	[^3^H]fananserin (Antagonist) (p*K*_d_ 9.9) [1188] – Rat, [^3^H]ketanserin (Antagonist) (p*K*_d_ 8.6–9.7) [970, 1559], [^11^C]volinanserin (Antagonist) [676], [^18^F]altanserin (Antagonist) [1601]	[^3^H]LSD (Agonist) (p*K*_d_ 8.7) [1559], [^3^H]5‐HT (Agonist) (p*K*_d_ 8.1) [1967] – Rat, [^3^H]mesulergine (Antagonist, Inverse agonist) (p*K*_d_ 7.9) [970], [^125^I]DOI (Agonist) (p*K*_d_ 7.7–7.6)	[^125^I]DOI (Agonist) (p*K*_d_ 8.7–9) [524], [^3^H]mesulergine (Antagonist, Inverse agonist) (p*K*_d_ 9.3–8.7) [524, 1559], [^3^H]LSD (Agonist)	[^123^I]SB 207710 (Antagonist) (p*K*_d_ 10.1) [228] – Pig, [^3^H]GR 113808 (Antagonist) (p*K*_d_ 10.3–9.7) [77, 128, 1268, 1941], [^3^H]RS 57639 (Selective Antagonist) (p*K*_d_ 9.7) [179] – Guinea pig, [^11^C]SB207145 (Antagonist) (p*K*_d_ 8.6) [1169]
Comments	–	LSD (lysergic acid) and ergotamine show a strong preference for arrestin recruitment over G protein coupling at the 5‐HT_2B_ receptor, with no such preference evident at 5‐HT_1B_ receptors, and they also antagonise 5‐HT_7A_ receptors [1963]. DHE (dihydroergocryptine), pergolide and cabergoline also show significant preference for arrestin recruitment over G protein coupling at 5‐HT_2B_ receptors [1963].	The serotonin antagonist mesulergine was key to the discovery of the 5‐HT_2C_ receptor [1479].	–

**Table:** 

Nomenclature	5‐ht_5a_ receptor	5‐ht_5b_ receptor	5‐HT_6_ receptor	5‐HT_7_ receptor
HGNC, UniProt	*HTR5A*, P47898	*HTR5BP*, –	*HTR6*, P50406	*HTR7*, P34969
Selective agonists	–	–	WAY‐181187 (p*K*_i_ 8.7) [1663], E6801 (Partial agonist) (p*K*_i_ 8.7) [769], WAY‐208466 (p*K*_i_ 8.3) [135], EMD‐386088 (pIC_50_ 8.1) [1228]	LP‐12 (p*K*_i_ 9.9) [1082], LP‐44 (p*K*_i_ 9.7) [1082], LP‐211 (p*K*_i_ 9.2) [1083] – Rat, AS‐19 (p*K*_i_ 9.2) [947], E55888 (p*K*_i_ 8.6) [206]
Antagonists	–	–	–	lurasidone (p*K*_i_ 9.3) [829], pimozide (p*K*_i_ 9.3) [1604] – Rat, vortioxetine (p*K*_i_ 6.3) [90]
Selective antagonists	SB 699551 (p*K*_i_ 8.2) [366]	–	SB399885 (p*K*_i_ 9) [763], SB 271046 (p*K*_i_ 8.9) [224], cerlapirdine (p*K*_i_ 8.9) [358], SB357134 (p*K*_i_ 8.5) [225], Ro 63‐0563 (p*K*_i_ 7.9–8.4) [168, 1754]	SB269970 (p*K*_i_ 8.6–8.9) [1874], SB656104 (p*K*_i_ 8.7) [531], DR‐4004 (p*K*_i_ 8.7) [615, 938], JNJ‐18038683 (p*K*_i_ 8.2) [177], SB 258719 (Inverse agonist) (p*K*_i_ 7.5) [1875]
Labelled ligands	[^125^I]LSD (Agonist) (p*K*_d_ 9.7) [636], [^3^H]5‐CT (Agonist) (p*K*_d_ 8.6) [636]	[^125^I]LSD (Agonist) (p*K*_d_ 9.3) [1227] – Mouse, [^3^H]5‐CT (Agonist) [1965] – Mouse	[^11^C]GSK215083 (Antagonist) (p*K*_i_ 9.8) [1462], [^125^I]SB258585 (Selective Antagonist) (p*K*_d_ 9) [763], [^3^H]LSD (Agonist) (p*K*_d_ 8.7) [167], [^3^H]Ro 63‐0563 (Antagonist) (p*K*_d_ 8.3) [168], [^3^H]5‐CT (Agonist)	[^3^H]5‐CT (Agonist) (p*K*_d_ 9.4) [1874], [^3^H]5‐HT (Agonist) (p*K*_d_ 8.1–9) [93, 1793], [^3^H]SB269970 (Selective Antagonist) (p*K*_d_ 8.9) [1874], [^3^H]LSD (Agonist) (p*K*_d_ 8.5–8.6) [1793]

### Comments

Tabulated *p* *K*_i_ and *K*_D_ values refer to binding to human 5‐HT receptors unless indicated otherwise. The nomenclature of 5‐HT_1B_/5‐HT_1D_ receptors has been revised [707]. Only the non‐rodent form of the receptor was previously called 5‐HT_1D_: the human 5‐HT_1B_ receptor (tabulated) displays a different pharmacology to the rodent forms of the receptor due to Thr335 of the human sequence being replaced by Asn in rodent receptors. NAS181 is a selective antagonist of the rodent 5‐HT_1B_ receptor. Fananserin and ketanserin bind with high affinity to dopamine D4 and histamine H_1_ receptors respectively, and ketanserin is a potent *α*1 adrenoceptor antagonist, in addition to blocking 5‐HT_2A_ receptors. The human 5‐ht_5A_ receptor has been claimed to couple to several signal transduction pathways when stably expressed in C6 glioma cells [1404]. The human orthologue of the mouse 5‐ht_5b_ receptor is non‐functional due to interruption of the gene by stop codons. The 5‐ht_1e_ receptor appears not to have been cloned from mouse, or rat, impeding definition of its function. In addition to the receptors listed in the table, an 'orphan' receptor, unofficially termed 5‐HT_1P_, has been described [600].

### Further Reading

Bockaert J *et al*. (2011) 5‐HT(4) receptors, a place in the sun: act two. *Curr Opin Pharmacol* **11**: 87‐93 [PMID:21342787]

Codony X *et al*. (2011) 5‐HT(6) receptor and cognition. *Curr Opin Pharmacol* **11**: 94‐100 [PMID:21330210]

Hartig PR *et al*. (1996) Alignment of receptor nomenclature with the human genome: classification of 5‐HT1B and 5‐HT1D receptor subtypes. *Trends Pharmacol. Sci.* **17**: 103‐5 [PMID:8936345]

Hayes DJ *et al*. (2011) 5‐HT receptors and reward‐related behaviour: a review. *Neurosci Biobehav Rev* **35**: 1419‐49 [PMID:21402098]

Hoyer D *et al*. (1994) International Union of Pharmacology classification of receptors for 5‐hydroxytryptamine (Serotonin). *Pharmacol. Rev.* **46**: 157‐203 [PMID:7938165]

Leopoldo M *et al*. (2011) Serotonin 5‐HT7 receptor agents: Structure‐activity relationships and potential therapeutic applications in central nervous system disorders. *Pharmacol. Ther.* **129**: 120‐48 [PMID:20923682]

Meltzer HY *et al*. (2011) The role of serotonin receptors in the action of atypical antipsychotic drugs. *Curr Opin Pharmacol* **11**: 59‐67 [PMID:21420906]

Roberts AJ *et al*. (2012) The 5‐HT(7) receptor in learning and memory. *Hippocampus* **22**: 762‐71 [PMID:21484935]

Sargent BJ *et al*. (2011) Targeting 5‐HT receptors for the treatment of obesity. *Curr Opin Pharmacol* **11**: 52‐8 [PMID:21330209]

## Acetylcholine receptors (muscarinic)

### Overview

Muscarinic acetylcholine receptors (**nomenclature as agreed by the NC‐IUPHAR Subcommittee on Muscarinic Acetylcholine Receptors [** **275** **]**) are GPCRs of the Class A, rhodopsin‐like family where the endogenous agonist is acetylcholine. In addition to the agents listed in the table, AC‐42, its structural analogues AC‐260584 and 77‐LH‐28‐1, *N*‐desmethylclozapine, TBPB and LuAE51090 have been described as functionally selective agonists of the M_1_ receptor subtype *via* binding in a mode distinct from that utilized by non‐selective agonists [71, 878, 1040, 1041, 1232, 1635, 1786, 1787, 1825]. There are two pharmacologically characterised allosteric sites on muscarinic receptors, one defined by it binding gallamine, strychnine and brucine, and the other defined by the binding of KT 5720, WIN 62,577, WIN 51,708 and staurosporine[1052, 1053].

**Table:** 

Nomenclature	M_1_ receptor	M_2_ receptor
HGNC, UniProt	*CHRM1*, P11229	*CHRM2*, P08172
Agonists	carbachol (p*K*_i_ 3.2–5.3) [334, 846, 2040], pilocarpine (Partial agonist) (p*K*_i_ 5.1) [846], bethanechol (p*K*_i_ 4) [846]	bethanechol (p*K*_i_ 4) [846]
Antagonists	glycopyrrolate (pIC_50_ 9.9) [1801], umeclidinium (p*K*_i_ 9.8) [1035, 1632], AE9C90CB (p*K*_i_ 9.7) [1749], propantheline (p*K*_i_ 9.7) [797], atropine (p*K*_i_ 8.5–9.6) [334, 552, 759, 797, 1486, 1762], tiotropium (p*K*_i_ 9.6) [428], 4‐DAMP (p*K*_i_ 9.2) [458], dicyclomine (p*K*_i_ 9.1) [68], scopolamine (p*K*_i_ 9) [797], trihexyphenidyl (p*K*_i_ 8.9) [68], tripitramine (p*K*_i_ 8.8) [1176], UH‐AH 37 (p*K*_i_ 8.6–8.7) [609, 2012], tolterodine (p*K*_i_ 8.5–8.7) [609, 1749], oxybutynin (p*K*_i_ 8.6) [410, 818, 1749], darifenacin (p*K*_i_ 7.5–8.3) [609, 730, 759, 818, 1749], pirenzepine (p*K*_i_ 7.8–8.3) [238, 458, 730, 797, 875, 2012], solifenacin (p*K*_i_ 7.6) [818, 1749], AFDX384 (p*K*_i_ 7.5) [458], AQ‐RA 741 (p*K*_i_ 7.2–7.5) [458, 609], methoctramine (p*K*_i_ 6.6–7.3) [458, 493, 730, 1762], himbacine (p*K*_i_ 6.7–7.1) [458, 875, 1286], muscarinic toxin 3 (p*K*_i_ 7.1) [875], otenzepad (p*K*_d_ 6.2) [493]	tiotropium (p*K*_i_ 9.9) [428], umeclidinium (p*K*_i_ 9.8) [1035, 1632], propantheline (p*K*_i_ 9.5) [797], glycopyrrolate (Full agonist) (pIC_50_ 9.3) [1801], atropine (p*K*_i_ 7.8–9.2) [238, 310, 759, 797, 1002, 1373, 1486], AE9C90CB (p*K*_i_ 8.6) [1749], tolterodine (Inverse agonist) (p*K*_i_ 8.4–8.6) [609, 1373, 1749], AQ‐RA 741 (p*K*_i_ 8.4) [458, 609], himbacine (p*K*_i_ 7.9–8.4) [458, 875, 1002, 1286], methoctramine (p*K*_i_ 7.3–8.4) [238, 458, 493, 730, 1002, 1373], 4‐DAMP (p*K*_i_ 8.3) [1002], AFDX384 (p*K*_i_ 8.2) [458], biperiden (p*K*_d_ 8.2) [173], oxybutynin (p*K*_i_ 7.7–8.1) [818, 1749], darifenacin (Inverse agonist) (p*K*_i_ 7–7.6) [609, 730, 759, 818, 1373, 1749], UH‐AH 37 (p*K*_i_ 7.3–7.4) [609, 2012], otenzepad (p*K*_i_ 6.7–7.2) [238, 1002], solifenacin (p*K*_i_ 6.9–7.1) [818, 1749], pirenzepine (p*K*_i_ 6–6.7) [238, 458, 730, 797, 875, 1002, 1373, 2012], VU0255035 (p*K*_i_ 6.2) [1717], muscarinic toxin 3 (p*K*_i_<6) [875], guanylpirenzepine (p*K*_i_ 5.3) [1966] – Rat, muscarinic toxin 7 (p*K*_i_<5) [1414]
Selective antagonists	biperiden (p*K*_d_ 9.3) [173], VU0255035 (p*K*_i_ 7.8) [1717], guanylpirenzepine (p*K*_i_ 7.3–7.6) [23, 1966] – Rat	tripitramine (p*K*_i_ 9.6) [1176]
Allosteric modulators	muscarinic toxin 7 (Negative) (p*K*_i_ 11–11.1) [1414], benzoquinazolinone 12 (Positive) (p*K*_B_ 6.6) [4], KT 5720 (Positive) (p*K*_d_ 6.4) [1052], brucine (Positive) (p*K*_d_ 4.5–5.8) [846, 1051], BQCA (Positive) (p*K*_B_ 4–4.8) [4, 5, 261, 1161], VU0029767 (Positive) [1208], VU0090157 (Positive) [1208]	W‐84 (Negative) (p*K*_d_ 6–7.5) [1299, 1908], C_7_/3‐phth (Negative) (p*K*_d_ 7.1) [335], alcuronium (Negative) (p*K*_d_ 6.1–6.9) [846, 1908], gallamine (Negative) (p*K*_d_ 5.9–6.3) [348, 1049], LY2119620 (Positive) (p*K*_d_ 5.7) [383, 1010], LY2033298 (Positive) (p*K*_d_ 4.4) [1933]
Labelled ligands	[^3^H]QNB (Antagonist) (p*K*_d_ 10.6–10.8) [336, 1486], Cy3B‐telenzepine (Antagonist) (p*K*_d_ 10.5) [742], [^3^H]N‐methyl scopolamine (Antagonist) (p*K*_d_ 9.4–10.3) [280, 334, 336, 759, 846, 847, 875, 932, 1049], [^3^H](+)telenzepine (Antagonist) (p*K*_i_ 9.4) [500] – Rat, Alexa‐488‐telenzepine (Antagonist) (p*K*_d_ 9.3) [742], [^3^H]pirenzepine (Antagonist) (p*K*_d_ 7.9) [1995], BODIPY‐pirenzepine (Antagonist) (p*K*_i_ 7) [820], [^11^C]butylthio‐TZTP (Agonist) [504], [^11^C]xanomeline (Agonist) [504], [^18^F](R,R)‐quinuclidinyl‐4‐fluoromethyl‐benzilate (Antagonist) [935] – Rat	[^3^H]QNB (Antagonist) (p*K*_d_ 10.1–10.6) [1486], Cy3B‐telenzepine (Antagonist) (p*K*_i_ 10.4) [1380], [^3^H]tiotropium (Antagonist) (p*K*_d_ 10.3) [1632], [^3^H]N‐methyl scopolamine (Antagonist) (p*K*_d_ 9.3–9.9) [280, 310, 759, 846, 847, 875, 932, 1049, 1985], Alexa‐488‐telenzepine (Antagonist) (p*K*_i_ 8.8) [1380], [^3^H]acetylcholine (Agonist) (p*K*_d_ 8.8) [1050], [^3^H]oxotremorine‐M (Agonist) (p*K*_d_ 8.7) [137], [^3^H]dimethyl‐W84 (Allosteric modulator, Positive) (p*K*_d_ 8.5) [1908], [^18^F]FP‐TZTP (Agonist) [845] – Mouse

**Table:** 

Nomenclature	M_3_ receptor	M_4_ receptor	M_5_ receptor
HGNC, UniProt	*CHRM3*, P20309	*CHRM4*, P08173	*CHRM5*, P08912
Agonists	pilocarpine (Partial agonist) (p*K*_i_ 5.1) [846], carbachol (p*K*_i_ 4–4.4) [310, 846, 2040], bethanechol (p*K*_i_ 4.2) [846]	pilocarpine (Partial agonist) (p*K*_i_ 5.2) [846], carbachol (p*K*_i_ 4.3–4.9) [846, 2040], bethanechol (p*K*_i_ 4) [846]	pilocarpine (Partial agonist) (p*K*_i_ 5) [639], carbachol (p*K*_i_ 4.9) [2040]
Antagonists	tiotropium (p*K*_i_ 9.5–11.1) [428, 442], umeclidinium (p*K*_i_ 10.2) [1035, 1632], propantheline (p*K*_i_ 10) [797], AE9C90CB (p*K*_i_ 9.9) [1749], atropine (p*K*_i_ 8.9–9.8) [238, 442, 759, 797, 1486, 1762], ipratropium (p*K*_i_ 9.3–9.8) [442, 759], aclidinium (pIC_50_ 9.8) [1530], clidinium (p*K*_i_ 9.6) [442], glycopyrrolate (pIC_50_ 9.6) [1801], 4‐DAMP (p*K*_i_ 9.3) [458], darifenacin (p*K*_i_ 8.6–9.1) [609, 730, 759, 818, 1749], dicyclomine (p*K*_i_ 9) [68], oxybutynin (p*K*_i_ 8.8–8.9) [410, 818, 1749], tolterodine (p*K*_i_ 8.4–8.5) [609, 1749], biperiden (p*K*_d_ 8.4) [173], UH‐AH 37 (p*K*_i_ 8.1–8.2) [609, 2012], solifenacin (p*K*_i_ 7.7–8) [818, 1749], tropicamide (p*K*_i_ 7.5) [68], AQ‐RA 741 (p*K*_i_ 7.2–7.3) [458, 609], AFDX384 (p*K*_i_ 7.2) [458], himbacine (p*K*_i_ 6.9–7.2) [458, 875, 1286], methoctramine (p*K*_i_ 6.1–6.9) [238, 458, 493, 730, 1762], tripitramine (p*K*_i_ 6.8) [1288], pirenzepine (p*K*_i_ 6.5–6.8) [238, 458, 730, 797, 875, 2012], guanylpirenzepine (p*K*_i_ 6.2) [1966] – Rat, VU0255035 (p*K*_i_ 6.1) [1717], otenzepad (p*K*_i_ 6.1) [238], muscarinic toxin 3 (p*K*_i_<6) [875], muscarinic toxin 7 (p*K*_i_<5) [1414]	umeclidinium (p*K*_i_ 10.3) [1632], glycopyrrolate (pIC_50_ 9.8) [1801], AE9C90CB (p*K*_i_ 9.5) [1749], 4‐DAMP (p*K*_i_ 8.9) [458], oxybutynin (p*K*_i_ 8.7) [1749], biperiden (p*K*_d_ 8.6) [173], UH‐AH 37 (p*K*_i_ 8.3–8.4) [609, 2012], tolterodine (p*K*_i_ 8.3–8.4) [609, 1749], AQ‐RA 741 (p*K*_i_ 7.8–8.2) [458, 609], himbacine (p*K*_i_ 7.9–8.2) [458, 875, 1286], darifenacin (p*K*_i_ 7.3–8.1) [609, 730, 759, 1749], AFDX384 (p*K*_i_ 8) [458], tripitramine (p*K*_i_ 7.9) [1288], pirenzepine (p*K*_i_ 7–7.6) [458, 730, 797, 875, 2012], methoctramine (p*K*_i_ 6.6–7.5) [238, 458, 493, 730], otenzepad (p*K*_d_ 7) [493], solifenacin (p*K*_i_ 6.8) [1749], guanylpirenzepine (p*K*_i_ 6.2) [1966] – Rat, VU0255035 (p*K*_i_ 5.9) [1717], muscarinic toxin 7 (p*K*_i_<5) [1414]	umeclidinium (p*K*_i_ 9.9) [1632], glycopyrrolate (pIC_50_ 9.7) [1801], AE9C90CB (p*K*_i_ 9.5) [1749], 4‐DAMP (p*K*_i_ 9) [458], tolterodine (p*K*_i_ 8.5–8.8) [609, 1749], darifenacin (p*K*_i_ 7.9–8.6) [609, 730, 759, 1749], UH‐AH 37 (p*K*_i_ 8.3) [609, 2012], biperiden (p*K*_d_ 8.2) [173], oxybutynin (p*K*_i_ 7.9) [1749], AQ‐RA 741 (p*K*_i_ 6.1–7.8) [458, 609], tripitramine (p*K*_i_ 7.5) [1176], methoctramine (p*K*_i_ 6.3–7.2) [238, 458, 493, 730], solifenacin (p*K*_i_ 7.2) [1749], pirenzepine (p*K*_i_ 6.8–7.1) [730, 875, 2012], guanylpirenzepine (p*K*_d_ 6.8) [514] – Unknown, himbacine (p*K*_i_ 5.4–6.5) [458, 875, 1286], AFDX384 (p*K*_i_ 6.3) [458], muscarinic toxin 3 (p*K*_i_<6) [875], otenzepad (p*K*_i_ 5.6) [238], muscarinic toxin 7 (p*K*_i_<5) [1414]
Selective antagonists	–	–	ML381 (p*K*_i_ 6.3) [593]
Allosteric modulators	WIN 62,577 (Positive) (p*K*_d_ 5.1) [1053], N‐chloromethyl‐brucine (Positive) (p*K*_d_ 3.3) [1051]	muscarinic toxin 3 (Negative) (p*K*_i_ 8.7) [875, 1444], LY2119620 (Positive) (p*K*_d_ 5.7) [383], thiochrome (Positive) (p*K*_d_ 4) [1050], LY2033298 (Positive) [286], VU0152099 (Positive) [201], VU0152100 (Positive) [201]	ML380 (Positive) (pEC_50_ 6.7) [595]
Selective allosteric modulators	–	–	ML375 (Negative) (pIC_50_ 6.5) [594]
Labelled ligands	[^3^H]tiotropium (Antagonist) (p*K*_d_ 10.7) [1632], [^3^H]QNB (Antagonist) (p*K*_d_ 10.4) [1486], [^3^H]N‐methyl scopolamine (Antagonist) (p*K*_d_ 9.7–10.2) [280, 310, 759, 797, 846, 875, 932, 1049], [^3^H]darifenacin (Antagonist) (p*K*_d_ 9.5) [1762]	[^3^H]QNB (Antagonist) (p*K*_d_ 9.7–10.5) [336, 1486], [^3^H]N‐methyl scopolamine (Antagonist) (p*K*_d_ 9.9–10.2) [280, 310, 336, 759, 846, 875, 932, 1049, 1444, 1985], [^3^H]acetylcholine (Agonist) (p*K*_d_ 8.2) [1050]	[^3^H]QNB (Antagonist) (p*K*_d_ 10.2–10.7), [^3^H]N‐methyl scopolamine (Antagonist) (p*K*_d_ 9.3–9.7) [280, 310, 759, 875, 932, 1985]

### Comments

LY2033298 and BQCA have also been shown to directly activate the M_4_ and M_1_ receptors, respectively, via an allosteric site [1059, 1060, 1366, 1367]. The allosteric site for gallamine and strychnine on M_2_ receptors can be labelled by [^3^H]dimethyl‐W84[1908]. McN‐A‐343 is a functionally selective partial agonist that appears to interact in a bitopic mode with both the orthosteric and an allosteric site on the M_2_ muscarinic receptor [1934]. THRX160209, hybrid 1 and hybrid 2, are multivalent (bitopic) ligands that also achieve selectivity for M_2_ receptors by binding both to the orthosteric and a nearby allosteric site [52, 1796].

Although numerous ligands for muscarinic acetylcholine receptors have been described, relatively few selective antagonists have been described, so it is common to assess the rank order of affinity of a number of antagonists of limited selectivity (*e.g.* 4‐DAMP, darifenacin, pirenzepine) in order to identify the involvement of particular subtypes. It should be noted that the measured affinities of antagonists (and agonists) in radioligand binding studies are sensitive to ionic strength and can increase over 10‐fold at low ionic strength compared to its value at physiological ionic strengths [151].

### Further Reading

Caulfield MP *et al*. (1998) International Union of Pharmacology. XVII. Classification of muscarinic acetylcholine receptors. *Pharmacol. Rev.* **50**: 279‐290 [PMID:9647869]

Eglen RM. (2012) Overview of muscarinic receptor subtypes. *Handb Exp Pharmacol* 3‐28 [PMID:22222692]

Gregory KJ *et al*. (2007) Allosteric modulation of muscarinic acetylcholine receptors. *Curr Neuropharmacol* **5**: 157‐67 [PMID:19305798]

Kruse AC *et al*. (2014) Muscarinic acetylcholine receptors: novel opportunities for drug development. *Nat Rev Drug Discov* **13**: 549‐60 [PMID:24903776]

Leach K *et al*. (2012) Structure‐function studies of muscarinic acetylcholine receptors. *Handb Exp Pharmacol* 29‐48 [PMID:22222693]

Valant C *et al*. (2012) The best of both worlds? Bitopic orthosteric/allosteric ligands of g protein‐coupled receptors. *Annu. Rev. Pharmacol. Toxicol.* **52**: 153‐78 [PMID:21910627]

## Adenosine receptors

### Overview

Adenosine receptors (**nomenclature as agreed by the NC‐IUPHAR Subcommittee on Adenosine Receptors [** **541** **]**) are activated by the endogenous ligand adenosine(potentially inosine also at A_3_ receptors). Crystal structures for the antagonist‐bound and agonist‐bound A_2A_ adenosine receptors have been described [835, 2065].

**Table:** 

Nomenclature	A_1_ receptor	A_2A_ receptor	A_2B_ receptor	A_3_ receptor
HGNC, UniProt	*ADORA1*, P30542	*ADORA2A*, P29274	*ADORA2B*, P29275	*ADORA3*, P0DMS8
Agonists	cyclopentyladenosine (p*K*_i_ 6.5–9.4) [388, 570, 736, 839, 870, 1592, 2141]	–	–	–
(Sub)family‐selective agonists	NECA (p*K*_i_ 5.3–8.2) [570, 870, 1592, 1903, 2077]	NECA (p*K*_i_ 6.9–8.7) [189, 427, 570, 943, 1021, 2077]	NECA (p*K*_i_ 5.7–6.9) [146, 189, 862, 1113, 1798, 1945, 2077]	NECA (p*K*_i_ 7.5–8.4) [189, 570, 840, 1634, 1946, 2077]
Selective agonists	5‐Cl‐5‐deoxy‐(±)‐ENBA (p*K*_i_ 9.3) [536], GR79236 (p*K*_i_ 8.5) [839] – Rat, CCPA (p*K*_i_ 7.7–8.1) [839, 1419]	apadenoson (p*K*_i_ 9.3) [1481], UK‐432,097 (p*K*_i_ 8.3) [2065], CGS 21680 (p*K*_i_ 6.7–8.1) [189, 427, 570, 839, 943, 968, 1021, 1419], regadenoson (p*K*_i_ 6.5) [839]	BAY 60‐6583 (p*K*_i_ 8–8.5) [460]	IB‐MECA (p*K*_i_ 8.7–9.2) [511, 561, 968, 1946], Cl‐IB‐MECA (p*K*_i_ 8–8.9) [202, 840, 941], MRS5698 (p*K*_i_ 8.5) [1898]
Antagonists	caffeine (p*K*_i_ 4.3–5) [6, 409, 838]	SCH 58261 (p*K*_i_ 8.3–9.2) [427, 1021, 1445], theophylline (p*K*_i_ 5.2–5.8) [427, 838, 968, 1945], caffeine (p*K*_i_ 4.6–5.6) [6, 838, 1021]	theophylline (p*K*_i_ 4.1–5) [141, 511, 944, 1945], caffeine (p*K*_i_ 4.5–5) [141, 188, 944]	caffeine (p*K*_i_ 4.9) [838]
(Sub)family‐selective antagonists	CGS 15943 (p*K*_i_ 8.5) [1445], xanthine amine congener (p*K*_d_ 7.5) [536]	CGS 15943 (p*K*_i_ 7.7–9.4) [427, 943, 968, 1445], xanthine amine congener (p*K*_i_ 8.4–9) [427, 968]	xanthine amine congener (p*K*_i_ 6.9–8.8) [146, 862, 863, 968, 1113, 1798], CGS 15943 (p*K*_i_ 6–8.1) [65, 862, 863, 968, 1445, 1798]	CGS 15943 (p*K*_i_ 7–7.9) [949, 968, 1445, 1946], xanthine amine congener (p*K*_i_ 7–7.4) [968, 1634, 1946]
Selective antagonists	PSB36 (p*K*_i_ 9.9) [6] – Rat, DPCPX (p*K*_i_ 7.4–9.2) [826, 1419, 1592, 2015, 2141], derenofylline (p*K*_i_ 9) [897], WRC‐0571 (p*K*_i_ 8.8) [1210]	SCH442416 (p*K*_i_ 8.4–10.3) [1728, 1891], ZM‐241385 (p*K*_i_ 8.8–9.1) [1445]	PSB‐0788 (p*K*_i_ 9.4) [188], PSB603 (p*K*_i_ 9.3) [188], MRS1754 (p*K*_i_ 8.8) [862, 948], PSB1115 (p*K*_i_ 7.3) [723]	MRS1220 (p*K*_i_ 8.2–9.2) [840, 949, 1818, 2090], VUF5574 (p*K*_i_ 8.4) [2143], MRS1523 (p*K*_i_ 7.7) [1092], MRS1191 (p*K*_i_ 7.5) [840, 866, 1097]
Labelled ligands	[^3^H]CCPA (Agonist) (p*K*_d_ 9.2) [968, 1592], [^3^H]DPCPX (Antagonist) (p*K*_d_ 8.4–9.2) [388, 511, 968, 1445, 1592, 1903]	[^3^H]ZM 241385 (Antagonist) (p*K*_d_ 8.7–9.1) [36, 569], [^3^H]CGS 21680 (Agonist) (p*K*_d_ 7.7–7.8) [852, 1976]	[^3^H]MRS1754 (Antagonist) (p*K*_d_ 8.8) [862]	[^125^I]AB‐MECA (Agonist) (p*K*_d_ 9–9.1) [1445, 1946]

### Comments

Adenosine inhibits many intracellular ATP‐utilising enzymes, including adenylyl cyclase (P‐site). A pseudogene exists for the A_2B_ adenosine receptor (*ADORA2BP1*) with 79% identity to the A_2B_ adenosine receptor cDNA coding sequence, but which is unable to encode a functional receptor [841]. DPCPX also exhibits antagonism at A_2B_ receptors (p*K*_i_ ca. 7,[34, 968]). Antagonists at A_3_ receptors exhibit marked species differences, such that only MRS1523 and MRS1191 are selective at the rat A_3_ receptor. In the absence of other adenosine receptors, [^3^H]DPCPX and [^3^H]ZM 241385 can also be used to label A_2B_ receptors (K_D_ *ca*. 30 and 60 nM respectively). [^125^I]AB‐MECA also binds to A_1_ receptors [968]. [^3^H]CGS 21680 is relatively selective for A_2A_ receptors, but may also bind to other sites in cerebral cortex [384, 871]. [^3^H]NECA binds to other non‐receptor elements, which also recognise adenosine [1143]. XAC‐BY630 has been described as a fluorescent antagonist for labelling A_1_ adenosine receptors in living cells, although activity at other adenosine receptors was not examined [212].

### Further Reading

Fredholm BB *et al*. (2011) International Union of Basic and Clinical Pharmacology. LXXXI. Nomenclature and classification of adenosine receptors–an update. *Pharmacol. Rev.* **63**: 1‐34 [PMID:21303899]

Göblyös A *et al*. (2011) Allosteric modulation of adenosine receptors. *Biochim. Biophys. Acta* **1808**: 1309‐18 [PMID:20599682]

Headrick JP *et al*. (2011) Adenosine and its receptors in the heart: regulation, retaliation and adaptation. *Biochim. Biophys. Acta* **1808**: 1413‐28 [PMID:21094127]

Lasley RD. (2011) Adenosine receptors and membrane microdomains. *Biochim. Biophys. Acta* **1808**: 1284‐9 [PMID:20888790]

Mundell S *et al*. (2011) Adenosine receptor desensitization and trafficking. *Biochim. Biophys. Acta* **1808**: 1319‐28 [PMID:20550943]

Ponnoth DS *et al*. (2011) Adenosine receptors and vascular inflammation. *Biochim. Biophys. Acta* **1808**: 1429‐34 [PMID:20832387]

Wei CJ *et al*. (2011) Normal and abnormal functions of adenosine receptors in the central nervous system revealed by genetic knockout studies. *Biochim. Biophys. Acta* **1808**: 1358‐79 [PMID:21185258]

## Adhesion Class GPCRs

### Overview

Adhesion GPCRs are structurally identified on the basis of a large extracellular region, similar to the Class B GPCR, but which is linked to the 7TM region by a "stalk" motif containing a GPCR proteolytic site. The N‐terminus often shares structural homology with proteins such as lectins and immunoglobulins, leading to the term adhesion GPCR [543, 2097]. **The nomenclature of these receptors was revised in 2015 as recommended by NC‐IUPHAR and the Adhesion GPCR Consortium** [683].

**Table:** 

Nomenclature	ADGRA1	ADGRA2	ADGRA3	ADGRB1	ADGRB2
HGNC, UniProt	*ADGRA1*, Q86SQ6	*ADGRA2*, Q96PE1	*ADGRA3*, Q8IWK6	*ADGRB1*, O14514	*ADGRB2*, O60241
Endogenous agonists	–	–	–	phosphatidylserine [1461]	–
Comments	–	–	–	ADGRB1 is reported to respond to phosphatidylserine [1461].	–

**Table:** 

Nomenclature	ADGRB3	*CELSR1*	*CELSR2*	*CELSR3*	ADGRD1
HGNC, UniProt	*ADGRB3*, O60242	*CELSR1*, Q9NYQ6	*CELSR2*, Q9HCU4	*CELSR3*, Q9NYQ7	*ADGRD1*, Q6QNK2

**Table:** 

Nomenclature	ADGRD2	ADGRE1	ADGRE2	ADGRE3	ADGRE4P
HGNC, UniProt	*ADGRD2*, Q7Z7M1	*ADGRE1*, Q14246	*ADGRE2*, Q9UHX3	*ADGRE3*, Q9BY15	*ADGRE4P*, Q86SQ3

**Table:** 

Nomenclature	ADGRE5	ADGRF1	ADGRF2	ADGRF3	ADGRF4
HGNC, UniProt	*ADGRE5*, P48960	*ADGRF1*, Q5T601	*ADGRF2*, Q8IZF7	*ADGRF3*, Q8IZF5	*ADGRF4*, Q8IZF3

**Table:** 

Nomenclature	ADGRF5	ADGRG1	ADGRG2	ADGRG3	ADGRG4
HGNC, UniProt	*ADGRF5*, Q8IZF2	*ADGRG1*, Q9Y653	*ADGRG2*, Q8IZP9	*ADGRG3*, Q86Y34	*ADGRG4*, Q8IZF6
Comments	–	Reported to bind tissue transglutaminase 2 [2066] and collagen, which activates the G_12/13_ pathway [1155].	–	–	–

**Table:** 

Nomenclature	ADGRG5	ADGRG6	ADGRG7	ADGRL1
HGNC, UniProt	*ADGRG5*, Q8IZF4	*ADGRG6*, Q86SQ4	*ADGRG7*, Q96K78	*ADGRL1*, O94910

**Table:** 

Nomenclature	ADGRL2	ADGRL3	ADGRL4	ADGRV1
HGNC, UniProt	*ADGRL2*, O95490	*ADGRL3*, Q9HAR2	*ADGRL4*, Q9HBW9	*ADGRV1*, Q8WXG9
Comments	–	–	–	Loss‐of‐function mutations are associated with Usher syndrome, a sensory deficit disorder [843].

## Adrenoceptors

### Overview

#### **Adrenoceptors, *α*_1_**

*α*_1_‐Adrenoceptors (**nomenclature as agreed by the NC‐IUPHAR Subcommittee on Adrenoceptors [** **248** **], see also [** **752** **]**) are activated by the endogenous agonists (‐)‐adrenaline and (‐)‐noradrenaline with equal potency. Phenylephrine, methoxamine and cirazoline are agonists selective for *α*_1_‐adrenoceptors relative to *α*_2_‐adrenoceptors, while prazosin (8.5‐10.5) and corynanthine(6.5‐7.5) are antagonists considered selective for *α*_1_‐adrenoceptors relative to *α*_2_‐adrenoceptors. [^3^H]prazosin(0.25 nM) and [^125^I]HEAT(0.1 nM; also known as BE2254) are relatively selective radioligands. The *α*_1A_‐adrenoceptor antagonist S(+)‐niguldipine also has high affinity for L‐type Ca^2+^ channels. The conotoxin rho‐TIA acts as a negative allosteric modulator at the *α*_1B_‐adrenoceptor [1716], while the snake toxin *ρ*‐Da1a acts as a selective non‐competitive antagonist at the *α*_1A_‐adrenoceptor [1236, 1548]. Fluorescent derivatives of prazosin (Bodipy PL‐prazosin‐QAPB) are increasingly used to examine cellular localisation of *α*_1_‐adrenoceptors. The vasoconstrictor effects of selective *α*1‐adrenoceptor agonists have led to their use as nasal decongestants; antagonists are used to treat hypertension (doxazosin, prazosin) and benign prostatic hyperplasia (alfuzosin, tamsulosin). The combined *α*_1_‐ and *β*_2_‐adrenoceptor antagonist carvedilol is widely used to treat congestive heart failure, although the contribution of *α*_1_‐adrenoceptor blockade to the therapeutic effect is unclear. Several anti‐depressants and anti‐psychotic drugs possess *α*_1_‐adrenoceptor blocking properties that are believed to contribute to side effects such as orthostatic hypotension and extrapyramidal effects.

**Table:** 

Nomenclature	*α*_1A_‐adrenoceptor	*α*_1B_‐adrenoceptor	*α*_1D_‐adrenoceptor
HGNC, UniProt	*ADRA1A*, P35348	*ADRA1B*, P35368	*ADRA1D*, P25100
Endogenous agonists	–	–	(‐)‐adrenaline (p*K*_i_ 7.2) [1722]
Agonists	oxymetazoline (p*K*_i_ 8–8.2) [780, 1420, 1722, 1862], phenylephrine (p*K*_i_ 5.2–5.4) [1862], methoxamine (p*K*_i_ 5–5.2) [1722, 1862]	phenylephrine (pIC_50_ 6.3–7.5) [532, 1289]	–
Selective agonists	A61603 (pIC_50_ 7.8–8.4) [532, 969], dabuzalgron (p*K*_i_ 7.4) [162]	–	–
Antagonists	prazosin (Inverse agonist) (p*K*_i_ 9–9.9) [289, 389, 532, 1722, 2029], doxazosin (p*K*_i_ 9.3) [689], terazosin (p*K*_i_ 8.7) [1263], phentolamine (p*K*_i_ 8.6) [1722], alfuzosin (p*K*_i_ 8.1) [750]	prazosin (Inverse agonist) (p*K*_i_ 9.6–9.9) [532, 1722, 2029], tamsulosin (Inverse agonist) (p*K*_i_ 9.5–9.7) [532, 1722, 2029], doxazosin (p*K*_i_ 9.1) [689], alfuzosin (p*K*_i_ 8.6) [751], terazosin (p*K*_i_ 8.6) [1263], phentolamine (p*K*_i_ 7.5) [1722]	prazosin (Inverse agonist) (p*K*_i_ 9.5–10.2) [532, 1722, 2029], tamsulosin (p*K*_i_ 9.8–10.2) [532, 1722, 2029], doxazosin (p*K*_i_ 9.1) [689], terazosin (p*K*_i_ 9.1) [1263], alfuzosin (p*K*_i_ 8.4) [750], dapiprazole (p*K*_i_ 8.4) [68], phentolamine (Inverse agonist) (p*K*_i_ 8.2) [1722], RS‐100329 (p*K*_i_ 7.9) [2029], labetalol (p*K*_i_ 6.6) [68]
Selective antagonists	tamsulosin (p*K*_i_ 10–10.7) [289, 389, 532, 1722, 2029], silodosin (p*K*_i_ 10.4) [1722], S(+)‐niguldipine (p*K*_i_ 9.1–10) [532, 1722], RS‐100329 (p*K*_i_ 9.6) [2029], SNAP5089 (p*K*_i_ 8.8–9.4) [750, 1081, 2014], *ρ*‐Da1a (p*K*_i_ 9.2–9.3) [1236, 1548], RS‐17053 (p*K*_i_ 9.2–9.3) [289, 389, 529, 532]	Rec 15/2615 (p*K*_i_ 9.5) [1867], L‐765314 (p*K*_i_ 7.7) [1470], AH 11110 (p*K*_i_ 7.5) [1652]	BMY‐7378 (p*K*_i_ 8.7–9.1) [268, 2102]

#### **Adrenoceptors, *α*_2_**

*α*_2_‐Adrenoceptors (**nomenclature as agreed by NC‐IUPHAR Subcommittee on Adrenoceptors; [** **248** **]**) are activated by endogenous agonists with a relative potency of (‐)‐adrenaline>(‐)‐noradrenaline. Brimonidine and talipexole are agonists selective for *α*_2_‐adrenoceptors relative to *α*_1_‐adrenoceptors, rauwolscine and yohimbine are antagonists selective for *α*_2_‐adrenoceptors relative to *α*_1_‐adrenoceptors. [^3^H]rauwolscine(1 nM), [^3^H]brimonidine(5 nM) and [^3^H]RX821002(0.5 nM and 0.1 nM at *α*_2C_) are relatively selective radioligands. There is species variation in the pharmacology of the *α*_2A_‐adrenoceptor; for example, yohimbine, rauwolscine and oxymetazoline have an 20‐fold higher affinity for the human *α*_2A_‐adrenoceptor compared to the rat, mouse and bovine receptor. These *α*_2A_ orthologues are sometimes referred to as *α*_2D_‐adrenoceptors. Multiple mutations of *α*_2_‐adrenoceptors have been described, some of which are associated with alterations in function. Presynaptic *α*_2_‐adrenoceptors are widespread in the nervous system and regulate many functions, hence the multiplicity of actions. The effects of classical (not subtype selective) *α*_2_‐adrenoceptor agonists such as clonidine, guanabenz and brimonidine on central baroreflex control (hypotension and bradycardia), as well as their ability to induce hypnotic effects and analgesia, and their ability to modulate seizure activity and platelet aggregation are mediated by *α*_2A_‐adrenoceptors. Clonidine has been used as an anti‐hypertensive and also to counteract opioid withdrawal. Actions on imidazoline recognition sites may contribute to the pharmacological effects of clonidine. *α*_2_‐Adrenoceptor agonists such as dexmedetomidine have been widely used as sedatives and analgesics in veterinary medicine (also xylazine) and are now used frequently in humans. Dexmedetomidine also has analgesic, sympatholytic and anxiolytic properties but is notable for the production of sedation without respiratory depression. *α*_2_‐Adrenoceptor antagonists are relatively little used therapeutically although yohimbine has been used to treat erectile dysfunction and several anti‐depressants (e.g. Mirtazapine) that block *α*_2_‐adrenoceptors may work through this mechanism. The roles of *α*_2B_ and *α*_2C_‐adrenoceptors are less clear but the *α*_2B_ subtype appears to be involved in neurotransmission in the spinal cord and *α*_2C_ in regulating catecholamine release from adrenal chromaffin cells.

**Table:** 

Nomenclature	*α*_2A_‐adrenoceptor	*α*_2B_‐adrenoceptor	*α*_2C_‐adrenoceptor
HGNC, UniProt	*ADRA2A*, P08913	*ADRA2B*, P18089	*ADRA2C*, P18825
Endogenous agonists	(‐)‐adrenaline (p*K*_i_ 5.6–8.3) [854, 1503]	(‐)‐noradrenaline (Partial agonist) (p*K*_i_ 5.6–9.1) [854, 1484, 1503]	(‐)‐noradrenaline (p*K*_i_ 5.9–8.7) [854, 1484, 1503]
Agonists	dexmedetomidine (Partial agonist) (p*K*_i_ 7.6–9.6) [854, 1163, 1484, 1503], clonidine (Partial agonist) (p*K*_i_ 7.2–9.2) [854, 1484, 1503], brimonidine (p*K*_i_ 6.7–8.7) [854, 1163, 1484, 1503], apraclonidine (p*K*_i_ 8.5) [1343], guanabenz (pIC_50_ 7.4) [68], guanfacine (Partial agonist) (p*K*_i_ 7.1–7.3) [854, 1165]	dexmedetomidine (p*K*_i_ 7.5–9.7) [854, 1163, 1484, 1503], clonidine (Partial agonist) (p*K*_i_ 6.7–9.5) [854, 1484, 1503], brimonidine (Partial agonist) (p*K*_i_ 6–8.3) [854, 1484, 1503], guanabenz (pIC_50_ 6.8) [68], guanfacine (p*K*_i_ 5.8–6.5) [854]	dexmedetomidine (p*K*_i_ 7–9.3) [854, 1484, 1503], brimonidine (Partial agonist) (p*K*_i_ 5.7–7.6) [854, 1163, 1484, 1503], apraclonidine (p*K*_i_ 7.5) [1343], guanfacine (Partial agonist) (p*K*_i_ 5.4–6.2) [854], guanabenz (pIC_50_ 6) [68]
Selective agonists	oxymetazoline (Partial agonist) (p*K*_i_ 8–8.6) [854, 1163, 1922]	–	–
Antagonists	yohimbine (p*K*_i_ 8.5–9.5) [247, 416, 1922], WB 4101 (p*K*_i_ 8.4–9.4) [247, 416, 1922], spiroxatrine (p*K*_i_ 9) [1922], mirtazapine (p*K*_i_ 7.7) [513], tolazoline (p*K*_i_ 5.4) [854]	yohimbine (p*K*_i_ 8.4–9.2) [247, 416, 1922]	yohimbine (p*K*_i_ 7.9–8.9) [247, 416, 1922], phenoxybenzamine (p*K*_i_ 8.5) [2002], tolazoline (p*K*_i_ 5.5) [854]
Selective antagonists	BRL 44408 (p*K*_i_ 8.2–8.8) [1922, 2104]	imiloxan (p*K*_i_ 7.3) [1269] – Rat	JP1302 (p*K*_B_ 7.8) [1631]
Labelled ligands	–	–	[^3^H]MK‐912 (Antagonist) (p*K*_d_ 10.1) [1922]

#### **Adrenoceptors, *β***

*β*‐Adrenoceptors (**nomenclature as agreed by the NC‐IUPHAR Subcommittee on Adrenoceptors, [** **248** **]**) are activated by the endogenous agonists (‐)‐adrenaline and (‐)‐noradrenaline. Isoprenaline is a synthetic agonist selective for *β*‐adrenoceptors relative to *α*_1_‐ and *α*_2_‐adrenoceptors, while for *β*_1_ and *β*_2_ adrenoceptors, propranolol(pK_i_8.2‐9.2) and cyanopindolol(pK_i_ 10.0‐11.0) are relatively selective antagonists. (‐)‐noradrenaline, xamoterol and (‐)‐Ro 363 are agonists that show selectivity for *β*_1_‐ relative to *β*_2_‐adrenoceptors. Pharmacological differences exist between human and mouse *β*_3_‐adrenoceptors, and the 'rodent selective' agonists BRL 37344 and CL316243 have low efficacy at the human *β*_3_‐adrenoceptor whereas CGP 12177 and L 755507 activate human *β*_3_‐adrenoceptors [1649]. *β*_3_‐Adrenoceptors are relatively resistant to blockade by propranolol(pK_i_5.8‐7.0), but can be blocked by high concentrations of bupranolol(pK_i_8.65 [1650]). SR59230A has reasonably high affinity at *β*_3_‐adrenoceptors [1197], but does not discriminate well between the three *β*‐adrenoceptor subtypes [262] and has been reported to have lower affinity for the *β*_3_‐adrenoceptor in some circumstances [913]. L‐748337 is the most selective antagonist for *β*_3_ adrenoceptors. [^125^I]‐cyanopindolol, [^125^I]‐hydroxybenzylpindolol and [^3^H]‐alprenolol are high affinity radioligands widely used to label *β*_1_‐ and *β*_2_‐adrenoceptors and *β*_3_‐adrenoceptors can be labelled with higher concentrations (nM) of [^125^I]‐cyanopindolol in the presence of appropriate concentrations of *β*_1_‐ and *β*_2_‐adrenoceptor antagonists. [^3^H]L‐748337 is a *β*_3_‐selective radioligand. Fluorescent ligands such as BODIPY‐TMR‐CGP12177 are also increasingly being used to track *β*‐adrenoceptors at the cellular level [86]. Somewhat selective *β*_1_‐adrenoceptor selective agonists (denopamine, dobutamine) are used short‐term to treat cardiogenic shock but, in the longer term, reduce survival. *β*_1_‐Adrenoceptor‐preferring antagonists are used to treat hypertension (atenolol, betaxolol, bisoprolol, metoprolol and nebivolol), cardiac arrhythmias (atenolol, bisoprolol, esmolol) and cardiac failure (metoprolol, nebivolol). Cardiac failure is also succesfully treated with carvedilol which blocks both *β*_1_‐ and *β*_2_‐adrenoceptors, as well as *α*_1_‐adrenoceptors. *β*_2_‐Adrenoceptor‐selective agonists are powerful bronchodilators widely used to treat respiratory disorders. There are both short (salbutamol, terbutaline) and long acting drugs (formoterol, salmeterol). Although many first generation *β*‐adrenoceptor antagonists (propranolol) block both *β*_1_‐ and *β*_2_‐adrenoceptors there are no *β*_2_‐adrenoceptor‐selective antagonists used therapeutically. The *β*_3_‐adrenoceptor agonist mirabegron is used to control overactive bladder syndrome.

**Table:** 

Nomenclature	*β*_1_‐adrenoceptor	*β*_2_‐adrenoceptor	*β*_3_‐adrenoceptor
HGNC, UniProt	*ADRB1*, P08588	*ADRB2*, P07550	*ADRB3*, P13945
Rank order of potency	(‐)‐noradrenaline>(‐)‐adrenaline	(‐)‐adrenaline>(‐)‐noradrenaline	(‐)‐noradrenaline = (‐)‐adrenaline
Endogenous agonists	noradrenaline (p*K*_i_ 6) [549]	–	–
Agonists	pindolol (Partial agonist) (p*K*_i_ 9.3) [1011], isoprenaline (p*K*_i_ 6.6–7) [549, 1651], dobutamine (Partial agonist) (p*K*_i_ 5.5) [831]	pindolol (Partial agonist) (p*K*_i_ 9.4) [1011], arformoterol (p*K*_i_ 8.6) [37], isoprenaline (p*K*_i_ 6.4) [1651], dobutamine (Partial agonist) (p*K*_i_ 6.2) [1100], ephedrine (Partial agonist) (p*K*_i_ 5.6) [851]	carazolol (p*K*_i_ 8.7) [1256]
Selective agonists	(‐)‐Ro 363 (p*K*_i_ 8) [1301], xamoterol (Partial agonist) (p*K*_i_ 7) [831], denopamine (Partial agonist) (p*K*_i_ 5.8) [831, 1830]	formoterol (pEC_50_ 10.1) [81], salmeterol (pEC_50_ 9.9) [81], zinterol (pEC_50_ 9.5) [81], vilanterol (pEC_50_ 9.4) [1534], procaterol (pEC_50_ 8.4) [81], indacaterol (p*K*_i_ 7.8) [107], fenoterol (p*K*_i_ 6.9) [55], salbutamol (Partial agonist) (p*K*_i_ 5.8–6.1) [83, 831], terbutaline (Partial agonist) (p*K*_i_ 5.6) [83], orciprenaline (p*K*_d_ 5.3) [1784]	L 755507 (pEC_50_ 10.1) [81], L742791 (pEC_50_ 8.8) [2000], mirabegron (pEC_50_ 7.7) [1849], CGP 12177 (Partial agonist) (p*K*_i_ 6.1–7.3) [159, 1144, 1256, 1301], SB251023 (pEC_50_ 7.1) [810] – Mouse, BRL 37344 (p*K*_i_ 6.4–7) [159, 431, 768, 1256], CL316243 (p*K*_i_ 5.2) [2080]
Antagonists	carvedilol (p*K*_i_ 9.5) [262], bupranolol (p*K*_i_ 7.3–9) [262, 1144], levobunolol (p*K*_i_ 8.4) [68], labetalol (p*K*_i_ 8.2) [68], metoprolol (p*K*_i_ 7–7.6) [83, 262, 768, 1144], esmolol (p*K*_i_ 6.9) [68], nadolol (p*K*_i_ 6.9) [262], practolol (p*K*_i_ 6.1–6.8) [83, 1144], propafenone (p*K*_i_ 6.7) [68], sotalol (p*K*_i_ 6.1) [68]	carvedilol (p*K*_i_ 9.4–9.9) [83, 262], timolol (p*K*_i_ 9.7) [83], propranolol (p*K*_i_ 9.1–9.5) [83, 86, 831, 1144], levobunolol (p*K*_i_ 9.3) [68], bupranolol (p*K*_i_ 8.3–9.1) [262, 1144], alprenolol (p*K*_i_ 9) [83], nadolol (p*K*_i_ 7–8.6) [83, 262], labetalol (p*K*_i_ 8) [68], propafenone (p*K*_i_ 7.4) [68], sotalol (p*K*_i_ 6.5) [68]	carvedilol (p*K*_i_ 9.4) [262], bupranolol (p*K*_i_ 6.8–7.3) [159, 262, 1144, 1256], propranolol (p*K*_i_ 6.3–7.2) [1144, 1517], levobunolol (p*K*_i_ 6.8) [1517]
Selective antagonists	CGP 20712A (p*K*_i_ 8.5–9.2) [83, 262, 1144], levobetaxolol (p*K*_i_ 9.1) [1715], betaxolol (p*K*_i_ 8.8) [1144], nebivolol (pIC_50_ 8.1–8.7) [1476] – Rabbit, atenolol (p*K*_i_ 6.7–7.6) [83, 886, 1144], acebutolol (p*K*_i_ 6.4) [68]	ICI 118551 (Inverse agonist) (p*K*_i_ 9.2–9.5) [83, 86, 1144]	L‐748337 (p*K*_i_ 8.4) [262], SR59230A (p*K*_i_ 6.9–8.4) [262, 405, 768], L748328 (p*K*_i_ 8.4) [262]
Labelled ligands	[^125^I]ICYP (Selective Antagonist) (p*K*_d_ 10.4–11.3) [831, 1144, 1651]	[^125^I]ICYP (Antagonist) (p*K*_d_ 11.1) [1144, 1651]	[^125^I]ICYP (Agonist, Partial agonist) (p*K*_d_ 9.2–9.8) [1144, 1301, 1517, 1651, 1806]
Comments	The agonists indicated have less than two orders of magnitude selectivity [81].	–	Agonist SB251023 has a pEC50 of 6.9 for the splice variant of the mouse *β*_3_ receptor, *β*_3b_ [810].

### Comments

#### **Adrenoceptors, *α*_1_**

The clone originally called the *α*_1C_‐adrenoceptor corresponds to the pharmacologically defined *α*_1A_‐adrenoceptor [752]. Some tissues possess *α*_1A_‐adrenoceptors (termed *α*_1L_‐adrenoceptors [532, 1329]) that display relatively low affinity in functional and binding assays for prazosin(p*K_i_*< 9) indicative of different receptor states or locations. *α*_1A_‐adrenoceptor C‐terminal splice variants form homo‐ and heterodimers, but fail to generate a functional *α*_1L_‐adrenoceptor [1557]. *α*_1D_‐Adrenoceptors form heterodimers with *α*_1B_‐ or *β*_2_‐adrenoceptors that show increased cell‐surface expression [1917]. Recombinant *α*_1D_‐adrenoceptors have been shown in some heterologous systems to be mainly located intracellularly but cell‐surface localization is attained by truncation of the N‐terminus, or by co‐expression of *α*_1B_‐ or *β*_2_‐adrenoceptors to form heterodimers [670, 1917]. In smooth muscle of native blood vessels all three *α*_1‐_adrenoceptor subtypes are located on the surface and intracellularly [1260, 1261].

Signalling is predominantly via G_q/11_ but *α*_1_‐adrenoceptors also couple to G_i/o_, G_s_ and G_12/13_. Several ligands activating *α*_1A_‐adrenoceptors display ligand directed signalling bias relative to noradrenaline. For example, oxymetazoline is a full agonist for extracellular acidification rate (ECAR) and a partial agonist for Ca^2+^ release but does not stimulate cAMP production. Phenylephrine is biased toward ECAR versus Ca^2+^ release or cAMP accumulation but not between Ca^2+^ release and cAMP accumulation [495]. There are also differences between subtypes in coupling efficiency to different pathways‐ *e.g.* in some systems coupling efficiency to Ca^2+^ signalling is *α*_1A_>*α*_1B_>*α*_1D_, but for MAP kinase signalling is *α*_1D_>*α*_1A_>*α*_1B_. In vascular smooth muscle, the potency of agonists is related to the predominant subtype, *α*_1D_‐ conveying greater agonist sensitivity than *α*_1A_‐adrenoceptors [526].

#### **Adrenoceptors, *α*_2_**

ARC‐239(pK_i_ 8.0) and prazosin(pK_i_ 7.5) show selectivity for *α*_2B_‐ and *α*_2C_‐adrenoceptors over *α*_2A_‐adrenoceptors.Oxymetazoline is a reduced efficacy agonist and is one of many *α*_2_‐adrenoceptor agonists that are imidazolines or closely related compounds. Other binding sites for imidazolines, distinct from *α*_2_‐adrenoceptors, and structurally distinct from the 7TM adrenoceptors, have been identified and classified as I_1_, I_2_ and I_3_sites [390]; catecholamines have a low affinity, while rilmenidine and moxonidine are selective ligands for these sites, evoking hypotensive effects *in vivo*. I_1_‐imidazoline receptors are involved in central inhibition of sympathetic tone, I_2_‐imidazoline receptors are an allosteric binding site on monoamine oxidase B, and I_3_‐imidazoline receptors regulate insulin secretion from pancreatic *β*‐cells. *α*_2A_‐adrenoceptor stimulation reduces insulin secretion from *β*‐islets [2083], with a polymorphism in the 5'‐UTR of the ADRA2A gene being associated with increased receptor expression in *β*‐islets and heightened susceptibility to diabetes [1599].

*α*_2A_‐ and *α*_2C_‐adrenoceptors form homodimers [1758]. Heterodimers between *α*_2A_‐ and either the *α*_2c_‐adrenoceptor or *μ* opioid peptide receptor exhibit altered signalling and trafficking properties compared to the individual receptors [1758, 1858, 1956]. Signalling by *α*_2_‐adrenoceptors is primarily via G_i/o_, however the *α*_2A_‐adrenoceptor also couples to G_s_[459]. Imidazoline compounds display bias relative to each other at the *α*_2A_‐adrenoceptor when assayed by [^35^S] GTP*γ*S binding compared to inhibition of cAMP accumulation [1477]. The noradrenaline reuptake inhibitor desipramine acts directly on the *α*_2A_‐adrenoceptor, promoting internalisation *via* recruitment of arrestin without activating G proteins [371].

#### **Adrenoceptors, *β***

Radioligand binding with [^125^I]ICYP can be used to define *β*_1_‐ or *β*_2_‐adrenoceptors when conducted in the presence of a 'saturating' concentration of either a *β*_1_‐ or *β*_2_‐adrenoceptor‐selective antagonist. [^3^H]CGP12177 or [^3^H]dihydroalprenolol can be used in place of [^125^I]ICYP. Binding of a fluorescent analogue of CGP 12177 to *β*_2_‐adrenoceptors in living cells has been described [84]. [^125^I]ICYP at higher (nM) concentrations can be used to label *β*_3_‐adrenoceptors in systems where there are few if any other *β*‐adrenoceptor subtypes. Pharmacological differences exist between human and mouse *β*_3_‐adrenoceptors, and the 'rodent selective' agonists BRL 37344 and CL316243 are partial agonists at the human *β*_3_‐adrenoceptor whereas CGP 12177 and L 755507 activate human *β*3‐adrenoceptors with greater potency [1650]. The *β*_3_‐adrenoceptor has an intron in the coding region, but splice variants have only been described for the mouse [496], where the isoforms display different signalling characteristics [810]. There are 3 *β*‐adrenoceptors in turkey (termed the t*β*, t*β*3c and t*β*4c) that have a pharmacology that differs from the human *β*‐adrenoceptors [82]. Numerous polymorphisms have been described for the three *β*‐adrenoceptors; some are associated with alterations in agonist‐evoked signalling and trafficking, altered susceptibility to disease and/or altered responses to pharmacotherapy [1103].

All *β*‐adrenoceptors couple to G_s_(activating adenylyl cyclase and elevating cAMP levels), but it is also clear that they activate other G proteins such as G_i_ and many other G protein‐independent signalling pathways, including arrestin‐mediated signalling, which may in turn lead to activation of MAP kinases. Many antagonists at *β*_1_‐ and *β*_2_‐adrenoceptors are agonists at *β*_3_‐adrenoceptors (CL316243, CGP 12177 and carazolol). Many ‘antagonists’ that block agonist‐stimulated cAMP accumulation, for example carvedilol and bucindolol, are able to activate MAP kinase pathways [85, 497, 559, 560, 1649, 1650] and thus display 'protean agonism'. Bupranolol appears to act as a neutral antagonist in most systems so far examined. Agonists also display biased signalling at the *β*_2_‐adrenoceptor via G_s_ or arrestins [443].

The X‐ray crystal structures have been described of the agonist bound [1988] and antagonist bound forms of the *β*_1_‐ [1989], agonist‐bound [313] and antagonist‐bound forms of the *β*_2_‐adrenoceptor [1561, 1598], as well as a fully active agonist‐bound, G_s_ protein‐coupled *β*_2_‐adrenoceptor [1562]. Carvedilol and bucindolol bind to an extended site of the *β*_1_‐adrenoceptor involving contacts in TM2, 3, and 7 and extracellular loop 2 that may facilitate coupling to arrestins [1989]. Compounds displaying arrestin‐biased signalling at the *β*_2_‐adrenoceptor also have a greater effect on the conformation of TM7, whereas full agonists for G_s_ coupling promote movement of TM5 and TM6 [1127]. Recent studies using NMR spectroscopy have demonstrated significant conformational flexibility in the *β*_2_‐adrenoceptor which is stabilized by both agonist and G proteins highlighting the dynamic nature of interactions with both ligand and downstream signalling partners [946, 1199, 1413]. Such flexibility will likely have consequences for our understanding of biased agonism, and for the future therapeutic exploitation of this phenomenon.

### Further Reading

Baker JG *et al*. (2011) Evolution of *β*‐blockers: from anti‐anginal drugs to ligand‐directed signalling. *Trends Pharmacol. Sci.* **32**: 227‐34 [PMID:21429598]

Bylund DB *et al*. (1994) International Union of Pharmacology nomenclature of adrenoceptors. *Pharmacol. Rev.* **46**: 121‐136 [PMID:7938162]

Cazzola M *et al*. (2011) *β*(2) ‐adrenoceptor agonists: current and future direction. *Br. J. Pharmacol.* **163**: 4‐17 [PMID:21232045]

Daly CJ *et al*. (2011) Previously unsuspected widespread cellular and tissue distribution of *β*‐adrenoceptors and its relevance to drug action. *Trends Pharmacol. Sci.* **32**: 219‐26 [PMID:21429599]

Evans BA *et al*. (2010) Ligand‐directed signalling at beta‐adrenoceptors. *Br. J. Pharmacol.* **159**: 1022‐38 [PMID:20132209]

Gilsbach R *et al*. (2012) Are the pharmacology and physiology of *α*_2 adrenoceptors determined by *α*_2‐heteroreceptors and autoreceptors respectively? *Br. J. Pharmacol.* **165**: 90‐102 [PMID:21658028]

Jensen BC *et al*. (2011) Alpha‐1‐adrenergic receptors: targets for agonist drugs to treat heart failure. *J. Mol. Cell. Cardiol.* **51**: 518‐28 [PMID:21118696]

Kobilka BK. (2011) Structural insights into adrenergic receptor function and pharmacology. *Trends Pharmacol. Sci.* **32**: 213‐8 [PMID:21414670]

Langer SZ. (2015) *α*2‐Adrenoceptors in the treatment of major neuropsychiatric disorders. *Trends Pharmacol. Sci.* **36**: 196‐202 [PMID:25771972]

McGrath JC. (2015) Localization of *α*‐adrenoceptors: JR Vane Medal Lecture. *Br. J. Pharmacol.* **172**: 1179‐94 [PMID:25377869]

Michel MC *et al*. (2011) Are there functional *β*_3‐adrenoceptors in the human heart? *Br. J. Pharmacol.* **162**: 817‐22 [PMID:20735409]

Michel MC *et al*. (2011) *β*‐adrenoceptor agonist effects in experimental models of bladder dysfunction. *Pharmacol. Ther.* **131**: 40‐9 [PMID:21510978]

Michel MC *et al*. (2015) Selectivity of pharmacological tools: implications for use in cell physiology. A review in the theme: Cell signaling: proteins, pathways and mechanisms. *Am. J. Physiol., Cell Physiol.* **308**: C505‐20 [PMID:25631871]

Nishimune A *et al*. (2012) Phenotype pharmacology of lower urinary tract *α*(1)‐adrenoceptors. *Br. J. Pharmacol.* **165**: 1226‐34 [PMID:21745191]

Vasudevan NT *et al*. (2011) Regulation of *β*‐adrenergic receptor function: an emphasis on receptor resensitization. *Cell Cycle* **10**: 3684‐91 [PMID:22041711]

Walker JK *et al*. (2011) New perspectives regarding *β*(2) ‐adrenoceptor ligands in the treatment of asthma. *Br. J. Pharmacol.* **163**: 18‐28 [PMID:21175591] Angiotensin receptors

## Angiotensin receptors

### Overview

The actions of angiotensin II (*AGT*, P01019) (Ang II) are mediated by AT_1_ and AT_2_ receptors (**nomenclature as agreed by the NC‐IUPHAR Subcommittee on Angiotensin Receptors [** **2137** **]**), which have around 30% sequence similarity. Endogenous ligands are angiotensin II(*AGT*, P01019) and angiotensin III(*AGT*, P01019) (Ang III), while angiotensin I(*AGT*, P01019) is weakly active in some systems.

**Table:** 

Nomenclature	AT_1_ receptor	AT_2_ receptor
HGNC, UniProt	*AGTR1*, P30556	*AGTR2*, P50052
Selective agonists	L‐162,313 (pIC_50_ 7.8–7.9) [1490]	CGP42112 (pIC_50_ 9.6) [190], [p‐aminoPhe6]ang II (p*K*_d_ 9.1–9.4) [1789, 2139] – Rat
Antagonists	telmisartan (pIC_50_ 8.4) [1241], olmesartan (pIC_50_ 8.1) [981]	–
Selective antagonists	candesartan (pIC_50_ 9.5–9.7) [1942], EXP3174 (pIC_50_ 7.4–9.5) [1887, 1942], eprosartan (pIC_50_ 8.4–8.8) [464], irbesartan (pIC_50_ 8.7–8.8) [1942], losartan (pIC_50_ 7.4–8.7) [1887, 2139], valsartan (pIC_50_ 8.6) [2138], azilsartan (pIC_50_ 8.1–8.1) [1551, 1844]	PD123177 (pIC_50_ 8.5–9.5) [291, 321, 450] – Rat, EMA401 (pIC_50_ 8.5–9.3) [518, 1582, 1767], PD123319 (p*K*_d_ 8.7–9.2) [449, 2025, 2139]
Labelled ligands	[^3^H]A81988 (Antagonist) (p*K*_d_ 9.2) [690] – Rat, [^3^H]L158809 (Antagonist) (p*K*_d_ 9.2) [305] – Rat, [^3^H]eprosartan (Antagonist) (p*K*_d_ 9.1) [21] – Rat, [^3^H]valsartan (Antagonist) (pIC_50_ 8.8–9) [1954], [^125^I]EXP985 (Antagonist) (p*K*_d_ 8.8) [322] – Rat, [^3^H]losartan (Antagonist) (p*K*_d_ 8.2) [294] – Rat	[^125^I]CGP42112 (Agonist) (p*K*_d_ 10.6) [2017, 2018, 2139]

### Comments

AT_1_ receptors are predominantly coupled to Gq_/11_, however they are also linked to arrestin recruitment and stimulate G protein‐independent arrestin signalling [1156]. Most species express a single *AGTR1* gene, but two related *agtr1a* and a*gtr1b* receptor genes are expressed in rodents. The AT_2_ receptor counteracts several of the growth responses initiated by the AT_1_ receptors. The AT_2_ receptor is much less abundant than the AT_1_ receptor in adult tissues and is upregulated in pathological conditions. AT_1_ receptor antagonists bearing substituted 4‐phenylquinoline moieties have been synthesized, which bind to AT_1_ receptors with nanomolar affinity and are slightly more potent than losartan in functional studies [264]. The antagonist activity of CGP42112 at the AT_2_ receptor has also been reported [2147].

The AT_1_ and bradykinin B2 receptors have been proposed to form a heterodimeric complex [3].

There is also evidence for an AT_4_ receptor that specifically binds angiotensin IV (*AGT*, P01019) and is located in the brain and kidney. An additional putative endogenous ligand for the AT_4_ receptor has been described (LVV‐hemorphin(*HBB*, P68871), a globin decapeptide) [1296].

### Further Reading

Ellis B *et al*. (2012) Evidence for a functional intracellular angiotensin system in the proximal tubule of the kidney. *Am. J. Physiol. Regul. Integr. Comp. Physiol.* **302**: R494‐509 [PMID:22170616]

Karnik SS *et al*. (2015) International Union of Pharmacology. LXXXIX. Angiotensin Receptors: Interpreters of pathophysiological angiotensinergic stimuli. *Pharmacological Reviews*

MacKenzie A. (2011) Endothelium‐derived vasoactive agents, AT1 receptors and inflammation. *Pharmacol. Ther.* **131**: 187‐203 [PMID:21115037]

Patel BM *et al*. (2012) Aldosterone and angiotensin: Role in diabetes and cardiovascular diseases. *Eur. J. Pharmacol.* **697**: 1‐12 [PMID:23041273]

Putnam K *et al*. (2012) The renin‐angiotensin system: a target of and contributor to dyslipidemias, altered glucose homeostasis, and hypertension of the metabolic syndrome. *Am. J. Physiol. Heart Circ. Physiol.* **302**: H1219‐30 [PMID:22227126]

Sevá Pessôa B *et al*. (2013) Key developments in renin‐angiotensin‐aldosterone system inhibition. *Nat Rev Nephrol* **9**: 26‐36 [PMID:23165302]

Zhang H *et al*. (2015) Structure of the Angiotensin receptor revealed by serial femtosecond crystallography. *Cell* **161**: 833‐44 [PMID:25913193]

de Gasparo M *et al*. (2000) International Union of Pharmacology. XXIII. The angiotensin II receptors. *Pharmacol. Rev.* **52**: 415‐472 [PMID:10977869]

## Apelin receptor

### Overview

The apelin receptor (**nomenclature as agreed by the NC‐IUPHAR Subcommittee on the apelin receptor [** **1510** **]**) responds to apelin, a 36 amino‐acid peptide derived initially from bovine stomach. Apelin‐36(*APLN*, Q9ULZ1), apelin‐13(*APLN*, Q9ULZ1) and [Pyr^1^]apelin‐13(*APLN*, Q9ULZ1) are the predominant endogenous ligands which are cleaved from a 77 amino‐acid precursor peptide (*APLN*, Q9ULZ1) by a so far unidentified enzymatic pathway [1864]. A second family of peptides discovered independently and named Elabela [323] or Toddler, that has little sequence similarity to apelin, has been proposed as a second endogenous apelin receptor ligand [1475].

**Table:** 

Nomenclature	apelin receptor
HGNC, UniProt	*APLNR*, P35414
Rank order of potency	[Pyr^1^]apelin‐13 (*APLN*, Q9ULZ1) ≥apelin‐13 (*APLN*, Q9ULZ1) >apelin‐36 (*APLN*, Q9ULZ1) [503, 1864]
Endogenous agonists	apelin‐13 (*APLN*, Q9ULZ1) (Selective) (pIC_50_ 8.8–9.5) [503, 785, 1254], apelin receptor early endogenous ligand (*APELA*, P0DMC3) (Selective) (p*K*_d_ 9.3) [412], apelin‐17 (*APLN*, Q9ULZ1) (Selective) (pIC_50_ 7.9–9) [468, 1254], [Pyr^1^]apelin‐13 (*APLN*, Q9ULZ1) (Selective) (pIC_50_ 7–8.8) [918, 1254], Elabela/Toddler‐21 (*APELA*, P0DMC3) (pIC_50_ 8.7) [2086], Elabela/Toddler‐32 (*APELA*, P0DMC3) (pIC_50_ 8.7) [2086], apelin‐36 (*APLN*, Q9ULZ1) (Selective) (pIC_50_ 8.2–8.6) [503, 785, 918, 1254], Elabela/Toddler‐11 (*APELA*, P0DMC3) (pIC_50_ 7.2) [2086]
Selective agonists	MM07 (Biased agonist) (pEC_50_ 9.5) [203]
Antagonists	MM54 (p*K*_i_ 8.2) [1166]
Labelled ligands	[^125^I][Nle^75^,Tyr^77^]apelin‐36 (human) (Agonist) (p*K*_d_ 11.2) [918], [^125^I][Glp^65^Nle^75^,Tyr^77^]apelin‐13 (Agonist) (p*K*_d_ 10.7) [785], [^125^I](Pyr^1^)apelin‐13 (Agonist) (p*K*_d_ 9.5) [911], [^125^I]apelin‐13 (Agonist) (p*K*_d_ 9.2) [503], [^3^H](Pyr^1^)[Met(0)11]‐apelin‐13 (Agonist) (p*K*_d_ 8.6) [1254]

### Comments

Potency order determined for heterologously expressed human apelin receptor (p*D*_2_ values range from 9.5 to 8.6). The apelin receptor may also act as a co‐receptor with CD4 for isolates of human immunodeficiency virus, with apelin blocking this function [279]. A modified apelin‐13 peptide, apelin‐13(F13A) was reported to block the hypotensive response to apelin in rat *in vivo*[1067], however, this peptide exhibits agonist activity in HEK293 cells stably expressing the recombinant apelin receptor [503].

### Further Reading

Chandrasekaran B *et al*. (2008) The role of apelin in cardiovascular function and heart failure. *Eur. J. Heart Fail.* **10**: 725‐32 [PMID:18583184]

Cheng B *et al*. (2012) Neuroprotection of apelin and its signaling pathway. *Peptides* **37**: 171‐3 [PMID:22820556]

Davenport AP *et al*. (2007) Apelins. *In Encyclopedic Reference of Molecular Pharmacology* Edited by Offermanns S, Rosenthal W: Springer: 201‐206

Japp AG *et al*. (2008) The apelin‐APJ system in heart failure: pathophysiologic relevance and therapeutic potential. *Biochem. Pharmacol.* **75**: 1882‐92 [PMID:18272138]

Langelaan DN *et al*. (2009) Structural insight into G‐protein coupled receptor binding by apelin. *Biochemistry* **48**: 537‐48 [PMID:19123778]

O'Carroll AM *et al*. (2013) The apelin receptor APJ: journey from an orphan to a multifaceted regulator of homeostasis. *J. Endocrinol.* **219**: R13‐35 [PMID:23943882]

Pitkin SL *et al*. (2010) International Union of Basic and Clinical Pharmacology. LXXIV. Apelin receptor nomenclature, distribution, pharmacology, and function. *Pharmacol. Rev.* **62**: 331‐42 [PMID:20605969]

Yang P *et al*. (2015) Apelin, Elabela/Toddler, and biased agonists as novel therapeutic agents in the cardiovascular system. *Trends Pharmacol. Sci.* [PMID:26143239]

## Bile acid receptor

### Overview

The bile acid receptor (GPBA) responds to bile acids produced during the liver metabolism of cholesterol. Selective agonists are promising drugs for the treatment of metabolic disorders, such as type II diabetes, obesity and atherosclerosis.

**Table:** 

Nomenclature	GPBA receptor
HGNC, UniProt	*GPBAR1*, Q8TDU6
Rank order of potency	lithocholic acid>deoxycholic acid>chenodeoxycholic acid, cholic acid (Unknown) [917, 1214]
Selective agonists	betulinic acid (pEC_50_ 6) [590], oleanolic acid (pEC_50_ 5.7) [1648]

### Comments

The triterpenoid natural product betulinic acid has also been reported to inhibit inflammatory signalling through the NF*κ*B pathway [1842]. Disruption of GPBA expression is reported to protect from cholesterol gallstone formation [1951]. A new series of 5‐phenoxy‐1,3‐dimethyl‐1H‐pyrazole‐4‐carboxamides have been reported as highly potent agonists [1138].

### Further Reading

Duboc H *et al*. (2014) The bile acid TGR5 membrane receptor: from basic research to clinical application. *Dig Liver Dis* **46**: 302‐12 [PMID:24411485]

Fiorucci S *et al*. (2010) Bile acid‐activated receptors in the treatment of dyslipidemia and related disorders. *Prog. Lipid Res.* **49**: 171‐85 [PMID:19932133]

Fiorucci S *et al*. (2009) Bile‐acid‐activated receptors: targeting TGR5 and farnesoid‐X‐receptor in lipid and glucose disorders. *Trends Pharmacol. Sci.* **30**: 570‐80 [PMID:19758712]

Keitel V *et al*. (2012) Perspective: TGR5 (Gpbar‐1) in liver physiology and disease. *Clin Res Hepatol Gastroenterol* **36**: 412‐9 [PMID:22521118]

Lefebvre P *et al*. (2009) Role of bile acids and bile acid receptors in metabolic regulation. *Physiol. Rev.* **89**: 147‐91 [PMID:19126757]

Lieu T *et al*. (2014) GPBA: a GPCR for bile acids and an emerging therapeutic target for disorders of digestion and sensation. *Br. J. Pharmacol.* **171**: 1156‐66 [PMID:24111923]

Pols TW *et al*. (2011) The bile acid membrane receptor TGR5 as an emerging target in metabolism and inflammation. *J. Hepatol.* **54**: 1263‐72 [PMID:21145931]

Tiwari A *et al*. (2009) TGR5: an emerging bile acid G‐protein‐coupled receptor target for the potential treatment of metabolic disorders. *Drug Discov. Today* **14**: 523‐30 [PMID:19429513]

## Bombesin receptors

### Overview

Bombesin receptors (**nomenclature recommended by the NC‐IUPHAR Subcommittee on bombesin receptors, [** **857** **]**) are activated by the endogenous ligands gastrin‐releasing peptide(*GRP*, P07492) (GRP), neuromedin B(*NMB*, P08949) (NMB) and GRP‐(18‐27)(*GRP*, P07492) (previously named neuromedin C). Bombesin is a tetradecapeptide, originally derived from amphibians, and is an agonist at BB_1_ and BB_2_ receptors. These receptors couple primarily to the G_q/11_ family of G proteins (but see also [857]). Each of these receptors is widely distributed in the CNS and peripheral tissues [625, 857, 1556, 1642]. Activation of BB_1_ and BB_2_ receptors causes a wide range of physiological actions, including the stimulation of normal and neoplastic tissue growth, smooth‐muscle contraction, appetite and feeding behavior, secretion and many central nervous system effects [857, 858, 859, 1185, 1317, 1556]. A physiological role for the BB_3_ receptor has yet to be fully defined although recently studies using receptor knockout mice and newly described agonists/antagonists suggest an important role in glucose and insulin regulation, metabolic homeostasis, feeding and other CNS behaviors and growth of normal/neoplastic tissues [625, 1186, 1430].

**Table:** 

Nomenclature	BB_1_ receptor	BB_2_ receptor	BB_3_ receptor
HGNC, UniProt	*NMBR*, P28336	*GRPR*, P30550	*BRS3*, P32247
Endogenous agonists	neuromedin B (*NMB*, P08949) (Selective) (p*K*_i_ 8.1–10.3) [857, 1556, 1919]	neuromedin C (pIC_50_ 9.9) [1919], gastrin releasing peptide(14‐27) (human) (Selective) (pIC_50_ 9.7–9.8) [1919]	–
Selective agonists	–	–	compound 8a [PMID: 24900283] (pIC_50_ 8.9) [1129], compound 9g [PMID: 24412111] (pEC_50_ 8.8) [1220], MK‐7725 (pIC_50_ 8.5) [324], MK‐5046 (p*K*_i_ 7.7–8.4) [1321, 1689], [D‐Tyr^6^,Apa‐4Cl^11^,Phe^13^,Nle^14^]bombesin‐(6‐14) (p*K*_i_ 8.1) [1202], compound 17c [PMID: 25497965] (pEC_50_ 7.9) [1219], compound 9f [PMID: 24412111] (pEC_50_ 7.8) [1220], bag‐1 (pIC_50_ 7.7) [659], compound 22e [PMID: 20167483] (pIC_50_ 7.6) [727], bag‐2 (pIC_50_ 7) [659]
Antagonists	D‐Nal‐Cys‐Tyr‐D‐Trp‐Lys‐Val‐Cys‐Nal‐NH_2_ (pIC_50_ 6.2–6.6) [624]	–	–
Selective antagonists	PD 176252 (pIC_50_ 9.3–9.8) [624], PD 168368 (pIC_50_ 9.3–9.6) [624], dNal‐*cyc*(Cys‐Tyr‐dTrp‐Orn‐Val)‐Nal‐NH_2_	[D‐Phe^6^, Leu^13^, Cpa^14^,*ψ*13‐14]bombesin‐(6‐14) (p*K*_i_ 9.8) [624], JMV641 (pIC_50_ 9.3) [1892] – Mouse, [(3‐Ph‐Pr^6^), His^7^,D‐Ala^11^,D‐Pro^13^,*ψ*13‐14),Phe^14^] bombesin‐(6‐14) (pIC_50_ 9.2) [624, 1062], [D‐Tpi^6^, Leu^13^*ψ*(CH_2_NH)‐Leu^14^]bombesin‐(6‐14) (pIC_50_ 8.9) [624], Ac‐GRP‐(20‐26)‐methylester (pIC_50_ 8.7) [624], JMV594 (pIC_50_ 8.7–8.7) [1133, 1892] – Mouse	bantag‐1 (pIC_50_ 8.6–8.7) [659, 1321], ML‐18 (pIC_50_ 5.3) [1316]
Labelled ligands	[^125^I]BH‐NMB (human, mouse, rat) (Agonist), [^125^I][Tyr^4^]bombesin (Agonist)	[^125^I][D‐Tyr^6^]bombesin‐(6‐13)‐methyl ester (Selective Antagonist) (p*K*_d_ 9.3) [1201] – Mouse, [^125^I][Tyr^4^]bombesin (Agonist) (p*K*_d_ 8.2) [131], [^125^I]GRP (human) (Agonist)	[^3^H]bag‐2 (Agonist) (p*K*_d_ 8.6) [659] – Mouse, [^125^I][D‐Tyr^6^,*β*‐Ala^11^,Phe^13^,Nle^14^]bombesin‐(6‐14) (Agonist) (p*K*_d_ 8–8.4) [1203, 1321]

### Comments

All three subtypes may be activated by [D‐Phe^6^,*β*‐Ala^11^,Phe^13^,Nle^14^]bombesin‐(6‐14)[1203]. [D‐Tyr^6^,Apa‐4Cl^11^,Phe^13^,Nle^14^]bombesin‐(6‐14) has more than 200‐fold selectivity for BB_3_ receptors over BB_1_ and BB_2_[1202].

### Further Reading

Gonzalez N *et al*. (2008) Bombesin‐related peptides and their receptors: recent advances in their role in physiology and disease states. *Curr Opin Endocrinol Diabetes Obes* **15**: 58‐64 [PMID:18185064]

González N *et al*. (2015) Bombesin receptor subtype 3 as a potential target for obesity and diabetes. *Expert Opin. Ther. Targets* 1‐18 [PMID:26066663]

Jensen RT *et al*. (2008) International Union of Pharmacology. LXVIII. Mammalian bombesin receptors: nomenclature, distribution, pharmacology, signaling, and functions in normal and disease states. *Pharmacol. Rev.* **60**: 1‐42 [PMID:18055507]

Jensen RT *et al*. (2013) Bombesin‐Related Peptides. *In Handbook of Biologically Active Peptides. 2nd Revised edition.* Edited by Kastin AJ: Elsevier: 1188‐1196 [ISBN: 9780123850959]

Jensen RT *et al*. (2013) Bombesin Peptides (Cancer). *In Handbook of Biologically Active Peptides. 2nd Revised edition.* Edited by Kastin AJ: Elsevier: 506‐511 [ISBN: 9780123850959]

Ladenheim EE.. (2013) Bombesin. *In Handbook of Biologically Active Peptides. 2nd Revised edition.* Edited by Kastin AJ: Elsevier: 1064‐1070 [ISBN: 9780123850959]

Majumdar ID *et al*. (2011) Biology of mammalian bombesin‐like peptides and their receptors. *Curr Opin Endocrinol Diabetes Obes* **18**: 68‐74 [PMID:21042212]

Moody TW *et al*. (2015) Neuropeptides as lung cancer growth factors. *Peptides* [PMID:25836991]

Ramos‐Álvarez I *et al*. (2015) Insights into bombesin receptors and ligands: Highlighting recent advances. *Peptides* [PMID:25976083]

Roesler R *et al*. (2012) Gastrin‐releasing peptide receptors in the central nervous system: role in brain function and as a drug target. *Front Endocrinol (Lausanne)* **3**: 159 [PMID:23251133]

Sun YG *et al*. (2009) Cellular basis of itch sensation. *Science* **325**: 1531‐4 [PMID:19661382]

## Bradykinin receptors

### Overview

Bradykinin (or kinin) receptors (**nomenclature as agreed by the NC‐IUPHAR subcommittee on Bradykinin (kinin) Receptors [** **1072** **]**) are activated by the endogenous peptides bradykinin(*KNG1*, P01042) (BK), [des‐Arg^9^]bradykinin(*KNG1*, P01042), Lys‐BK (kallidin(*KNG1*, P01042)), [des‐Arg^10^]kallidin(*KNG1*, P01042), T‐kinin(*KNG1*, P01042) (Ile‐Ser‐BK), [Hyp^3^]bradykinin(*KNG1*, P01042) and Lys‐[Hyp^3^]‐bradykinin(*KNG1*, P01042). The variation in affinity or inactivity of B_2_ receptor antagonists could reflect the existence of species homologues of B_2_ receptors.

**Table:** 

Nomenclature	B_1_ receptor	B_2_ receptor
HGNC, UniProt	*BDKRB1*, P46663	*BDKRB2*, P30411
Rank order of potency	[des‐Arg^10^]kallidin (*KNG1*, P01042) >[des‐Arg^9^]bradykinin (*KNG1*, P01042) = kallidin (*KNG1*, P01042) >bradykinin (*KNG1*, P01042)	kallidin (*KNG1*, P01042) >bradykinin (*KNG1*, P01042) ≫[des‐Arg^9^]bradykinin (*KNG1*, P01042), [des‐Arg^10^]kallidin (*KNG1*, P01042)
Endogenous agonists	[des‐Arg^10^]kallidin (*KNG1*, P01042) (Selective) (p*K*_i_ 9.6–10) [69, 104, 876]	–
Selective agonists	[Sar,D‐Phe^8^,des‐Arg^9^]bradykinin (p*K*_i_ 5.7) [876]	[Hyp^3^,Tyr(Me)^8^]BK, [Phe^8^,*ψ*(CH_2_‐NH)Arg^9^]BK
Antagonists	[Leu^9^,des‐Arg^10^]kallidin (p*K*_i_ 9.1–9.3) [69, 104]	–
Selective antagonists	B‐9958 (p*K*_i_ 9.2–10.3) [596, 1570], R‐914 (pA_2_ 8.6) [617], R‐715 (pA_2_ 8.5) [618]	icatibant (p*K*_i_ 10.2) [39], FR173657 (pA_2_ 8.2) [1593], anatibant (p*K*_i_ 8.2) [1537]
Labelled ligands	[^125^I]Hpp‐desArg^10^HOE140 (p*K*_d_ 10), [^3^H]Lys‐[des‐Arg^9^]BK (Agonist) (p*K*_d_ 9.4), [^3^H]Lys‐[Leu^8^][des‐Arg^9^]BK (Antagonist)	[^3^H]BK (human, mouse, rat) (Agonist) (p*K*_d_ 9.4) [2034] – Mouse, [^3^H]NPC17731 (Antagonist) (p*K*_d_ 9.1–9.4) [2119, 2120], [^125^I][Tyr^8^]bradykinin (Agonist)

### Further Reading

Campos MM *et al*. (2006) Non‐peptide antagonists for kinin B1 receptors: new insights into their therapeutic potential for the management of inflammation and pain. *Trends Pharmacol. Sci.* **27**: 646‐51 [PMID:17056130]

Duchene J *et al*. (2009) The kinin B(1) receptor and inflammation: new therapeutic target for cardiovascular disease. *Curr Opin Pharmacol* **9**: 125‐31 [PMID:19124274]

Marceau F *et al*. (2004) Bradykinin receptor ligands: therapeutic perspectives. *Nat Rev Drug Discov* **3**: 845‐52 [PMID:15459675]

Paquet JL *et al*. (1999) Pharmacological characterization of the bradykinin B2 receptor: inter‐species variability and dissociation between binding and functional responses. *Br. J. Pharmacol.* **126**: 1083‐90 [PMID:10204994]

Thornton E *et al*. (2010) Kinin receptor antagonists as potential neuroprotective agents in central nervous system injury. *Molecules* **15**: 6598‐618 [PMID:20877247]

## Calcitonin receptors

### Overview

This receptor family comprises a group of receptors for the calcitonin/CGRP family of peptides. The calcitonin (CT), amylin (AMY), calcitonin gene‐related peptide (CGRP) and adrenomedullin (AM) receptors (**nomenclature as agreed by the NC‐IUPHAR Subcommittee on CGRP, AM, AMY, and CT receptors [** **721** **,** **1528** **]**) are generated by the genes *CALCR* (which codes for the CT receptor (CTR)) and *CALCRL*(which codes for the calcitonin receptor‐like receptor, CLR, previously known as CRLR). Their function and pharmacology are altered in the presence of RAMPs (receptor activity‐modifying proteins), which are single TM domain proteins of *ca.* 130 amino acids, identified as a family of three members; RAMP1, RAMP2 and RAMP3. There are splice variants of CTR; these in turn produce variants of the AMY receptor [1528], some of which can be potently activated by CGRP. The endogenous agonists are the peptides calcitonin(*CALCA*, P01258), *α*‐CGRP(*CALCA*, P06881) (formerly known as CGRP‐I), *β*‐CGRP(*CALCB*, P10092) (formerly known as CGRP‐II), amylin(*IAPP*, P10997) (occasionally called islet‐amyloid polypeptide, diabetes‐associated polypeptide), adrenomedullin(*ADM*, P35318) and adrenomedullin 2/intermedin (*ADM2*, Q7Z4H4). There are species differences in peptide sequences, particularly for the CTs. CTR‐stimulating peptide {Pig} (CRSP) is another member of the family with selectivity for the CTR but it is not expressed in humans [907]. Olcegepant (also known as BIBN4096BS, *p*Ki 10.5) and telcagepant(also known as MK0974, *p*Ki 9) are the most selective antagonists available, having a high selectivity for CGRP receptors, with a particular preference for those of primate origin.

CLR by itself binds no known endogenous ligand, but in the presence of RAMPs it gives receptors for CGRP, adrenomedullin and adrenomedullin 2/intermedin.

**Table:** 

Nomenclature	CT receptor	AMY_1_ receptor	AMY_2_ receptor	AMY_3_ receptor
HGNC, UniProt	*CALCR*, P30988	–	–	–
Subunits	–	RAMP1 (Accessory protein), CT receptor	CT receptor, RAMP2 (Accessory protein)	CT receptor, RAMP3 (Accessory protein)
Rank order of potency	calcitonin (salmon)≥calcitonin (*CALCA*, P01258) ≥amylin (*IAPP*, P10997), *α*‐CGRP (*CALCA*, P06881) >adrenomedullin (*ADM*, P35318), adrenomedullin 2/intermedin (*ADM2*, Q7Z4H4)	calcitonin (salmon)≥amylin (*IAPP*, P10997) ≥*α*‐CGRP (*CALCA*, P06881) >adrenomedullin 2/intermedin (*ADM2*, Q7Z4H4) ≥calcitonin (*CALCA*, P01258) >adrenomedullin (*ADM*, P35318)	Poorly defined	calcitonin (salmon)≥amylin (*IAPP*, P10997) >*α*‐CGRP (*CALCA*, P06881) ≥adrenomedullin 2/intermedin (*ADM2*, Q7Z4H4) ≥calcitonin (*CALCA*, P01258) >adrenomedullin (*ADM*, P35318)
Endogenous agonists	calcitonin (*CALCA*, P01258) (Selective) (pEC_50_ 9–11.2) [32, 58, 718, 1027, 1087, 1341]	amylin (*IAPP*, P10997) (pEC_50_ 9–9.7) [610]	amylin (*IAPP*, P10997) (pEC_50_ 8.3–9.1) [610]	amylin (*IAPP*, P10997) (pEC_50_ 8.9–9.6) [610]
Labelled ligands	[^125^I]CT (human) (Agonist) (p*K*_d_ 9–10), [^125^I]CT (salmon) (Agonist) (p*K*_d_ 10)	[^125^I]BH‐AMY (rat, mouse) (Agonist) (p*K*_d_ 9–10)	[^125^I]BH‐AMY (rat, mouse) (Agonist) (p*K*_d_ 9–10)	[^125^I]BH‐AMY (rat, mouse) (Agonist) (p*K*_d_ 9–10)

**Table:** 

Nomenclature	calcitonin receptor‐like receptor	CGRP receptor	AM_1_ receptor	AM_2_ receptor
HGNC, UniProt	*CALCRL*, Q16602	–	–	–
Subunits	–	calcitonin receptor‐like receptor, RAMP1 (Accessory protein)	calcitonin receptor‐like receptor, RAMP2 (Accessory protein)	calcitonin receptor‐like receptor, RAMP3 (Accessory protein)
Rank order of potency	–	*α*‐CGRP (*CALCA*, P06881) >adrenomedullin (*ADM*, P35318) ≥adrenomedullin 2/intermedin (*ADM2*, Q7Z4H4) >amylin (*IAPP*, P10997) ≥calcitonin (salmon)	adrenomedullin (*ADM*, P35318) >adrenomedullin 2/intermedin (*ADM2*, Q7Z4H4) >*α*‐CGRP (*CALCA*, P06881), amylin (*IAPP*, P10997) >calcitonin (salmon)	adrenomedullin (*ADM*, P35318) ≥adrenomedullin 2/intermedin (*ADM2*, Q7Z4H4) ≥*α*‐CGRP (*CALCA*, P06881) >amylin (*IAPP*, P10997) >calcitonin (salmon)
Endogenous agonists	–	*β*‐CGRP (*CALCB*, P10092) (p*K*_i_ 9.9–11) [20, 1251], *α*‐CGRP (*CALCA*, P06881) (p*K*_i_ 9.7–10) [20, 1251]	adrenomedullin (*ADM*, P35318) (p*K*_i_ 8.3–9.2) [20, 1251]	adrenomedullin (*ADM*, P35318) (p*K*_i_ 8.3–9) [20, 539]
Antagonists	–	olcegepant (p*K*_i_ 10.2–10.7) [435, 719, 720, 1194], telcagepant (p*K*_i_ 9.1) [1633]	–	–
Selective antagonists	–	–	AM‐(22‐52) (human) (p*K*_i_ 7–7.8) [20, 720, 1251]	–
Labelled ligands	–	[^125^I]*α*CGRP (human) (Agonist) (p*K*_d_ 10), [^125^I]*α*CGRP (mouse, rat) (Agonist)	[^125^I]AM (rat) (Agonist) (p*K*_d_ 10–9)	[^125^I]AM (rat) (Agonist) (p*K*_d_ 9–10)

### Comments

It is important to note that a complication with the interpretation of pharmacological studies with AMY receptors in transfected cells is that most of this work has likely used a mixed population of receptors, encompassing RAMP‐coupled CTR as well as CTR alone. This means that although in binding assays human calcitonin(*CALCA*, P01258) has low affinity for ^125^I‐AMY binding sites, cells transfected with CTR and RAMPs can display potent CT functional responses. Transfection of human CTR with any RAMP can generate receptors with a high affinity for both salmon CT and AMY and varying affinity for different antagonists [337, 718, 719]. The major human CTR splice variant (hCT_(a)_, which does not contain an insert) with RAMP1 (*i.e.* the AMY_1(a)_ receptor) has a high affinity for CGRP, unlike hCT_(a)_‐RAMP3 (*i.e*. AMY_3(a)_ receptor) [337, 718]. However, the AMY receptor phenotype is RAMP‐type, splice variant and cell‐line‐dependent [1886]. In particular, CGRP is a more potent agonist than amylin(*IAPP*, P10997) at increasing cAMP at the delta 47 hCT(a) receptor, when transfected with RAMP1 (to give the corresponding AMY1(a) receptor) in Cos 7 cells [1543].

The ligands described represent the best available but their selectivity is limited. For example, adrenomedullin has appreciable affinity for CGRP receptors. CGRP can show significant cross‐reactivity at AMY receptors and AM_2_ receptors. Adrenomedullin 2/intermedin also has high affinity for the AM_2_ receptor [779]. CGRP‐(8‐37) acts as an antagonist of CGRP (*p*K_i_ 8) and inhibits some AM and AMY responses (*p*K_i_ 6‐7). It is weak at CT receptors. Salmon CT‐(8‐32) is an antagonist at both AMY and CT receptors. AC187, a salmon CT analogue, is also an antagonist at AMY and CT receptors. Human AM‐(22‐52) has some selectivity towards AM receptors, but with modest potency (*p*K_i_  7), limiting its use [720]. AM‐(22‐52) is slightly more effective at AM_1_ than AM_2_ receptors but this difference is not sufficient for this peptide to be a useful discriminator of the AM receptor subtypes. Olcegepant shows the greatest selectivity between receptors but still has significant affinity for AMY_1_ receptors [1973].

Ligand responsiveness at CT and AMY receptors can be affected by receptor splice variation and can depend on the pathway being measured. Particularly for AMY receptors, relative potency can vary with the type and level of RAMP present and can be influenced by other factors such as G proteins [1324, 1886].

G_s_ is a prominent route for effector coupling for CLR and CTR but other pathways (*e.g.* Ca^2+^, ERK, Akt), and G proteins can be activated [1972]. There is evidence that CGRP‐RCP (a 148 amino‐acid hydrophilic protein, *ASL*(P04424) is important for the coupling of CLR to adenylyl cyclase [498]. [^125^I]‐Salmon CT is the most common radioligand for CT receptors but it has high affinity for AMY receptors and is also poorly reversible. [^125^I]‐Tyr^0^‐CGRP is widely used as a radioligand for CGRP receptors.

Some early literature distinguished between *CGRP_1_* and *CGRP_2_* receptors. It is now clear that the complex of *CALCRL* and *RAMP1* represents the *CGRP_1_* subtype and is now known simply as the CGRP receptor [721]. The *CGRP_2_* receptor is now considered to have arisen from the actions of CGRP at AM_2_ and AMY receptors. This term should not be used [721].

### Further Reading

Barwell J *et al*. (2012) Calcitonin and calcitonin receptor‐like receptors: common themes with family B GPCRs? *Br. J. Pharmacol.* **166**: 51‐65 [PMID:21649645]

Booe JM *et al*. (2015) Structural Basis for Receptor Activity‐Modifying Protein‐Dependent Selective Peptide Recognition by a G Protein‐Coupled Receptor. *Mol. Cell* [PMID:25982113]

Hay DL *et al*. (2008) International Union of Pharmacology. LXIX. Status of the calcitonin gene‐related peptide subtype 2 receptor. *Pharmacol. Rev.* **60**: 143‐5 [PMID:18552275]

Hong Y *et al*. (2011) The pharmacology of Adrenomedullin 2/Intermedin. *Br J Pharmacol* [PMID:21658025]

Moore EL *et al*. (2011) Targeting a Family B GPCR/RAMP Receptor Complex: CGRP Receptor Antagonists and Migraine. *Br J Pharmacol* [PMID:21871019]

Poyner DR *et al*. (2002) International Union of Pharmacology. XXXII. The mammalian calcitonin gene‐related peptides, adrenomedullin, amylin, and calcitonin receptors. *Pharmacol Rev.* **54**: 233‐246 [PMID:12037140]

Russo AF. (2015) Calcitonin gene‐related peptide (CGRP): a new target for migraine. *Annu. Rev. Pharmacol. Toxicol.* **55**: 533‐52 [PMID:25340934]

## Calcium‐sensing receptors

### Overview

The calcium‐sensing receptor (CaS, **provisional nomenclature as recommended by NC‐IUPHAR[** **530** **]**) responds to extracellular calcium and magnesium in the millimolar range and to gadolinium and some polycations in the micromolar range [229]. The sensitivity of CaS to primary agonists can be increased by aromatic L‐amino acids [362] and also by elevated extracellular pH [1544] or decreased extracellular ionic strength [1545]. This receptor bears no sequence or structural relation to the plant calcium receptor, also called CaS.

**Table:** 

Nomenclature	CaS receptor	GPRC6 receptor
HGNC, UniProt	*CASR*, P41180	*GPRC6A*, Q5T6X5
Amino‐acid rank order of potency	L‐phenylalanine, L‐tryptophan, L‐histidine>L‐alanine>L‐serine, L‐proline, L‐glutamic acid>L‐aspartic acid (not L‐lysine, L‐arginine, L‐leucine and L‐isoleucine) [362]	–
Cation rank order of potency	Gd^3+^>Ca^2+^>Mg^2+^ [229]	–
Polyamine rank order of potency	spermine>spermidine>putrescine [1546]	–
Allosteric modulators	AC265347 (Positive) (pEC_50_ 7.6–8.1) [1160], NPS 2143 (Negative) (pIC_50_ 7.1–7.4) [1377, 2087], cinacalcet (Positive) (pEC_50_ 7.3) [1378], calindol (Positive) (pEC_50_ 6.5) [1499], calindol (Positive) (p*K*_d_ 6–6.5) [930], tecalcet (Positive) (p*K*_d_ 6.5) [1379], calhex 231 (Negative) (pIC_50_ 6.4) [1500]	–
Comments	2‐benzylpyrrolidine derivatives of NPS 2143 are also negative allosteric modulators of the calcium sensing receptor [2087]. etelcalcetide is a novel peptide agonist of the receptor [1975].	GPRC6 is a related G_q_‐coupled receptor which responds to basic amino acids [2004].

### Comments

Positive allosteric modulators of CaS are termed Type II calcimimetics and can suppress parathyroid hormone (PTH(*PTH*, P01270)) secretion [1379]. Negative allosteric modulators are called calcilytics and can act to increase PTH(*PTH*, P01270) secretion [1377].

The central role of CaS in the maintenance of extracellular calcium homeostasis is seen most clearly in patients with loss‐of‐function CaS mutations who develop familial hypocalciuric hypercalcaemia (heterozygous mutation) or neonatal severe hyperparathyroidism (homozygous mutation) and in CaS null mice [293, 765], which exhibit similar increases in PTH secretion and blood Ca^2+^ levels. A gain‐of‐function mutation in the CaS gene is associated with autosomal dominant hypocalcaemia.

### Further Reading

Breitwieser GE. (2012) Minireview: the intimate link between calcium sensing receptor trafficking and signaling: implications for disorders of calcium homeostasis. *Mol. Endocrinol.* **26**: 1482‐95 [PMID:22745192]

Brown EM. (2013) Role of the calcium‐sensing receptor in extracellular calcium homeostasis. *Best Pract. Res. Clin. Endocrinol. Metab.* **27**: 333‐43 [PMID:23856263]

Conigrave AD *et al*. (2013) Calcium‐sensing receptor (CaSR): pharmacological properties and signaling pathways. *Best Pract. Res. Clin. Endocrinol. Metab.* **27**: 315‐31 [PMID:23856262]

Magno AL *et al*. (2011) The calcium‐sensing receptor: a molecular perspective. *Endocr. Rev.* **32**: 3‐30 [PMID:20729338]

Nemeth EF *et al*. (2013) Calcimimetic and calcilytic drugs for treating bone and mineral‐related disorders. *Best Pract. Res. Clin. Endocrinol. Metab.* **27**: 373‐84 [PMID:23856266]

Wellendorph P *et al*. (2004) Molecular cloning, expression, and sequence analysis of GPRC6A, a novel family C G‐protein‐coupled receptor. *Gene* **335**: 37‐46 [PMID:15194188]

Yarova PL *et al*. (2015) Calcium‐sensing receptor antagonists abrogate airway hyperresponsiveness and inflammation in allergic asthma. *Sci Transl Med* **7**: 284ra60 [PMID:25904744]

## Cannabinoid receptors

### Overview

Cannabinoid receptors (**nomenclature as agreed by the NC‐IUPHAR Subcommittee on Cannabinoid Receptors [** **1494** **]**) are activated by endogenous ligands that include N‐arachidonoylethanolamine (anandamide), N‐homo‐*γ*‐linolenoylethanolamine, N‐docosatetra‐7,10,13,16‐enoylethanolamine and 2‐arachidonoylglycerol. Potency determinations of endogenous agonists at these receptors are complicated by the possibility of differential susceptibility of endogenous ligands to enzymatic conversion [35].

**Table:** 

Nomenclature	CB_1_ receptor	CB_2_ receptor
HGNC, UniProt	*CNR1*, P21554	*CNR2*, P34972
(Sub)family‐selective agonists	HU‐210 (p*K*_i_ 9.1–10.2) [509, 1733], CP55940 (p*K*_i_ 8.3–9.2) [509, 1602, 1733], WIN55212‐2 (p*K*_i_ 6.9–8.7) [509, 1730, 1733], *Δ*^9^‐tetrahydrocannabinol (Partial agonist) (p*K*_i_ 7.3–7.4) [509, 1733]	HU‐210 (p*K*_i_ 9.3–9.8) [509, 1579, 1733], WIN55212‐2 (p*K*_i_ 8.4–9.6) [509, 1730, 1733], CP55940 (p*K*_i_ 8.6–9.2) [509, 1602, 1733], *Δ*^9^‐tetrahydrocannabinol (Partial agonist) (p*K*_i_ 7.1–7.5) [106, 509, 1579, 1733]
Selective agonists	arachidonyl‐2‐chloroethylamide (p*K*_i_ 8.9) [755] – Rat, arachidonylcyclopropylamide (p*K*_i_ 8.7) [755] – Rat, O‐1812 (p*K*_i_ 8.5) [420] – Rat, *R*‐(+)‐methanandamide (p*K*_i_ 7.7) [931] – Rat	JWH‐133 (p*K*_i_ 8.5) [804, 1493], L‐759,633 (p*K*_i_ 7.7–8.2) [576, 1602], AM1241 (p*K*_i_ 8.1) [2088], L‐759,656 (p*K*_i_ 7.7–7.9) [576, 1602], HU‐308 (p*K*_i_ 7.6) [699]
Selective antagonists	rimonabant (p*K*_i_ 7.9–8.7) [508, 509, 1586, 1613, 1733], AM251 (p*K*_i_ 8.1) [1038] – Rat, AM281 (p*K*_i_ 7.9) [1037] – Rat, LY320135 (p*K*_i_ 6.9) [508]	SR144528 (p*K*_i_ 8.3–9.2) [1587, 1602], AM‐630 (p*K*_i_ 7.5) [1602]
Labelled ligands	[^3^H]rimonabant (Antagonist) (p*K*_d_ 8.9–10) [205, 761, 889, 1498, 1588, 1742, 1873] – Rat	–

### Comments

Both CB_1_ and CB_2_ receptors may be labelled with [^3^H]CP55940(0.5 nM; [1733]) and [^3^H]WIN55212‐2(2‐2.4 nM; [1756, 1783]). Anandamide is also an agonist at vanilloid receptors (TRPV1) and PPARs[1418, 2135]. There is evidence for an allosteric site on the CB_1_ receptor [1532]. All of the compounds listed as antagonists behave as inverse agonists in some bioassay systems [1494]. Moreover, GPR18, GPR55 and GPR119, although showing little structural similarity to CB_1_ and CB_2_receptors, respond to endogenous agents that are structurally similar to the endogenous cannabinoid ligands [1494].

### Further Reading

Alexander SP *et al*. (2007) The complications of promiscuity: endocannabinoid action and metabolism. *Br. J. Pharmacol.* **152**: 602‐23 [PMID:17876303]

Di Marzo V *et al*. (2007) Endocannabinoids and the regulation of their levels in health and disease. *Curr. Opin. Lipidol.* **18**: 129‐40 [PMID:17353660]

Howlett AC *et al*. (2011) Endocannabinoid tone versus constitutive activity of cannabinoid receptors. *Br. J. Pharmacol.* **163**: 1329‐43 [PMID:21545414]

McPartland JM *et al*. (2007) Meta‐analysis of cannabinoid ligand binding affinity and receptor distribution: interspecies differences. *Br. J. Pharmacol.* **152**: 583‐93 [PMID:17641667]

Mechoulam R *et al*. (2013) The endocannabinoid system and the brain. *Annu Rev Psychol* **64**: 21‐47 [PMID:22804774]

O'Sullivan SE. (2007) Cannabinoids go nuclear: evidence for activation of peroxisome proliferator‐activated receptors. *Br. J. Pharmacol.* **152**: 576‐82 [PMID:17704824]

Pertwee RG *et al*. (2010) International Union of Basic and Clinical Pharmacology. LXXIX. Cannabinoid receptors and their ligands: beyond CB_1 and CB_2. *Pharmacol. Rev.* **62**: 588‐631 [PMID:21079038]

Ross RA. (2011) L‐*α*‐lysophosphatidylinositol meets GPR55: a deadly relationship. *Trends Pharmacol. Sci.* **32**: 265‐9 [PMID:21367464]

## Chemerin receptor

### Overview

The chemerin receptor (**nomenclature as recommended by NC‐IUPHAR[** **396** **]**) is activated by chemerin [1148, 1253, 2108] and the lipid‐derived, anti‐inflammatory ligand resolvin E1 (RvE1), which is the result of sequential metabolism of EPA by aspirin‐modified cyclooxygenase and lipoxygenase [56, 57]. In addition, two GPCRs for resolvin D1 (RvD1) have been identified, FPR2/ALX, the lipoxin A_4_ receptor, and GPR32, an orphan receptor [1006].

**Table:** 

Nomenclature	chemerin receptor
HGNC, UniProt	*CMKLR1*, Q99788
Rank order of potency	resolvin E1>chemerin C‐terminal peptide>18R‐HEPE>EPA [56]
Selective agonists	resolvin E1
Labelled ligands	[^3^H]resolvin E1 (Agonist) (p*K*_d_ 8) [56, 57]

## Chemokine receptors

### Overview

Chemokine receptors (**nomenclature as agreed by the NC‐IUPHAR Subcommittee on Chemokine Receptors [** **78** **,** **1346** **,** **1347** **]**) comprise a large subfamily of 7TM proteins that bind one or more chemokines, a large family of small cytokines typically possessing chemotactic activity for leukocytes. Chemokine receptors can be divided by function into two main groups: G protein‐coupled chemokine receptors, which mediate leukocyte trafficking, and “Atypical chemokine receptors”, which may signal through non‐G protein‐coupled mechanisms and act as chemokine scavengers to downregulate inflammation or shape chemokine gradients [78].

Chemokines in turn can be divided by structure into four subclasses by the number and arrangement of conserved cysteines. CC (also known as *β*‐chemokines; *n*= 28), CXC (also known as *α*‐chemokines; *n*= 17) and CX3C (*n*= 1) chemokines all have four conserved cysteines, with zero, one and three amino acids separating the first two cysteines respectively. C chemokines (*n*= 2) have only the second and fourth cysteines found in other chemokines. Chemokines can also be classified by function into homeostatic and inflammatory subgroups. Most chemokine receptors are able to bind multiple high‐affinity chemokine ligands, but the ligands for a given receptor are almost always restricted to the same structural subclass. Most chemokines bind to more than one receptor subtype. Receptors for inflammatory chemokines are typically highly promiscuous with regard to ligand specificity, and may lack a selective endogenous ligand. G protein‐coupled chemokine receptors are named acccording to the class of chemokines bound, whereas ACKR is the root acronym for atypical chemokine receptors [79]. Listed are those human agonists with EC_50_ values <50 nM in either Ca^2+^ flux or chemotaxis assays at human recombinant G protein‐coupled chemokine receptors expressed in mammalian cell lines. There can be substantial cross‐species differences in the sequences of both chemokines and chemokine receptors, and in the pharmacology and biology of chemokine receptors. Endogenous and microbial non‐chemokine ligands have also been identified for chemokine receptors. Many chemokine receptors function as HIV co‐receptors, but CCR5 is the only one demonstrated to play an essential role in HIV/AIDS pathogenesis. The tables include both standard chemokine receptor names [2101] and the most commonly used aliases. Numerical data quoted are typically p*K*_i_ or pIC_50_ values from radioligand binding to heterologously expressed receptors.

**Table:** 

Nomenclature	CCR1	CCR2	CCR3
HGNC, UniProt	*CCR1*, P32246	*CCR2*, P41597	*CCR3*, P51677
Endogenous agonists	CCL3 (*CCL3*, P10147) (p*K*_i_ 7.8–10.2) [328, 357, 747, 2134], CCL23 (*CCL23*, P55773) (Selective) (p*K*_i_ 8.9) [328], CCL5 (*CCL5*, P13501) (p*K*_i_ 6.8–8.2) [357, 747], CCL7 (*CCL7*, P80098) (p*K*_i_ 8.1) [328, 667], CCL15 (*CCL15*, Q16663) (Selective) (pIC_50_ 7.9) [373], CCL14 (*CCL14*, Q16627) (p*K*_i_ 7.4) [328], CCL13 (*CCL13*, Q99616), CCL8 (*CCL8*, P80075)	CCL2 (*CCL2*, P13500) (pIC_50_ 9.3–10.2) [373, 1159, 1291, 1465, 1920], CCL13 (*CCL13*, Q99616) (pIC_50_ 8.6–8.7) [1159, 1920], CCL7 (*CCL7*, P80098) (pIC_50_ 8.4–8.7) [373, 1159, 1920], CCL11 (*CCL11*, P51671) (Partial agonist) (pIC_50_ 7.1–7.7) [1159, 1465], CCL16 (*CCL16*, O15467)	CCL13 (*CCL13*, Q99616) (pIC_50_ 8.7–10.3) [1332, 1920], CCL24 (*CCL24*, O00175) (Selective) (pIC_50_ 8–9.4) [1332, 1465], CCL5 (*CCL5*, P13501) (p*K*_i_ 8.5–9.3) [391], CCL7 (*CCL7*, P80098) (p*K*_i_ 8.6–9.2) [391], CCL11 (*CCL11*, P51671) (Selective) (pIC_50_ 8.7–9) [452, 961, 1332, 1625, 1920], CCL26 (*CCL26*, Q9Y258) (Selective) (pIC_50_ 7.9–8.9) [961, 1332, 1465], CCL15 (*CCL15*, Q16663) (pIC_50_ 8.6) [373], CCL28 (*CCL28*, Q9NRJ3), CCL8 (*CCL8*, P80075)
Agonists	–	–	CCL11 {Mouse} (p*K*_i_ 9.5–10) [391]
Endogenous antagonists	CCL4 (*CCL4*, P13236) (Selective) (p*K*_i_ 7.1–7.8) [328, 357]	CCL26 (*CCL26*, Q9Y258) (Selective) (pIC_50_ 8.5) [1465]	CXCL10 (*CXCL10*, P02778) (Selective), CXCL11 (*CXCL11*, O14625) (Selective), CXCL9 (*CXCL9*, Q07325) (Selective)
Antagonists	–	–	–
Selective antagonists	BX 471 (p*K*_i_ 8.2–9) [1098], compound 2b‐1 [PMID: 12614873] (pIC_50_ 8.7) [1368], CP‐481,715 (p*K*_d_ 8) [614], UCB35625 (pIC_50_ 8) [1625]	GSK Compound 34 (p*K*_i_ 7.6)	banyu (I) (Inverse agonist) (p*K*_i_ 8.5) [1977], SB328437 (p*K*_i_ 8.4), BMS compound 87b (p*K*_i_ 8.1) [1964]
Antibodies	–	–	–
Labelled ligands	[^125^I]CCL7 (human) (Agonist) (p*K*_d_ 9.2) [127], [^125^I]CCL3 (human) (Agonist) (p*K*_d_ 8–8.8) [127, 623, 1646], [^125^I]CCL5 (human) (Agonist) (p*K*_d_ 8.2) [1646]	[^125^I]CCL2 (human) (Agonist), [^125^I]CCL7 (human) (Agonist)	[^125^I]CCL11 (human) (Antagonist) (p*K*_d_ 8.3) [1977], [^125^I]CCL5 (human) (Agonist), [^125^I]CCL7 (human) (Agonist)

**Table:** 

Nomenclature	CCR4	CCR5
HGNC, UniProt	*CCR4*, P51679	*CCR5*, P51681
Endogenous agonists	CCL22 (*CCL22*, O00626) (Selective) (pIC_50_ 9.2) [822], CCL17 (*CCL17*, Q92583) (Selective) (pIC_50_ 8.7) [822]	CCL5 (*CCL5*, P13501) (p*K*_i_ 9.2–9.7) [75, 1364, 1611], CCL4 (*CCL4*, P13236) (Selective) (p*K*_i_ 9.4–9.6) [1364, 1611], CCL8 (*CCL8*, P80075) (p*K*_i_ 9.3) [1611], CCL3 (*CCL3*, P10147) (p*K*_i_ 8–8.9) [1364, 1611, 2134], CCL11 (*CCL11*, P51671) (pIC_50_ 7.7) [157], CCL2 (*CCL2*, P13500) (p*K*_i_ 7.5) [1364], CCL14 (*CCL14*, Q16627) (p*K*_i_ 7.2) [1364], CCL16 (*CCL16*, O15467)
Agonists	vMIP‐III	R5‐HIV‐1 gp120
Endogenous antagonists	–	CCL7 (*CCL7*, P80098) (Selective) (p*K*_i_ 7.5) [1364]
Antagonists	–	vicriviroc (p*K*_i_ 9.1) [1805], ancriviroc (p*K*_i_ 7.8–8.7) [1173, 1455, 1805]
Selective antagonists	–	E913 (pIC_50_ 8.7) [1174], aplaviroc (p*K*_i_ 8.5) [1173], maraviroc (pIC_50_ 8.1) [1364], TAK‐779 (p*K*_i_ 7.5) [1173], MRK‐1 [1023] – Rat
Antibodies	mogamulizumab (Inhibition) [51, 1731]	–
Labelled ligands	[^125^I]CCL17 (human) (Agonist), [^125^I]CCL27 (human) (Agonist)	[^125^I]CCL4 (human) (Agonist) (p*K*_d_ 9.6) [1364], [^125^I]CCL3 (human) (Agonist), [^125^I]CCL5 (human) (Agonist), [^125^I]CCL8 (human) (Agonist)

**Table:** 

Nomenclature	CCR6	CCR7	CCR8	CCR9	CCR10
HGNC, UniProt	*CCR6*, P51684	*CCR7*, P32248	*CCR8*, P51685	*CCR9*, P51686	*CCR10*, P46092
Endogenous agonists	CCL20 (*CCL20*, P78556) (pIC_50_ 7.9–8.5) [18, 74, 1526], beta‐defensin 4A (*DEFB4A* *DEFB4B*, O15263) (Selective) [2081]	CCL21 (*CCL21*, O00585) (Selective) (pIC_50_ 9.3) [2099], CCL19 (*CCL19*, Q99731) (Selective) (pIC_50_ 7.7–9, median 8.6) [1449, 2098, 2099]	CCL1 (*CCL1*, P22362) (Selective) (pIC_50_ 8.5–9.8) [387, 710, 824], CCL8 {Mouse} – Mouse	CCL25 (*CCL25*, O15444) (Selective)	CCL27 (*CCL27*, Q9Y4X3) (Selective), CCL28 (*CCL28*, Q9NRJ3) (Selective)
Agonists	–	–	vMIP‐I (pIC_50_ 8.9–9.9) [387, 824]	–	–
Selective antagonists	–	–	vMCC‐I (pIC_50_ 9.4) [387]	–	–
Labelled ligands	[^125^I]CCL20 (human) (Agonist) (p*K*_d_∼10) [641]	[^125^I]CCL19 (human) (Agonist), [^125^I]CCL21 (human) (Agonist) [856]	[^125^I]CCL1 (human) (Agonist) (p*K*_d_ 8.9–9.7) [824, 1597]	[^125^I]CCL25 (human) (Agonist)	–

**Table:** 

Nomenclature	CXCR1	CXCR2	CXCR3
HGNC, UniProt	*CXCR1*, P25024	*CXCR2*, P25025	*CXCR3*, P49682
Endogenous agonists	CXCL8 (*CXCL8*, P10145) (p*K*_i_ 8.8–9.5) [142, 675, 1068, 2032, 2049], CXCL6 (*CXCL6*, P80162) (p*K*_i_ 7) [2053]	CXCL1 (*CXCL1*, P09341) (Selective) (p*K*_i_ 8.4–9.7) [675, 1068, 2049], CXCL8 (*CXCL8*, P10145) (p*K*_i_ 8.8–9.5) [142, 675, 1068, 2032, 2049], CXCL7 (*PPBP*, P02775) (Selective) (pIC_50_ 6.3–9.3) [16], CXCL3 (*CXCL3*, P19876) (Selective) (pIC_50_ 7.8–9.2) [16], CXCL2 (*CXCL2*, P19875) (Selective) (pIC_50_ 7–9.1) [16], CXCL5 (*CXCL5*, P42830) (Selective) (pIC_50_ 6.9–9) [16], CXCL6 (*CXCL6*, P80162) (p*K*_d_ 7) [2053]	CXCL11 (*CXCL11*, O14625) (Selective) (p*K*_i_ 10.4–10.5) [734], CXCL10 (*CXCL10*, P02778) (Selective) (p*K*_i_ 7.8–9.8) [734, 2006], CXCL9 (*CXCL9*, Q07325) (Selective) (p*K*_i_ 7.3–8.3) [734, 2006]
Agonists	vCXCL1 (pIC_50_ 7.4) [1158], HIV‐1 matrix protein p17 (p*K*_d_ 5.7) [602]	vCXCL1 (pIC_50_ 8.2) [1158], HIV‐1 matrix protein p17 (p*K*_d_ 6.9) [602]	–
Selective agonists	–	–	–
Endogenous antagonists	–	–	CCL11 (*CCL11*, P51671) (Selective) (p*K*_i_ 7.2) [2006], CCL7 (*CCL7*, P80098) (Selective) (p*K*_i_ 6.6) [2006]
Antagonists	–	–	–
Selective antagonists	–	navarixin (pIC_50_ 10.3) [78, 456], danirixin (pIC_50_ 7.9) [1285], SB 225002 (pIC_50_ 7.7) [2016], elubirixin (pIC_50_ 7.7) [78], SX‐517 (pIC_50_ 7.2) [1172]	–
Allosteric modulators	reparixin (Negative) (pIC_50_ 9) [142]	reparixin (Negative) (pIC_50_ 6.4) [142]	–
Labelled ligands	[^125^I]CXCL8 (human) (Agonist) (p*K*_d_ 8.9–9.6) [675, 1584]	[^125^I]CXCL8 (human) (Agonist) (p*K*_d_ 9–9.4) [675, 1584], [^125^I]CXCL1 (human) (Agonist), [^125^I]CXCL5 (human) (Agonist), [^125^I]CXCL7 (human) (Agonist)	[^125^I]CXCL10 (human) (Agonist), [^125^I]CXCL11 (human) (Agonist)
Comments	Reports on the expression of native CXCR1 by mouse leukocytes are not conclusive. There are reports on the existence of mouse *Cxcr1* and on *Cxcr1* knockout mice, but the distinct function of the gene and of its knockout phenotype are unclear [118, 351, 1297, 1628, 1794].	–	–

**Table:** 

Nomenclature	CXCR4	CXCR5	CXCR6
HGNC, UniProt	*CXCR4*, P61073	*CXCR5*, P32302	*CXCR6*, O00574
Endogenous agonists	CXCL12*α* (*CXCL12*, P48061) (Selective) (p*K*_d_ 7.7–8.2) [746, 1136], CXCL12*β* (*CXCL12*, P48061) (Selective) (p*K*_d_ 7.9) [746]	CXCL13 (*CXCL13*, O43927) (Selective) (p*K*_d_ 7.3) [97]	CXCL16 (*CXCL16*, Q9H2A7) (Selective) (p*K*_d_ 9) [2026]
Agonists	–	–	
Selective agonists	ALX40‐4C (Partial agonist) (pIC_50_ 6.1) [2121], X4‐HIV‐1 gp120	–	
Endogenous antagonists	–	–	
Antagonists	plerixafor (p*K*_i_ 7) [2121]	–	
Selective antagonists	T134 (pIC_50_ 8.4) [1856], AMD070 (pIC_50_ 7.9) [1750], HIV‐Tat	–	
Allosteric modulators	–	–	
Labelled ligands	[^125^I]CXCL12*α* (human) (Agonist) (p*K*_d_ 8.1–8.4) [421, 746]	[^125^I]CXCL13 (mouse) (Agonist) [222] – Mouse	[^125^I]CXCL16 (human) (Agonist)
Comments	–	–	–

**Table:** 

Nomenclature	CX_3_CR1	XCR1	ACKR1	ACKR2
HGNC, UniProt	*CX3CR1*, P49238	*XCR1*, P46094	*ACKR1*, Q16570	*ACKR2*, O00590
Endogenous ligands	–	–	CXCL5 (*CXCL5*, P42830), CXCL6 (*CXCL6*, P80162), CXCL8 (*CXCL8*, P10145), CXCL11 (*CXCL11*, O14625), CCL2 (*CCL2*, P13500), CCL5 (*CCL5*, P13501), CCL7 (*CCL7*, P80098), CCL11 (*CCL11*, P51671), CCL14 (*CCL14*, Q16627), CCL17 (*CCL17*, Q92583)	CCL2 (*CCL2*, P13500), CCL3 (*CCL3*, P10147), CCL4 (*CCL4*, P13236), CCL5 (*CCL5*, P13501), CCL7 (*CCL7*, P80098), CCL8 (*CCL8*, P80075), CCL11 (*CCL11*, P51671), CCL13 (*CCL13*, Q99616), CCL14 (*CCL14*, Q16627), CCL17 (*CCL17*, Q92583), CCL22 (*CCL22*, O00626)
Endogenous agonists	CX_3_CL1 (*CX3CL1*, P78423) (Selective) (pIC_50_ 8.9) [577]	XCL1 (*XCL1*, P47992) (Selective), XCL2 (*XCL2*, Q9UBD3) (Selective)	–	–
Labelled ligands	[^125^I]CX_3_CL1 (human) (Agonist)	–	–	
Comments	–	When fused with secreted alkaline phophatase (SEAP), XCL1 functions as a probe at XCR1	ACKR1 is used by *Plasmodium vivax* and *Plasmodium knowlsei* for entering erythrocytes.	

**Table:** 

Nomenclature	ACKR3	ACKR4	CCRL2
HGNC, UniProt	*ACKR3*, P25106	*ACKR4*, Q9NPB9	*CCRL2*, O00421
Endogenous ligands	–	–	chemerin C‐terminal peptide, CCL19 (*CCL19*, Q99731) [95]
Endogenous agonists	CXCL12*α* (*CXCL12*, P48061) (pEC_50_ 7.5–7.9) [640, 1785], CXCL11 (*CXCL11*, O14625), adrenomedullin {Mouse} [965] – Mouse	CCL19 (*CCL19*, Q99731) (p*K*_i_ 8.4) [1997], CCL25 (*CCL25*, O15444) (p*K*_i_ 7.6) [1997], CCL21 (*CCL21*, O00585) (p*K*_i_ 6.9) [1997]	–

### Comments

Mouse Cxcr binds iodinated mouse KC (CXCL1{Mouse}) and mouse MIP‐2 (CXCL2{Mouse}) with high affinity (mouse KC and MIP‐2 are homologues of human CXCL1(*CXCL1*, P09341), CXCL2(*CXCL2*, P19875) and CXCL3(*CXCL3*, P19876)), but shows low affinity for human IL‐8 (CXCL8(*CXCL8*, P10145)).

Specific chemokine receptors facilitate cell entry by microbes, such as ACKR1 for *Plasmodium vivax*, and CCR5 for HIV‐1. Virally encoded chemokine receptors are known (*e.g.* US28, a homologue of CCR1 from human cytomegalovirus and ORF74, which encodes a homolog of CXCR2 in *Herpesvirus saimiri* and Herpesvirus‐68), but their role in viral life cycles is not established. Viruses can exploit or subvert the chemokine system by producing chemokine antagonists and scavengers.

The CC chemokine family (CCL1‐28) includes I309 (CCL1(*CCL1*, P22362)), MCP‐1 (CCL2(*CCL2*, P13500)), MIP‐1*α*(CCL3(*CCL3*, P10147)), MIP‐1*β*(CCL4(*CCL4*, P13236)), RANTES (CCL5(*CCL5*, P13501)), MCP‐3 (CCL7(*CCL7*, P80098)), MCP‐2 (CCL8(*CCL8*, P80075)), eotaxin (CCL11(*CCL11*, P51671)), MCP‐4 (CCL13(*CCL13*, Q99616)), HCC‐1 (CCL14(*CCL14*, Q16627)), Lkn‐1/HCC‐2 (CCL15(*CCL15*, Q16663)), TARC (CCL17(*CCL17*, Q92583)), ELC (CCL19(*CCL19*, Q99731)), LARC (CCL20(*CCL20*, P78556)), SLC (CCL21(*CCL21*, O00585)), MDC (CCL22(*CCL22*, O00626)), MPIF‐1 (CCL23(*CCL23*, P55773)), eotaxin‐2 (CCL24(*CCL24*, O00175)), TECK (CCL25(*CCL25*, O15444)), eotaxin (CCL26(*CCL26*, Q9Y258)), eskine/CTACK (CCL27(*CCL27*, Q9Y4X3)) and MEC (CCL28(*CCL28*, Q9NRJ3)). The CXC chemokine family (CXCL1‐17) includes GRO*α*(CXCL1(*CXCL1*, P09341)), GRO*β*(CXCL2(*CXCL2*, P19875)), GRO*γ*(CXCL3(*CXCL3*, P19876)), platelet factor 4 (CXCL4(*PF4*, P02776)), ENA78 (CXCL5(*CXCL5*, P42830)), GCP‐2 (CXCL6(*CXCL6*, P80162)), NAP‐2 (CXCL7(*PPBP*, P02775)), IL‐8 (CXCL8(*CXCL8*, P10145)), MIG (CXCL9(*CXCL9*, Q07325)), IP10 (CXCL10(*CXCL10*, P02778)), I‐TAC (CXCL11(*CXCL11*, O14625)), SDF‐1 (CXCL12, *i.e.* CXCL12*α*(*CXCL12*, P48061) and CXCL12*β*(*CXCL12*, P48061)), BLC (CXCL13(*CXCL13*, O43927)), BRAK (CXCL14(*CXCL14*, O95715)), mouse lungkine (CXCL15 {Mouse}) SR‐PSOX (CXCL16(*CXCL16*, Q9H2A7)) and CXCL17(*CXCL17*, Q6UXB2). The CX_3_C chemokine (CX_3_CL1(*CX3CL1*, P78423)) is also known as fractalkine (neurotactin in the mouse). Like CXCL16(*CXCL16*, Q9H2A7), and unlike other chemokines, CX_3_CL1(*CX3CL1*, P78423) is multimodular containing a chemokine domain, an elongated mucin‐like stalk, a transmembrane domain and a cytoplasmic tail. Both plasma membrane‐associated and shed forms have been identified. The C chemokine (XCL1(*XCL1*, P47992)) is also known as lymphotactin.

Two chemokine receptor antagonists have now been approved by the FDA: the CCR5 antagonist maraviroc (Pfizer) for treatment of HIV/AIDS in patients with CCR5‐using strains; and the CXCR4 antagonist plerixafor (Sanofi) for hematopoietic stem cell mobilization with G‐CSF(*CSF3*, P09919) in patients undergoing transplantation in the context of chemotherapy for lymphoma and multiple myeloma.

### Further Reading

Bachelerie F *et al*. (2015) An atypical addition to the chemokine receptor nomenclature: IUPHAR Review ~15~. *Br. J. Pharmacol.* [PMID:25958743]

Koelink PJ *et al*. (2012) Targeting chemokine receptors in chronic inflammatory diseases: an extensive review. *Pharmacol. Ther.* **133**: 1‐18 [PMID:21839114]

Murphy PM. (2002) International Union of Pharmacology. XXX. Update on chemokine receptor nomenclature. *Pharmacol. Rev.* **54**: 227‐9 [PMID:12037138]

Murphy PM *et al*. (2000) International Union of Pharmacology. XXII. Nomenclature for chemokine receptors. *Pharmacol. Rev.* **52**: 145‐176 [PMID:10699158]

Muñoz LM *et al*. (2011) Receptor oligomerization: a pivotal mechanism for regulating chemokine function. *Pharmacol. Ther.* **131**: 351‐8 [PMID:21600920]

Scholten DJ *et al*. (2012) Pharmacological modulation of chemokine receptor function. *Br. J. Pharmacol.* **165**: 1617‐43 [PMID:21699506]

Szpakowska M *et al*. (2012) Function, diversity and therapeutic potential of the N‐terminal domain of human chemokine receptors. *Biochem. Pharmacol.* **84**: 1366‐80 [PMID:22935450]

White GE *et al*. (2013) CC chemokine receptors and chronic inflammation–therapeutic opportunities and pharmacological challenges. *Pharmacol. Rev.* **65**: 47‐89 [PMID:23300131]

## Cholecystokinin receptors

### Overview

Cholecystokinin receptors (**nomenclature as agreed by the NC‐IUPHAR Subcommittee on CCK receptors [** **1403** **]**) are activated by the endogenous peptides cholecystokinin‐8 (CCK‐8(*CCK*, P06307)), CCK‐33(*CCK*, P06307), CCK‐58(*CCK*, P06307) and gastrin (gastrin‐17(*GAST*, P01350)). There are only two distinct subtypes of CCK receptors, CCK_1_ and CCK_2_ receptors [992, 1986], with some alternatively spliced forms most often identified in neoplastic cells. The CCK receptor subtypes are distinguished by their peptide selectivity, with the CCK_1_ receptor requiring the carboxyl‐terminal heptapeptide‐amide that includes a sulfated tyrosine for high affinity and potency, while the CCK_2_ receptor requires only the carboxyl‐terminal tetrapeptide shared by both CCK and gastrin peptides. These receptors have characteristic and distinct distributions, with both present in both the central nervous system and peripheral tissues.

**Table:** 

Nomenclature	CCK_1_ receptor	CCK_2_ receptor
HGNC, UniProt	*CCKAR*, P32238	*CCKBR*, P32239
Rank order of potency	CCK‐8 (*CCK*, P06307) ≫gastrin‐17 (*GAST*, P01350), desulfated cholecystokinin‐8>CCK‐4 (*CCK*, P06307)	CCK‐8 (*CCK*, P06307) ≥gastrin‐17 (*GAST*, P01350), desulfated cholecystokinin‐8, CCK‐4 (*CCK*, P06307)
Endogenous agonists	–	desulfated cholecystokinin‐8 (pIC_50_ 8.3–8.7) [1071], gastrin‐17 (*GAST*, P01350) (Selective) (pIC_50_ 8.3) [805] – Mouse, CCK‐4 (*CCK*, P06307) (pIC_50_ 7.5) [832], desulfated gastrin‐14 (*GAST*, P01350), desulfated gastrin‐17 (*GAST*, P01350), desulfated gastrin‐34 (*GAST*, P01350), desulfated gastrin‐71 (*GAST*, P01350), gastrin‐14 (*GAST*, P01350), gastrin‐34 (*GAST*, P01350), gastrin‐71 (*GAST*, P01350)
Selective agonists	A‐71623 (pIC_50_ 8.4) [63] – Rat, JMV180 (pIC_50_ 8.3) [926], GW‐5823 (pIC_50_ 7.6) [737]	RB‐400 (p*K*_i_ 9.1) [123] – Rat, PBC‐264 (pIC_50_ 9.1) [844] – Rat
Antagonists	lintitript (pIC_50_ 8.3) [632]	–
Selective antagonists	devazepide (pIC_50_ 9.7) [805] – Rat, T‐0632 (pIC_50_ 9.6) [1861] – Rat, PD‐140548 (pIC_50_ 8.6) [1748] – Rat, lorglumide (pIC_50_ 6.7–8.2) [805, 834] – Rat	YF‐476 (pIC_50_ 9.7) [196, 1854], GV150013 (pIC_50_ 9.4) [1930], L‐740093 (pIC_50_ 9.2) [1398], YM‐022 (pIC_50_ 9.2) [1398], JNJ‐26070109 (pIC_50_ 8.5) [1336], L‐365260 (pIC_50_ 8.4) [1071], RP73870 (pIC_50_ 8) [1115] – Rat, LY262691 (pIC_50_ 7.5) [1561] – Rat
Labelled ligands	[^3^H]devazepide (Antagonist) (p*K*_d_ 9.7) [292], [^125^I]DTyr‐Gly‐[(Nle28,31)CCK‐26‐33 (Agonist) (pIC_50_ 9) [1527]	[^3^H]PD140376 (Antagonist) (p*K*_i_ 9.7–10) [809] – Guinea pig, [^125^I]PD142308 (Antagonist) (p*K*_d_ 9.6) [781] – Guinea pig, [^125^I]DTyr‐Gly‐[(Nle28,31)CCK‐26‐33 (Agonist) (pIC_50_ 9) [1527], [^125^I]gastrin (Agonist) (pIC_50_ 9), [^3^H]gastrin (Agonist) (pIC_50_ 9), [^3^H]L365260 (Antagonist) (p*K*_d_ 8.2–8.5) [1398], [^125^I]‐BDZ_2_ (Antagonist) (p*K*_i_ 8.4) [25]

### Comments

While a cancer‐specific CCK receptor has been postulated to exist, which also might be responsive to incompletely processed forms of CCK (Gly‐extended forms), this has never been isolated. An alternatively spliced form of the CCK_2_ receptor in which intron 4 is retained, adding 69 amino acids to the intracellular loop 3 (ICL3) region, has been described to be present particularly in certain neoplasms where mRNA mis‐splicing has been commonly observed [1764], but it is not clear that this receptor splice form plays a special role in carcinogenesis. Another alternative splicing event for the CCK_2_ receptor was reported [1782], with alternative donor sites in exon 4 resulting in long (452 amino acids) and short (447 amino acids) forms of the receptor differing by five residues in ICL3, however, no clear functional differences have been observed.

### Further Reading

Cawston EE *et al*. (2010) Therapeutic potential for novel drugs targeting the type 1 cholecystokinin receptor. *Br. J. Pharmacol.* **159**: 1009‐21 [PMID:19922535]

Dockray GJ. (2009) Cholecystokinin and gut‐brain signalling. *Regul. Pept.* **155**: 6‐10 [PMID:19345244]

Dufresne M *et al*. (2006) Cholecystokinin and gastrin receptors. *Physiol. Rev.* **86**: 805‐47 [PMID:16816139]

Miller LJ *et al*. (2008) Structural basis of cholecystokinin receptor binding and regulation. *Pharmacol. Ther.* **119**: 83‐95 [PMID:18558433]

## Class Frizzled GPCRs

### Overview

Receptors of the Class Frizzled (FZD, **nomenclature as agreed by the NC‐IUPHAR subcommittee on the Class Frizzled GPCRs [** **1676** **]**), are GPCRs originally identified in*Drosophila* [285], which are highly conserved across species. FZDs are activated by WNTs, which are cysteine‐rich lipoglycoproteins with fundamental functions in ontogeny and tissue homeostatis. FZD signalling was initially divided into two pathways, being either dependent on the accumulation of the transcription regulator *β*‐catenin(*CTNNB1*, P35222) or being *β*‐catenin‐independent (often referred to as canonical *vs.* non‐canonical WNT/FZD signalling, respectively). WNT stimulation of FZDs can, in cooperation with the low density lipoprotein receptors *LRP5* (O75197) and *LRP6*(O75581), lead to the inhibition of a constitutively active destruction complex, which results in the accumulation of *β*‐catenin and subsequently its translocation to the nucleus. *β*‐Catenin, in turn, modifies gene transcription by interacting with TCF/LEF transcription factors. *β*‐Catenin‐independent FZD signalling is far more complex with regard to the diversity of the activated pathways. WNT/FZD signalling can lead to the activation of pertussis toxin‐sensitive heterotrimeric G proteins [939], the elevation of intracellular calcium [1757], activation of cGMP‐specific PDE6 [17] and elevation of cAMP as well as RAC‐1, JNK, Rho and Rho kinase signalling [695]. Furthermore, the phosphoprotein Disheveled constitutes a key player in WNT/FZD signalling. As with other GPCRs, members of the Frizzled family are functionally dependent on the arrestin scaffolding protein for internalization [306], as well as for *β*‐catenin‐dependent [235] and ‐independent [236, 940] signalling. The pattern of cell signalling is complicated by the presence of additional ligands, which can enhance or inhibit FZD signalling (secreted Frizzled‐related proteins (sFRP), Wnt‐inhibitory factor(*WIF1*, Q9Y5W5) (WIF), sclerostin(*SOST*, Q9BQB4) or Dickkopf (DKK)), as well as modulatory (co)‐receptors with Ryk, ROR1, ROR2 and Kremen, which may also function as independent signalling proteins.

**Table:** 

Nomenclature	FZD_1_	FZD_2_	FZD_3_	FZD_4_	FZD_5_
HGNC, UniProt	*FZD1*, Q9UP38	*FZD2*, Q14332	*FZD3*, Q9NPG1	*FZD4*, Q9ULV1	*FZD5*, Q13467

**Table:** 

Nomenclature	FZD_6_	FZD_7_	FZD_8_	FZD_9_	FZD_10_
HGNC, UniProt	*FZD6*, O60353	*FZD7*, O75084	*FZD8*, Q9H461	*FZD9*, O00144	*FZD10*, Q9ULW2

**Table:** 

Nomenclature	SMO
HGNC, UniProt	*SMO*, Q99835
Antagonists	saridegib (pIC_50_ 8.9) [1904], glasdegib (pIC_50_ 8.3) [1342], erismodegib (p*K*_i_ 8.2) [1979]
Selective antagonists	vismodegib (p*K*_i_ 7.8) [1979]

### Comments

There is limited knowledge about WNT/FZD specificity and which molecular entities determine the signalling outcome of a specific WNT/FZD pair. Understanding of the coupling to G proteins is incomplete (see [423]). There is also a scarcity of information on basic pharmacological characteristics of FZDs, such as binding constants, ligand specificity or concentration‐response relationships [937].

**Ligands associated with FZD signalling**

***WNTs***: Wnt‐1(*WNT1*, P04628), Wnt‐2(*WNT2*, P09544) (also known as Int‐1‐related protein), Wnt‐2b(*WNT2B*, Q93097) (also known as WNT‐13), Wnt‐3(*WNT3*, P56703) , Wnt‐3a(*WNT3A*, P56704), Wnt‐4(*WNT4*, P56705), Wnt‐5a(*WNT5A*, P41221) , Wnt‐5b(*WNT5B*, Q9H1J7), Wnt‐6(*WNT6*, Q9Y6F9), Wnt‐7a(*WNT7A*, O00755), Wnt‐7b(*WNT7B*, P56706), Wnt‐8a(*WNT8A*, Q9H1J5), Wnt‐8b(*WNT8B*, Q93098), Wnt‐9a(*WNT9A*, O14904) (also known as WNT‐14), Wnt‐9b(*WNT9B*, O14905) (also known as WNT‐15 or WNT‐14b), Wnt‐10a(*WNT10A*, Q9GZT5), Wnt‐10b(*WNT10B*, O00744) (also known as WNT‐12), Wnt‐11(*WNT11*, O96014) and Wnt‐16(*WNT16*, Q9UBV4).

***Extracellular proteins that interact with FZDs**:* norrin(*NDP*, Q00604), R‐spondin‐1(*RSPO1*, Q2MKA7), R‐spondin‐2(*RSPO2*, Q6UXX9) , R‐spondin‐3(*RSPO3*, Q9BXY4), R‐spondin‐4(*RSPO4*, Q2I0M5), sFRP‐1(*SFRP1*, Q8N474), sFRP‐2(*SFRP2*, Q96HF1), sFRP‐3(*FRZB*, Q92765), sFRP‐4(*SFRP4*, Q6FHJ7), sFRP‐5(*SFRP5*, Q6FHJ7).

***Extracellular proteins that interact with WNTs or LRPs:*** Dickkopf 1(*DKK1*, O94907), *WIF1* (Q9Y5W5), sclerostin(*SOST*, Q9BQB4), kremen 1(*KREMEN1*, Q96MU8) and kremen 2 (*KREMEN2*, Q8NCW0)

***Small exogenous ligands:*** Foxy‐5 [1835], Box‐5, UM206 [1031], and XWnt8 (P28026) also known as mini‐Wnt8.

### Further Reading

Dijksterhuis JP *et al*. (2013) WNT/Frizzled signaling: receptor‐ligand selectivity with focus on FZD‐G protein signaling and its physiological relevance. *Br J Pharmacol* [PMID:24032637]

King TD *et al*. (2012) The Wnt/*β*‐catenin signaling pathway: a potential therapeutic target in the treatment of triple negative breast cancer. *J. Cell. Biochem.* **113**: 13‐8 [PMID:21898546]

King TD *et al*. (2012) Frizzled7 as an emerging target for cancer therapy. *Cell. Signal.* **24**: 846‐51 [PMID:22182510]

Koval A *et al*. (2011) Yellow submarine of the Wnt/Frizzled signaling: submerging from the G protein harbor to the targets. *Biochem. Pharmacol.* **82**: 1311‐9 [PMID:21689640]

Schuijers J *et al*. (2012) Adult mammalian stem cells: the role of Wnt, Lgr5 and R‐spondins. *EMBO J.* **31**: 2685‐96 [PMID:22617424]

Schulte G. (2010) International Union of Basic and Clinical Pharmacology. LXXX. The class Frizzled receptors. *Pharmacol. Rev.* **62**: 632‐67 [PMID:21079039]

Schulte G *et al*. (2010) beta‐Arrestins ‐ scaffolds and signalling elements essential for WNT/Frizzled signalling pathways? *Br. J. Pharmacol.* **159**: 1051‐8 [PMID:19888962]

## Complement peptide receptors

### Overview

Complement peptide receptors (**nomenclature as agreed by the NC‐IUPHAR subcommittee on Complement peptide receptors [** **967** **]**) are activated by the endogenous  75 amino‐acid anaphylatoxin polypeptides C3a(*C3*, P01024). C4a(*C4A*, P0C0L4) and C5a(*C5*, P01031), generated upon stimulation of the complement cascade.

**Table:** 

Nomenclature	C3a receptor	C5a_1_ receptor	C5a_2_ receptor
HGNC, UniProt	*C3AR1*, Q16581	*C5AR1*, P21730	*C5AR2*, Q9P296
Rank order of potency	C3a (*C3*, P01024) >C5a (*C5*, P01031) [41]	C5a (*C5*, P01031), C5a des‐Arg (*C5*) >C3a (*C3*, P01024) [41]	–
Endogenous agonists	–	ribosomal protein S19 (*RPS19*, P39019) [2071]	–
Agonists	E7 (pEC_50_ 8.7) [43], compound 21 [PMID: 25259874] (pEC_50_ 7.7) [1571], SQ007‐5 (Partial agonist) (pEC_50_ 6.7) [124], Ac‐RHYPLWR (pEC_50_ 6) [672]	*N*‐methyl‐Phe‐Lys‐Pro‐D‐Cha‐Cha‐D‐Arg‐CO_2_H (pIC_50_ 7.6) [916, 989]	–
Antagonists	SB290157 (pIC_50_ 7.6) [40], compound 4 [PMID: 25259874] (pIC_50_ 5.9) [1571]	CHIPS (p*K*_d_ 9) [1522], W54011 (p*K*_i_ 8.7) [1819], AcPhe‐Orn‐Pro‐D‐Cha‐Trp‐Arg (pIC_50_ 7.9) [2039], *N*‐methyl‐Phe‐Lys‐Pro‐D‐Cha‐Trp‐D‐Arg‐CO_2_H (pIC_50_ 7.2) [989]	–
Labelled ligands	[^125^I]C3a (human) (Agonist) (p*K*_d_ 8.4) [296]	[^125^I]C5a (human) (Agonist) (p*K*_d_ 8.7) [803]	[^125^I]C5a (human) (Agonist)
Comments	–	–	Binds C5a complement factor, but appears to lack G protein signalling and has been termed a decoy receptor [1684].

### Comments

SB290157 has also been reported to have agonist properties at the C3a receptor [1218]. The putative chemoattractant receptor termed C5a_2_ (also known as GPR77, C5L2) binds [^125^I]C5a with no clear signalling function, but has a putative role opposing inflammatory responses [257, 568, 585]. Binding to this site may be displaced with the rank order C5a des‐Arg(*C5*)>C5a(*C5*, P01031) [257, 1440] while there is controversy over the ability of C3a(*C3*, P01024) and C3a des Arg(*C3*, P01024) to compete [778, 894, 895, 1440]. C5a_2_ appears to lack G protein signalling and has been termed a decoy receptor [1684]. However, C5a_2_ does recruit arrestin after ligand binding, which might provide a signaling pathway for this receptor [89, 1937], and forms heteromers with C5a_1_. C5a, but not C5a‐des Arg, induces upregulation of heteromer formation between complement C5a receptors C5aR and C5L2 [380]. There are also reports of pro‐inflammatory activity of C5a_2_, mediated by HMGB1, but the signaling pathway that underlies this is currently unclear (reviewed in [1095]).

### Further Reading

Hajishengallis G. (2010) Complement and periodontitis. *Biochem. Pharmacol.* **80**: 1992‐2001 [PMID:20599785]

Klos A *et al*. (2013) International Union of Pharmacology. LXXXVII. Complement peptide C5a, C4a, and C3a receptors. *Pharmacol. Rev.* **65**: 500‐43 [PMID:23383423]

Manthey HD *et al*. (2009) Complement component 5a (C5a). *Int. J. Biochem. Cell Biol.* **41**: 2114‐7 [PMID:19464229]

Monk PN *et al*. (2007) Function, structure and therapeutic potential of complement C5a receptors. *Br. J. Pharmacol.* **152**: 429‐48 [PMID:17603557]

Sacks SH. (2010) Complement fragments C3a and C5a: the salt and pepper of the immune response. *Eur. J. Immunol.* **40**: 668‐70 [PMID:20186746]

## Corticotropin‐releasing factor receptors

### Overview

Corticotropin‐releasing factor (CRF, **nomenclature as agreed by the NC‐IUPHAR subcommittee on Corticotropin‐releasing Factor Receptors [** **716** **]**) receptors are activated by the endogenous peptides corticotrophin‐releasing hormone (*CRH*, P06850), a 41 amino‐acid peptide, urocortin 1(*UCN*, P55089), 40 amino‐acids, urocortin 2 (*UCN2*, Q96RP3), 38 amino‐acids and urocortin 3 (*UCN3*, Q969E3), 38 amino‐acids. CRF_1_ and CRF_2_ receptors are activated non‐selectively by corticotrophin‐releasing hormone (*CRH*, P06850) and urocortin 1 (*UCN*, P55089). Binding to CRF receptors can be conducted using [^125^I]Tyr^0^‐CRF or [^125^I]Tyr^0^‐sauvagine with *K*_d_ values of 0.1‐0.4 nM. CRF_1_ and CRF_2_ receptors are non‐selectively antagonized by *α*‐helical CRF, D‐Phe‐CRF‐(12‐41) and astressin.

**Table:** 

Nomenclature	CRF_1_ receptor	CRF_2_ receptor
HGNC, UniProt	*CRHR1*, P34998	*CRHR2*, Q13324
Endogenous agonists	–	urocortin 2 (*UCN2*, Q96RP3) (Selective) (p*K*_d_ 8.5–8.6) [392], urocortin 3 (*UCN3*, Q969E3) (Selective) (p*K*_d_ 7.9–8) [392]
Antagonists	SSR125543A (p*K*_i_ 8.7) [663]	–
Selective antagonists	CP 154,526 (pIC_50_ 9.3–10.4) [1153] – Rat, DMP696 (p*K*_i_ 8.3–9) [726], NBI27914 (p*K*_i_ 8.3–9) [298], R121919 (p*K*_i_ 8.3–9) [2133], antalarmin (p*K*_i_ 8.3–9) [2001], CP376395 (pIC_50_ 8.3) [307] – Rat, CRA1000 (pIC_50_ 6.4–7.1) [284]	antisauvagine (p*K*_d_ 8.8–9.6) [394], K41498 (p*K*_i_ 9.2) [1048], K31440 (p*K*_i_ 8.7–8.8) [1622]

### Comments

A CRF binding protein has been identified (*CRHBP*, P24387) to which both corticotrophin‐releasing hormone(*CRH*, P06850) and urocortin 1(*UCN*, P55089) bind with high affinities, which has been suggested to bind and inactivate circulating corticotrophin‐releasing hormone(*CRH*, P06850) [1489].

### Further Reading

Grammatopoulos DK. (2012) Insights into mechanisms of corticotropin‐releasing hormone receptor signal transduction. *Br. J. Pharmacol.* **166**: 85‐97 [PMID:21883143]

Gysling K. (2012) Relevance of both type‐1 and type‐2 corticotropin releasing factor receptors in stress‐induced relapse to cocaine seeking behaviour. *Biochem. Pharmacol.* **83**: 1‐5 [PMID:21843515]

Hauger RL *et al*. (2003) International Union of Pharmacology. XXXVI. Current status of the nomenclature for receptors for corticotropin‐releasing factor and their ligands. *Pharmacol Rev.* **55**: 21‐26 [PMID:12615952]

Valentino RJ *et al*. (2013) Sex‐biased stress signaling: the corticotropin‐releasing factor receptor as a model. *Mol. Pharmacol.* **83**: 737‐45 [PMID:23239826]

Zhu H *et al*. (2011) Corticotropin‐releasing factor family and its receptors: pro‐inflammatory or anti‐inflammatory targets in the periphery? *Inflamm. Res.* **60**: 715‐21 [PMID:21476084]

## Dopamine receptors

### Overview

Dopamine receptors (**nomenclature as agreed by the NC‐IUPHAR Subcommittee on Dopamine Receptors [1677]**) are commonly divided into D_1_‐like (D_1_ and D_5_) and D_2_‐like (D_2_, D_3_ and D_4_) families, where the endogenous agonist is dopamine.

**Table:** 

Nomenclature	D_1_ receptor	D_2_ receptor
HGNC, UniProt	*DRD1*, P21728	*DRD2*, P14416
Endogenous agonists	dopamine (p*K*_i_ 4.3–5.6) [1823, 1884]	dopamine (p*K*_i_ 4.7–7.2) [245, 545, 1653]
Agonists	fenoldopam (p*K*_i_ 6.5–7.9) [1884]	rotigotine (p*K*_i_ 10.2) [424], cabergoline (Partial agonist) (p*K*_i_ 9–9.2) [1279], aripiprazole (Partial agonist) (p*K*_i_ 9.1) [2111], bromocriptine (p*K*_i_ 7.3–8.3) [545, 1279, 1653], MLS1547 (Biased agonist) (p*K*_i_ 8.2) [544], ropinirole (p*K*_i_ 8.1) [732], apomorphine (Partial agonist) (p*K*_i_ 5.7–7.5) [245, 545, 1279, 1653, 1776], pramipexole (p*K*_i_ 5.1–7.4) [1273, 1653], benzquinamide (p*K*_i_ 5.4) [643]
(Sub)family‐selective agonists	A68930 (pEC_50_ 6.8) [1381], SKF‐38393 (Partial agonist) (p*K*_i_ 6.2–6.8) [1823, 1884]	quinpirole (p*K*_i_ 4.9–7.7) [245, 1273, 1473, 1776, 1778, 1940]
Selective agonists	SKF‐83959 (Biased agonist) (pEC_50_ 9.7) [364], SKF‐81297 (p*K*_i_ 8.7) [46] – Rat	sumanirole (p*K*_i_ 8.1) [1239]
Antagonists	flupentixol (p*K*_i_ 7–8.4) [1823, 1884]	blonanserin (p*K*_i_ 9.9) [1421], pipotiazine (p*K*_i_ 9.7) [1777], perphenazine (p*K*_i_ 8.9–9.6) [1008, 1691], risperidone (p*K*_i_ 9.4) [60], perospirone (p*K*_i_ 9.2) [1692], trifluoperazine (p*K*_i_ 8.9–9) [1008, 1693], asenapine (p*K*_i_ 8.9) [1711], sertindole (p*K*_i_ 8–8.9) [986, 1008, 1691], fluphenazine (p*K*_i_ 8.8) [1647], flupentixol (p*K*_i_ 8.8) [545], pimozide (p*K*_i_ 7–8.8) [545, 1776], olanzapine (p*K*_i_ 8.7) [60], mesoridazine (p*K*_i_ 8.7) [326], ziprasidone (p*K*_i_ 8.6) [60], prochlorperazine (p*K*_i_ 8.4) [68], loxapine (p*K*_i_ 7.9–8.3) [1008, 1693], (‐)‐sulpiride (p*K*_i_ 6.3–8) [545, 1776, 1860], amisulpride (p*K*_i_ 7.9–8) [1195, 1776], metoclopramide (p*K*_i_ 7.5) [1221] – Mouse, quetiapine (p*K*_i_ 7.2) [60], *trans*‐flupenthixol (p*K*_i_ 6.9) [545], clozapine (p*K*_i_ 5.8–6.9) [545, 1164, 1711, 1776, 1860], promazine (p*K*_i_ 6.5) [246]
(Sub)family‐selective antagonists	SCH‐23390 (p*K*_i_ 7.4–9.5) [1823, 1884], SKF‐83556 (p*K*_i_ 9.5) [1823], ecopipam (p*K*_i_ 8.3) [1885]	haloperidol (p*K*_i_ 7.4–8.8) [545, 1164, 1273, 1776, 1885]
Selective antagonists	–	L‐741,626 (p*K*_i_ 7.9–8.5) [655, 1020], domperidone (p*K*_i_ 7.9–8.4) [545, 1776], raclopride (p*K*_i_ 8) [1281], ML321 (p*K*_i_ 7) [2058, 2059]
Labelled ligands	[^3^H]SCH‐23390 (Antagonist) (p*K*_d_ 9.5) [2127]	[^3^H]spiperone (Antagonist) (p*K*_d_ 10.2) [239, 767, 2125] – Rat
Labelled ligands	[^125^I]SCH23982 (Antagonist) (p*K*_d_ 9.5) [408]	[^3^H]raclopride (Antagonist) (p*K*_d_ 8.9) [1028] – Rat

**Table:** 

Nomenclature	D_3_ receptor	D_4_ receptor	D_5_ receptor
HGNC, UniProt	*DRD3*, P35462	*DRD4*, P21917	*DRD5*, P21918
Endogenous agonists	dopamine (p*K*_i_ 6.4–7.3) [245, 545, 1653, 1778]	dopamine (p*K*_i_ 7.6) [1940]	dopamine (p*K*_i_ 6.6) [1823]
Agonists	pramipexole (p*K*_i_ 8.4–8.7) [1273, 1653], bromocriptine (Partial agonist) (p*K*_i_ 7.1–8.2) [545, 1279, 1653], ropinirole (p*K*_i_ 7.7) [732], apomorphine (Partial agonist) (p*K*_i_ 6.1–7.6) [245, 545, 1279, 1653, 1776]	apomorphine (Partial agonist) (p*K*_i_ 8.4) [1279]	–
(Sub)family‐selective agonists	quinpirole (p*K*_i_ 6.4–8) [245, 1273, 1281, 1473, 1653, 1776, 1778, 1940]	quinpirole (p*K*_i_ 7.5) [1279, 1473, 1940]	A68930 (pEC_50_ 6.6) [1381]
Selective agonists	PD 128907 (p*K*_i_ 7.6–7.7) [1539, 1653]	PD168,077 (Partial agonist) (p*K*_i_ 8.8) [995] – Rat, A412997 (p*K*_i_ 8.1) [1319] – Rat, A412997 (p*K*_i_ 8.1) [1319]	–
Antagonists	perospirone (p*K*_i_ 9.6) [1776], sertindole (p*K*_i_ 8–8.8) [60, 1675, 1691], prochlorperazine (p*K*_i_ 8.4) [68], (‐)‐sulpiride (p*K*_i_ 6.7–7.7) [545, 1776, 1860], loxapine (p*K*_i_ 7.7) [1691], domperidone (p*K*_i_ 7.1–7.6) [545, 1776], promazine (p*K*_i_ 6.8) [246]	perospirone (p*K*_i_ 10.1) [1694], sertindole (p*K*_i_ 7.8–9.1) [246, 1691, 1693, 1694], sonepiprazole (p*K*_i_ 8.9) [1668], loxapine (p*K*_i_ 8.1) [1693]	–
(Sub)family‐selective antagonists	haloperidol (p*K*_i_ 7.5–8.6) [545, 1711, 1776, 1885]	haloperidol (p*K*_i_ 8.7–8.8) [1033, 1711, 1885]	SCH‐23390 (p*K*_i_ 7.5–9.5) [1823], SKF‐83556 (p*K*_i_ 9.4) [1823], ecopipam (p*K*_i_ 8.3) [1823]
Selective antagonists	S33084 (p*K*_i_ 9.6) [1278], nafadotride (p*K*_i_ 9.5) [1654], PG01037 (p*K*_i_ 9.2) [656], NGB 2904 (p*K*_i_ 8.8) [2055], SB 277011‐A (p*K*_i_ 8) [1569], (+)‐S‐14297 (p*K*_i_ 6.9–7.9) [1275, 1281]	L745870 (p*K*_i_ 9.4) [1020], A‐381393 (p*K*_i_ 8.8) [1361], L741742 (p*K*_i_ 8.5) [1609], ML398 (p*K*_i_ 7.4) [138]	–
Selective allosteric modulators	SB269652 (Negative) (p*K*_i_∼9) [558]	–	–
Labelled ligands	–	[^3^H]spiperone (Antagonist) (p*K*_d_ 9.5) [749, 1940]	[^3^H]SCH‐23390 (Antagonist) (p*K*_d_ 9.2) [1580]
Labelled ligands	[^3^H]spiperone (Antagonist) (p*K*_d_ 9.9) [767, 2125] – Rat, [^3^H]7‐OH‐DPAT (Agonist) (p*K*_d_ 9.6) [1581], [^3^H]PD128907 (Agonist) (p*K*_d_ 9) [27]	[^125^I]L750667 (Antagonist) (p*K*_d_ 9.8) [1473], [^3^H]NGD941 (Antagonist) (p*K*_d_ 8.3) [1533]	[^125^I]SCH23982 (Antagonist) (p*K*_d_ 9.1) – Unknown

### Comments

The selectivity of many of these agents is less than two orders of magnitude. [^3^H]raclopride exhibits similar high affinity for D_2_ and D_3_ receptors (low affinity for D_4_), but has been used to label D_2_ receptors in the presence of a D_3_‐selective antagonist. [^3^H]7‐OH‐DPAT has similar affinity for D_2_ and D_3_ receptors, but labels only D_3_ receptors in the absence of divalent cations. The pharmacological profile of the D_5_ receptor is similar to, yet distinct from, that of the D_1_ receptor. The splice variants of the D_2_ receptor are commonly termed D_2S_ and D_2L_ (short and long). The *DRD4* gene encoding the D_4_ receptor is highly polymorphic in humans, with allelic variations of the protein from amino acid 387 to 515.

### Further Reading

Beaulieu JM *et al*. (2015) Dopamine receptors ‐ IUPHAR Review 13. *Br. J. Pharmacol.* **172**: 1‐23 [PMID:25671228]

Beaulieu JM *et al*. (2011) The physiology, signaling, and pharmacology of dopamine receptors. *Pharmacol. Rev.* **63**: 182‐217 [PMID:21303898]

Cumming P. (2011) Absolute abundances and affinity states of dopamine receptors in mammalian brain: A review. *Synapse* **65**: 892‐909 [PMID:21308799]

Maggio R *et al*. (2010) Dopamine D2‐D3 receptor heteromers: pharmacological properties and therapeutic significance. *Curr Opin Pharmacol* **10**: 100‐7 [PMID:19896900]

Ptácek R *et al*. (2011) Dopamine D4 receptor gene DRD4 and its association with psychiatric disorders. *Med. Sci. Monit.* **17**: RA215‐20 [PMID:21873960]

Schwartz J‐C *et al.*. (1998) Dopamine Receptors. *In The IUPHAR Compendium of Receptor Characterization and Classification* Edited by Girdlestone D: IUPHAR Media: 141‐151

Undieh AS. (2010) Pharmacology of signaling induced by dopamine D(1)‐like receptor activation. *Pharmacol. Ther.* **128**: 37‐60 [PMID:20547182]

## Endothelin receptors

### Overview

Endothelin receptors (**nomenclature as agreed by the NC‐IUPHAR Subcommittee on Endothelin Receptors [** **395** **]**) are activated by the endogenous 21 amino‐acid peptides endothelins 1‐3 (endothelin‐1(*EDN1*, P05305), endothelin‐2(*EDN2*, P20800) and endothelin‐3(*EDN3*, P14138)).

**Table:** 

Nomenclature	ET_A_ receptor	ET_B_ receptor
HGNC, UniProt	*EDNRA*, P25101	*EDNRB*, P24530
Family selective agonists	endothelin‐1 (*EDN1*, P05305) = endothelin‐2 (*EDN2*, P20800) >endothelin‐3 (*EDN3*, P14138) [1178]	endothelin‐1 (*EDN1*, P05305) = endothelin‐2 (*EDN2*, P20800), endothelin‐3 (*EDN3*, P14138)
Selective agonists	–	sarafotoxin S6c (p*K*_d_ 8.8–9.8) [1016, 1616], BQ 3020 (p*K*_i_ 9.7) [1576], [Ala^1,3,11,15^]ET‐1 (p*K*_d_ 8.7–9.2) [1300], IRL 1620 (p*K*_i_ 8.7) [1991]
(Sub)family‐selective antagonists	SB209670 (p*K*_B_ 9.4) [474] – Rat, TAK 044 (pA_2_ 8.4) [1993] – Rat, bosentan (pA_2_ 7.2) [354] – Rat	SB209670 (p*K*_B_ 9.4) [474] – Rat, TAK 044 (pA_2_ 8.4) [1993] – Rat, bosentan (p*K*_i_ 7.1) [1349]
Selective antagonists	atrasentan (pA_2_ 9.2–10.5) [1446], PD‐156707 (p*K*_d_ 9–9.8) [1180], macitentan (pIC_50_ 9.3) [174], sitaxsentan (pA_2_ 8) [2047], FR139317 (Inverse agonist) (pIC_50_ 7.3–7.9) [1178], ambrisentan (pIC_50_ 7.7) [175], BQ123 (pA_2_ 6.9–7.4) [1178], avosentan (pIC_50_ 7.3) [210], ambrisentan (pA_2_ 7.1) [175]	A192621 (p*K*_d_ 8.1) [2145], BQ788 (p*K*_d_ 7.9–8) [1616], IRL 2500 (p*K*_d_ 7.2) [1616], Ro 46‐8443 (pIC_50_ 7.2) [209]
Labelled ligands	[^125^I]PD164333 (Antagonist) (p*K*_d_ 9.6–9.8) [398], [^3^H]S0139 (Antagonist) (p*K*_d_ 9.2), [^125^I]PD151242 (Antagonist) (p*K*_d_ 9–9.1) [399], [^3^H]BQ123 (Antagonist) (p*K*_d_ 8.5) [817]	[^125^I]IRL1620 (Agonist) (p*K*_d_ 9.9–10.1) [1362], [^125^I]BQ3020 (Agonist) (p*K*_d_ 8.3–10) [702, 1300, 1495], [^125^I][Ala^1,3,11,15^]ET‐1 (Agonist) (p*K*_d_ 9.7) [1300]

### Comments

Splice variants of the ET_A_ receptor have been identified in rat pituitary cells; one of these, ET_A_R‐C13, appeared to show loss of function with comparable plasma membrane expression to wild type receptor [713]. Subtypes of the ET_B_ receptor have been proposed, although gene disruption studies in mice suggest that only a single gene product exists [1295].

### Further Reading

Clozel M *et al*. (2013) Endothelin receptor antagonists. *Handb Exp Pharmacol* **218**: 199‐227 [PMID:24092342]

Davenport AP. (2002) International Union of Pharmacology. XXIX. Update on endothelin receptornomenclature. *Pharmacol. Rev.* **54**: 219‐226 [PMID:12037137]

Dhaun N *et al*. (2012) Endothelin‐1 and the kidney–beyond BP. *Br. J. Pharmacol.* **167**: 720‐31 [PMID:22670597]

Kohan DE *et al*. (2012) Clinical trials with endothelin receptor antagonists: what went wrong and where can we improve? *Life Sci.* **91**: 528‐39 [PMID:22967485]

Kohan DE *et al*. (2011) Regulation of blood pressure and salt homeostasis by endothelin. *Physiol. Rev.* **91**: 1‐77 [PMID:21248162]

Ling L *et al*. (2013) Endothelin‐2, the forgotten isoform: emerging role in the cardiovascular system, ovarian development, immunology and cancer. *Br. J. Pharmacol.* **168**: 283‐95 [PMID:22118774]

Maguire JJ *et al*. (2014) Endothelin@25 ‐ new agonists, antagonists, inhibitors and emerging research frontiers: IUPHAR Review 12. *Br. J. Pharmacol.* **171**: 5555‐72 [PMID:25131455]

Maguire JJ *et al*. (2015) Endothelin Receptors and Their Antagonists. *Semin. Nephrol.* **35**: 125‐136 [PMID:25966344]

Said N *et al*. (2012) Permissive role of endothelin receptors in tumor metastasis. *Life Sci.* **91**: 522‐7 [PMID:22846215]

Speed JS *et al*. (2013) Endothelin, kidney disease, and hypertension. *Hypertension* **61**: 1142‐5 [PMID:23608655]

## G protein‐coupled estrogen receptor

### Overview

The G protein‐coupled estrogen receptor (GPER, **nomenclature as agreed by the NC‐IUPHAR Subcommittee on the G protein‐coupled estrogen receptor [** **1536** **]**) was identified following observations of estrogen‐evoked cyclic AMP signalling in breast cancer cells [61], which mirrored the differential expression of an orphan 7‐transmembrane receptor GPR30 [265]. There are observations of both cell‐surface and intracellular expression of the GPER receptor [1573, 1877].

**Table:** 

Nomenclature	GPER
HGNC, UniProt	*GPER1*, Q99527
Selective agonists	G1 (p*K*_i_ 8) [176]
Selective antagonists	G36 (pIC_50_ 6.8–6.9) [414], G15 (pIC_50_ 6.7) [413]
Labelled ligands	[^3^H]17*β*‐estradiol (Agonist) (p*K*_d_ 8.5–8.6) [1877]

### Comments

Antagonists at the nuclear estrogen receptor, such as fulvestrant and tamoxifen[515], as well as the flavonoid ‘phytoestrogens’ genistein and quercetin[1177], are agonists at GPER receptors.

### Further Reading

Barton M *et al*. (2015) Emerging roles of GPER in diabetes and atherosclerosis. *Trends Endocrinol. Metab.* **26**: 185‐92 [PMID:25767029]

Han G *et al*. (2013) GPER: a novel target for non‐genomic estrogen action in the cardiovascular system. *Pharmacol. Res.* **71**: 53‐60 [PMID:23466742]

Lappano R *et al*. (2014) GPER Function in Breast Cancer: An Overview. *Front Endocrinol (Lausanne)* **5**: 66 [PMID:24834064]

Prossnitz ER *et al*. (2015) International Union of Basic and Clinical Pharmacology. XCVII. G Protein‐Coupled Estrogen Receptor and Its Pharmacologic Modulators. *Pharmacol. Rev.* **67**: 505‐40 [PMID:26023144]

Prossnitz ER *et al*. (2014) Estrogen biology: new insights into GPER function and clinical opportunities. *Mol. Cell. Endocrinol.* **389**: 71‐83 [PMID:24530924]

## Formylpeptide receptors

### Overview

The formylpeptide receptors (**nomenclature agreed by the NC‐IUPHAR Subcommittee on the formyl peptide receptor family [** **2092** **]**) respond to exogenous ligands such as the bacterial product fMet‐Leu‐Phe (fMLP) and endogenous ligands such as annexin I(*ANXA1*, P04083) , cathepsin G(*CTSG*, P08311), amyloid *β*42, serum amyloid A and spinorphin, derived from *β*‐haemoglobin(*HBB*, P68871).

**Table:** 

Nomenclature	FPR1	FPR2/ALX	FPR3
HGNC, UniProt	*FPR1*, P21462	*FPR2*, P25090	*FPR3*, P25089
Rank order of potency	fMet‐Leu‐Phe>cathepsin G (*CTSG*, P08311) >annexin I (*ANXA1*, P04083) [1058, 1821]	LXA_4_=aspirin triggered lipoxin A4=ATLa2>LTC_4_=LTD_4_≫15‐deoxy‐LXA4≫fMet‐Leu‐Phe [352, 519, 521, 651, 1846]	–
Endogenous agonists	–	LXA_4_ (Selective) (pEC_50_∼12) [1006], resolvin D1 (Selective) (pEC_50_∼11.9) [1006], aspirin triggered lipoxin A4 (Selective)	F2L (*HEBP1*, Q9NRV9) (Selective) (pEC_50_ 8–8.2) [1274]
Agonists	fMet‐Leu‐Phe (pEC_50_ 10.1–10.2) [546, 1734]	–	–
Selective agonists	–	ATLa2 [662]	–
Endogenous antagonists	spinorphin (Selective) (pIC_50_ 4.3) [1099, 1348]	–	–
Antagonists	t‐Boc‐FLFLF (p*K*_i_ 6–6.5) [2008]	–	–
Selective antagonists	cyclosporin H (p*K*_i_ 6.1–7.1) [2008, 2078]	–	–
Labelled ligands	[^3^H]fMet‐Leu‐Phe (Agonist) (p*K*_d_ 7.6–9.3) [990]	[^3^H]LXA_4_ (Agonist) (p*K*_d_ 9.2–9.3) [519, 520]	–
Comments	A FITC‐conjugated fMLP analogue has been used for binding to the mouse recombinant receptor [724].	The agonist activity of the lipid mediators described has been questioned [697, 1513], which may derive from batch‐to‐batch differences, partial agonism or biased agonism. Recent results from Cooray *et al.* (2013) [365] have addressed this issue and the role of homodimers and heterodimers in the intracellular signaling .	–

### Comments

Note that the data for FPR2/ALX are also reproduced on the leukotriene receptor page.

### Further Reading

Dorward DA *et al*. (2015) The Role of Formylated Peptides and Formyl Peptide Receptor 1 in Governing Neutrophil Function during Acute Inflammation. *Am. J. Pathol.* **185**: 1172‐1184 [PMID:25791526]

Dufton N *et al*. (2010) Therapeutic anti‐inflammatory potential of formyl‐peptide receptor agonists. *Pharmacol. Ther.* **127**: 175‐88 [PMID:20546777]

Liu M *et al*. (2012) G protein‐coupled receptor FPR1 as a pharmacologic target in inflammation and human glioblastoma. *Int. Immunopharmacol.* **14**: 283‐8 [PMID:22863814]

Rabiet MJ *et al*. (2011) N‐formyl peptide receptor 3 (FPR3) departs from the homologous FPR2/ALX receptor with regard to the major processes governing chemoattractant receptor regulation, expression at the cell surface, and phosphorylation. *J. Biol. Chem.* **286**: 26718‐31 [PMID:21543323]

Yazid S *et al*. (2012) Anti‐inflammatory drugs, eicosanoids and the annexin A1/FPR2 anti‐inflammatory system. *Prostaglandins Other Lipid Mediat.* **98**: 94‐100 [PMID:22123264]

Ye RD *et al*. (2009) International Union of Basic and Clinical Pharmacology. LXXIII. Nomenclature for the formyl peptide receptor (FPR) family. *Pharmacol. Rev.* **61**: 119‐61 [PMID:19498085]

## Free fatty acid receptors

### Overview

Free fatty acid receptors (FFA, **nomenclature as agreed by the NC‐IUPHAR Subcommittee on free fatty acid receptors [** **396** **,** **1803** **]**) are activated by free fatty acids. Long‐chain saturated and unsaturated fatty acids (C14.0 (myristic acid), C16:0 (palmitic acid), C18:1 (oleic acid), C18:2 (linoleic acid), C18:3, (*α*‐linolenic acid), C20:4 (arachidonic acid), C20:5,n‐3 (EPA), C22:6,n‐3 (docosahexaenoic acid)) activate FFA1 [218, 833, 998] and FFA4 receptors [757, 812, 1427], while short chain fatty acids (C2 (acetic acid), C3 (propanoic acid), C4 (butyric acid) and C5 (pentanoic acid)) activate FFA2 [226, 1057, 1399] and FFA3 [226, 1057] receptors. In addition, thiazolidinedione PPAR*γ* agonists such as rosiglitazone activate FFA1 (pEC_50_ 5.2; [999, 1768, 1802]) and small molecule allosteric modulators, such as 4‐CMTB, have recently been characterised for FFA2 [801, 1070, 1769].

**Table:** 

Nomenclature	FFA1 receptor	FFA2 receptor	FFA3 receptor	FFA4 receptor	*GPR42*
HGNC, UniProt	*FFAR1*, O14842	*FFAR2*, O15552	*FFAR3*, O14843	*FFAR4*, Q5NUL3	*GPR42*, O15529
Endogenous agonists	docosahexaenoic acid (pEC_50_ 5.4–6) [218, 833]	–	–	*α*‐linolenic acid (pEC_50_ 5.5) [1727]	–
Agonists	fasiglifam (pEC_50_ 7.1) [893, 1791, 1909]	–	–	–	–
(Sub)family‐selective agonists	*α*‐linolenic acid (pEC_50_ 4.6–5.7) [218, 833, 998], oleic acid (pEC_50_ 3.9–5.7) [218, 833, 998], myristic acid (pEC_50_ 4.5–5.1) [218, 833, 998]	propanoic acid (pEC_50_ 3–4.9) [226, 1057, 1399, 1670], acetic acid (pEC_50_ 3.1–4.6) [226, 1057, 1399, 1670], butyric acid (pEC_50_ 2.9–4.6) [226, 1057, 1399, 1670], *trans*‐2‐methylcrotonic acid (pEC_50_ 3.8) [1670], 1‐methylcyclopropanecarboxylic acid (pEC_50_ 2.6) [1670]	propanoic acid (pEC_50_ 3.9–5.7) [226, 1057, 1670, 2063], butyric acid (pEC_50_ 3.8–4.9) [226, 1057, 1670, 2063], 1‐methylcyclopropanecarboxylic acid (pEC_50_ 3.9) [1670], acetic acid (pEC_50_ 2.8–3.9) [226, 1057, 1670, 2063]	myristic acid (pEC_50_ 5.2) [1996], oleic acid (pEC_50_ 4.7) [1996]	–
Selective agonists	AMG‐837 (pEC_50_ 8.5) [1110], TUG‐770 (pEC_50_ 8.2) [332], GW9508 (pEC_50_ 7.3) [217], linoleic acid (pEC_50_ 4.4–5.7) [218, 833, 998]	compound 1 [PMID: 23589301] (pEC_50_ 7.1) [800] – Rat, (S)‐4‐CMTB (pEC_50_ 6.4) [801, 1070]	–	compound A [PMID 24997608] (pEC_50_ 7.6) [1428], TUG‐891 (pEC_50_ 7) [1727] – Unknown, NCG21 (pEC_50_ 5.9) [1829]	–
Selective antagonists	GW1100 (pIC_50_ 6) [217]	GLPG0974 (pIC_50_ 8.1) [1512], CATPB (pIC_50_ 6.5) [801]	–	–	–
Comments	Antagonist GW1100 has been shown to reduce [^35^S]GTP*γ*S binding in *FFAR1*‐expressing cells [1802]. GW1100 is also an oxytocin receptor antagonist [217]. TUG‐770 and GW9508 are both approximately 100 fold selective for FFA1 over FFA4 [217, 332]. AMG‐837 and the related analogue AM6331 have been suggested to have an allosteric mechanism of action at FFA1, with respect to the orthosteric fatty acid binding site [1110, 2064].	–	Beta‐hydroxybutyrate has been reported to antagonise FFA3 responses to short chain fatty acids [951]. A range of FFA3 selective molecules with agonist and antagonist properties, but which bind at sites distinct from the short chain fatty acid binding site, have recently been described [799].	compound A [PMID 24997608] exhibits more than 1000 fold selectivity [1428], and TUG‐891 50‐1000 fold selectivity for FFA4 over FFA1 [1727], dependent on the assay. NGC21 exhibits approximately 15 fold selectivity for FFA4 over FFA1 [1820].	–

### Comments

Short (361 amino acids) and long (377 amino acids) splice variants of human FFA4 have been reported [1318], which differ by a 16 amino acid insertion in intracellular loop 3, and exhibit differences in intracellular signalling properties in recombinant systems [1996]. The long FFA4 splice variant has not been identified in other primates or rodents to date [757, 1318].

*GPR42* was originally described as a pseudogene within the family (ENSFM00250000002583), but the recent discovery of several polymorphisms suggests that some versions of GPR42 may be functional [1101]. *GPR84* is a structurally‐unrelated G protein‐coupled receptor which has been found to respond to medium chain fatty acids [1981].

### Further Reading

Mancini AD *et al*. (2013) The fatty acid receptor FFA1/GPR40 a decade later: how much do we know? *Trends Endocrinol. Metab.* **24**: 398‐407 [PMID:23631851]

Maslowski KM *et al*. (2011) Diet, gut microbiota and immune responses. *Nat. Immunol.* **12**: 5‐9 [PMID:21169997]

Milligan G *et al*. (2009) Agonism and allosterism: the pharmacology of the free fatty acid receptors FFA2 and FFA3. *Br. J. Pharmacol.* **158**: 146‐53 [PMID:19719777]

Reimann F *et al*. (2012) G‐protein‐coupled receptors in intestinal chemosensation. *Cell Metab.* **15**: 421‐31 [PMID:22482725]

Stoddart LA *et al*. (2008) International Union of Pharmacology. LXXI. Free fatty acid receptors FFA1, ‐2, and ‐3: pharmacology and pathophysiological functions. *Pharmacol. Rev.* **60**: 405‐17 [PMID:19047536]

Talukdar S *et al*. (2011) Targeting GPR120 and other fatty acid‐sensing GPCRs ameliorates insulin resistance and inflammatory diseases. *Trends Pharmacol. Sci.* **32**: 543‐50 [PMID:21663979]

Watterson KR *et al*. (2014) Treatment of type 2 diabetes by free Fatty Acid receptor agonists. *Front Endocrinol (Lausanne)* **5**: 137 [PMID:25221541]

## GABA_B_ receptors

### Overview

Functional GABA_B_ receptors (**nomenclature as agreed by the NC‐IUPHAR Subcommittee on GABA_B_ receptors [** **194** **,** **1507** **]**) are formed from the heterodimerization of two similar 7TM subunits termed GABA_B1_ and GABA_B2_[194, 478, 1506, 1507, 1926]. GABA_B_ receptors are widespread in the CNS and regulate both pre‐ and postsynaptic activity. The GABA_B1_ subunit, when expressed alone, binds both antagonists and agonists, but the affinity of the latter is generally 10‐100‐fold less than for the native receptor. The GABA_B1_ subunit when expressed alone is not transported to the cell membrane and is non‐functional. However, Richer *et al.*. (2008) report that GABA_B1_ alone can control ERK/MAPK pathway activity [1585]. Co‐expression of GABA_B1_ and GABA_B2_ subunits allows transport of GABA_B1_ to the cell surface and generates a functional receptor that can couple to signal transduction pathways such as high‐voltage‐activated Ca^2+^ channels (Ca_v_2.1, Ca_v_2.2), or inwardly rectifying potassium channels (Kir3) [144, 194, 195]. The GABA_B2_subunit also determines the rate of internalisation of the dimeric GABA_B_ receptor [693]. The GABA_B1_ subunit harbours the GABA (orthosteric)‐binding site within an extracellular domain (ECD) venus flytrap module (VTM), whereas the GABA_B2_ subunit mediates G protein‐coupled signalling [194, 591, 592, 1506]. The two subunits interact by direct allosteric coupling [1313], such that GABA_B2_ increases the affinity of GABA_B1_ for agonists and reciprocally GABA_B1_ facilitates the coupling of GABA_B2_ to G proteins [591, 1013, 1506]. GABA_B1_ and GABA_B2_ subunits assemble in a 1:1 stoichiometry by means of a coiled‐coil interaction between *α*‐helices within their carboxy‐termini that masks an endoplasmic reticulum retention motif (RXRR) within the GABA_B1_ subunit but other domains of the proteins also contribute to their heteromerization [144, 243, 1506]. Recent evidence indicates that higher order assemblies of GABA_B_receptor comprising dimers of heterodimers occur in recombinant expression systems and *in vivo* and that such complexes exhibit negative functional cooperativity between heterodimers [361, 1505]. Adding further complexity, KCTD (potassium channel tetramerization proteins) 8, 12, 12b and 16 associate as tetramers with the carboxy terminus of the GABA_B2_ subunit to impart altered signalling kinetics and agonist potency to the receptor complex [102, 1680, 1914] and reviewed by [1508]. Four isoforms of the human GABA_B1_ subunit have been cloned. The predominant GABA_B1(a)_ and GABA_B1(b)_ isoforms, which are most prevalent in neonatal and adult brain tissue respectively, differ in their ECD sequences as a result of the use of alternative transcription initiation sites. GABA_B1(a)_‐containing heterodimers localise to distal axons and mediate inhibition of glutamate release in the CA3‐CA1 terminals, and GABA release onto the layer 5 pyramidal neurons, whereas GABA_B1(b)_‐containing receptors occur within dendritic spines and mediate slow postsynaptic inhibition [1541, 1955]. Isoforms generated by alternative splicing are GABA_B1(c)_ that differs in the ECD, and GABA_B1(e)_, which is a truncated protein that can heterodimerize with the GABA_B2_ subunit but does not constitute a functional receptor. Only the 1a and 1b variants are identified as components of native receptors [194]. Additional GABA_B1_ subunit isoforms have been described in rodents and humans [1065] and reviewed by [144].

**Table:** 

Nomenclature	GABA_B_ receptor
Subunits	kcdt12b (Accessory protein), KCTD16 (Accessory protein), KCTD12 (Accessory protein), GABA_B2_, GABA_B1_, KCTD8 (Accessory protein)
Agonists	CGP 44532 (pIC_50_ 8.6) [551] – Rat, (‐)‐baclofen (pIC_50_ 8.5) [551] – Rat, 3‐APPA (p*K*_i_ 5.2–7.2) [762], baclofen (p*K*_i_ 4.3–6.2) [762, 2041], 3‐APMPA (p*K*_i_ 5.1) [2041]
Antagonists	CGP 62349 (p*K*_i_ 8.5–8.9) [762, 2041], CGP 55845 (p*K*_i_ 7.8) [2041], SCH 50911 (p*K*_i_ 5.5–6) [762, 2041], CGP 35348 (p*K*_i_ 4.4) [2041], 2‐hydroxy‐saclofen (pIC_50_ 4.1) [914] – Rat
Labelled ligands	[^3^H]CGP 54626 (Antagonist) (p*K*_i_ 9.1) [879] – Rat, [^3^H]CGP 62349 (Antagonist) (p*K*_d_ 9.1) [922] – Rat, [^125^I]CGP 64213 (Antagonist) (p*K*_d_ 9) [563] – Rat, [^125^I]CGP 71872 (Antagonist) (p*K*_d_ 9) [914] – Rat, [^3^H](R)‐(‐)‐baclofen (Agonist)

### Subunits

**Table:** 

Nomenclature	GABA_B1_	GABA_B2_
HGNC, UniProt	*GABBR1*, Q9UBS5	*GABBR2*, O75899

### Comments

Potencies of agonists and antagonists listed in the table, quantified as IC_50_ values for the inhibition of [^3^H]CGP27492 binding to rat cerebral cortex membranes, are from [194, 550, 551]. Radioligand *K*_D_ values relate to binding to rat brain membranes. CGP 71872 is a photoaffinity ligand for the GABA_B1_ subunit [122]. CGP27492 (3‐APPA), CGP35024 (3‐APMPA) and CGP 44532 act as antagonists at human GABA_A_ *ρ*1 receptors, with potencies in the low micromolar range [550]. In addition to the ligands listed in the table, Ca^2+^ binds to the VTM of the GABA_B1_ subunit to act as a positive allosteric modulator of GABA[563]. In cerebellar Purkinje neurones, the interaction of Ca^2+^ with the GABA_B_ receptor enhances the activity of mGlu_1_, through functional cross‐talk involving G‐protein G*β* *γ* subunits [1590, 1837]. Synthetic positive allosteric modulators with low, or no, intrinsic activity include CGP7930, GS39783, BHF‐177 and (+)‐BHFF[9, 144, 150, 550]. The site of action of CGP7930 and GS39783 appears to be on the heptahelical domain of the GABA_B2_ subunit [455, 1506]. In the presence of CGP7930 or GS39783, CGP 35348 and 2‐hydroxy‐saclofen behave as partial agonists [550]. A negative allosteric modulator of GABA_B_ activity has been reported [302]. Knock‐out of the GABA_B1_ subunit in C57B mice causes the development of severe tonic‐clonic convulsions that prove fatal within a month of birth, whereas ${\text{GABA}}_{\text{B1}}^{\;\;-\text{/}-}$ BALB/c mice, although also displaying spontaneous epileptiform activity, are viable. The phenotype of the latter animals additionally includes hyperalgesia, hyperlocomotion (in a novel, but not familiar, environment), hyperdopaminergia, memory impairment and behaviours indicative of anxiety [482, 1932]. A similar phenotype has been found for ${\text{GABA}}_{\text{B2}}^{\;\;-\text{/}-}$ BALB/c mice [582].

### Further Reading

Bettler B *et al*. (2004) Molecular structure and physiological functions of GABA(B) receptors. *Physiol. Rev.* **84**: 835‐67 [PMID:15269338]

Bowery NG *et al*. (2002) International Union of Pharmacology. XXXIII. Mammalian gamma‐aminobutyricacid(B) receptors: structure and function. *Pharmacol Rev.* **54**: 247‐264 [PMID:12037141]

Froestl W. (2011) An historical perspective on GABAergic drugs. *Future Med Chem* **3**: 163‐75 [PMID:21428811]

Gassmann M *et al*. (2012) Regulation of neuronal GABA(B) receptor functions by subunit composition. *Nat. Rev. Neurosci.* **13**: 380‐94 [PMID:22595784]

Marshall FH. (2005) Is the GABA B heterodimer a good drug target? *J. Mol. Neurosci.* **26**: 169‐76 [PMID:16012190]

Rondard P *et al*. (2011) The complexity of their activation mechanism opens new possibilities for the modulation of mGlu and GABAB class C G protein‐coupled receptors. *Neuropharmacology* **60**: 82‐92 [PMID:20713070]

## Galanin receptors

### Overview

Galanin receptors (**provisional nomenclature as recommended by NC‐IUPHAR[** **530** **]**) are activated by the endogenous peptides galanin(*GAL*, P22466) and galanin‐like peptide(*GALP*, Q9UBC7). Human galanin(*GAL*, P22466) is a 30 amino‐acid non‐amidated peptide [499]; in other species, it is 29 amino acids long and C‐terminally amidated. Amino acids 1‐14 of galanin are highly conserved in mammals, birds, reptiles, amphibia and fish. Shorter peptide species (*e.g*. human galanin‐1‐19 [139] and porcine galanin‐5‐29 [1740]) and N‐terminally extended forms (*e.g.* N‐terminally seven and nine residue elongated forms of porcine galanin [140, 1740]) have been reported.

**Table:** 

Nomenclature	GAL_1_ receptor	GAL_2_ receptor	GAL_3_ receptor
HGNC, UniProt	*GALR1*, P47211	*GALR2*, O43603	*GALR3*, O60755
Rank order of potency	galanin (*GAL*, P22466) >galanin‐like peptide (*GALP*, Q9UBC7) [1433]	galanin‐like peptide (*GALP*, Q9UBC7) ≥galanin (*GAL*, P22466) [1433]	galanin‐like peptide (*GALP*, Q9UBC7) >galanin (*GAL*, P22466) [1039]
Agonists	–	galanin(2‐29) (rat/mouse) (p*K*_i_ 7.2–8.7) [1457, 1982, 1983, 1984] – Rat	–
Selective agonists	–	[D‐Trp^2^]galanin‐(1‐29) (p*K*_i_ 8.1) [1765] – Rat	–
Selective antagonists	2,3‐dihydro‐1,4‐dithiin‐1,1,4,4‐tetroxide (pIC_50_ 5.6) [1688]	M871 (p*K*_i_ 7.9) [1780]	SNAP 398299 (p*K*_i_ 8.3) [987, 988, 1833], SNAP 37889 (p*K*_i_ 7.8–7.8) [987, 988, 1833]
Selective allosteric modulators	–	CYM2503 (Positive) (pEC_50_ 9.2) [1147] – Rat	–
Labelled ligands	[^125^I][Tyr^26^]galanin (human) (Agonist) (p*K*_d_ 10.3) [525], [^125^I][Tyr^26^]galanin (human) (Agonist) (p*K*_d_ 7.8) [525]	[^125^I][Tyr^26^]galanin (human) (Agonist) (p*K*_d_ 9.2) [1983] – Rat	[^125^I][Tyr^26^]galanin (pig) (Agonist) (p*K*_d_ 8.6) [187, 1766]
Comments	–	The CYM2503 PAM potentiates the anticonvulsant activity of endogenous galanin in mouse seizure models [1147].	–

### Comments

galanin‐(1‐11) is a high‐affinity agonist at GAL_1_/GAL_2_(p*K*_i_ 9), and galanin(2‐11) is selective for GAL_2_ and GAL_3_ compared with GAL_1_[1146]. [^125^I]‐[Tyr^26^]galanin binds to all three subtypes with *K*_d_ values generally reported to range from 0.05 to 1 nM, depending on the assay conditions used [525, 1752, 1765, 1766, 1983]. Porcine galanin‐(3‐29) does not bind to cloned GAL_1_, GAL_2_ or GAL_3_ receptors, but a receptor that is functionally activated by porcine galanin‐(3‐29) has been reported in pituitary and gastric smooth muscle cells [658, 2054]. Additional galanin receptor subtypes are also suggested from studies with chimeric peptides (*e.g*. M15, M35 and M40), which act as antagonists in functional assays in the cardiovascular system [1924], spinal cord [2024], locus coeruleus, hippocampus [100] and hypothalamus [101, 1078], but exhibit agonist activity at some peripheral sites [101, 658]. The chimeric peptides M15, M32, M35, M40 and C7 are agonists at GAL_1_ receptors expressed endogenously in Bowes human melanoma cells [1433], and at heterologously expressed recombinant GAL_1_, GAL_2_ and GAL_3_ receptors [525, 1765, 1766]. Recent studies have described the synthesis of a series of novel, systemically‐active, galanin analogues, with modest preferential binding at the GAL_2_ receptor. Specific chemical modifications to the galanin backbone increased brain levels of these peptides after *i.v.* injection and several of these peptides exerted a potent antidepressant‐like effect in mouse models of depression [1623].

### Further Reading

Foord SM *et al*. (2005) International Union of Pharmacology. XLVI. G protein‐coupled receptor list. *Pharmacol Rev* **57**: 279‐288 [PMID:15914470]

Lang R *et al*. (2015) Physiology, signaling, and pharmacology of galanin peptides and receptors: three decades of emerging diversity. *Pharmacol. Rev.* **67**: 118‐75 [PMID:25428932]

Lang R *et al*. (2011) The galanin peptide family in inflammation. *Neuropeptides* **45**: 1‐8 [PMID:21087790]

Lawrence C *et al*. (2011) Galanin‐like peptide (GALP) is a hypothalamic regulator of energy homeostasis and reproduction. *Front Neuroendocrinol* **32**: 1‐9 [PMID:20558195]

Webling KE *et al*. (2012) Galanin receptors and ligands. *Front Endocrinol (Lausanne)* **3**: 146 [PMID:23233848]

## Ghrelin receptor

### Overview

The ghrelin receptor (**nomenclature as agreed by the NC‐IUPHAR Subcommittee for the Ghrelin receptor [** **397** **]**) is activated by a 28 amino‐acid peptide originally isolated from rat stomach, where it is cleaved from a 117 amino‐acid precursor (*GHRL*, Q9UBU3). The human gene encoding the precursor peptide has 83% sequence homology to rat prepro‐ghrelin, although the mature peptides from rat and human differ by only two amino acids [1222]. Alternative splicing results in the formation of a second peptide, [des‐Gln^14^]ghrelin(*GHRL*, Q9UBU3) with equipotent biological activity [783]. A unique post‐translational modification (octanoylation of Ser^3^, catalysed by ghrelin *O*‐acyltransferase (*MBOAT4*, Q96T53) [2082] occurs in both peptides, essential for full activity in binding to ghrelin receptors in the hypothalamus and pituitary, and for the release of growth hormone from the pituitary [983]. Structure activity studies showed the first five N‐terminal amino acids to be the minimum required for binding [116], and receptor mutagenesis has indicated overlap of the ghrelin binding site with those for small molecule agonists and allosteric modulators of ghrelin(*GHRL*, Q9UBU3) function [776]. In cell systems, the ghrelin receptor is constitutively active [777], but this is abolished by a naturally occurring mutation (A204E) that results in decreased cell surface receptor expression and is associated with familial short stature [1458].

**Table:** 

Nomenclature	ghrelin receptor
HGNC, UniProt	*GHSR*, Q92847
Rank order of potency	ghrelin (*GHRL*, Q9UBU3) = [des‐Gln^14^]ghrelin (*GHRL*, Q9UBU3) [115, 1222]
Selective antagonists	GSK1614343 (pIC_50_ 8.4) [1624], GSK1614343 (p*K*_B_ 8) [1487] – Rat
Labelled ligands	[^125^I][His^9^]ghrelin (human) (Agonist) (p*K*_d_ 9.4) [912], [^125^I][Tyr^4^]ghrelin (human) (Agonist) (p*K*_d_ 9.4) [1339]

### Comments

[des‐octanoyl]ghrelin(*GHRL*, Q9UBU3) has been shown to bind (as [^125^I]Tyr^4^‐des‐octanoyl‐ghrelin) and have effects in the cardiovascular system [115], which raises the possible existence of different receptor subtypes in peripheral tissues and the central nervous system. A potent inverse agonist has been identified ([D‐Arg^1^,D‐Phe^5^,D‐Trp^7,9^,Leu^11^]substance P, p*D*_2_ 8.3; [774]). Ulimorelin, described as a ghrelin receptor agonist (p*K*_i_ 7.8 and p*D*_2_ 7.5 at human recombinant ghrelin receptors), has been shown to stimulate ghrelin receptor mediated food intake and gastric emptying but not elicit release of growth hormone, or modify ghrelin stimulated growth hormone release, thus pharmacologically discriminating the orexigenic and gastrointestinal actions of ghrelin(*GHRL*, Q9UBU3) from the release of growth hormone [538]. A number of selective antagonists have been reported, including peptidomimetic [1338] and non‐peptide small molecules including GSK1614343[1487, 1624].

### Further Reading

Andrews ZB. (2011) The extra‐hypothalamic actions of ghrelin on neuronal function. *Trends Neurosci.* **34**: 31‐40 [PMID:21035199]

Angelidis G *et al*. (2010) Current and potential roles of ghrelin in clinical practice. *J. Endocrinol. Invest.* **33**: 823‐38 [PMID:21293171]

Briggs DI *et al*. (2011) Metabolic status regulates ghrelin function on energy homeostasis. *Neuroendocrinology* **93**: 48‐57 [PMID:21124019]

Callaghan B *et al*. (2014) Novel and conventional receptors for ghrelin, desacyl‐ghrelin, and pharmacologically related compounds. *Pharmacol. Rev.* **66**: 984‐1001 [PMID:25107984]

Davenport AP *et al*. (2005) International Union of Pharmacology. LVI. Ghrelin receptor nomenclature, distribution, and function. *Pharmacol. Rev.* **57**: 541‐6 [PMID:16382107]

De Smet B *et al*. (2009) Motilin and ghrelin as prokinetic drug targets. *Pharmacol. Ther.* **123**: 207‐23 [PMID:19427331]

De Vriese C *et al*. (2007) Influence of ghrelin on food intake and energy homeostasis. *Curr Opin Clin Nutr Metab Care* **10**: 615‐9 [PMID:17693746]

Dezaki K *et al*. (2008) Ghrelin is a physiological regulator of insulin release in pancreatic islets and glucose homeostasis. *Pharmacol. Ther.* **118**: 239‐49 [PMID:18433874]

Granata R *et al*. (2010) Unraveling the role of the ghrelin gene peptides in the endocrine pancreas. *J. Mol. Endocrinol.* **45**: 107‐18 [PMID:20595321]

Nikolopoulos D *et al*. (2010) Ghrelin: a potential therapeutic target for cancer. *Regul. Pept.* **163**: 7‐17 [PMID:20382189]

## Glucagon receptor family

### Overview

The glucagon family of receptors (**nomenclature as agreed by the NC‐IUPHAR Subcommittee on the Glucagon receptor family [** **1234** **]**) are activated by the endogenous peptide (27‐44 aa) hormones glucagon(*GCG*, P01275), glucagon‐like peptide 1(*GCG*, P01275), glucagon‐like peptide 2 (*GCG*, P01275), glucose‐dependent insulinotropic polypeptide (also known as gastric inhibitory polypeptide(*GIP*, P09681)), GHRH(*GHRH*, P01286) and secretin(*SCT*, P09683). One common precursor (*GCG*) generates glucagon(*GCG*, P01275), glucagon‐like peptide 1(*GCG*, P01275) and glucagon‐like peptide 2(*GCG*, P01275) peptides [827].

**Table:** 

Nomenclature	GHRH receptor	GIP receptor	GLP‐1 receptor
HGNC, UniProt	*GHRHR*, Q02643	*GIPR*, P48546	*GLP1R*, P43220
Endogenous agonists	–	gastric inhibitory polypeptide (*GIP*, P09681) (Selective) (p*K*_d_ 8.7) [1961]	glucagon‐like peptide 1‐(7‐36) amide (*GCG*, P01275) (Selective) (p*K*_i_ 9.2) [885], glucagon‐like peptide 1‐(7‐37) (*GCG*, P01275) (Selective) [425]
Agonists	JI‐38 [255], sermorelin	–	liraglutide (pEC_50_ 10.2) [972], lixisenatide (p*K*_i_ 8.9) [2010], WB4‐24 (pA_2_ 4.9) [502]
Selective agonists	BIM28011 [379], tesamorelin	–	exendin‐4 (pIC_50_ 9.2) [1290], exendin‐4 (p*K*_i_ 8.7–9) [885], exendin‐3 (P20394) [1564]
Selective antagonists	JV‐1‐36 (p*K*_i_ 10.1–10.4) [1662, 1947, 1948] – Rat, JV‐1‐38 (p*K*_i_ 10.1) [1662, 1947, 1948] – Rat	[Pro^3^]GIP [584] – Mouse	exendin‐(9‐39) (p*K*_i_ 8.1) [885], GLP‐1‐(9‐36) (pIC_50_ 6.9) [1314] – Rat, T‐0632 (pIC_50_ 4.7) [1883]
Labelled ligands	[^125^I]GHRH (human) (Agonist) (p*K*_d_ 7.6) [192] – Rat	[^125^I]GIP (human) (Agonist) (p*K*_d_ 8.6) [562] – Rat	[^125^I]GLP‐1‐(7‐36)‐amide (Agonist) (p*K*_d_ 9.3) [885], [^125^I]exendin‐(9‐39) (Antagonist) (p*K*_d_ 8.3) [885], [^125^I]GLP‐1‐(7‐37) (human) (Agonist)

**Table:** 

Nomenclature	GLP‐2 receptor	glucagon receptor	secretin receptor
HGNC, UniProt	*GLP2R*, O95838	*GCGR*, P47871	*SCTR*, P47872
Endogenous agonists	glucagon‐like peptide 2 (*GCG*, P01275) (Selective) (pIC_50_ 8.5) [1880]	glucagon (*GCG*, P01275) (Selective) (pEC_50_ 9) [1515]	secretin (*SCT*, P09683) (Selective) (pEC_50_ 9.7) [329]
Agonists	teduglutide [1248]	–	–
Selective antagonists	–	L‐168,049 (pIC_50_ 8.4) [269], des‐His^1^‐[Glu^9^]glucagon‐NH_2_ (pA_2_ 7.2) [1928, 1929] – Rat, NNC 92‐1687 (p*K*_i_ 5) [1170], BAY27‐9955 [1496]	[(CH_2_NH)^4,5^]secretin (p*K*_i_ 5.3) [668]
Labelled ligands	–	[^125^I]glucagon (human, mouse, rat) (Agonist)	[^125^I](Tyr^10^)secretin‐27 (rat) (Agonist) [1925] – Rat

### Comments

The glucagon receptor has been reported to interact with receptor activity modifying proteins (RAMPs), specifically RAMP2, in heterologous expression systems [333], although the physiological significance of this has yet to be established.

### Further Reading

Ahrén B. (2015) Glucagon–Early breakthroughs and recent discoveries. *Peptides* **67**: 74‐81 [PMID:25814364]

Campbell JE *et al*. (2013) Pharmacology, physiology, and mechanisms of incretin hormone action. *Cell Metab.* **17**: 819‐37 [PMID:23684623]

Cho YM *et al*. (2012) Targeting the glucagon receptor family for diabetes and obesity therapy. *Pharmacol. Ther.* **135**: 247‐78 [PMID:22659620]

Corazzini V *et al*. (2013) Molecular and clinical aspects of GHRH receptor mutations. *Endocr Dev* **24**: 106‐17 [PMID:23392099]

Donnelly D. (2012) The structure and function of the glucagon‐like peptide‐1 receptor and its ligands. *Br. J. Pharmacol.* **166**: 27‐41 [PMID:21950636]

Drucker DJ *et al*. (2014) Physiology and pharmacology of the enteroendocrine hormone glucagon‐like peptide‐2. *Annu. Rev. Physiol.* **76**: 561‐83 [PMID:24161075]

Jones BJ *et al*. (2012) Minireview: Glucagon in stress and energy homeostasis. *Endocrinology* **153**: 1049‐54 [PMID:22294753]

Mayo KE *et al*. (2003) International Union of Pharmacology. XXXV. The glucagon receptor family. *Pharmacol. Rev.* **55**: 167‐94 [PMID:12615957]

Miller LJ *et al*. (2013) The orthosteric agonist‐binding pocket in the prototypic class B G‐protein‐coupled secretin receptor. *Biochem. Soc. Trans.* **41**: 154‐8 [PMID:23356276]

Rowland KJ *et al*. (2011) The ~cryptic~ mechanism of action of glucagon‐like peptide‐2. *Am. J. Physiol. Gastrointest. Liver Physiol.* **301**: G1‐8 [PMID:21527727]

Trujillo JM *et al*. (2014) GLP‐1 receptor agonists for type 2 diabetes mellitus: recent developments and emerging agents. *Pharmacotherapy* **34**: 1174‐86 [PMID:25382096]

## Glycoprotein hormone receptors

### Overview

Glycoprotein hormone receptors (**provisional nomenclature [** **530** **]**) are activated by a non‐covalent heterodimeric glycoprotein made up of a common *α* chain (glycoprotein hormone common alpha subunit (*CGA*, P01215) *CGA*, P01215), with a unique *β* chain that confers the biological specificity to FSH(*CGA* *FSHB*, P01215 P01225), LH(*CGA* *LHB*, P01215 P01229), hCG(*CGA* *CGB*, P01215 P01233) or TSH(*CGA* *TSHB*, P01215 P01222). There is binding cross‐reactivity across the endogenous agonists for each of the glycoprotein hormone receptors. The deglycosylated hormones appear to exhibit reduced efficacy at these receptors [1626].

**Table:** 

Nomenclature	FSH receptor	LH receptor	TSH receptor
HGNC, UniProt	*FSHR*, P23945	*LHCGR*, P22888	*TSHR*, P16473
Endogenous agonists	–	hCG (*CGA* *CGB*, P01215 P01233) (Selective) (p*K*_d_ 9.9–11.8) [864, 1353], LH (*CGA* *LHB*, P01215 P01229) (Selective) (pIC_50_ 9.9–10.9) [864, 1353]	–
Antagonists	FSH deglycosylated *α*/*β* (p*K*_d_ 10) [527, 921]	–	–
Labelled ligands	[^125^I]FSH (human) (Agonist)	[^125^I]LH (Agonist), [^125^I]chorionic gonadotropin (human) (Agonist)	[^125^I]TSH (human) (Agonist)
Comments	Animal follitropins are less potent than the human hormone as agonists at the human FSH receptor. Gain‐ and loss‐of‐function mutations of the FSH receptor are associated with human reproductive disorders [19, 109, 650, 1900]. The rat FSH receptor also stimulates phosphoinositide turnover through an unidentified G protein [1547].	Loss‐of‐function mutations of the LH receptor are associated with Leydig cell hypoplasia and gain‐of‐function mutations are associated with male‐limited gonadotropin‐independent precocious puberty (*e.g*. [1044, 1720]) and Leydig cell tumours [1126].	Autoimmune antibodies that act as agonists of the TSH receptor are found in patients with Graves' disease (*e.g.* [1558]). Mutants of the TSH receptor exhibiting constitutive activity underlie hyperfunctioning thyroid adenomas [1464] and congenital hyperthyroidism [993]. TSH receptor loss‐of‐function mutations are associated with TSH resistance [1824].

### Further Reading

Chiamolera MI *et al*. (2009) Minireview: Thyrotropin‐releasing hormone and the thyroid hormone feedback mechanism. *Endocrinology* **150**: 1091‐6 [PMID:19179434]

George JW *et al*. (2011) Current concepts of follicle‐stimulating hormone receptor gene regulation. *Biol. Reprod.* **84**: 7‐17 [PMID:20739665]

Gershengorn MC *et al*. (2012) Update in TSH receptor agonists and antagonists. *J. Clin. Endocrinol. Metab.* **97**: 4287‐92 [PMID:23019348]

Melamed P *et al*. (2012) Gonadotrophin‐releasing hormone signalling downstream of calmodulin. *J. Neuroendocrinol.* **24**: 1463‐75 [PMID:22775470]

Menon KM *et al*. (2012) Structure, function and regulation of gonadotropin receptors ‐ a perspective. *Mol. Cell. Endocrinol.* **356**: 88‐97 [PMID:22342845]

Puett D *et al*. (2010) The luteinizing hormone receptor: insights into structure‐function relationships and hormone‐receptor‐mediated changes in gene expression in ovarian cancer cells. *Mol. Cell. Endocrinol.* **329**: 47‐55 [PMID:20444430]

## Gonadotrophin‐releasing hormone receptors

### Overview

GnRH_1_ and GnRH_2_ receptors (**provisonal nomenclature [** **530** **]**, also called Type I and Type II GnRH receptor, respectively [1284]) have been cloned from numerous species, most of which express two or three types of GnRH receptor [1283, 1284, 1741]. GnRH I (*GNRH1*, P01148) (p‐Glu‐His‐Trp‐Ser‐Tyr‐Gly‐Leu‐Arg‐Pro‐Gly‐NH2) is a hypothalamic decapeptide also known as luteinizing hormone‐releasing hormone, gonadoliberin, luliberin, gonadorelin or simply as GnRH. It is a member of a family of similar peptides found in many species [1283, 1284, 1741] including GnRH II(*GNRH2*, O43555) (pGlu‐His‐Trp‐Ser‐His‐Gly‐Trp‐Tyr‐Pro‐Gly‐NH_2_(which is also known as chicken GnRH‐II). Receptors for three forms of GnRH exist in some species but only GnRH I and GnRH II and their cognate receptors have been found in mammals [1283, 1284, 1741]. GnRH_1_ receptors are expressed primarily by pituitary gonadotrophs, and mediate central control of mammalian reproduction. They are selectively activated by GnRH I and all lack the COOH‐terminal tails found in other GPCRs. GnRH_2_ receptors do have COOH‐terminal tails and (where tested) are selective for GnRH II over GnRH I. GnRH_2_ receptors are expressed by some primates but are thought not to be expressed by humans because the human *GNRHR2* gene contains a frame shift and an internal stop codon [1325]. An alternative phylogenetic classification divides GnRH receptors into three classes and includes both GnRH I‐selective mammalian and GnRH II‐selective non‐mammalian GnRH receptors as GnRH_1_ receptors [1284]. A more recent phylogenetic classification groups vertebrate GnRH receptors into five subfamilies [2028] and highlights examples of gene loss through evolution, with humans notably retaining only one ancient gene. Although thousands of peptide analogues of GnRH I have been synthesized and several (agonists and antagonists) are used therapeutically [934], the potency of most of these peptides at GnRH_2_ receptors is unknown.

**Table:** 

Nomenclature	GnRH_1_ receptor	GnRH_2_ receptor
HGNC, UniProt	*GNRHR*, P30968	*GNRHR2*, Q96P88
Rank order of potency	GnRH I (*GNRH1*, P01148) >GnRH II (*GNRH2*, O43555) [1284]	GnRH II (*GNRH2*, O43555) >GnRH I (*GNRH1*, P01148) (Monkey) [1282]
Endogenous agonists	–	GnRH II (*GNRH2*, O43555) (pIC_50_ 9) [1282] – Monkey, GnRH I (*GNRH1*, P01148) (pIC_50_ 7.4) [1282] – Monkey
Selective agonists	triptorelin (p*K*_i_ 9.3–9.5) [112], leuprolide (p*K*_i_ 8.5–9.1) [1807], buserelin, goserelin, histrelin, nafarelin	–
Antagonists	iturelix (p*K*_i_ 9.5) [1591]	–
Selective antagonists	cetrorelix (p*K*_i_ 9.3–10) [113, 114, 1807], abarelix (p*K*_i_ 9.1–9.5) [1807], degarelix (p*K*_i_ 8.8) [1938], ganirelix	trptorelix‐1 [1183] – Monkey
Labelled ligands	[^125^I]buserelin (Agonist) (p*K*_d_ 7.4) [1024] – Rat, [^125^I]GnRH I (human, mouse, rat) (Agonist)	–
Comments	–	Probable transcribed pseudogene in man [1284].

### Comments

GnRH_1_ and GnRH_2_ receptors couple primarily to G_q/11_[653] but coupling to G_s_ and G_i_ is evident in some systems [1009, 1024]. GnRH_2_ receptors may also mediate (heterotrimeric) G protein‐independent signalling to protein kinases [276]. There is increasing evidence for expression of GnRH receptors on hormone‐dependent cancer cells where they can exert antiproliferative and/or proapoptotic effects and mediate effects of cytotoxins conjugated to GnRH analogues [309, 706, 1108, 1661]. In some human cancer cell models GnRH II(*GNRH2*, O43555) is more potent than GnRH I (*GNRH1*, P01148), implying mediation by GnRH_2_ receptors [657]. However, GnRH_2_ receptors that are expressed by some primates are probably not expressed in humans because the human GNRHR_2_ gene contains a frame shift and internal stop codon [1325]. The possibility remains that this gene generates GnRH_2_ receptor‐related proteins (other than the full‐length receptor) that mediate responses to GnRH II(*GNRH2*, O43555) (see [1372]). Alternatively, there is evidence for multiple active GnRH receptor conformations [276, 277, 516, 1231, 1284] raising the possibility that GnRH_1_ receptor‐mediated proliferation inhibition in hormone‐dependent cancer cells is dependent upon different conformations (with different ligand specificity and ligand biased signalling) than effects on G_q/11_ in pituitary cells [277, 1231]. Loss‐of‐function mutations in the GnRH_1_ receptor and deficiency of GnRH I (*GNRH1*, P01148) are associated with hypogonadotropic hypogonadism although some ‘loss of function’ mutations may actually prevent trafficking of ‘functional’ GnRH_1_ receptors to the cell surface, as evidenced by recovery of function by nonpeptide antagonists [1061]. Human GnRH_1_ receptors appear to be poorly expressed at the cell surface because of failure to meet structural quality control criteria for endoplasmic reticulum exit [517, 1061]. This may increase susceptibility to point mutations that further impair trafficking and also increase effects of non‐peptide antagonists on GnRH_1_ receptor trafficking to the plasma membrane [517, 1061]. GnRH receptor signalling may be dependent upon receptor oligomerisation [363, 1007].

### Further Reading

Bianco SD *et al*. (2009) The genetic and molecular basis of idiopathic hypogonadotropic hypogonadism. *Nat Rev Endocrinol* **5**: 569‐76 [PMID:19707180]

Bliss SP *et al*. (2010) GnRH signaling, the gonadotrope and endocrine control of fertility. *Front Neuroendocrinol* **31**: 322‐40 [PMID:20451543]

Limonta P *et al*. (2012) GnRH receptors in cancer: from cell biology to novel targeted therapeutic strategies. *Endocr. Rev.* **33**: 784‐811 [PMID:22778172]

McArdle CA and Roberson MS.. (2015) Gonadotropes and gonadotropin‐releasing hormone signaling. *In Knobil and Neill's Physiology of Reproduction (4th edition).* Edited by Plant TM and Zeleznik AJ.: Elsevier Inc.: [ISBN: 9780123971753]

Millar RP *et al*. (2004) Gonadotropin‐releasing hormone receptors. *Endocr Rev* **25**: 235‐275 [PMID:15082521]

Tao YX *et al*. (2014) Chaperoning G protein‐coupled receptors: from cell biology to therapeutics. *Endocr. Rev.* **35**: 602‐47 [PMID:24661201]

## GPR18, GPR55 and GPR119

### Overview

GPR18, GPR55 and GPR119 (**provisional nomenclature**), although showing little structural similarity to CB_1_ and CB_2_ cannabinoid receptors, respond to endogenous agents analogous to the endogenous cannabinoid ligands, as well as some natural/synthetic cannabinoid receptor ligands [1494]. Although there are multiple reports to indicate that GPR18, GPR55 and GPR119 can be activated *in vitro* by N‐arachidonoylglycine, lysophosphatidylinositol and N‐oleoylethanolamide, respectively, there is a lack of evidence for activation by these lipid messengers *in vivo*. As such, therefore, these receptors retain their orphan status.

**Table:** 

Nomenclature	*GPR18*	*GPR55*	*GPR119*
HGNC, UniProt	*GPR18*, Q14330	*GPR55*, Q9Y2T6	*GPR119*, Q8TDV5
Rank order of potency	–	–	N‐oleoylethanolamide, N‐palmitoylethanolamine>SEA (anandamide is ineffective) [1452]
Endogenous agonists	N‐arachidonoylglycine [980]	lysophosphatidylinositol (pEC_50_ 5.5–7.3) [738, 1435, 1785], 2‐arachidonoylglycerolphosphoinositol (Selective) [1437]	N‐oleoylethanolamide (pEC_50_ 5.4–6.3) [338, 1452, 1785], N‐palmitoylethanolamine, SEA
Selective agonists	–	AM251 (pEC_50_ 5–7.4) [738, 905, 1620]	AS1269574 (pEC_50_ 5.6) [2100], PSN632408 (pEC_50_ 5.3) [1452], PSN375963 (pEC_50_ 5.1) [1452]
Selective antagonists	–	CID16020046 (apparent pA_2_) (pA_2_ 7.3) [906]	–
Comments	The pairing of N‐arachidonoylglycine with GPR18 was not replicated in two studies based on arrestin assays [1785, 2093]. See [396] for discussion.	See reviews [396] and [1732].	In addition to those shown above, further small molecule agonists have been reported [687].

### Comments

GPR18 failed to respond to a variety of lipid‐derived agents in an *in vitro* screen [2093], but has been reported to be activated by *Δ*^9^‐tetrahydrocannabinol[1246]. GPR55 responds to AM251 and rimonabant at micromolar concentrations, compared to their nanomolar affinity as CB_1_ receptor antagonists/inverse agonists [1494]. It has been reported that lysophosphatidylinositol acts at other sites in addition to GPR55 [2075]. N‐Arachidonoylserine has been suggested to act as a low efficacy agonist/antagonist at GPR18 *in vitro*[1244]. It has also been suggested oleoyl‐lysophosphatidylcholine acts, at least in part, through GPR119 [1400]. Although PSN375963 and PSN632408 produce GPR119‐dependent responses in heterologous expression systems, comparison with N‐oleoylethanolamide‐mediated responses suggests additional mechanisms of action [1400].

### Further Reading

Alexander SP. (2012) So what do we call GPR18 now? *Br. J. Pharmacol.* **165**: 2411‐3 [PMID:22014123]

Davenport AP *et al*. (2013) International Union of Basic and Clinical Pharmacology. LXXXVIII. G protein‐coupled receptor list: recommendations for new pairings with cognate ligands. *Pharmacol. Rev.* **65**: 967‐86 [PMID:23686350]

Hansen HS *et al*. (2012) GPR119 as a fat sensor. *Trends Pharmacol. Sci.* **33**: 374‐81 [PMID:22560300]

McHugh D. (2012) GPR18 in Microglia: implications for the CNS and endocannabinoid system signalling. *Br J Pharmacol* [PMID:22563843]

Pertwee RG *et al*. (2010) International Union of Basic and Clinical Pharmacology. LXXIX. Cannabinoid receptors and their ligands: beyond CB_1 and CB_2. *Pharmacol. Rev.* **62**: 588‐631 [PMID:21079038]

Ross RA. (2011) L‐*α*‐lysophosphatidylinositol meets GPR55: a deadly relationship. *Trends Pharmacol. Sci.* **32**: 265‐9 [PMID:21367464]

Yamashita A *et al*. (2013) The actions and metabolism of lysophosphatidylinositol, an endogenous agonist for GPR55. *Prostaglandins Other Lipid Mediat.* [PMID:23714700]

Zhao P *et al*. (2013) GPR55 and GPR35 and their relationship to cannabinoid and lysophospholipid receptors. *Life Sci.* **92**: 453‐7 [PMID:22820167]

## Histamine receptors

### Overview

Histamine receptors (**nomenclature as agreed by the NC‐IUPHAR Subcommittee on Histamine Receptors [** **754** **,** **1459** **]**) are activated by the endogenous ligand histamine. Marked species differences exist between histamine receptor orthologues [754].

**Table:** 

Nomenclature	H_1_ receptor	H_2_ receptor	H_3_ receptor	H_4_ receptor
HGNC, UniProt	*HRH1*, P35367	*HRH2*, P25021	*HRH3*, Q9Y5N1	*HRH4*, Q9H3N8
Selective agonists	methylhistaprodifen (p*K*_i_ 6.4) [1695], histaprodifen (p*K*_i_ 5.7) [1107]	amthamine (pEC_50_ 6.4) [1003]	immethridine (p*K*_i_ 9.1) [963], methimepip (p*K*_i_ 9) [962], MK‐0249 (Inverse agonist) (p*K*_i_ 8.8) [1354]	clobenpropit (Partial agonist) (p*K*_i_ 7.4–8.3) [490, 1107, 1122, 1123, 1335], 4‐methylhistamine (p*K*_i_ 7.3–8.2) [586, 1107], VUF 8430 (p*K*_i_ 7.5) [1106]
Antagonists	cyproheptadine (p*K*_i_ 10.2) [1298], promethazine (p*K*_i_ 9.6) [601], pyrilamine (Inverse agonist) (p*K*_i_ 8.7–9) [184, 1563], hydroxyzine (p*K*_i_ 8.7) [608], ketotifen (p*K*_i_ 8.6) [1014], cetirizine (Inverse agonist) (p*K*_i_ 8.2) [1298], diphenhydramine (p*K*_i_ 7.9) [184]	–	iodophenpropit (p*K*_i_ 8.2–8.7) [2022, 2051], thioperamide (p*K*_i_ 7.1–7.7) [355, 489, 490, 1104, 1145, 2022, 2051]	–
Selective antagonists	clemastine (p*K*_i_ 10.3) [68], desloratadine (p*K*_i_ 9) [1090], triprolidine (p*K*_i_ 8.5–9) [184, 1298], azelastine (p*K*_i_ 8.9) [1535], astemizole (p*K*_i_ 8.5) [1480], cyclizine (p*K*_i_ 8.4) [68], chlorpheniramine (p*K*_i_ 8.1) [1535], fexofenadine (p*K*_i_ 7.6) [64], loratadine (p*K*_i_ 7.4) [850], terfenadine (p*K*_i_ 7.4) [64], tripelennamine (pIC_50_ 7.4) [635]	tiotidine (p*K*_i_ 7.5) [145] – Rat, ranitidine (p*K*_i_ 7.1) [1086], cimetidine (p*K*_i_ 6.8) [263]	clobenpropit (p*K*_i_ 8.4–9.4) [355, 490, 1104, 1122, 1145, 2022, 2051], A331440 (p*K*_i_ 8.5) [688]	JNJ 7777120 (p*K*_i_ 7.8–8.3) [1107, 1771, 1881]
Labelled ligands	[^3^H]pyrilamine (Antagonist, Inverse agonist) (p*K*_d_ 8.4–9.1) [403, 1298, 1675, 1695], [^11^C]doxepin (Antagonist) (p*K*_d_ 9) [830], [^11^C]pyrilamine (Antagonist, Inverse agonist)	[^125^I]iodoaminopotentidine (Antagonist) (p*K*_d_ 8.7) [1029] – Rat, [^3^H]tiotidine (Antagonist) (p*K*_d_ 7.7–8.7) [1310]	[^123^I]iodoproxyfan (Antagonist) (p*K*_d_ 10.2) [1104], [^125^I]iodophenpropit (Antagonist) (p*K*_d_ 9.2) [849] – Rat, [^3^H](*R*)‐*α*‐methylhistamine (Agonist) (p*K*_d_ 9.2) [1122], *N*‐[^3^H]*α*‐methylhistamine (Agonist) (p*K*_d_ 9) [301] – Mouse	[^3^H]JNJ 7777120 (Antagonist) (p*K*_d_ 8.4) [1881]

### Comments

histaprodifen and methylhistaprodifen are reduced efficacy agonists. The H_4_ receptor appears to exhibit broadly similar pharmacology to the H_3_ receptor for imidazole‐containing ligands, although (*R*)‐*α*‐methylhistamine and *N*‐*α*‐methylhistamine are less potent, while clobenpropit acts as a reduced efficacy agonist at the H_4_ receptor and an antagonist at the H_3_ receptor [1122, 1360, 1390, 1422, 2132]. Moreover, 4‐methylhistamine is identified as a high affinity, full agonist for the human H_4_ receptor [1107]. [^3^H]histamine has been used to label the H_4_ receptor in heterologous expression systems.

### Further Reading

Berlin M *et al*. (2011) Histamine H3 receptor as a drug discovery target. *J. Med. Chem.* **54**: 26‐53 [PMID:21062081]

Hill SJ *et al*. (1997) International Union of Pharmacology. XIII. Classification of histamine receptors. *Pharmacol. Rev.* **49**: 253‐278 [PMID:9311023]

Leurs R *et al*. (2011) En route to new blockbuster anti‐histamines: surveying the offspring of the expanding histamine receptor family. *Trends Pharmacol. Sci.* **32**: 250‐7 [PMID:21414671]

Marson CM. (2011) Targeting the histamine H4 receptor. *Chem. Rev.* **111**: 7121‐56 [PMID:21842846]

Passani MB *et al*. (2011) Histamine receptors in the CNS as targets for therapeutic intervention. *Trends Pharmacol. Sci.* **32**: 242‐9 [PMID:21324537]

Schwartz JC. (2011) The histamine H3 receptor: from discovery to clinical trials with pitolisant. *Br. J. Pharmacol.* **163**: 713‐21 [PMID:21615387]

## Hydroxycarboxylic acid receptors

### Overview

The hydroxycarboxylic acid family of receptors (ENSFM00500000271913, **nomenclature as agreed by the NC‐IUPHAR Subcommittee on Hydroxycarboxylic acid receptors [** **396** **,** **1424** **]**) respond to organic acids, including the endogenous hydroxy carboxylic acids 3‐hydroxy butyric acid and L‐lactic acid, as well as the lipid lowering agents nicotinic acid (niacin), acipimox and acifran[1774, 1913, 2036]. These receptors were provisionally described as nicotinic acid receptors, although nicotinic acid shows submicromolar potency at HCA_2_receptors only and is unlikely to be the natural ligand [1913, 2036].

**Table:** 

Nomenclature	HCA_1_ receptor	HCA_2_ receptor	HCA_3_ receptor
HGNC, UniProt	*HCAR1*, Q9BXC0	*HCAR2*, Q8TDS4	*HCAR3*, P49019
Endogenous agonists	L‐lactic acid (Selective) (pEC_50_ 1.3–2.9) [14, 256, 1124, 1785]	*β*‐D‐hydroxybutyric acid (pEC_50_ 3.1) [1838]	3‐hydroxyoctanoic acid (pEC_50_ 5.1) [13]
Agonists	compound 2 [PMID: 24486398] (pEC_50_ 7.2) [1630], 3,5‐dihydroxybenzoic acid (pEC_50_ 3.7) [1121]	SCH 900271 (pEC_50_ 8.7) [1454], GSK256073 (pEC_50_ 7.5) [1790]	–
Selective agonists	–	MK 6892 (pEC_50_ 7.8) [1719], MK 1903 (pEC_50_ 7.6) [163], nicotinic acid (pEC_50_ 6–7.2) [1774, 1913, 2036], acipimox (pEC_50_ 5.2–5.6) [1774, 2036], monomethylfumarate (pEC_50_ 5) [1859]	compound 6o [PMID: 19524438] (pEC_50_ 8.5) [1751], IBC 293 (pEC_50_ 6.4) [1697]
Labelled ligands	–	[^3^H]nicotinic acid (Agonist) (p*K*_d_ 7–7.3) [1774, 1913, 2036]	–

### Comments

Further closely‐related GPCRs include the 5‐oxoeicosanoid receptor (*OXER1*, Q8TDS5) and *GPR31*(O00270).

### Further Reading

Chapman MJ *et al*. (2010) Niacin and fibrates in atherogenic dyslipidemia: pharmacotherapy to reduce cardiovascular risk. *Pharmacol. Ther.* **126**: 314‐45 [PMID:20153365]

Digby JE *et al*. (2012) Niacin in cardiovascular disease: recent preclinical and clinical developments. *Arterioscler. Thromb. Vasc. Biol.* **32**: 582‐8 [PMID:22207729]

Hanson J *et al*. (2012) Role of HCA_2(GPR109A) in nicotinic acid and fumaric acid ester‐induced effects on the skin. *Pharmacol. Ther.* **136**: 1‐7 [PMID:22743741]

Kamanna VS *et al*. (2013) Recent advances in niacin and lipid metabolism. *Curr. Opin. Lipidol.* **24**: 239‐45 [PMID:23619367]

Offermanns S. (2014) Free fatty acid (FFA) and hydroxy carboxylic acid (HCA) receptors. *Annu. Rev. Pharmacol. Toxicol.* **54**: 407‐34 [PMID:24160702]

Offermanns S *et al*. (2011) International Union of Basic and Clinical Pharmacology. LXXXII: Nomenclature and Classification of Hydroxy‐carboxylic Acid Receptors (GPR81, GPR109A, and GPR109B). *Pharmacol. Rev.* **63**: 269‐90 [PMID:21454438]

Offermanns S *et al*. (2015) Nutritional or pharmacological activation of HCA(2) ameliorates neuroinflammation. *Trends Mol Med* **21**: 245‐55 [PMID:25766751]

## Kisspeptin receptor

### Overview

The kisspeptin receptor (**nomenclature as agreed by the NC‐IUPHAR Subcommittee on the kisspeptin receptor [** **958** **]**), like neuropeptide FF (NPFF), prolactin‐releasing peptide (PrP) and QRFP receptors (provisional nomenclature) responds to endogenous peptides with an arginine‐phenylalanine‐amide (RFamide) motif. Kisspeptin‐54(*KISS1*, Q15726) (KP54, originally named metastin), kisspeptin‐13(*KISS1*, Q15726) (KP13) and kisspeptin‐10(*KISS1*) (KP10) are biologically‐active peptides cleaved from the *KISS1*(Q15726) gene product.

**Table:** 

Nomenclature	kisspeptin receptor
HGNC, UniProt	*KISS1R*, Q969F8
Endogenous agonists	kisspeptin‐10 (*KISS1*) (Selective) (p*K*_i_ 8.6–10.4) [996, 1434], kisspeptin‐54 (*KISS1*, Q15726) (Selective) (p*K*_i_ 8.8–9.5) [996, 1434], kisspeptin‐14 (*KISS1*, Q15726) (p*K*_i_ 8.8) [996], kisspeptin‐13 (*KISS1*, Q15726) (Selective) (p*K*_i_ 8.4) [996]
Selective agonists	4‐fluorobenzoyl‐FGLRW‐NH2 (pEC_50_ 9.2) [1894], [dY]^1^KP‐10 (pIC_50_ 8.4) [385] – Mouse
Selective antagonists	peptide 234 [1600]
Labelled ligands	[^125^I]Tyr^45^‐kisspeptin‐15 (Agonist) (p*K*_d_ 10) [1434], [^125^I]kisspeptin‐13 (human) (Agonist) (p*K*_d_ 9.7) [1252], [^125^I]kisspeptin‐10 (human) (Agonist) (p*K*_d_ 8.7) [996], [^125^I]kisspeptin‐14 (human) (Agonist) [1252]

### Further Reading

Kanda S *et al*. (2013) Structure, synthesis, and phylogeny of kisspeptin and its receptor. *Adv. Exp. Med. Biol.* **784**: 9‐26 [PMID:23550000]

Kirby HR *et al*. (2010) International Union of Basic and Clinical Pharmacology. LXXVII. Kisspeptin receptor nomenclature, distribution, and function. *Pharmacol. Rev.* **62**: 565‐78 [PMID:21079036]

Millar RP *et al*. (2010) Kisspeptin antagonists: unraveling the role of kisspeptin in reproductive physiology. *Brain Res.* **1364**: 81‐9 [PMID:20858467]

Pasquier J *et al*. (2014) Molecular evolution of GPCRs: Kisspeptin/kisspeptin receptors. *J. Mol. Endocrinol.* **52**: T101‐17 [PMID:24577719]

Roseweir AK *et al*. (2013) Kisspeptin antagonists. *Adv. Exp. Med. Biol.* **784**: 159‐86 [PMID:23550006]

## Leukotriene receptors

### Overview

Leukotriene receptors (**nomenclature as agreed by the NC‐IUPHAR subcommittee on Leukotriene Receptors [** **249** **,** **250** **]**) is activated by the endogenous ligands leukotrienes (LT), synthesized from lipoxygenase metabolism of arachidonic acid.

The human BLT_1_ receptor is the high affinity LTB_4_ receptor whereas the BLT_2_ receptor in addition to being a low‐affinity LTB_4_ receptor also binds several other lipoxygenase‐products, such as 12S‐HETE, 12S‐HPETE, 15S‐HETE, and the thromboxane synthase product 12‐hydroxyheptadecatrienoic acid. The BLT receptors mediate chemotaxis and immunomodulation in several leukocyte populations and are in addition expressed on non‐myeloid cells, such as vascular smooth muscle and endothelial cells. In addition to BLT receptors, LTB_4_ has been reported to bind to the peroxisome proliferator activated receptor (PPAR) *α*[1112] and the vanilloid TRPV1 ligand‐gated nonselective cation channel [1245].

The receptors for the cysteinyl‐leukotrienes (*i.e.* LTC_4_, LTD_4_ and LTE_4_) are termed CysLT_1_ and CysLT_2_ and exhibit distinct expression patterns in human tissues, mediating for example smooth muscle cell contraction, regulation of vascular permeability, and leukocyte activation. There is also evidence in the literature for additional CysLT receptor subtypes, derived from functional in vitro studies, radioligand binding and in mice lacking both CysLT_1_ and CysLT_2_ receptors [250]. Cysteinyl‐leukotrienes have also been suggested to signal through the P2Y_12_ receptor [542, 1407, 1466], GPR17 [344] and GPR99 [900].

**Table:** 

Nomenclature	BLT_1_ receptor	BLT_2_ receptor	CysLT_1_ receptor	CysLT_2_ receptor
HGNC, UniProt	*LTB4R*, Q15722	*LTB4R2*, Q9NPC1	*CYSLTR1*, Q9Y271	*CYSLTR2*, Q9NS75
Rank order of potency	LTB_4_>20‐hydroxy‐LTB_4_≫12R‐HETE [2096]	12‐hydroxyheptadecatrienoic acid>LTB_4_>12S‐HETE = 12S‐HPETE>15S‐HETE>12R‐HETE>20‐hydroxy‐LTB_4_ [1442, 2096]	LTD_4_>LTC_4_>LTE_4_ [1157, 1643]	LTC_4_≥LTD_4_≫LTE_4_ [733, 1411, 1847]
Endogenous agonists	–	12S‐HETE (Partial agonist) (pEC_50_<7.5) [2096]	–	–
Antagonists	–	–	ICI198615 (‐ 8.4–8.6)	BAYu9773 (against LTC_4_ and LTD_4_ induced contraction in smooth muscle preparation) (pA_2_ 6.8–7.7) [1912] – Rat
Selective antagonists	BIIL 260 (p*K*_i_ 8.8) [152, 457], CP105696 (pIC_50_ 8.1) [1735], U75302 (p*K*_i_ 6.4) [172]	LY255283 (pIC_50_ 6–7.1) [744, 2096]	zafirlukast (against [^3^H]LTD_4_ in COS‐7 or HEK‐293 cells) (pIC_50_ 8.6–8.7) [1157, 1643], SR2640 (p*K*_i_ 8.7), montelukast (against [^3^H]LTD4 in COS‐7 or HEK‐293 cells) (pIC_50_ 8.3–8.6) [1157, 1643], sulukast (p*K*_i_ 8.3), pobilukast (against [^3^H]LTD_4_ in HEK‐293) (pIC_50_ 7.5) [1643]	BayCysLT_2_ (against 30‐300nM LTD_4_ *β*‐arrestin assay in C2C12 myofibroblasts) (pIC_50_ 6.6–7.3) [1391]
Labelled ligands	[^3^H]LTB_4_ (Agonist) (p*K*_d_ 9.8) [2095], [^3^H]CGS23131 (Antagonist) (p*K*_d_ 7.9) [836]	[^3^H]LTB_4_ (p*K*_d_ 7.6–9.7)	[^3^H]LTD_4_ (Agonist) (p*K*_d_ 8–10.7), [^3^H]ICI‐198615 (Antagonist) (p*K*_d_ 10.6) [1608]	[^3^H]LTD_4_ (Agonist) (p*K*_d_ 7.3–9.4) [733]

**Table:** 

Nomenclature	FPR2/ALX	OXE receptor
HGNC, UniProt	*FPR2*, P25090	*OXER1*, Q8TDS5
Rank order of potency	LXA_4_=aspirin triggered lipoxin A4=ATLa2>LTC_4_=LTD_4_≫15‐deoxy‐LXA4≫fMet‐Leu‐Phe [352, 519, 521, 651, 1846]	5‐oxo‐ETE, 5‐oxo‐C20:3, 5‐oxo‐ODE>5‐oxo‐15‐HETE>5S‐HPETE>5S‐HETE [784, 877, 1472]
Endogenous agonists	LXA_4_ (Selective) (pEC_50_∼12) [1006], resolvin D1 (Selective) (pEC_50_∼11.9) [1006], aspirin triggered lipoxin A4 (Selective)	5‐oxo‐ETE (Selective) (pEC_50_ 8.3–8.5) [638, 1417, 1472, 1525, 1681]
Selective agonists	ATLa2 [662]	–
Endogenous antagonists	–	5‐oxo‐12‐HETE (Selective) (pIC_50_ 6.3) [1524]
Antagonists	–	–
Selective antagonists	–	–
Labelled ligands	[^3^H]LXA_4_ (Agonist) (p*K*_d_ 9.2–9.3) [519, 520]	[^3^H]5‐oxo‐ETE (Agonist) (p*K*_d_ 8.4) [1417]
Comments	The agonist activity of the lipid mediators described has been questioned [697, 1513], which may derive from batch‐to‐batch differences, partial agonism or biased agonism. Recent results from Cooray *et al.* (2013) [365] have addressed this issue and the role of homodimers and heterodimers in the intracellular signaling .	–

### Comments

The FPR2/ALX receptor (**nomenclature as agreed by the NC‐IUPHAR subcommittee on Leukotriene and Lipoxin Receptors [** **250** **]**) is activated by the endogenous lipid‐derived, anti‐inflammatory ligands lipoxin A_4_(LXA_4_) and 15‐epi‐LXA_4_(aspirin triggered lipoxin A4, ATL). The FPR2/ALX receptor also interacts with endogenous peptide and protein ligands, such as MHC binding peptide [315] as well as annexin I (*ANXA1*, P04083) (ANXA1) and its *N*‐terminal peptides [365, 1491]. In addition, a soluble hydrolytic product of protease action on the urokinase‐type plasminogen activator receptor has been reported to activate the FPR2/ALX receptor [1572]. Furthermore, FPR2/ALX has been suggested to act as a receptor mediating the proinflammatory actions of the acute‐phase reactant, serum amyloid A [1772, 1809]. A receptor selective for LXB_4_ has been suggested from functional studies [53, 1168, 1596]. Note that the data for FPR2/ALX are also reproduced on the Formylpeptide receptor pages.

Oxoeicosanoid receptors (**OXE, nomenclature agreed by the NC‐IUPHAR subcommittee on Oxoeicosanoid Receptors [** **214** **]**) are activated by endogenous chemotactic eicosanoid ligands oxidised at the C‐5 position, with 5‐oxo‐ETE the most potent agonist identified for this receptor. Initial characterization of the heterologously expressed OXE receptor suggested that polyunsaturated fatty acids, such as docosahexaenoic acid and EPA, acted as receptor antagonists [784]

### Further Reading

Bäck M *et al*. (2014) Update on leukotriene, lipoxin and oxoeicosanoid receptors: IUPHAR Review 7. *Br. J. Pharmacol.* **171**: 3551‐74 [PMID:24588652]

Cooray SN *et al*. (2013) Ligand‐specific conformational change of the G‐protein‐coupled receptor ALX/FPR2 determines proresolving functional responses. *Proc. Natl. Acad. Sci. U.S.A.* **110**: 18232‐7 [PMID:24108355]

Nelson JW *et al*. (2014) ALX/FPR2 receptor for RvD1 is expressed and functional in salivary glands. *Am. J. Physiol., Cell Physiol.* **306**: C178‐85 [PMID:24259417]

Norling LV *et al*. (2012) Resolvin D1 limits polymorphonuclear leukocyte recruitment to inflammatory loci: receptor‐dependent actions. *Arterioscler. Thromb. Vasc. Biol.* **32**: 1970‐8 [PMID:22499990]

## Lysophospholipid (LPA) receptors

### Overview

Lysophosphatidic acid (LPA) receptors (**nomenclature as agreed by the NC‐IUPHAR Subcommittee on Lysophospholipid Receptors [** **396** **,** **936** **]**) are activated by the endogenous phospholipid metabolite LPA. The first receptor, LPA_1_, was identified as *ventricular zone gene‐1* (*vzg‐1*), leading to deorphanisation of members of the endothelial differentiation gene (*edg*) family as other LPA receptors along with sphingosine 1‐phosphate (S1P) receptors. Additional LPA receptor GPCRs were later identified. Gene names have been codified as *LPAR1*, etc. to reflect the receptor function of proteins. The crystal structure of LPA_1_ was recently solved and demonstrates ligand access characteristics that allows for extracellular LPA binding [331]; these studies have also implicated cross‐talk with endocannabinoids *via* phosphorylated intermediates that can activate this receptor. The identified receptors can account for most, although not all, LPA‐induced phenomena in the literature, indicating that a majority of LPA‐dependent phenomena are receptor‐mediated. Radioligand binding has been conducted in heterologous expression systems using [^3^H]LPA(*e.g.* [556]). In native systems, analysis of binding data is complicated by metabolism and high levels of nonspecific binding, and therefore the relationship between recombinant and endogenously expressed receptors is unclear. Targeted deletion of LPA receptors has clarified signalling pathways and identified physiological and pathophysiological roles. Independent validation by multiple groups has been reported in the peer‐reviewed literature for all six LPA receptors described in the tables, including further validation using a distinct read‐out via a novel TGF*α*“shedding” assay [825]. LPA has also been described as an agonist at other orphan GPCRs (PSP24, GPR87 and GPR35), as well as at the nuclear hormone PPAR*γ* receptors [1247, 1743], although the physiological significance of these observations remain unclear.

**Table:** 

Nomenclature	LPA_1_ receptor	LPA_2_ receptor	LPA_3_ receptor
HGNC, UniProt	*LPAR1*, Q92633	*LPAR2*, Q9HBW0	*LPAR3*, Q9UBY5
Selective agonists	–	dodecylphosphate (pEC_50_ 6.2) [1958], decyl dihydrogen phosphate (pEC_50_ 5.4) [1958], GRI977143 (pEC_50_ 4.5) [959]	OMPT (pEC_50_ 7.2) [709]
Selective antagonists	AM966 (pIC_50_ 6.7–7.8) [1832]	–	dioctanoylglycerol pyrophosphate (p*K*_i_ 5.5–7) [522, 1432]
Comments	–	Virtual screening experiments have shown H2L5186303 to be a potent antagonist of LPA_2_ [510]. dodecylphosphate is also an antagonist at LPA_3_ receptors [1958].	–

**Table:** 

Nomenclature	LPA_4_ receptor	LPA_5_ receptor	LPA_6_ receptor
HGNC, UniProt	*LPAR4*, Q99677	*LPAR5*, Q9H1C0	*LPAR6*, P43657

### Comments

Ki16425[1432], VPC12249[735] and VPC32179[729] have antagonist activity at LPA_1_ and LPA_3_ receptors. There is growing evidence for *in vivo* efficacy of these chemical antagonists in several disorders, including fetal hydrocephalus [2107], lung fibrosis [1429], and systemic sclerosis [1429].

### Further Reading

Chun J *et al*. (2010) International Union of Basic and Clinical Pharmacology. LXXVIII. Lysophospholipid receptor nomenclature. *Pharmacol. Rev.* **62**: 579‐87 [PMID:21079037]

Kihara Y *et al*. (2014) Lysophospholipid receptor nomenclature review: IUPHAR Review 8. *Br. J. Pharmacol.* [PMID:24602016]

Mirendil H *et al*. (2015) LPA signaling initiates schizophrenia‐like brain and behavioral changes in a mouse model of prenatal brain hemorrhage. *Transl Psychiatry* **5**: e541 [PMID:25849980]

Mutoh T *et al*. (2012) Insights into the pharmacological relevance of lysophospholipid receptors. *Br. J. Pharmacol.* **165**: 829‐44 [PMID:21838759]

Schober A *et al*. (2012) Lysophosphatidic acid in atherosclerotic diseases. *Br. J. Pharmacol.* **167**: 465‐82 [PMID:22568609]

Sheng X *et al*. (2015) Lysophosphatidic acid signalling in development. *Development* **142**: 1390‐5 [PMID:25852197]

Yung YC *et al*. (2014) LPA receptor signaling: pharmacology, physiology, and pathophysiology. *J. Lipid Res.* **55**: 1192‐1214 [PMID:24643338]

Yung YC *et al*. (2015) Lysophosphatidic Acid signaling in the nervous system. *Neuron* **85**: 669‐82 [PMID:25695267]

## Lysophospholipid (S1P) receptors

### Overview

Sphingosine 1‐phosphate (S1P) receptors (**nomenclature as agreed by the NC‐IUPHAR Subcommittee on Lysophospholipid receptors [** **936** **]**) are activated by the endogenous lipid sphingosine 1‐phosphate (S1P) and with lower apparent affinity, sphingosylphosphorylcholine(SPC). Originally cloned as orphan members of the endothelial differentiation gene (*edg*) family, deorphanisation as lysophospholipid receptors for S1P was based on sequence homology to LPA receptors. Current gene names have been codified as S1PR1, etc. to reflect the receptor function of these proteins. Most cellular phenomena ascribed to S1P can be explained by receptor‐mediated mechanisms; S1P has also been described to act at intracellular sites [1841], and awaits precise definition. Previously‐proposed SPC (or lysophophosphatidylcholine) receptors‐ G2A, TDAG8, OGR1 and GPR4 ‐ continue to lack confirmation of these roles [396]. The relationship between recombinant and endogenously expressed receptors is unclear. Radioligand binding has been conducted in heterologous expression systems using [^32^P]S1P(*e.g* [1438]). In native systems, analysis of binding data is complicated by metabolism and high levels of nonspecific binding. Targeted deletion of several S1P receptors and key enzymes involved in S1P biosynthesis or degradation has clarified signalling pathways and physiological roles. A crystal structure of an S1P_1_‐T4 fusion protein has been described [698].

The S1P receptor modulator, fingolimod(FTY720, Gilenya), has received world‐wide approval as the first oral therapy for relapsing forms of multiple sclerosis. This drug has a novel mechanism of action involving modulation of S1P receptors in both the immune and nervous systems [325, 356, 654], although the precise nature of its interaction requires clarification.

**Table:** 

Nomenclature	S1P_1_ receptor	S1P_2_ receptor	S1P_3_ receptor	S1P_4_ receptor	S1P_5_ receptor
HGNC, UniProt	*S1PR1*, P21453	*S1PR2*, O95136	*S1PR3*, Q99500	*S1PR4*, O95977	*S1PR5*, Q9H228
Rank order of potency	sphingosine 1‐phosphate>dihydrosphingosine‐1‐phosphate>sphingosylphosphorylcholine [45, 1438]	sphingosine 1‐phosphate>dihydrosphingosine‐1‐phosphate>sphingosylphosphorylcholine [45, 1438]	sphingosine 1‐phosphate>dihydrosphingosine‐1‐phosphate>sphingosylphosphorylcholine [1438]	sphingosine 1‐phosphate>dihydrosphingosine‐1‐phosphate>sphingosylphosphorylcholine [1936]	sphingosine 1‐phosphate>dihydrosphingosine‐1‐phosphate>sphingosylphosphorylcholine [821]
Agonists	SEW2871 (p*K*_i_ 5.5–7.7) [1640]	–	–	–	–
Antagonists	VPC44116 (p*K*_i_ 8.5) [533], VPC23019 (p*K*_i_ 7.9) [400]	–	VPC44116 (p*K*_i_ 6.5) [533], VPC23019 (p*K*_i_ 5.9) [400]	–	–
Selective antagonists	W146 (p*K*_i_ 7.1) [1641]	JTE‐013 (pIC_50_ 7.8) [1447]	–	–	–
Selective agonists	AUY954 (pEC_50_ 8.9) [1456]	–	–	–	–

### Comments

The approved immunomodulator drug fingolimod can be phosphorylated *in vivo* [31] to generate a relatively potent agonist with activity at S1P_1_, S1P_3_, S1P_4_ and S1P_5_ receptors [215, 1198], although its biological activity appears to involve an element of functional antagonism [339, 356, 1405].

### Further Reading

Chi H. (2011) Sphingosine‐1‐phosphate and immune regulation: trafficking and beyond. *Trends Pharmacol. Sci.* **32**: 16‐24 [PMID:21159389]

Chun J *et al*. (2010) International Union of Basic and Clinical Pharmacology. LXXVIII. Lysophospholipid receptor nomenclature. *Pharmacol. Rev.* **62**: 579‐87 [PMID:21079037]

Kihara Y *et al*. (2015) Lysophospholipid receptors in drug discovery. *Exp. Cell Res.* **333**: 171‐7 [PMID:25499971]

Mutoh T *et al*. (2012) Insights into the pharmacological relevance of lysophospholipid receptors. *Br. J. Pharmacol.* **165**: 829‐44 [PMID:21838759]

O'Sullivan C *et al*. (2013) The structure and function of the S1P1 receptor. *Trends Pharmacol. Sci.* **34**: 401‐12 [PMID:23763867]

Spiegel S *et al*. (2011) The outs and the ins of sphingosine‐1‐phosphate in immunity. *Nat. Rev. Immunol.* **11**: 403‐15 [PMID:21546914]

## Melanin‐concentrating hormone receptors

### Overview

Melanin‐concentrating hormone (MCH) receptors (**provisional nomenclature as recommended by NC‐IUPHAR [** **530** **]**) are activated by an endogenous nonadecameric cyclic peptide identical in humans and rats (DFDMLRCMLGRVYRPCWQV) generated from a precursor (*PMCH*, P20382), which also produces neuropeptide EI(*PMCH*, P20382) and neuropeptide GE (*PMCH*, P20382).

**Table:** 

Nomenclature	MCH_1_ receptor	MCH_2_ receptor
HGNC, UniProt	*MCHR1*, Q99705	*MCHR2*, Q969V1
Rank order of potency	melanin‐concentrating hormone (*PMCH*, P20382) >MCH (salmon)	melanin‐concentrating hormone (*PMCH*, P20382) = MCH (salmon) [753]
Selective antagonists	GW803430 (pIC_50_ 9.3) [745], SNAP‐7941 (pA_2_ 9.2) [186], T‐226296 (pIC_50_ 8.3) [1853], ATC0175 (pIC_50_ 7.9–8.1) [283]	–
Labelled ligands	[^125^I]S36057 (Antagonist) (p*K*_d_ 9.2–9.5) [66], [^125^I][Phe^13^,Tyr^19^]MCH (Agonist) (p*K*_d_ 9.2) [242], [^3^H]MCH (human, mouse, rat) (Agonist) [242]	–

### Comments

The MCH_2_ receptor appears to be a non‐functional pseudogene in rodents [1857].

### Further Reading

Boughton CK *et al*. (2012) Can Neuropeptides Treat Obesity? A review of neuropeptides and their potential role in the treatment of obesity. *Br. J. Pharmacol.* [PMID:23121386]

Chung S *et al*. (2011) Recent updates on the melanin‐concentrating hormone (MCH) and its receptor system: lessons from MCH1R antagonists. *J. Mol. Neurosci.* **43**: 115‐21 [PMID:20582487]

Eberle AN *et al*. (2010) Cellular models for the study of the pharmacology and signaling of melanin‐concentrating hormone receptors. *J. Recept. Signal Transduct. Res.* **30**: 385‐402 [PMID:21083507]

Foord SM *et al*. (2005) International Union of Pharmacology. XLVI. G protein‐coupled receptor list. *Pharmacol Rev* **57**: 279‐288 [PMID:15914470]

Macneil DJ. (2013) The role of melanin‐concentrating hormone and its receptors in energy homeostasis. *Front Endocrinol (Lausanne)* **4**: 49 [PMID:23626585]

Parker JA *et al*. (2012) Hypothalamic neuropeptides and the regulation of appetite. *Neuropharmacology* **63**: 18‐30 [PMID:22369786]

Takase K *et al*. (2014) Meta‐analysis of melanin‐concentrating hormone signaling‐deficient mice on behavioral and metabolic phenotypes. *PLoS ONE* **9**: e99961 [PMID:24924345]

## Melanocortin receptors

### Overview

Melanocortin receptors (**provisional nomenclature as recommended by NC‐IUPHAR[** **530** **]**) are activated by members of the melanocortin family (*α*‐MSH(*POMC*, P01189), *β*‐MSH(*POMC*, P01189) and *γ*‐MSH(*POMC*, P01189) forms; ‐*δ* form is not found in mammals) and adrenocorticotrophin (ACTH(*POMC*, P01189)). Endogenous antagonists include agouti(*ASIP*, P42127) and agouti‐related protein (*AGRP*, O00253).

**Table:** 

Nomenclature	MC_1_ receptor	MC_2_ receptor	MC_3_ receptor	MC_4_ receptor	MC_5_ receptor
HGNC, UniProt	*MC1R*, Q01726	*MC2R*, Q01718	*MC3R*, P41968	*MC4R*, P32245	*MC5R*, P33032
Rank order of potency	*α*‐MSH (*POMC*, P01189) >*β*‐MSH (*POMC*, P01189) >ACTH (*POMC*, P01189), *γ*‐MSH (*POMC*, P01189)	ACTH (*POMC*, P01189)	*γ*‐MSH (*POMC*, P01189), *β*‐MSH (*POMC*, P01189) >ACTH (*POMC*, P01189), *α*‐MSH (*POMC*, P01189)	*β*‐MSH (*POMC*, P01189) >*α*‐MSH (*POMC*, P01189), ACTH (*POMC*, P01189) >*γ*‐MSH (*POMC*, P01189)	*α*‐MSH (*POMC*, P01189) >*β*‐MSH (*POMC*, P01189) >ACTH (*POMC*, P01189) >*γ*‐MSH (*POMC*, P01189)
Selective agonists	–	corticotropin zinchydroxide	[D‐Trp^8^]*γ*‐MSH (pIC_50_ 8.2) [645]	THIQ (pIC_50_ 8.9) [1690]	–
Antagonists	–	–	PG‐106 (pIC_50_ 6.7) [646]	–	–
Selective antagonists	–	–	–	MBP10 (pIC_50_ 10) [117], HS014 (p*K*_i_ 8.5) [1667]	–
Labelled ligands	[^125^I]NDP‐MSH (Agonist) (p*K*_d_ 9.5) [991]	[^125^I]ACTH‐(1‐24) (Agonist)	[^125^I]NDP‐MSH (Agonist) (p*K*_d_ 9.7) [991], [^125^I]SHU9119 (Antagonist) [1392]	[^125^I]SHU9119 (Antagonist) (p*K*_d_ 9.2) [1392], [^125^I]NDP‐MSH (Agonist) (p*K*_d_ 8.4–8.9) [991, 1665]	[^125^I]NDP‐MSH (Agonist) (p*K*_d_ 8.6) [991]

### Comments

Polymorphisms of the MC1 receptor have been linked to variations in skin pigmentation. Defects of the MC2 receptor underlie familial glucocorticoid deficiency. Polymorphisms of the MC4 receptor have been linked to obesity [282, 505].

### Further Reading

Beaumont KA *et al*. (2011) Melanocortin MC_1 receptor in human genetics and model systems. *Eur. J. Pharmacol.* **660**: 103‐10 [PMID:21199646]

Cooray SN *et al*. (2011) Melanocortin receptors and their accessory proteins. *Mol. Cell. Endocrinol.* **331**: 215‐21 [PMID:20654690]

Foord SM *et al*. (2005) International Union of Pharmacology. XLVI. G protein‐coupled receptor list. *Pharmacol Rev* **57**: 279‐288 [PMID:15914470]

Holloway PM *et al*. (2011) Targeting the melanocortin receptor system for anti‐stroke therapy. *Trends Pharmacol. Sci.* **32**: 90‐8 [PMID:21185610]

Hruby VJ *et al*. (2011) Design of novel melanocortin receptor ligands: multiple receptors, complex pharmacology, the challenge. *Eur. J. Pharmacol.* **660**: 88‐93 [PMID:21208601]

Loos RJ. (2011) The genetic epidemiology of melanocortin 4 receptor variants. *Eur. J. Pharmacol.* **660**: 156‐64 [PMID:21295023]

Meimaridou E *et al*. (2013) ACTH resistance: genes and mechanisms. *Endocr Dev* **24**: 57‐66 [PMID:23392095]

Renquist BJ *et al*. (2011) Physiological roles of the melanocortin MC_3 receptor. *Eur. J. Pharmacol.* **660**: 13‐20 [PMID:21211527]

Yang Y. (2011) Structure, function and regulation of the melanocortin receptors. *Eur. J. Pharmacol.* **660**: 125‐30 [PMID:21208602]

## Melatonin receptors

### Overview

Melatonin receptors (**nomenclature as agreed by the NC‐IUPHARSubcommittee on Melatonin Receptors [** **446** **]**) are activated by the endogenous ligands melatonin and N‐acetylserotonin.

**Table:** 

Nomenclature	MT_1_ receptor	MT_2_ receptor
HGNC, UniProt	*MTNR1A*, P48039	*MTNR1B*, P49286
Endogenous agonists	melatonin (p*K*_i_ 9.1–9.7) [67, 445, 447]	melatonin (p*K*_i_ 9.4–9.8) [67, 445, 447]
Agonists	ramelteon (p*K*_i_ 10.9) [909], agomelatine (p*K*_i_ 10–10.4) [67, 132]	agomelatine (p*K*_i_ 9.9–10.5) [67, 132], ramelteon (p*K*_i_ 10) [909, 1565]
Selective agonists	–	IIK7 (p*K*_i_ 10.3) [506, 1814], 5‐methoxy‐luzindole (Partial agonist) (p*K*_i_ 9.6) [447]
Selective antagonists	–	4P‐PDOT (p*K*_i_ 8.8–9.4) [67, 447, 448], K185 (p*K*_i_ 9.3) [506, 1814], DH97 (p*K*_i_ 8) [1865]
Labelled ligands	[^125^I]SD6 (Agonist) (p*K*_d_ 10.9) [1074], 2‐[^125^I]melatonin (Agonist) (p*K*_d_ 9.9–10.7) [67, 447], [^3^H]melatonin (Agonist) (p*K*_d_ 9.4–9.9) [230]	[^125^I]SD6 (Agonist) (p*K*_d_ 10.2) [1074], 2‐[^125^I]melatonin (Agonist) (p*K*_d_ 9.7–10) [67, 447], [^125^I]DIV880 (Agonist, Partial agonist) (p*K*_d_ 9.7) [1074], [^3^H]melatonin (Agonist) (p*K*_d_ 9–9.6) [230]

### Comments

melatonin, 2‐iodo‐melatonin, agomelatine, GR 196429, LY 156735 and ramelteon[909] are nonselective agonists for MT_1_ and MT_2_ receptors. (‐)‐AMMTC displays an ~400‐fold greater agonist potency than (+)‐AMMTC at rat MT_1_ receptors (see AMMTC for structure) [1888]. Luzindole is an MT_1_/MT_2_ melatonin receptor‐selective competitive antagonist with some selectivity for the MT2 receptor [448]. MT_1_/MT_2_ heterodimers present different pharmacological profiles from MT_1_ and MT_2_ receptors [72].

The *MT_3_* binding site of hamster brain and peripheral tissues such as kidney and testis, also termed the ML_2_ receptor, binds selectively 2‐iodo‐[^125^I]5MCA‐NAT[1302]. Pharmacological investigations of *MT_3_* binding sites have primarily been conducted in hamster tissues. At this site, N‐acetylserotonin[467, 1149, 1302, 1516] and 5MCA‐NAT[1516] appear to function as agonists, while prazosin[1149] functions as an antagonist. The *MT_3_* binding site of hamster kidney was also identified as the hamster homologue of human quinone reductase 2 (*NQO2*, P16083[1408, 1409]). The *MT_3_* binding site activated by 5MCA‐NAT in eye ciliary body is positively coupled to adenylyl cyclase and regulates chloride secretion [802]. *Xenopus* melanophores and chick brain express a distinct receptor (x420, P49219; c346, P49288, initially termed Mel_1C_) coupled to the G_i/o_ family of G proteins, for which GPR50 has recently been suggested to be a mammalian counterpart [451] although melatonin does not bind to GPR50 receptors.

### Further Reading

Cardinali DP *et al*. (2012) Melatonin and its analogs in insomnia and depression. *J. Pineal Res.* **52**: 365‐75 [PMID:21951153]

Dardente H. (2012) Melatonin‐dependent timing of seasonal reproduction by the pars tuberalis: pivotal roles for long daylengths and thyroid hormones. *J. Neuroendocrinol.* **24**: 249‐66 [PMID:22070540]

Dubocovich ML *et al*. (2010) International Union of Basic and Clinical Pharmacology. LXXV. Nomenclature, classification, and pharmacology of G protein‐coupled melatonin receptors. *Pharmacol. Rev.* **62**: 343‐80 [PMID:20605968]

Hickie IB *et al*. (2011) Novel melatonin‐based therapies: potential advances in the treatment of major depression. *Lancet* **378**: 621‐31 [PMID:21596429]

Korkmaz A *et al*. (2012) Gene regulation by melatonin linked to epigenetic phenomena. *Gene* **503**: 1‐11 [PMID:22569208]

Slominski A *et al*. (2008) Melatonin in the skin: synthesis, metabolism and functions. *Trends Endocrinol. Metab.* **19**: 17‐24 [PMID:18155917]

## Metabotropic glutamate receptors

### Overview

Metabotropic glutamate (mGlu) receptors (**nomenclature as agreed by the NC‐IUPHAR Subcommittee on Metabotropic Glutamate Receptors [1672]**) are activated by the endogenous ligands L‐glutamic acid, L‐serine‐O‐phosphate, N‐acetylaspartylglutamate (NAAG) and L‐cysteine sulphinic acid. Examples of agonists selective for mGlu receptors compared with ionotropic glutamate receptors are (1*S*,3*R*)‐ACPD and L‐CCG‐I, which show limited selectivity for Group‐II receptors. An example of an antagonist selective for mGlu receptors is LY341495, which blocks mGlu_2_ and mGlu_3_ at low nanomolar concentrations, mGlu_8_ at high nanomolar concentrations, and mGlu_4_, mGlu_5_, and mGlu_7_ in the micromolar range [955]. Three groups of native receptors are distinguishable on the bases of similarities of agonist pharmacology, primary sequence and G protein coupling to effector: Group‐I (mGlu_1_ and mGlu_5_), Group‐II (mGlu_2_ and mGlu_3_) and Group‐III (mGlu_4_, mGlu_6_, mGlu_7_ and mGlu_8_) (see Further reading). Group‐I mGlu receptors may be activated by 3,5‐DHPG and (*S*)‐3HPG[198] and antagonized by (S)‐hexylhomoibotenic acid[1171]. Group‐II mGlu receptors may be activated by LY389795[1311], LY379268[1311], eglumegad[1673, 2050], DCG‐IV and (2*R*,3*R*)‐APDC[1674], and antagonised by eGlu(4.3, [848] and LY307452[491, 2009]. Group‐III mGlu receptors may be activated by L‐AP4 and (*R,S*)‐4‐PPG[579].

In addition to orthosteric ligands that directly interact with the glutamate recognition site directly, allosteric modulators have been described. Negative allosteric modulators are listed separately. The positive allosteric modulators most often act as ‘potentiators’ of an orthosteric agonist response, without significantly activating the receptor in the absence of agonist.

Although mGlu receptors have been thought to only form homodimers, recent studies revealed the possible formation of heterodimers between either group‐I receptors, or within and between group‐II and ‐III receptors [441]. Although well characterized in transfected cells, co‐localization and specific pharmacological properties also suggest the existence of such heterodimers in the brain [2094].

The structure of the 7 transmembrane (TM) domains of both mGlu_1_ and mGlu_5_ have been solved, and confirm a general helical organization similar to that of other GPCRs, although the helices appear more compacted [438, 2048].

**Table:** 

Nomenclature r	mGlu_1_ receptor	mGlu_2_ receptor	mGlu_3_ receptor	mGlu_4_ receptor
HGNC, UniProt	*GRM1*, Q13255	*GRM2*, Q14416	*GRM3*, Q14832	*GRM4*, Q14833
Endogenous agonists	–	–	NAAG (Selective) (p*K*_i_ 4.7) [1679]	–
Agonists	–	–	–	L‐AP4 (pEC_50_ 6.5) [2050], L‐serine‐O‐phosphate (pEC_50_ 5.9) [2050]
Selective agonists	–	–	–	LSP4‐2022 (pEC_50_ 7) [631]
Antagonists	LY367385 (pIC_50_ 5.1) [349]	–	–	MAP4 (p*K*_i_ 4.6) [686] – Rat
Selective antagonists	3‐MATIDA (pIC_50_ 5.2) [1333] – Rat, (S)‐(+)‐CBPG (pIC_50_ 4.2) [1200] – Rat, (S)‐TBPG (pIC_50_ 4.2) [367] – Rat, AIDA (pA_2_ 4.2) [1334]	PCCG‐4 (pIC_50_ 5.1) [1483] – Rat	–	–
Allosteric modulators	YM298198 (Negative) (pIC_50_ 7.8) [979] – Rat	CBiPES (Positive) (pEC_50_ 7) [874], 4‐MPPTS (Positive) (pIC_50_ 5.8) [94, 873, 874, 1660]	–	SIB‐1893 (Positive) (pEC_50_ 6.3–6.8) [1217], MPEP (Positive) (pEC_50_ 6.3–6.6) [1217], PHCCC (Positive) (pEC_50_ 4.5) [1184]
Selective allosteric modulators	BAY 367620 (Negative) (p*K*_i_ 9.5) [267] – Rat, JNJ16259685 (Negative) (pIC_50_ 8.9) [1047], A‐841720 (Negative) (pIC_50_ 8) [2126], Ro67‐7476 (Positive) (p*K*_i_ 7.5–7.9) [971] – Rat, 3,5‐dimethyl PPP (Negative) (pIC_50_ 7.8) [1271] – Rat, EM‐TBPC (Negative) (p*K*_i_ 7.8) [1191] – Rat, Ro01‐6128 (Positive) (p*K*_i_ 7.5–7.7) [971] – Rat, LY456236 (Negative) (pIC_50_ 6.9) [1094], CPCCOEt (Negative) (pIC_50_ 5.2–5.8) [1116], Ro67‐4853 (Positive) (p*K*_i_ 5.1) [971] – Rat, PHCCC (Positive)	Ro64‐5229 (Negative) (pIC_50_ 7) [985] – Rat, biphenylindanone A (Positive) (pEC_50_ 7) [183]	–	VU0361737 (Positive) (pEC_50_ 6.6) [480], VU0155041 (Positive) (pEC_50_ 6.1) [1402]
Comments	–	–	–	pEC50 values for MPEP and SIB‐1893 were obtained in the presence of L‐AP4 [1217].

**Table:** 

Nomenclature	mGlu_5_ receptor	mGlu_6_ receptor	mGlu_7_ receptor	mGlu_8_ receptor
HGNC, UniProt	*GRM5*, P41594	*GRM6*, O15303	*GRM7*, Q14831	*GRM8*, O00222
Endogenous agonists	–	–	–	L‐serine‐O‐phosphate (pIC_50_ 6.2–7.2) [1192, 2050]
Agonists	–	–	LSP4‐2022 (pEC_50_ 4.9) [631], L‐serine‐O‐phosphate (pEC_50_ 4.5) [2050], L‐AP4 (pEC_50_ 3.8) [2050]	(*S*)‐3,4‐DCPG (pEC_50_ 7.5) [1876], L‐AP4 (pIC_50_ 7–7.2) [1192]
Selective agonists	(S)‐(+)‐CBPG (Partial agonist) (pEC_50_ 4.3) [1200] – Rat, CHPG (pIC_50_ 3.4) [1350]	1‐benzyl‐APDC (pEC_50_ 4.7) [1911] – Rat, homo‐AMPA (pEC_50_ 4.1) [237]	–	–
Antagonists	–	MAP4 (pIC_50_ 3.5) [1504] – Rat, THPG [1879] – Unknown	–	MPPG (pIC_50_ 4.3) [2050]
Selective antagonists	ACDPP (pIC_50_ 6.9) [182]	–	–	–
Allosteric modulators	3,3'‐difluorobenzaldazine (Positive) (pIC_50_ 5.6–8.5) [1415, 1416], alloswitch‐1 (Negative) (pIC_50_ 8.1) [1511] – Rat, CDPPB (Positive) (pEC_50_ 7.6–8) [956, 1114], MTEP (Negative) (p*K*_i_ 7.8) [223], MPEP (Negative) (pIC_50_ 7.4–7.7) [578, 580], fenobam (Negative) (pIC_50_ 7.2) [1519], SIB‐1893 (Negative) (pIC_50_ 5.9–6.5) [578, 1949], SIB‐1757 (Negative) (pIC_50_ 6–6.4) [578, 1949], CPPHA (Positive) (pIC_50_ 6.3) [1416]	–	MMPIP (Negative) (pIC_50_ 6.1–7.6) [1401, 1827] – Rat, AMN082 (Positive) (pEC_50_ 6.5–6.8) [1293], XAP044 (Negative) (pIC_50_ 5.6) [587]	–
Selective allosteric modulators	VU‐1545 (Positive) (pEC_50_ 8) [2142]	–	–	–

### Comments

The activity of NAAG as an agonist at mGlu_3_ receptors was questioned on the basis of contamination with glutamate [327, 547], but this has been refuted [1369].

Radioligand binding using a variety of radioligands has been conducted on recombinant receptors (for example, [^3^H]R214127[1046] and [^3^H]YM298198[979] at mGlu_1_ receptors and [^3^H]M‐MPEP[578] and [^3^H]methoxymethyl‐MTEP[47] at mGlu_5_ receptors. Although a number of radioligands have been used to examine binding in native tissues, correlation with individual subtypes is limited. Many pharmacological agents have not been fully tested across all known subtypes of mGlu receptors. Potential differences linked to the species (*e.g.* human *versus* rat or mouse) of the receptors and the receptor splice variants are generally not known. The influence of receptor expression level on pharmacology and selectivity has not been controlled for in most studies, particularly those involving functional assays of receptor coupling.

(S)‐(+)‐CBPG is an antagonist at mGlu_1_, but is an agonist (albeit of reduced efficacy) at mGlu_5_ receptors. DCG‐IV also exhibits agonist activity at NMDA glutamate receptors [1931], and is an antagonist at all group‐III mGluRs with an IC_50_ of 30*μ*M. A potential novel metabotropic glutamate receptor coupled to phosphoinositide turnover has been observed in rat brain; it is activated by 4‐methylhomoibotenic acid (ineffective as an agonist at recombinant Group I metabotropic glutamate receptors), but resistant to LY341495[341]. There are also reports of a distinct metabotropic glutamate receptor coupled to phospholipase D in rat brain, which does not readily fit into the current classification [964, 1482]

A related class C receptor composed of two distinct subunits, T1R1 + T1R3 is also activated by glutamate and is responsible for umami taste detection.

All selective antagonists at metabotropic glutamate receptors are competitive.

### Further Reading

Conn PJ *et al*. (1997) Pharmacology and functions of metabotropic glutamate receptors. *Annu. Rev. Pharmacol. Toxicol.* **37**: 205‐237 [PMID:9131252]

Ferraguti F *et al*. (2006) Metabotropic glutamate receptors. *Cell Tissue Res.* **326**: 483‐504 [PMID:16847639]

Nicoletti F *et al*. (2011) Metabotropic glutamate receptors: from the workbench to the bedside. *Neuropharmacology* **60**: 1017‐41 [PMID:21036182]

Niswender CM *et al*. (2010) Metabotropic glutamate receptors: physiology, pharmacology, and disease. *Annu. Rev. Pharmacol. Toxicol.* **50**: 295‐322 [PMID:20055706]

Rondard P *et al*. (2011) The complexity of their activation mechanism opens new possibilities for the modulation of mGlu and GABAB class C G protein‐coupled receptors. *Neuropharmacology* **60**: 82‐92 [PMID:20713070]

## Motilin receptor

### Overview

Motilin receptors (**provisional nomenclature**) are activated by a 22 amino‐acid peptide derived from a precursor (*MLN*, P12872), which may also generate a motilin‐associated peptide (*MLN*, P12872). These receptors are also suggested to be responsible for the gastrointestinal prokinetic effects of certain macrolide antibiotics (often called motilides; *e.g.* erythromycin), although for many of these molecules the evidence is sparse.

**Table:** 

Nomenclature	motilin receptor
HGNC, UniProt	*MLNR*, O43193
Endogenous agonists	motilin (*MLN*, P12872) (p*K*_i_ 8.4–8.7) [372, 1223, 1224, 1225]
Agonists	alemcinal (pIC_50_ 7.2) [1872], erythromycin‐A (pIC_50_ 5.5–6.5) [507, 1872], azithromycin (pEC_50_ 5.5) [220]
Selective agonists	camicinal (pEC_50_ 7.9) [99, 1639], mitemcinal (pEC_50_ 7.5–7.8) [977, 1845] – Rabbit
Selective antagonists	MA‐2029 (pA_2_ 9.2) [1811], GM‐109 (pIC_50_ 8) [701] – Pig
Labelled ligands	[^125^I]motilin (human) (Agonist) (p*K*_d_ 10) [507]

### Comments

In laboratory rodents, the gene encoding the motilin percursor appears to be absent, while the receptor appears to be a pseudogene [725, 1637]. Functions of motilin(*MLN*, P12872) are not usually detected in rodents, although brain and other responses to motilin and the macrolide alemcinal have been reported and the mechanism of these actions are obscure [1249, 1396]. Marked differences in ligand affinities for the motilin receptor in dogs and humans may be explained by significant differences in receptor structure [1638]. Note that for the complex macrolide structures, selectivity of action has often not been rigorously examined and other actions are possible (*e.g.* P2X inhibition by erythromycin; [2123]). Small molecule motilin receptor agonists are now described [1093, 1639, 2013]. The motilin receptor does not appear to have constitutive activity [774]. Although not proven, the existence of biased agonism at the receptor has been suggested [1225, 1292, 1636]. A truncated 5‐transmembrane structure has been identified but this is without activity when transfected into a host cell [507].

### Further Reading

De Smet B *et al*. (2009) Motilin and ghrelin as prokinetic drug targets. *Pharmacol. Ther.* **123**: 207‐23 [PMID:19427331]

Foord SM *et al*. (2005) International Union of Pharmacology. XLVI. G protein‐coupled receptor list. *Pharmacol Rev* **57**: 279‐288 [PMID:15914470]

Sanger GJ *et al*. (2012) Motilin: Toward a new understanding of the gastrointestinal neuropharmacology and therapeutic use of motilin receptor agonists. *Br. J. Pharmacol.* [PMID:23189978]

Takeshita E *et al*. (2006) Molecular characterization and distribution of motilin family receptors in the human gastrointestinal tract. *J Gastroenterol* **41**: 223‐230 [PMID:16699856]

## Neuromedin U receptors

### Overview

Neuromedin U receptors (**provisional nomenclature as recommended by NC‐IUPHAR[** **530** **]**) are activated by the endogenous 25 amino acid peptide neuromedin U (neuromedin U‐25 (*NMU*, P48645), NmU‐25), a peptide originally isolated from pig spinal cord [1287]. In humans, NmU‐25 appears to be the sole product of a precursor gene (*NMU*, P48645) showing a broad tissue distribution, but which is expressed at highest levels in the upper gastrointestinal tract, CNS, bone marrow and fetal liver. Much shorter versions of NmU are found in some species, but not in human, and are derived at least in some instances from the proteolytic cleavage of the longer NmU. Despite species differences in NmU structure, the C‐terminal region (particularly the C‐terminal pentapeptide) is highly conserved and contains biological activity. Neuromedin S (neuromedin S‐33 (*NMS*, Q5H8A3)) has also been identified as an endogenous agonist [1326]. NmS‐33 is, as its name suggests, a 33 amino‐acid product of a precursor protein derived from a single gene and contains an amidated C‐terminal heptapeptide identical to NmU. NmS‐33 appears to activate NMU receptors with equivalent potency to NmU‐25.

**Table:** 

Nomenclature	NMU1 receptor	NMU2 receptor
HGNC, UniProt	*NMUR1*, Q9HB89	*NMUR2*, Q9GZQ4
Antagonists	–	R‐PSOP (p*K*_B_ 7) [1128]

### Comments

NMU1 and NMU2 couple predominantly to G_q/11_ although there is evidence of good coupling to G_i/o_[213, 786, 794]. NMU1 and NMU2 can be labelled with [^125^I]‐NmU and [^125^I]‐NmS (of various species, *e.g.* [1259]), BODIPY^®;^ TMR‐NMU or Cy3B‐NMU‐8[213]. A range of radiolabelled (^125^I‐), fluorescently labelled (*e.g.* Cy3, Cy5, rhodamine and FAM) and biotin labelled versions of neuromedin U‐25(*NMU*, P48645) and neuromedin S‐33 (*NMS*, Q5H8A3) are now commercially available.

### Further Reading

Brighton PJ *et al*. (2004) Neuromedin U and its receptors: structure, function, and physiological roles. *Pharmacol. Rev.* **56**: 231‐48 [PMID:15169928]

Budhiraja S *et al*. (2009) Neuromedin U: physiology, pharmacology and therapeutic potential. *Fundam Clin Pharmacol* **23**: 149‐57 [PMID:19645813]

Foord SM *et al*. (2005) International Union of Pharmacology. XLVI. G protein‐coupled receptor list. *Pharmacol Rev* **57**: 279‐288 [PMID:15914470]

Mitchell JD *et al*. (2009) Emerging pharmacology and physiology of neuromedin U and the structurally related peptide neuromedin S. *Br. J. Pharmacol.* **158**: 87‐103 [PMID:19519756]

Novak CM. (2009) Neuromedin S and U. *Endocrinology* **150**: 2985‐7 [PMID:19549882]

## Neuropeptide FF/neuropeptide AF receptors

### Overview

The Neuropeptide FF receptor family contains two subtypes, NPFF1 and NPFF2 (**provisional nomenclature [** **530** **]**), which exhibit high affinities for neuropeptide FF (*NPFF*, O15130) and RFamide related peptides (RFRP: precursor gene symbol NPVF, Q9HCQ7). NPFF1 is broadly distributed in the central nervous system with the highest levels found in the limbic system and the hypothalamus. NPFF2 is present in high density in the superficial layers of the mammalian spinal cord where it is involved in nociception and modulation of opioid functions.

**Table:** 

Nomenclature	NPFF1 receptor	NPFF2 receptor
HGNC, UniProt	*NPFFR1*, Q9GZQ6	*NPFFR2*, Q9Y5X5
Rank order of potency	RFRP‐1 (*NPVF*, Q9HCQ7) >RFRP‐3 (*NPVF*, Q9HCQ7) > FMRFneuropeptide FF (*NPFF*, O15130) >neuropeptide AF (*NPFF*, O15130) >neuropeptide SF (*NPFF*, O15130), QRFP43 (*QRFP*, P83859), PrRP‐31 (*PRLH*, P81277) [628]	neuropeptide AF (*NPFF*, O15130), neuropeptide FF (*NPFF*, O15130) >PrRP‐31 (*PRLH*, P81277) > FMRF, QRFP43 (*QRFP*, P83859) >neuropeptide SF (*NPFF*, O15130) [628]
Endogenous agonists	neuropeptide FF (*NPFF*, O15130) (Selective) (p*K*_i_ 8.5–9.9) [628, 629, 1306], RFRP‐3 (*NPVF*, Q9HCQ7) (Selective) (p*K*_i_ 9.2–9.3) [629, 630, 1306]	neuropeptide FF (*NPFF*, O15130) (Selective) (p*K*_i_ 9.7) [629, 1305]
Selective agonists	–	dNPA (p*K*_i_ 10.6) [1607], AC263093 (pEC_50_ 5.2–5.9) [1036]
Antagonists	RF9 (p*K*_i_ 7.2) [1745]	–
Selective antagonists	AC262620 (p*K*_i_ 7.7–8.1) [1036], AC262970 (p*K*_i_ 7.4–8.1) [1036]	–
Labelled ligands	[^125^I]Y‐RFRP‐3 (Agonist) (p*K*_d_ 9.7) [629], [^3^H]NPVF (Agonist) (p*K*_d_ 8.6) [1855], [^125^I]NPFF (Agonist) [628]	[^125^I]EYF (Agonist) (p*K*_d_ 10.2) [1306], [^3^H]EYF (Agonist) (p*K*_d_ 9.3) [1855], [^125^I]NPFF (Agonist) [628]

### Comments

An orphan receptor *GPR83*(Q9NYM4) shows sequence similarities with NPFF1, NPFF2, PrRP and QRFP receptors. The antagonist RF9 is selective for NPFF receptors, but does not distinguish between the NPFF1 and NPFF2 subtypes (p*K*_i_ 7.1 and 7.2, respectively, [1745]).

### Further Reading

Moulédous L *et al*. (2010) Opioid‐modulating properties of the neuropeptide FF system. *Biofactors* **36**: 423‐9 [PMID:20803521]

Vyas N *et al*. (2006) Structure‐activity relationships of neuropeptide FF and related peptidic and non‐peptidic derivatives. *Peptides* **27**: 990‐6 [PMID:16490282]

Yang HY *et al*. (2008) Modulatory role of neuropeptide FF system in nociception and opiate analgesia. *Neuropeptides* **42**: 1‐18 [PMID:17854890]

## Neuropeptide S receptor

### Overview

The neuropeptide S receptor (NPS, **provisional nomenclature [** **530** **]**) responds to the 20 amino‐acid peptide neuropeptide S derived from the precursor (*NPS*, P0C0P6).

**Table:** 

Nomenclature	NPS receptor
HGNC, UniProt	*NPSR1*, Q6W5P4
Endogenous agonists	neuropeptide S (*NPS*, P0C0P6) (pEC_50_ 8) [2070]
Labelled ligands	[^125^I]Tyr^10^NPS (human) (Agonist) (p*K*_d_ 9.5) [2070]

### Comments

Polymorphisms in the NPS receptor have been suggested to be associated with asthma [1953] and irritable bowel syndrome [386].

### Further Reading

Cannella N *et al*. (2013) The role of the neuropeptide S system in addiction: focus on its interaction with the CRF and hypocretin/orexin neurotransmission. *Prog. Neurobiol.* **100**: 48‐59 [PMID:23041581]

Dal Ben D *et al*. (2011) Neuropeptide S receptor: recent updates on nonpeptide antagonist discovery. *ChemMedChem* **6**: 1163‐71 [PMID:21452188]

Foord SM *et al*. (2005) International Union of Pharmacology. XLVI. G protein‐coupled receptor list. *Pharmacol Rev* **57**: 279‐288 [PMID:15914470]

Guerrini R *et al*. (2010) Neurobiology, pharmacology, and medicinal chemistry of neuropeptide S and its receptor. *Med Res Rev* **30**: 751‐77 [PMID:19824051]

Pape HC *et al*. (2010) Neuropeptide S: a transmitter system in the brain regulating fear and anxiety. *Neuropharmacology* **58**: 29‐34 [PMID:19523478]

Reinscheid RK. (2008) Neuropeptide S: anatomy, pharmacology, genetics and physiological functions. *Results Probl Cell Differ* **46**: 145‐58 [PMID:18204825]

## Neuropeptide W/neuropeptide B receptors

### Overview

The neuropeptide BW receptor 1 (NPBW1, **provisional nomenclature [** **530** **]**) is activated by two 23‐amino‐acid peptides, neuropeptide W (neuropeptide W‐23 (*NPW*, Q8N729)) and neuropeptide B (neuropeptide B‐23 (*NPB*, Q8NG41)) [554, 1725]. C‐terminally extended forms of the peptides (neuropeptide W‐30(*NPW*, Q8N729) and neuropeptide B‐29 (*NPB*, Q8NG41)) also activate NPBW1 [211]. Unique to both forms of neuropeptide B is the N‐terminal bromination of the first tryptophan residue, and it is from this post‐translational modification that the nomenclature NPB is derived. These peptides were first identified from bovine hypothalamus and therefore are classed as neuropeptides. Endogenous variants of the peptides without the N‐terminal bromination, des‐Br‐neuropeptide B‐23(*NPB*, Q8NG41) and des‐Br‐neuropeptide B‐29 (*NPB*, Q8NG41), were not found to be major components of bovine hypothalamic tissue extracts. The NPBW2 receptor is activated by the short and C‐terminal extended forms of neuropeptide W and neuropeptide B [211].

**Table:** 

Nomenclature	NPBW1 receptor	NPBW2 receptor
HGNC, UniProt	*NPBWR1*, P48145	*NPBWR2*, P48146
Rank order of potency	neuropeptide B‐29 (*NPB*, Q8NG41) >neuropeptide B‐23 (*NPB*, Q8NG41) >neuropeptide W‐23 (*NPW*, Q8N729) >neuropeptide W‐30 (*NPW*, Q8N729) [211]	neuropeptide W‐23 (*NPW*, Q8N729) >neuropeptide W‐30 (*NPW*, Q8N729) >neuropeptide B‐29 (*NPB*, Q8NG41) >neuropeptide B‐23 (*NPB*, Q8NG41) [211]
Selective agonists	Ava3 (p*K*_i_ 9.4–9.4) [902], Ava5 (p*K*_i_ 8.8–9) [902]	–
Labelled ligands	[^125^I]NPW‐23 (human) (Agonist) (p*K*_d_ 9.4) [1747]	[^125^I]NPW‐23 (human) (Agonist) (p*K*_d_ 7.7) [1725]

### Comments

Potency measurements were conducted with heterologously‐expressed receptors with a range of 0.14‐0.57 nM (NPBW1) and 0.98‐21 nM (NPBW2).

NPBW1^‐/‐^ mice show changes in social behavior, suggesting that the NPBW1 pathway may have an important role in the emotional responses of social interaction [1355].

### Further Reading

Date Y *et al*. (2010) Neuropeptide W: an anorectic peptide regulated by leptin and metabolic state. *Endocrinology* **151**: 2200‐10 [PMID:20189998]

Hondo M *et al*. (2008) The NPB/NPW neuropeptide system and its role in regulating energy homeostasis, pain, and emotion. *Results Probl Cell Differ* **46**: 239‐56 [PMID:18204824]

Sakurai T. (2013) NPBWR1 and NPBWR2: Implications in Energy Homeostasis, Pain, and Emotion. *Front Endocrinol (Lausanne)* **4**: 23 [PMID:23515889]

Singh G *et al*. (2006) Neuropeptide B and W: neurotransmitters in an emerging G‐protein‐coupled receptor system. *Br. J. Pharmacol.* **148**: 1033‐41 [PMID:16847439]

## Neuropeptide Y receptors

### Overview

Neuropeptide Y (NPY) receptors (**nomenclature as agreed by the NC‐IUPHAR Subcommittee on Neuropeptide Y Receptors [** **1270** **]**) are activated by the endogenous peptides neuropeptide Y (*NPY*, P01303), neuropeptide Y‐(3‐36), peptide YY(*PYY*, P10082), PYY‐(3‐36) and pancreatic polypeptide (*PPY*, P01298) (PP). The receptor originally identified as the Y3 receptor has been identified as the CXCR4 chemokine recepter (originally named LESTR, [1135]). The y6 receptor is a functional gene product in mouse, absent in rat, but contains a frame‐shift mutation in primates producing a truncated non‐functional gene [642]. Many of the agonists exhibit differing degrees of selectivity dependent on the species examined. For example, the potency of PP is greater at the rat Y_4_receptor than at the human receptor [485]. In addition, many agonists lack selectivity for individual subtypes, but can exhibit comparable potency against pairs of NPY receptor subtypes, or have not been examined for activity at all subtypes. [^125^I]‐PYY or [^125^I]‐NPY can be used to label Y_1_, Y_2_, Y_5_ and y_6_ subtypes non‐selectively, while [^125^I][cPP(1‐7), NPY(19‐23), Ala^31^, Aib^32^, Gln^34^]hPP may be used to label Y_5_ receptors preferentially (note that cPP denotes chicken peptide sequence and hPP is the human sequence).

**Table:** 

Nomenclature	Y_1_ receptor	Y_2_ receptor	Y_4_ receptor	Y_5_ receptor	y_6_ receptor
HGNC, UniProt	*NPY1R*, P25929	*NPY2R*, P49146	*NPY4R*, P50391	*NPY5R*, Q15761	*NPY6R*, Q99463
Rank order of potency	neuropeptide Y > peptide YY ≫ pancreatic polypeptide	neuropeptide Y > peptide YY ≫ pancreatic polypeptide	pancreatic polypeptide > neuropeptide Y = peptide YY	neuropeptide Y > peptide YY > pancreatic polypeptide	neuropeptide Y = peptide YY > pancreatic polypeptide
Endogenous agonists	neuropeptide Y (*NPY*, P01303), peptide YY (*PYY*, P10082)	PYY‐(3‐36) (*PYY*, P10082) (p*K*_i_ 9.2–9.7) [588, 599], neuropeptide Y (*NPY*, P01303), neuropeptide Y‐(3‐36) (*NPY*, P01303), peptide YY (*PYY*, P10082)	pancreatic polypeptide (*PPY*, P01298) (p*K*_i_ 8.7–10.9) [92, 1152, 1899, 2076]	–	–
Selective agonists	[Leu^31^,Pro^34^]NPY (pEC_50_ 7.1) [378], [Leu^31^,Pro^34^]PYY (human), [Pro^34^]NPY, [Pro^34^]PYY (human)	–	–	[Ala^31^,Aib^32^]NPY (pig) (pIC_50_ 8.2) [254]	–
Selective antagonists	BIBO3304 (pIC_50_ 9.5) [2020], BIBP3226 (p*K*_i_ 8.1–9.3) [436, 2021]	BIIE0246 (pIC_50_ 8.5) [434], JNJ‐5207787 (pIC_50_ 6.9–7.1) [178]	–	L‐152,804 (p*K*_i_ 7.6) [901]	–
Labelled ligands	[^3^H]BIBP3226 (Antagonist) (p*K*_d_ 8.7), [^125^I][Leu^31^,Pro^34^]NPY (Agonist)	[^125^I]PYY‐(3‐36) (human) (Agonist)	[^125^I]PP (human) (Agonist)	[^125^I][cPP(1‐7), NPY(19‐23), Ala^31^, Aib^32^, Gln^34^]hPP (Agonist) (p*K*_d_ 9.2–9.3) [453] – Rat	–
Comments	Note that Pro^34^‐containing NPY and PYY can also bind Y_4_ and Y_5_ receptors, so strictly speaking are not selective, but are the 'preferred' agonists.	–	–	–	The y_6_ receptor is a pseudogene in humans, but is functional in mouse, rabbit and some other mammals.

### Comments

The Y_1_ agonists indicated are selective relative to Y_2_ receptors. BIBP3226 is selective relative to Y_2_, Y_4_ and Y_5_ receptors [598]. NPY‐(13‐36) is Y_2_ selective relative to Y_1_ and Y_5_ receptors. PYY‐(3‐36) is Y_2_ selective relative to Y_1_ receptors.

### Further Reading

Bowers ME *et al*. (2012) Neuropeptide regulation of fear and anxiety: Implications of cholecystokinin, endogenous opioids, and neuropeptide Y. *Physiol. Behav.* **107**: 699‐710 [PMID:22429904]

Decressac M *et al*. (2012) Neuropeptide Y and its role in CNS disease and repair. *Exp. Neurol.* **238**: 265‐72 [PMID:23022456]

Michel MC *et al*. (1998) XVI. International Union of Pharmacology recommendations for the nomenclature of neuropeptide Y, peptide YY and pancreatic polypeptide receptors. *Pharmacol. Rev.* **50**: 143‐150 [PMID:9549761]

Morales‐Medina JC *et al*. (2010) A possible role of neuropeptide Y in depression and stress. *Brain Res.* **1314**: 194‐205 [PMID:19782662]

Zengin A *et al*. (2010) Neuropeptide Y and sex hormone interactions in humoral and neuronal regulation of bone and fat. *Trends Endocrinol. Metab.* **21**: 411‐8 [PMID:20202858]

Zhang L *et al*. (2011) The neuropeptide Y system: pathophysiological and therapeutic implications in obesity and cancer. *Pharmacol. Ther.* **131**: 91‐113 [PMID:21439311]

## Neurotensin receptors

### Overview

Neurotensin receptors (**nomenclature as recommended by NC‐IUPHAR[** **530** **]**) are activated by the endogenous tridecapeptide neurotensin (pGlu‐Leu‐Tyr‐Glu‐Asn‐Lys‐Pro‐Arg‐Arg‐Pro‐Tyr‐Ile‐Leu) derived from a precursor (*NTS*, 30990), which also generates neuromedin N, an agonist at the NTS_2_ receptor. A nonpeptide antagonist, SR142948A, shows high affinity (pK_i_~9) at both NTS_1_ and NTS_2_ receptors [664]. [^3^H]neurotensin (human, mouse, rat) and [^125^I]neurotensin (human, mouse, rat) may be used to label NTS_1_ and NTS_2_ receptors at 0.1‐0.3 and 3‐5 nM concentrations respectively.

**Table:** 

Nomenclature	NTS_1_ receptor	NTS_2_ receptor
HGNC, UniProt	*NTSR1*, P30989	*NTSR2*, O95665
Rank order of potency	neurotensin (*NTS*, P30990) >neuromedin N {Mouse, Rat} [741]	neurotensin (*NTS*, P30990) = neuromedin N {Mouse, Rat} [1235]
Selective agonists	JMV449 (p*K*_i_ 10) [1753] – Rat	levocabastine (p*K*_i_ 6.8) [1235, 1583]
Antagonists	meclinertant (pIC_50_ 7.5–8.2) [664]	–
Labelled ligands	[^3^H]meclinertant (Antagonist) (p*K*_d_ 8.5) [1030] – Rat	–
Comments	–	A splice variant of the NTS_2_ receptor bearing 5 transmembrane domains has been identified in mouse [191] and later in rat [1492].

### Comments

neurotensin(*NTS*, P30990) appears to be a low‐efficacy agonist at the NTS_2_ receptor [1959], while the NTS_1_ receptor antagonist meclinertant is an agonist at NTS_2_ receptors [1959]. An additional protein, provisionally termed NTS_3_ (also known as NTR3, gp95 and sortilin; ENSG00000134243), has been suggested to bind lipoprotein lipase and mediate its degradation [1395]. It has been reported to interact with the NTS_1_ receptor [1211] and has been implicated in hormone trafficking and/or neurotensin uptake.

### Further Reading

Boules M *et al*. (2013) Diverse roles of neurotensin agonists in the central nervous system. *Front Endocrinol (Lausanne)* **4**: 36 [PMID:23526754]

Dupouy S *et al*. (2011) The potential use of the neurotensin high affinity receptor 1 as a biomarker for cancer progression and as a component of personalized medicine in selective cancers. *Biochimie* **93**: 1369‐78 [PMID:21605619]

Foord SM *et al*. (2005) International Union of Pharmacology. XLVI. G protein‐coupled receptor list. *Pharmacol Rev* **57**: 279‐288 [PMID:15914470]

Kalafatakis K *et al*. (2011) Contribution of neurotensin in the immune and neuroendocrine modulation of normal and abnormal enteric function. *Regul. Pept.* **170**: 7‐17 [PMID:21549161]

Mazella J *et al*. (2012) Neurotensin and its receptors in the control of glucose homeostasis. *Front Endocrinol (Lausanne)* **3**: 143 [PMID:23230428]

Myers RM *et al*. (2009) Cancer, chemistry, and the cell: molecules that interact with the neurotensin receptors. *ACS Chem. Biol.* **4**: 503‐25 [PMID:19462983]

## Opioid receptors

### Overview

Opioid and opioid‐like receptors are activated by a variety of endogenous peptides including [Met]enkephalin(*PENK*, P01210) (met), [Leu]enkephalin(*PENK*, P01210) (leu), *β*‐endorphin(*POMC*, P01189) (*β*‐end), *α*‐neodynorphin(*PDYN*, P01213), dynorphin A(*PDYN*, P01213) (dynA), dynorphin B(*PDYN*, P01213) (dynB), big dynorphin(*PDYN*, P01213) (Big dyn), nociceptin/orphanin FQ(*PNOC*, Q13519) (N/OFQ); endomorphin‐1 and endomorphin‐2 are also potential endogenous peptides. The Greek letter nomenclature for the opioid receptors, *μ*, *δ* and *κ*, is well established, and **NC‐IUPHAR** considers this nomenclature most appropriate [376, 417, 530]. The human N/OFQ receptor is considered 'opioid‐related' rather than opioid because while it exhibits a high degree of structural homology with the conventional opioid receptors [1308], it displays a distinct pharmacology.

**Table:** 

Nomenclature	*δ* receptor	*κ* receptor	*μ* receptor	NOP receptor
HGNC, UniProt	*OPRD1*, P41143	*OPRK1*, P41145	*OPRM1*, P35372	*OPRL1*, P41146
Principal endogenous agonists	*β*‐endorphin (*POMC*, P01189), [Leu]enkephalin (*PENK*, P01210), [Met]enkephalin (*PENK*, P01210)	big dynorphin (*PDYN*, P01213), dynorphin A (*PDYN*, P01213)	*β*‐endorphin (*POMC*, P01189), [Met]enkephalin (*PENK*, P01210), [Leu]enkephalin (*PENK*, P01210), endomorphin‐1, endomorphin‐2	–
Endogenous agonists	–	–	endomorphin‐2 (Selective) (p*K*_i_ 8.5) [2109] – Rat, endomorphin‐1 (Selective) (p*K*_i_ 8.3) [622, 2109]	nociceptin/orphanin FQ (*PNOC*, Q13519) (Selective) (p*K*_i_ 9.7–10.4) [149, 1242, 1303, 1307, 1439]
Agonists	–	–	levorphanol (pIC_50_ 9.9) [692], hydromorphone (p*K*_i_ 9.6) [2007], fentanyl (p*K*_i_ 9.2) [1893], buprenorphine (Partial agonist) (p*K*_i_ 8.8) [1893], methadone (pIC_50_ 8.4) [1523], codeine (p*K*_i_ 6.9) [1893], tapentadol (p*K*_i_ 6.8) [1916], meperidine (pIC_50_ 6.5) [1523]	–
Selective agonists	[D‐Ala^2^]deltorphin I (p*K*_d_ 9.4) [487, 1795], DPDPE (p*K*_i_ 8.8) [1337, 1893], [D‐Ala^2^]deltorphin II (p*K*_i_ 8.8) [488], SNC80 (p*K*_i_ 7.2) [258, 1549]	U50488 (p*K*_i_ 7.8–9.7) [297, 1478, 1744, 1893, 1962, 2128, 2130], enadoline (p*K*_i_ 9.6) [808, 1383], U69593 (p*K*_i_ 9.5) [1034, 1893], salvinorin A (p*K*_i_ 7.8–8.7) [251, 1603]	sufentanil (p*K*_i_ 9.9) [1960], DAMGO (p*K*_i_ 9.3) [691, 1893], loperamide (p*K*_i_ 9.3) [308], morphine (p*K*_i_ 9) [620, 1893], PL017 (p*K*_i_ 8.2) [290, 1893]	N/OFQ‐(1‐13)‐NH_2_ (p*K*_i_ 10.1–10.4) [149, 661, 1242, 1439], Ro64‐6198 (p*K*_i_ 9.6) [855]
Antagonists	naltrexone (p*K*_i_ 8) [1893], naloxone (p*K*_i_ 7.2) [1893]	buprenorphine (p*K*_i_ 9.1–10.2) [1893, 2130], nalmefene (p*K*_i_ 9.5) [1893], naltrexone (p*K*_i_ 8.4–9.4) [1478, 1744, 1893], naloxone (p*K*_i_ 7.6–8.6) [1478, 1744, 1893, 2128, 2130]	naltrexone (p*K*_i_ 9.7) [1893], nalmefene (p*K*_i_ 9.5) [1893], nalorphine (p*K*_i_ 8.9) [1893], methylnaltrexone (p*K*_i_ 8.7) [2007]	–
Selective antagonists	naltriben (p*K*_i_ 10) [1773, 1893], naltrindole (p*K*_i_ 9.7) [1521, 1893], TIPP*ψ* (Inverse agonist) (p*K*_i_ 9) [1664, 1893]	nor‐binaltorphimine (p*K*_i_ 8.9–11) [1478, 1520, 1744, 1893, 2128, 2130], 5'‐guanidinonaltrindole (p*K*_i_ 9.7–9.9) [882, 1478, 1797]	alvimopan (p*K*_i_ 9.3) [1056], levallorphan (p*K*_i_ 8.8–9.3) [1187], CTAP (p*K*_i_ 8.6) [290, 1893]	UFP‐101 (p*K*_i_ 10.2) [259], Banyu Compound‐24 (p*K*_i_ 9.6) [523], SB 612111 (p*K*_i_ 9.5) [2112], J‐113397 (pIC_50_ 8.3) [919]
Labelled ligands	[^3^H]naltrindole (Antagonist) (p*K*_d_ 10.4) [2072] – Rat, [^3^H]DPDPE (Agonist) [26], [^3^H]deltorphin II (Agonist) [252], [^3^H]naltriben (Antagonist) [1088]	[^3^H]U69593 (Agonist) (p*K*_d_ 8.7–8.8) [1034, 1478, 1744], [^3^H]enadoline (Agonist) [1746]	[^3^H]DAMGO (Agonist) (p*K*_d_ 9.2) [1567] – Rat, [^3^H]PL017 (Agonist) [717] – Rat	[^3^H]N/OFQ (Agonist) (p*K*_d_ 10.2) [437, 1307]

### Comments

Three naloxone‐sensitive opioid receptor genes have been identified in humans, and while the *μ*‐receptor in particular may be subject to extensive alternative splicing [1468], these putative isoforms have not been correlated with any of the subtypes of receptor proposed in years past. Opioid receptors may heterodimerize with each other or with other 7TM receptors [884], and give rise to complexes with a unique pharmacology, however, evidence for such heterodimers in native cells is equivocal and the consequences this heterodimerization for signalling remains largely unknown. For *μ*‐opioid receptors at least, dimerization does not seem to be required for signalling [1026]. A distinct met‐enkephalin receptor lacking structural resemblance to the opioid receptors listed has been identified (*OGFR*, 9NZT2) and termed an opioid growth factor receptor [2110].

Endomorphin‐1 and endomorphin‐2 have been identified as highly selective, putative endogenous agonists for the *μ*‐opioid receptor. At present, however, the mechanisms for endomorphin synthesis *in vivo* have not been established, and there is no gene identified that encodes for either. Thus, the status of these peptides as endogenous ligands remains unproven.

Two areas of increasing importance in defining opioid receptor function are the presence of functionally relevant single nucleotide polymorphisms in human *μ*‐receptors [1423] and the identification of biased signalling by opioid receptor ligands, in particular, compounds previously characterized as antagonists [231]. Pathway bias for agonists makes general rank orders of potency and efficacy somewhat obsolete, so these do not appear in the table. As ever, the mechanisms underlying the acute and long term regulation of opiod receptor function are the subject of intense investigation and debate.

The richness of opioid receptor pharmacology has been enhanced with the recent discovery of allosteric modulators of MOPr and DOPr, notably the positive allosteric modulators and silent allosteric "antagonists" outlined in [240, 241]. Negative allosteric modulation of opioid receptors has been previously suggested [908], whether all compounds are acting at a similar site remains to be established.

### Further Reading

Butelman ER *et al*. (2012) *κ*‐opioid receptor/dynorphin system: genetic and pharmacotherapeutic implications for addiction. *Trends Neurosci.* **35**: 587‐96 [PMID:22709632]

Cox BM *et al*. (2015) Challenges for opioid receptor nomenclature: IUPHAR Review 9. *Br. J. Pharmacol.* **172**: 317‐23 [PMID:24528283]

Kelly E. (2011) The subtleties of *μ*‐opioid receptor phosphorylation. *Br. J. Pharmacol.* **164**: 294‐7 [PMID:21449916]

Pradhan AA *et al*. (2011) The delta opioid receptor: an evolving target for the treatment of brain disorders. *Trends Pharmacol. Sci.* **32**: 581‐90 [PMID:21925742]

Schröder W *et al*. (2014) Functional plasticity of the N/OFQ‐NOP receptor system determines analgesic properties of NOP receptor agonists. *Br. J. Pharmacol.* **171**: 3777‐800 [PMID:24762001]

Williams JT *et al*. (2013) Regulation of *μ*‐opioid receptors: desensitization, phosphorylation, internalization, and tolerance. *Pharmacol. Rev.* **65**: 223‐54 [PMID:23321159]

## Orexin receptors

### Overview

Orexin receptors (**nomenclature as agreed by the NC‐IUPHAR Subcommittee on Orexin receptors [** **627** **]**) are activated by the endogenous polypeptides orexin‐A(*HCRT*, O43612) and orexin‐B(*HCRT*, O43612) (also known as hypocretin‐1 and ‐2; 33 and 28 aa) derived from a common precursor, preproorexin or orexin precursor, by proteolytic cleavage [1629]. Binding to both receptors may be accomplished with [^125^I]orexin A (human, mouse, rat) [773].

**Table:** 

Nomenclature	OX_1_ receptor	OX_2_ receptor
HGNC, UniProt	*HCRTR1*, O43613	*HCRTR2*, O43614
Rank order of potency	orexin‐A (*HCRT*, O43612) >orexin‐B (*HCRT*, O43612)	orexin‐A (*HCRT*, O43612) = orexin‐B (*HCRT*, O43612)
Selective agonists	–	[Ala^11^, D‐Leu^15^]orexin‐B (pEC_50_ 9.9) [62]
(Sub)family‐selective antagonists	suvorexant (p*K*_i_ 9.3) [377], SB‐649868 (p*K*_i_ 9.1) [419], filorexant (p*K*_i_ 8.6) [2035], almorexant (pIC_50_ 7.9) [216]	filorexant (p*K*_i_ 9.5) [2035], suvorexant (p*K*_i_ 9.5) [377], SB‐649868 (p*K*_i_ 8.9) [419], almorexant (pIC_50_ 8.1) [216]
Selective antagonists	SB‐408124 (p*K*_i_ 7.2–7.6) [1042, 1190], SB‐334867 (p*K*_i_ 7.4–7.5) [1190, 1518]	EMPA (p*K*_i_ 9) [1189], JNJ 10397049 (p*K*_i_ 7.9–8.6) [1238], TCS‐OX2‐29 (p*K*_i_ 7.4) [760]
Labelled ligands	[^3^H]SB‐674042 (Antagonist) (p*K*_d_ 8.3–9.1) [1042, 1190, 1193]	–

### Comments

The primary coupling of orexin receptors to G_q/11_ proteins is rather speculative and based on the strong activation of phospholipase C. Coupling of both receptors to G_i/o_ and G_s_ has also been reported [1019, 1555]; for most cellular responses observed, the G protein pathway is unknown. The rank order of endogenous agonist potency may depend on the cellular signal transduction machinery. The synthetic [Ala^11^, D‐Leu^15^]orexin‐B may show poor OX_2_ receptor selectivity [1540].

Loss‐of‐function mutations in the gene encoding the OX_2_ receptor underlie canine hereditary narcolepsy [1111].

### Further Reading

Boss C. (2014) Orexin receptor antagonists–a patent review (2010 to August 2014). *Expert Opin Ther Pat* **24**: 1367‐81 [PMID:25407283]

Boss C *et al*. (2009) Biomedical application of orexin/hypocretin receptor ligands in neuroscience. *J. Med. Chem.* **52**: 891‐903 [PMID:19199652]

Christopher JA. (2014) Small‐molecule antagonists of the orexin receptors. *Pharm Pat Anal* **3**: 625‐38 [PMID:25489915]

Gotter AL *et al*. (2012) International Union of Basic and Clinical Pharmacology. LXXXVI. Orexin receptor function, nomenclature and pharmacology. *Pharmacol. Rev.* **64**: 389‐420 [PMID:22759794]

Lebold TP *et al*. (2013) Selective orexin receptor antagonists. *Bioorg. Med. Chem. Lett.* **23**: 4761‐9 [PMID:23891187]

Mieda M *et al*. (2013) Orexin (hypocretin) receptor agonists and antagonists for treatment of sleep disorders. Rationale for development and current status. *CNS Drugs* **27**: 83‐90 [PMID:23359095]

## Oxoglutarate receptor

### Overview

**Nomenclature as recommended by NC‐IUPHAR[** **396** **]**.

**Table:** 

Nomenclature	oxoglutarate receptor
HGNC, UniProt	*OXGR1*, Q96P68
Endogenous agonists	*α*‐ketoglutaric acid (pEC_50_ 3.3–4.5) [728, 1785]

## P2Y receptors

### Overview

P2Y receptors (**nomenclature as agreed by the NC‐IUPHAR Subcommittee on P2Y Receptors [** **1** **,** **2** **]**) are activated by the endogenous ligands ATP, adenosine diphosphate, uridine triphosphate, uridine diphosphate and UDP‐glucose. The relationship of many of the cloned receptors to endogenously expressed receptors is not yet established and so it might be appropriate to use wording such as ‘uridine triphosphate‐preferring (or ATP‐, *etc*.) P2Y receptor’ or ‘P2Y_1_‐like’, *etc*., until further, as yet undefined, corroborative criteria can be applied [244, 486, 837, 2003, 2146].

**Table:** 

Nomenclature	P2Y_1_ receptor	P2Y_2_ receptor	P2Y_4_ receptor	P2Y_6_ receptor
HGNC, UniProt	*P2RY1*, P47900	*P2RY2*, P41231	*P2RY4*, P51582	*P2RY6*, Q15077
Rank order of potency	adenosine diphosphate>ATP	uridine triphosphate=ATP	uridine triphosphate>ATP (at rat recombinant receptors, UTP = ATP)	uridine diphosphate≫uridine triphosphate>ATP
Endogenous agonists	–	–	–	uridine diphosphate (Selective) (pEC_50_ 6.5) [359]
Agonists	ADP*β*S (pEC_50_ 7.3) [1848], 2MeSADP (pIC_50_ 5.4–7) [1658, 1970]	–	–	Rp‐5‐OMe‐UDP*α*B (pEC_50_ 8.1) [611, 666]
Selective agonists	MRS2365 (pEC_50_ 9.4) [314], 2‐Cl‐ADP(*α*‐BH_3_) (pEC_50_ 8.1) [73]	2‐thioUTP (pEC_50_ 7.3) [470], PSB1114 (EC_50_ value determined using an IP_3_ functional assay) (pEC_50_ 6.9) [471], Ap_4_A (pEC_50_ 6.1) [270, 1471], UTP*γ*S (pEC_50_ 5.8) [1054], MRS2768 (EC_50_ value determined using an IP_3_ functional assay) (pEC_50_ 5.7) [973]	MRS4062 (pEC_50_ 7.6) [1213], UTP*γ*S [1055] – Unknown	MRS2957 (pEC_50_ 7.9) [1212], MRS2693 (pEC_50_ 7.8) [143], 3‐phenacyl‐UDP (pEC_50_ 7.2) [470]
Antagonists	–	–	ATP (p*K*_d_ 6.2) [925]	–
Selective antagonists	MRS2500 (p*K*_i_ 8.8–9.1) [274, 942], BMS compound 16 [PMID:23368907] (p*K*_i_ 8.2) [295, 2115], MRS2279 (p*K*_i_ 7.9) [1970], MRS2179 (p*K*_i_ 7–7.1) [197, 1970], 2,2'‐pyridylisatogen tosylate (p*K*_i_ 6.8) [570]	AR‐C118925XX (pIC_50_∼6) [924]	–	MRS2578 (pIC_50_ 7.4) [1196]
Labelled ligands	[^3^H]MRS2279 (Antagonist) (p*K*_d_ 8.1) [1970], [^3^H]2MeSADP (Agonist) (p*K*_d_ 7.3) [1848], [^35^S]ADP*β*S (Agonist) – Unknown	–	–	–

**Table:** 

Nomenclature	P2Y_11_ receptor	P2Y_12_ receptor	P2Y_13_ receptor	P2Y_14_ receptor
HGNC, UniProt	*P2RY11*, Q96G91	*P2RY12*, Q9H244	*P2RY13*, Q9BPV8	*P2RY14*, Q15391
Rank order of potency	ATP>uridine triphosphate	–	adenosine diphosphate≫ATP	–
Rank order of potency Human	–	–	–	uridine diphosphate≥UDP‐glucose
Endogenous agonists	–	adenosine diphosphate (Selective) (p*K*_i_ 5.9) [740]	–	–
Agonists	–	2MeSADP (p*K*_i_ 9.2) [740]	–	MRS2690 (pEC_50_ 6.6–7.3) [571, 974]
Selective agonists	AR‐C67085 (pEC_50_ 8.5) [88, 360], NF546 (pEC_50_ 6.3) [1255], NAADP [1322], NAD [1323]	–	–	–
Antagonists	NF340 (pIC_50_ 6.4–7.1) [1255]	PSB‐0739 (p*K*_i_ 7.6) [91]	–	–
Selective antagonists	NF157 (p*K*_i_ 7.3) [1923]	AZD1283 (p*K*_i_ 8) [76, 2116], ARL66096 (pIC_50_ 7.9) [806, 807], ticagrelor (p*K*_i_ 7.8) [2113]	MRS2211 (pIC_50_ 6) [950]	PPTN (p*K*_i_ 10.1) [96]
Labelled ligands	–	[^3^H]2MeSADP (Agonist) (pIC_50_ 7.5–9.6) [1848], [^3^H]PSB‐0413 (Antagonist) (p*K*_d_ 8.3–8.5) [469, 1431]	–	–

### Comments

cangrelor shows selectivity for P2Y_12_ and P2Y_13_ receptors compared with other P2Y receptors [1209, 1848]. NF157 also has antagonist activity at P2X_1_ receptors [1923]. Uridine diphosphate has been reported to be an antagonist at the P2Y_14_ receptor [548]. [^35^S]ATP*α*S has been used to label P2Y receptors in rat synaptosomal membranes [1682, 1683].

An orphan GPCR suggested to be a ‘P2Y_15_’ receptor [823] appears not to be a genuine nucleotide receptor [2], but rather responds to dicarboxylic acids [728]. Further P2Y‐like receptors have been cloned from non‐mammalian sources; a clone from chick brain, termed a p2y_3_ receptor (ENSGALG00000017327), couples to the G_q/11_ family of G proteins and shows the rank order of potency adenosine diphosphate>uridine triphosphate>ATP = uridine diphosphate [1998]. In addition, human sources have yielded a clone with a preliminary identification of p2y5 (*LPAR6*, P43657) and contradictory evidence of responses to ATP[954, 1999]. This protein is now classified as LPA_6_, a receptor for lysophosphatidic acid (LPA) [1467, 2079]. The clone termed p2y9 (*LPAR4*, Q99677) is also a receptor for lysophosphatidic acid, LPA_4_[1406]. The clone p2y7 (*NOP9*, Q86U38), originally suggested to be a P2Y receptor [22], has been shown to encode a leukotriene receptor [2095]. A P2Y receptor that was initially termed a p2y8 receptor (P79928) has been cloned from *Xenopus laevis*; it shows the rank order of potency ADP*β*S>ATP = uridine triphosphate = guanosine‐5'‐triphosphate= CTP = TTP = ITP>ATP*γ*S and elicits a Ca^2+^‐dependent Cl^‐^ current in *Xenopus* oocytes [169]. The p2y10 clone (*P2RY10*, O00398) lacks functional data. Diadenosine polyphosphates also have effects on as yet uncloned P2Y‐like receptors with the rank order of potency of Ap_4_A>Ap5a>Ap3a, coupling via G_q/11_[270]. P2Y‐like receptors have recently been described on mitochondria [126]. CysLT1 and CysLT2 leukotriene receptors respond to nanomolar concentrations of uridine diphosphate, although they are activated principally by leukotrienes LTC_4_ and LTD_4_[1257, 1258]. Human GPR17 (13304) and rat GPR17, which are structurally related to CysLT and P2Y receptors, are also activated by leukotrienes [1542] as well as uridine diphosphate and UDP‐glucose[344, 540]. Activity at the rat GPR17 is inhibited by submicromolar concentrations of MRS2179 and cangrelor[344].

### Further Reading

Abbracchio MP *et al*. (2006) International Union of Pharmacology LVIII: update on the P2Y G protein‐coupled nucleotide receptors: from molecular mechanisms and pathophysiology to therapy. *Pharmacol. Rev.* **58**: 281‐341 [PMID:16968944]

Burnstock G. (2007) Purine and pyrimidine receptors. *Cell. Mol. Life Sci.* **64**: 1471‐83 [PMID:17375261]

Burnstock G *et al*. (2012) Purinergic signalling and the nervous system. Springer: 1‐715

Erlinge D. (2011) P2Y receptors in health and disease. *Adv. Pharmacol.* **61**: 417‐39 [PMID:21586366]

Jacobson KA. (2013) Structure‐based approaches to ligands for G‐protein‐coupled adenosine and P2Y receptors, from small molecules to nanoconjugates. *J. Med. Chem.* **56**: 3749‐67 [PMID:23597047]

Jacobson KA *et al*. (2009) Development of selective agonists and antagonists of P2Y receptors. *Purinergic Signalling* **5**: 75‐89 [PMID:18600475]

Weisman GA *et al*. (2012) P2Y receptors in the mammalian nervous system: pharmacology, ligands and therapeutic potential. *CNS Neurol Disord Drug Targets* **11**: 722‐38 [PMID:22963441]

von Kügelgen I *et al*. (2011) Molecular pharmacology, physiology, and structure of the P2Y receptors. *Adv. Pharmacol.* **61**: 373‐415 [PMID:21586365]

## Parathyroid hormone receptors

### Overview

The parathyroid hormone receptors (**nomenclature as agreed by the NC‐IUPHAR Subcommittee on Parathyroid Hormone Receptors [** **575** **]**) are family B G protein‐coupled receptors. The parathyroid hormone (PTH)/parathyroid hormone‐related peptide (PTHrP) receptor (PTH1 receptor) is activated by precursor‐derived peptides: PTH(*PTH*, P01270) (84 amino acids), and PTHrP(*PTHLH*, P12272) (141 amino‐acids) and related peptides (PTH‐(1‐34), PTHrP‐(1‐36)(*PTHLH*, P12272)). The parathyroid hormone 2 receptor (PTH2 receptor) is activated by the precursor‐derived peptide TIP39(*PTH2*, Q96A98) (39 amino acids). [^125^I]PTH may be used to label both PTH1 and PTH2 receptors.

**Table:** 

Nomenclature	PTH1 receptor	PTH2 receptor
HGNC, UniProt	*PTH1R*, Q03431	*PTH2R*, P49190
Rank order of potency	PTH (*PTH*, P01270) = PTHrP (*PTHLH*, P12272)	TIP39 (*PTH2*, Q96A98), PTH (*PTH*, P01270) ≫PTHrP (*PTHLH*, P12272)
Endogenous agonists	–	TIP39 (*PTH2*, Q96A98) (pIC_50_ 7.6–9.2) [626, 766]
Agonists	teriparatide (pIC_50_ 7.4) [573]	–
Selective agonists	PTHrP‐(1‐34) (human) (pIC_50_ 7.8–8.1) [574] – Rat	–

### Comments

Although PTH(*PTH*, P01270) is an agonist at human PTH2 receptors, it fails to activate the rodent orthologues. TIP39(*PTH2*, Q96A98) is a weak antagonist at PTH1 receptors [883].

### Further Reading

Cheloha RW *et al*. (2015) Signal transduction at type‐1 parathyroid hormone receptor. *Nat Rev Endo.*

Datta NS *et al*. (2009) PTH and PTHrP signaling in osteoblasts. *Cell. Signal.* **21**: 1245‐54 [PMID:19249350]

Gardella TJ *et al*. (2015) International Union of Basic and Clinical Pharmacology. XCIII. The Parathyroid Hormone Receptors‐Family B G Protein‐Coupled Receptors. *Pharmacol. Rev.* **67**: 310‐37 [PMID:25713287]

Kraenzlin ME *et al*. (2011) Parathyroid hormone analogues in the treatment of osteoporosis. *Nat Rev Endocrinol* [PMID:21750510]

Vilardaga JP *et al*. (2014) Endosomal generation of cAMP in GPCR signaling. *Nat. Chem. Biol.* **10**: 700‐6 [PMID:25271346]

## Platelet‐activating factor receptor

### Overview

Platelet‐activating factor (PAF, 1‐*O*‐alkyl‐2‐acetyl‐sn‐glycero‐3‐phosphocholine) is an ether phospholipid mediator associated with platelet coagulation, but also subserves inflammatory roles. The PAF receptor (**provisional nomenclature recommended by NC‐IUPHAR [** **530** **]**) is activated by PAF and other suggested endogenous ligands are oxidized phosphatidylcholine [1204] and lysophosphatidylcholine[1425]. It may also be activated by bacterial lipopolysaccharide [1358].

**Table:** 

Nomenclature	PAF receptor
HGNC, UniProt	*PTAFR*, P25105
Selective agonists	methylcarbamyl PAF – Unknown
Selective antagonists	foropafant (p*K*_i_ 10.3) [739], ABT‐491 (p*K*_i_ 9.2) [30], CV‐6209 (pIC_50_ 8.1–8.3) [619, 1357], L659989 (p*K*_i_ 7.8) [811], apafant (p*K*_i_ 5.2–7.5) [1460, 1831]
Labelled ligands	[^3^H]PAF (Agonist) (p*K*_d_ 8.8–8.9) [555, 1357]

### Comments

Note that a previously recommended radioligand ([^3^H]apafant; K_d_44.6 nM) is currently unavailable.

### Further Reading

Foord SM *et al*. (2005) International Union of Pharmacology. XLVI. G protein‐coupled receptor list. *Pharmacol Rev* **57**: 279‐288 [PMID:15914470]

Ishii S *et al*. (2000) Platelet‐activating factor (PAF) receptor and genetically engineered PAF receptor mutant mice. *Prog. Lipid Res.* **39**: 41‐82 [PMID:10729607]

Montrucchio G *et al*. (2000) Role of platelet‐activating factor in cardiovascular pathophysiology. *Physiol. Rev.* **80**: 1669‐99 [PMID:11015622]

Prescott SM *et al*. (2000) Platelet‐activating factor and related lipid mediators. *Annu. Rev. Biochem.* **69**: 419‐45 [PMID:10966465]

Shimizu T. (2009) Lipid mediators in health and disease: enzymes and receptors as therapeutic targets for the regulation of immunity and inflammation. *Annu. Rev. Pharmacol. Toxicol.* **49**: 123‐50 [PMID:18834304]

Summers JB *et al*. (1995) Platelet activating factor antagonists. *Adv Pharmacol* **32**: 67‐168 [PMID:7748804]

## Prokineticin receptors

### Overview

Prokineticin receptors, PKR_1_ and PKR_2_(**provisional nomenclature as recommended by NC‐IUPHAR[** **530** **]**) respond to the cysteine‐rich 81‐86 amino‐acid peptides prokineticin‐1(*PROK1*, Q9HC23) (also known as endocrine gland‐derived vascular endothelial growth factor, mambakine) and prokineticin‐2(*PROK2*, Q9HC23) (protein Bv8 homologue). An orthologue of PROK1 from black mamba (*Dendroaspis polylepi*s) venom, mamba intestinal toxin 1 (MIT1, [1678]) is a potent, non‐selective agonist at prokineticin receptors [1215], while Bv8, an orthologue of PROK2 from amphibians (*Bombina sp.*, [1304]), is equipotent at recombinant PKR_1_ and PKR_2_[1371], and has high potency in macrophage chemotaxis assays, which are lost in PKR_1_‐null mice.

**Table:** 

Nomenclature	PKR_1_	PKR_2_
HGNC, UniProt	*PROKR1*, Q8TCW9	*PROKR2*, Q8NFJ6
Rank order of potency	prokineticin‐2 (*PROK2*, Q9HC23) >prokineticin‐1 (*PROK1*, Q9HC23) >prokineticin‐2*β* (*PROK2*) [1109, 1215, 1775]	prokineticin‐2 (*PROK2*, Q9HC23) >prokineticin‐1 (*PROK1*, Q9HC23) >prokineticin‐2*β* (*PROK2*) [1109, 1215, 1775]
Endogenous agonists	prokineticin‐2 (*PROK2*, Q9HC23) (pIC_50_ 8.2–8.4) [300, 1215], prokineticin‐1 (*PROK1*, Q9HC23) (pIC_50_ 6.6–7.6) [300, 1215], prokineticin‐2*β* (*PROK2*) (pIC_50_ 7.5) [300]	prokineticin‐2 (*PROK2*, Q9HC23) (pIC_50_ 8.1–8.2) [300, 1215], prokineticin‐1 (*PROK1*, Q9HC23) (pIC_50_ 7.1–7.3) [300, 1215], prokineticin‐2*β* (*PROK2*) (pIC_50_<6) [300]
Agonists	MIT1 (pIC_50_ 8.4) [1215]	MIT1 (pIC_50_ 9.2) [1215]
Selective agonists	IS20 (pEC_50_ 7.4) [581], IS1 (pEC_50_ 5.6) [581]	–
Selective antagonists	triazine compound PC1 (p*K*_i_ 7.7) [87], triazine compound PC7 (pIC_50_ 7.5) [842, 1552], triazine compound PC10 (pIC_50_ 7) [842]	PKR‐A (pIC_50_ 7.3–7.4) [312]
Labelled ligands	[^125^I]BH‐MIT1 (Agonist) (pIC_50_ 8.4) [1215]	[^125^I]BH‐MIT1 (Agonist) (pIC_50_ 9.2) [1215]

### Comments

Genetic mutations in *PROKR1* are associated with Hirschsprung's disease [1614], while genetic mutations in *PROKR2* are associated with hypogonadotropic hypogonadism with anosmia [430], hypopituitarism with pituitary stalk interruption [1575] and Hirschsprung's disease [1614].

### Further Reading

Boulberdaa M *et al*. (2011) Prokineticin receptor 1 (PKR1) signalling in cardiovascular and kidney functions. *Cardiovasc. Res.* **92**: 191‐8 [PMID:21856786]

Martin C *et al*. (2011) The role of the prokineticin 2 pathway in human reproduction: evidence from the study of human and murine gene mutations. *Endocr. Rev.* **32**: 225‐46 [PMID:21037178]

Monnier J *et al*. (2010) Prokineticins in angiogenesis and cancer. *Cancer Lett.* **296**: 144‐9 [PMID:20633984]

Negri L *et al*. (2012) Bv8/PK2 and prokineticin receptors: a druggable pronociceptive system. *Curr Opin Pharmacol* **12**: 62‐6 [PMID:22136937]

Negri L *et al*. (2007) Bv8/Prokineticin proteins and their receptors. *Life Sci.* **81**: 1103‐16 [PMID:17881008]

Ngan ES *et al*. (2008) Prokineticin‐signaling pathway. *Int. J. Biochem. Cell Biol.* **40**: 1679‐84 [PMID:18440852]

## Prolactin‐releasing peptide receptor

### Overview

The precursor (*PRLH*, P81277) for PrRP generates 31 and 20‐amino‐acid versions. QRFP43(*QRFP*, P83859) (named after a pyroglutamylated arginine‐phenylalanine‐amide peptide) is a 43 amino acid peptide derived from QRFP(P83859) and is also known as P518 or 26RFa. RFRP is an RF amide‐related peptide [756] derived from a FMRFamide‐related peptide precursor (*NPVF*, Q9HCQ7), which is cleaved to generate neuropeptide SF(*NPFF*, O15130), neuropeptide RFRP‐1(*NPVF*, Q9HCQ7), neuropeptide RFRP‐2(*NPVF*, Q9HCQ7) and neuropeptide RFRP‐3(*NPVF*, Q9HCQ7) (neuropeptide NPVF).

**Table:** 

Nomenclature	PrRP receptor
HGNC, UniProt	*PRLHR*, P49683
Rank order of potency	PrRP‐20 (*PRLH*, P81277), PrRP‐31 (*PRLH*, P81277) [1043]
Endogenous agonists	PrRP‐20 (*PRLH*, P81277) (Selective) (p*K*_i_ 9–9.6) [481, 1043], PrRP‐31 (*PRLH*, P81277) (Selective) (p*K*_i_ 9–9.2) [481, 1043]
Endogenous antagonists	neuropeptide Y (*NPY*, P01303) (Selective) (p*K*_i_ 5.4) [1032]
Labelled ligands	[^125^I]PrRP‐20 (human) (Agonist) (p*K*_d_ 9.2–10.6) [1043], [^125^I]PrRP31 (Agonist) [473]

### Comments

The orphan receptor *GPR83*(Q9NYM4) shows sequence similarities with NPFF1, NPFF2, PrRP and QRFP receptors.

### Further Reading

Samson WK *et al*. (2006) Prolactin releasing peptide (PrRP): an endogenous regulator of cell growth. *Peptides* **27**: 1099‐103 [PMID:16500730]

Takayanagi Y *et al*. (2010) Roles of prolactin‐releasing peptide and RFamide related peptides in the control of stress and food intake. *FEBS J.* **277**: 4998‐5005 [PMID:21126313]

## Prostanoid receptors

### Overview

Prostanoid receptors (**nomenclature as agreed by the NC‐IUPHAR Subcommittee on Prostanoid Receptors [** **2043** **]**) are activated by the endogenous ligands prostaglandins PGD_2_, PGE_2_ , PGF_2*α*_, PGH_2_, prostacyclin [PGI_2_] and thromboxane A_2_. Measurement of the potency of PGI_2_ and thromboxane A_2_ is hampered by their instability in physiological salt solution; they are often replaced by cicaprost and U46619, respectively, in receptor characterization studies.

**Table:** 

Nomenclature	DP_1_ receptor	DP_2_ receptor	IP receptor	FP receptor	TP receptor
HGNC, UniProt	*PTGDR*, Q13258	*PTGDR2*, Q9Y5Y4	*PTGIR*, P43119	*PTGFR*, P43088	*TBXA2R*, P21731
Rank order of potency	PGD_2_≫PGE_2_>PGF_2*α*_>PGI_2_, thromboxane A_2_	–	PGI_2_≫PGD_2_, PGE_2_, PGF_2*α*_>thromboxane A_2_	PGF_2*α*_>PGD_2_>PGE_2_>PGI_2_, thromboxane A_2_	thromboxane A_2_ = PGH_2_≫PGD_2_, PGE_2_, PGF_2*α*_, PGI_2_
Rank order of potency	–	PGD_2_≫PGF_2*α*_, PGE_2_>PGI_2_, thromboxane A_2_			
Agonists	–	13,14‐dihydro‐15‐keto‐PGD_2_ (p*K*_i_ 7.4–8.5) [712, 1656, 1815]	iloprost (p*K*_i_ 7.5–8) [7, 2030], treprostinil (p*K*_i_ 7.5) [2019]	bimatoprost (pIC_50_ 5.3) [2044]	–
Selective agonists	BW 245C (p*K*_i_ 8.4–9.4) [171, 2045, 2046], L‐644,698 (p*K*_i_ 9–9.3) [2045, 2046], SQ‐27986 (p*K*_i_ 8) [1712], RS 93520 (Partial agonist) (p*K*_i_ 7.5) [1712], ZK118182 (p*K*_i_ 7.3) [1712]	15(*R*)‐15‐methyl‐PGD_2_ (p*K*_i_ 8.9) [712, 1312, 1815]	AFP‐07 (pIC_50_ 8.5) [288], BMY 45778 (pIC_50_ 8) [881], esuberaprost (p*K*_d_ 7.9) [892], cicaprost (p*K*_i_ 7.8) [7]	fluprostenol (p*K*_i_ 8.6) [7], latanoprost (free acid form) (p*K*_i_ 8.6) [7], AL12180 (pEC_50_ 7.7–7.9) [1714], tafluprost [1843]	I‐BOP (p*K*_d_ 8.9–9.3) [1233], U46619 (p*K*_i_ 7.5) [7], STA_2_ (pIC_50_ 6.4–7.1) [59]
Antagonists	–	ramatroban (p*K*_i_ 7.4) [1815]	–	–	ramatroban (p*K*_i_ 8) [1869]
Selective antagonists	laropiprant (p*K*_i_ 10.1) [1808] – Unknown, BWA868C (p*K*_i_ 8.6–9.3) [171, 606, 2045], S‐5751 (p*K*_i_ 8.8) [54], ONO‐AE3‐237 (p*K*_i_ 7.7) [758, 1895, 1897]	CAY 10471 (pIC_50_ 8.9) [1610, 1927], AZD1981 (pIC_50_ 8.4) [1150]	RO1138452 (p*K*_i_ 8.7) [158], RO3244794 (pA_2_ 8.5) [158]	AS604872 (p*K*_i_ 7.5) [346]	ifetroban (p*K*_i_ 8.4–10) [1426], vapiprost (p*K*_i_ 8.3–9.4) [59, 1151], SQ‐29548 (p*K*_i_ 8.1–9.1) [7, 1834, 2030], ONO‐3708 (p*K*_i_ 7.4–8.9) [910]
Labelled ligands	[^3^H]PGD_2_ (Agonist) (p*K*_d_ 7.9–9.5) [2030, 2045]	[^3^H]PGD_2_ (Agonist) (p*K*_d_ 7.8–8.2) [1216, 1723]	[^3^H]iloprost (Agonist) (p*K*_d_ 7.7–9) [7, 170, 2030]	[^3^H]PGF_2*α*_ (Agonist) (p*K*_d_ 8.1–9) [7, 8, 2030], [^3^H](+)‐fluprostenol (Agonist) (p*K*_d_ 7.5) – Unknown	[^125^I]SAP (Antagonist) (p*K*_d_ 7.7–9.3) [1356], [^125^I]BOP (Agonist) (p*K*_d_ 8.7) [1328], [^3^H]SQ‐29548 (Antagonist) (p*K*_d_ 7.4–8.2) [7, 2030]

**Table:** 

Nomenclature	EP_1_ receptor	EP_2_ receptor	EP_3_ receptor	EP_4_ receptor
HGNC, UniProt	*PTGER1*, P34995	*PTGER2*, P43116	*PTGER3*, P43115	*PTGER4*, P35408
Rank order of potency	PGE_2_>PGF_2*α*_, PGI_2_>PGD_2_, thromboxane A_2_	PGE_2_>PGF_2*α*_, PGI_2_>PGD_2_, thromboxane A_2_	PGE_2_>PGF_2*α*_, PGI_2_>PGD_2_, thromboxane A_2_	PGE_2_>PGF_2*α*_, PGI_2_>PGD_2_, thromboxane A_2_
Endogenous agonists	PGE_1_ (p*K*_i_ 6.8) [1713], PGI_2_ (p*K*_i_ 4.8) [1713]	PGE_2_ (p*K*_i_ 7.5–8.3) [7, 1799, 2030]	–	
Agonists	17‐phenyl‐*ω*‐trinor‐PGE_2_ (p*K*_i_ 8.1) [1713]	evatanepag (pIC_50_ 7.3) [260] – Rat	misoprostol (methyl ester) (EP_3_‐III isoform) (p*K*_i_ 6.5) [7]	–
Selective agonists	ONO‐DI‐004 (p*K*_i_ 6.8) [1826] – Mouse	ONO‐AE1‐259 (p*K*_i_ 8.5) [1826] – Mouse, butaprost (free acid form) (p*K*_i_ 5.9–7) [7, 1799]	SC46275 (pEC_50_ 10.4) [1655] – Guinea pig, MB‐28767 (EP_3_‐III isoform) (p*K*_i_ 9.9) [7], ONO‐AE‐248 (pEC_50_ 5.6–6.7) [534, 1140]	L902688 (pEC_50_ 8.1–10.3) [535, 1064], ONO‐AE1‐437 (p*K*_i_ 9.1) [1294] – Mouse, CP734432 (pIC_50_ 8.7) [1529], ONO‐AE1‐329 (pEC_50_ 7.7–7.8) [534, 535]
Antagonists	–	–	–	evatanepag (p*K*_i_ 8.6) [1345],
Selective antagonists	ONO‐8711 (p*K*_i_ 9.2) [1992], GW848687X (pIC_50_ 8.6) [605], SC‐51322 (p*K*_i_ 7.9) [7]	TG4‐155 (TG4‐155 also has affinity for the human DP1 receptor (p*K*_b_ = 7.8)) (p*K*_B_ 8.6) [865], TG7‐171 (p*K*_B_ 8.6) [567], PF‐04852946 (p*K*_B_ 8.4–8.5) [920], PF‐04418948 (PF‐04418948 has weaker affinity at the EP2‐receptor in guinea‐pigs) (p*K*_B_ 8.3) [153, 2136]	L‐798,106 (EP_3_‐III isoform) (p*K*_i_ 7.8–9.7) [888, 890, 1810], L‐826266 (EP_3_‐III isoform (pK_i_=8.04 in the presence of HSA)) (p*K*_i_ 9.1) [890], ONO‐AE3‐240 (pIC_50_ 8.8) [38] – Mouse, DG‐041 (p*K*_i_ 8.4) [888]	MK‐2894 (p*K*_i_ 9.2) [7, 161, 350], ONO‐AE3‐208 (p*K*_i_ 8.5), BGC201531 (p*K*_i_ 7.9) [1230], ER819762 (pIC_50_ 7.2) [304], GW 627368 (p*K*_i_ 7–7.1) [2030, 2031]
Labelled ligands	[^3^H]PGE_2_ (Agonist) (p*K*_d_ 7.6–7.9) [7, 1713, 2030]	[^3^H]PGE_2_ (Agonist) (p*K*_d_ 7.7–7.9) [7, 2030]	[^3^H]PGE_2_ (Agonist) (p*K*_d_ 8.2–9.5) [7, 2030]	[^3^H]PGE_2_ (Agonist) (p*K*_d_ 7.6–9.5) [7, 401, 2019, 2030]

### Comments

ramatroban is an antagonist at both DP_2_ and TP receptors. Whilst cicaprost is selective for IP receptors, it does exhibit moderate agonist potency at EP_4_ receptors [7]. Apart from IP receptors, iloprost also binds to other prostanoid receptors such as EP_1_ receptors. The TP receptor exists in *α* and *β* isoforms due to alternative splicing of the cytoplasmic tail [1566]. The IP receptor agonist treprostinil binds also to human EP_2_ and DP_1_ receptors with high affinity (p*K*_i_ 8.44 and 8.36, respectively).

The EP_1_ agonist 17‐phenyl‐*ω*‐trinor‐PGE_2_ also shows agonist activity at EP_3_ receptors. Butaprost and SC46275 may require de‐esterification within tissues to attain full agonist potency. There is evidence for subtypes of FP [1105], IP [1851, 2037] and TP [1005] receptors. mRNA for the EP_1_ and EP_3_ receptors undergo alternative splicing to produce two [1441] and at least six variants, respectively, which can interfere with signalling [1441] or generate complex patterns of G‐protein (G_i/o_, G_q/11_, G_s_ and G_12,13_) coupling (*e.g.* [997, 1370]). The number of EP_3_ receptor (protein) variants are variable depending on species, with five in human, three in rat and three in mouse. The possibility of additional receptors for the isoprostanes has been suggested [1531]. Putative receptor(s) for prostamide F (which as yet lack molecular correlates) and which preferentially recognize PGF2‐1‐ethanolamide and its analogues (*e.g*. Bimatoprost) have been identified, together with moderate‐potency antagonists (*e.g.* AGN 211334) [2042].

The free acid form of AL‐12182, AL12180, used in *in vitro* studies, has a EC_50_ value of 15nM which is the concentration of the compound giving half‐maximal stimulation of inositol phosphate turnover in HEK‐293 cells expressing the human FP receptor [1714].

References given alongside the TP receptor agonists I‐BOP [1233] and STA_2_[59] use human platelets as the source of TP receptors for competition radio‐ligand binding assays to determine the indicated activity values.

Pharmacological evidence for a second IP receptor, denoted IP_2_, in the central nervous system [1851, 1994] and in the BEAS‐2B human airway epithelial cell line [2033] is available. This receptor is selectively activated by 15R‐17,18,19,20‐tetranor‐16‐m‐tolyl‐isocarbacyclin (15R‐TIC) and 15R‐Deoxy 17,18,19,20‐tetranor‐16‐m‐tolyl‐isocarbacyclin (15‐deoxy‐TIC). However, molecular biological evidence for the IP_2_ subtype is currently lacking.

### Further Reading

Billot X *et al*. (2003) Discovery of a potent and selective agonist of the prostaglandin EP4 receptor. *Bioorg. Med. Chem. Lett.* **13**: 1129‐32 [PMID:12643927]

Félétou M *et al*. (2010) Vasoconstrictor prostanoids. *Pflugers Arch.* **459**: 941‐50 [PMID:20333529]

Félétou M *et al*. (2010) The thromboxane/endoperoxide receptor (TP): the common villain. *J. Cardiovasc. Pharmacol.* **55**: 317‐32 [PMID:20422736]

Schuligoi R *et al*. (2010) CRTH2 and D‐type prostanoid receptor antagonists as novel therapeutic agents for inflammatory diseases. *Pharmacology* **85**: 372‐82 [PMID:20559016]

Woodward DF *et al*. (2011) International union of basic and clinical pharmacology. LXXXIII: classification of prostanoid receptors, updating 15 years of progress. *Pharmacol. Rev.* **63**: 471‐538 [PMID:21752876]

Yang C *et al*. (2011) Prostaglandin E receptors as inflammatory therapeutic targets for atherosclerosis. *Life Sci.* **88**: 201‐5 [PMID:21112342]

af Forselles KJ *et al*. (2011) In vitro and in vivo characterization of PF‐04418948, a novel, potent and selective prostaglandin EP_2 receptor antagonist. *Br. J. Pharmacol.* **164**: 1847‐56 [PMID:21595651]

## Proteinase‐activated receptors

### Overview

Proteinase‐activated receptors (PARs, **nomenclature as agreed by the NC‐IUPHAR Subcommittee on Proteinase‐activated Receptors [** **770** **]**) are unique members of the GPCR superfamily activated by proteolytic cleavage of their amino terminal exodomains. Agonist proteinase‐induced hydrolysis unmasks a tethered ligand (TL) at the exposed amino terminus, which acts intramolecularly at the binding site in the body of the receptor to effect transmembrane signalling. TL sequences at human PAR1‐4 are SFLLRN‐NH_2_, SLIGKV‐NH_2_, TFRGAP‐NH_2_ and GYPGQV‐NH_2_, respectively. With the exception of PAR3, these synthetic peptide sequences (as carboxyl terminal amides) are able to act as agonists at their respective receptors. Several proteinases, including neutrophil elastase, cathepsin G and chymotrypsin can have inhibitory effects at PAR1 and PAR2 such that they cleave the exodomain of the receptor without inducing activation of G*α*q‐coupled calcium signalling, thereby preventing activation by activating proteinases but not by agonist peptides. Neutrophil elastase cleavage of PAR2 can however activate MAP kinase signaling by exposing a TL that is different from the one revealed by trypsin [1553]. The role of such an action *in vivo* is unclear.

**Table:** 

Nomenclature	PAR1	PAR2	PAR3	PAR4
HGNC, UniProt	*F2R*, P25116	*F2RL1*, P55085	*F2RL2*, O00254	*F2RL3*, Q96RI0
Agonist proteases	thrombin (*F2*, P00734), activated protein C (*PROC*, P04070), matrix metalloproteinase 1 (*MMP1*, P45452), matrix metalloproteinase 13 (*MMP13*, P45452) [70]	Trypsin, tryptase, TF/VIIa, Xa	thrombin (*F2*, P00734)	thrombin (*F2*, P00734), trypsin, cathepsin G (*CTSG*, P08311)
Selective agonists	TFLLR‐NH_2_ (pEC_50_ 5.4) [340]	GB110 (pEC_50_ 6.5) [98], 2‐furoyl‐LIGRLO‐amide (p*K*_i_ 5.4) [1243], SLIGKV‐NH_2_ [1069], SLIGRL‐NH_2_ [1069]	–	AYPGKF‐NH2, GYPGKF‐NH2, GYPGQV‐NH_2_
Selective antagonists	vorapaxar (p*K*_i_ 8.1) [281], atopaxar (pIC_50_ 7.7) [978], RWJ‐56110 (pIC_50_ 6.4) [48]	GB88 (pIC_50_ 5.7) [1813], P2pal18s [1705]	–	–
Labelled ligands	[^3^H]haTRAP (Agonist) (p*K*_d_ 7.8) [15]	2‐furoyl‐LIGRL[N‐(Alexa Fluor 594)‐O]‐NH_2_ (Agonist) [771], 2‐furoyl‐LIGRL[N[^3^H]propionyl]‐O‐NH_2_ (Agonist) [771], [^3^H]2‐furoyl‐LIGRL‐NH_2_ (Selective Agonist) [903], trans‐cinnamoyl‐LIGRLO [N‐[^3^H]propionyl]‐NH_2_ (Agonist) [28]	–	–
Comments	TFLLR‐NH_2_ is selective relative to the PAR_2_ receptor [155, 915].	2‐Furoyl‐LIGRLO‐NH_2_ activity was measured via calcium mobilisation in HEK 293 cells which constitutively coexpress human PAR_1_ and PAR_2_.	–	–

### Comments

thrombin(*F2*, P00734) is inactive at the PAR_2_ receptor.

Endogenous serine proteases (EC 3.4.21.) active at the proteinase‐activated receptors include: thrombin(*F2*, P00734), generated by the action of Factor X (*F10*, P00742) on liver‐derived prothrombin (*F2*, P00734); trypsin, generated by the action of enterokinase (*TMPRSS15*, P98073) on pancreatic‐derived trypsinogen (*PRSS1*, P07477); tryptase, a family of enzymes (*α*/*β*1 *TPSAB1*, Q15661 ; *γ*1 *TPSG1*, Q9NRR2; *δ*1 *TPSD1*, Q9BZJ3) secreted from mast cells; cathepsin G (*CTSG*, P08311) generated from leukocytes; liver‐derived protein C (*PROC*, P04070) generated in plasma by thrombin(*F2*, P00734) and matrix metalloproteinase 1 (*MMP1*, P45452).

### Further Reading

Adams MN *et al*. (2011) Structure, function and pathophysiology of protease activated receptors. *Pharmacol. Ther.* **130**: 248‐82 [PMID:21277892]

Canto I *et al*. (2012) Allosteric modulation of protease‐activated receptor signaling. *Mini Rev Med Chem* **12**: 804‐11 [PMID:22681248]

García PS *et al*. (2010) The role of thrombin and protease‐activated receptors in pain mechanisms. *Thromb. Haemost.* **103**: 1145‐51 [PMID:20431855]

Hollenberg MD *et al*. (2002) International Union of Pharmacology. XXVIII. Proteinase‐activated receptors. *Pharmacol. Rev.* **54**: 203‐17 [PMID:12037136]

Ramachandran R *et al*. (2012) Targeting proteinase‐activated receptors: therapeutic potential and challenges. *Nat Rev Drug Discov* **11**: 69‐86 [PMID:22212680]

Soh UJ *et al*. (2010) Signal transduction by protease‐activated receptors. *Br. J. Pharmacol.* **160**: 191‐203 [PMID:20423334]

Vergnolle N. (2009) Protease‐activated receptors as drug targets in inflammation and pain. *Pharmacol. Ther.* **123**: 292‐309 [PMID:19481569]

## QRFP receptor

### Overview

The human gene encoding the QRFP receptor (QRFPR, also known as the peptide P518 receptor), previously designated as an orphan GPCR receptor was identified in 2001 by Lee *et al.* from a hypothalamus cDNA library [1066]. However, the reported cDNA (AF411117) is a chimera with bases 1‐127 derived from chromosome 1 and bases 155‐1368 derived from chromosome 4. When corrected, QRFPR (also referred to as SP9155 or AQ27) encodes a 431 amino acid protein that shares sequence similarities in the transmembrane spanning regions with other peptide receptors. These include neuropeptide FF2 (38%), neuropeptide Y2 (37%) and galanin GalR1 (35%) receptors.

**Table:** 

Nomenclature	QRFP receptor
HGNC, UniProt	*QRFPR*, Q96P65
Endogenous agonists	QRFP43 (*QRFP*, P83859) (pIC_50_ 7.8–9.3) [557, 1850] – Rat, QRFP26 (*QRFP*) (pEC_50_ 8.2) [867]
Labelled ligands	[^125^I]QRFP43 (human) (Agonist) (p*K*_d_ 7.8–10.3) [557, 1017, 1850]

### Comments

The orphan receptor *GPR83*(9NYM4) shows sequence similarities with the QRFP receptor, as well as with the NPFF1, NPFF2, and PrRP receptors.

### Further Reading

Fukusumi S *et al*. (2006) Recent advances in mammalian RFamide peptides: the discovery and functional analyses of PrRP, RFRPs and QRFP. *Peptides* **27**: 1073‐86 [PMID:16500002]

## Relaxin family peptide receptors

### Overview

Relaxin family peptide receptors (RXFP, **nomenclature as agreed by the NC‐IUPHAR Subcommittee on Relaxin family peptide receptors [** **105** **,** **677** **]**) may be divided into two pairs, RXFP1/2 and RXFP3/4. Endogenous agonists at these receptors are a number of heterodimeric peptide hormones analogous to insulin: relaxin‐1(*RLN1*, P04808), relaxin(*RLN2*, P04090), relaxin‐3(*RLN3*, Q8WXF3) (also known as INSL7), insulin‐like peptide 3 (INSL3(*INSL3*, P51460)) and INSL5(*INSL5*, Q9Y5Q6).

Species homologues of relaxin have distinct pharmacology ‐ relaxin(*RLN2*, P04090) interacts with RXFP1, RXFP2 and RXFP3, whereas mouse and rat relaxin selectively bind to and activate RXFP1 [1686] and porcine relaxin may have a higher efficacy than human relaxin(*RLN2*, P04090) [678]. Relaxin‐3(*RLN3*, Q8WXF3) has differential affinity for RXFP2 receptors between species; mouse and rat RXFP2 have a higher affinity for relaxin‐3(*RLN3*, Q8WXF3) [1685]. At least two binding sites have been identified on the RXFP1 and RXFP2 receptors: a high‐affinity site in the leucine‐rich repeat region of the ectodomain and a somewhat lower‐affinity site located in the surface loops of the transmembrane domain [678, 1812]. The unique N‐terminal LDLa module of RXFP1 and RXFP2 is essential for receptor signalling [1687].

**Table:** 

Nomenclature	RXFP1 receptor	RXFP2 receptor	RXFP3 receptor	RXFP4 receptor
HGNC, UniProt	*RXFP1*, Q9HBX9	*RXFP2*, Q8WXD0	*RXFP3*, Q9NSD7	*RXFP4*, Q8TDU9
Rank order of potency	relaxin (*RLN2*, P04090) = relaxin‐1 (*RLN1*, P04808) >relaxin‐3 (*RLN3*, Q8WXF3) [1812]	INSL3 (*INSL3*, P51460) >relaxin (*RLN2*, P04090) ≫relaxin‐3 (*RLN3*, Q8WXF3) [1022, 1812]	relaxin‐3 (*RLN3*, Q8WXF3) >relaxin‐3 (B chain) (*RLN3*, Q8WXF3) >relaxin (*RLN2*, P04090) [1119]	INSL5 (*INSL5*, Q9Y5Q6) = relaxin‐3 (*RLN3*, Q8WXF3) >relaxin‐3 (B chain) (*RLN3*, Q8WXF3) [1117, 1118]
Endogenous antagonists	–	–	INSL5 (*INSL5*, Q9Y5Q6) (p*K*_i_ 7) [2129]	–
Antagonists	B‐R13/17K H2 relaxin (pEC_50_ 5.7–6.7) [788, 1382], LGR7‐truncate [1687]	–	R3(B*Δ*23‐27)R/I5 chimeric peptide (pIC_50_ 9.2) [1018]	R3(B*Δ*23‐27)R/I5 chimeric peptide (pIC_50_ 8–8.6) [714, 1018]
Selective antagonists	–	A(9‐26)INSL3 (p*K*_i_ 9.1) [787], A(10‐24)INSL3 (p*K*_i_ 8.7) [787], A(C10/15S)INSL3 (p*K*_i_ 8.6) [2118], INSL3 B chain dimer analogue 8 (p*K*_i_ 8.5) [1710], A(*Δ*10/15C)INSL3 (p*K*_i_ 8.3) [2118], cyclic INSL3 B‐chain analogue 6 (p*K*_i_ 6.7) [1708], INSL3 B‐chain analogue (p*K*_i_ 5.1) [411], (des 1‐8) A‐chain INSL3 analogue [253]	minimised relaxin‐3 analogue 3 (p*K*_i_ 7.6) [1706], R3‐B1‐22R (pIC_50_ 7.4) [714]	minimised relaxin‐3 analogue 3 (pIC_50_ 6.6) [1706]
Selective allosteric modulators	ML290 (Agonist) (pEC_50_ 7) [2057, 2060]	–	–	–
Labelled ligands	[^33^P]relaxin (human) (Agonist) (p*K*_d_ 9.3–9.7) [678, 1812], [^125^I]relaxin (human) (Agonist)	[^125^I]INSL3 (human) (Agonist) (p*K*_d_ 10) [1340], [^33^P]relaxin (human) (Agonist) (p*K*_d_ 9–9.2) [678, 1812]	[^125^I]relaxin‐3 (human) (Agonist) (p*K*_d_ 9.5) [1119], [^125^I]relaxin‐3‐B/INSL5 A chimera (Agonist) (p*K*_d_ 9.3) [1117]	[^125^I]relaxin‐3 (human) (Agonist) (p*K*_d_ 8.7–9.7) [1118], [^125^I]relaxin‐3‐B/INSL5 A chimera (Agonist) (p*K*_d_ 8.9) [1117], europium‐labelled INSL5 (p*K*_d_ 8.3) [714]
Comments	europium‐labelled relaxin is a fluorescent ligand for this receptor (*K*_d_=0.5nM) [1707].	europium‐labelled INSL3 is a fluorescent ligand for this receptor (*K*_d_=1nM) [1709].	europium‐labelled relaxin‐3‐B/INSL5 A chimera and R3‐B1‐22R are fluorescent ligands for this receptor (*K*_d_=5nM and 28nM) [714, 715].	europium‐labelled relaxin‐3‐B/INSL5 A chimera is a fluorescent probe at this receptor (*K*_d_=5nM) [714]. europium‐labelled mouse INSL5 is a fluorescent ligand at this receptor (*K*_d_=5nM) [120].

### Comments

Relaxin has recently successfully completed a Phase III clinical trial for the treatment of acute heart failure. 48 hr infusion of relaxin reduced dyspnoea and 180 day mortality [1262]. Small molecule agonists active at RXFP1 receptors have been developed [1718, 2060], and one of these (ML290) is an allosteric agonist at RXFP1 [2060]. The antifibrotic actions of relaxin are dependent on the angiotensin receptor AT_2_, are absent in AT_2_ knockout mice, and are associated with heterodimer formation between RXFP1 and AT_2_[330]. Mutations in *INSL3* and *LGR8* (RXFP2) have been reported in populations of patients with cryptorchidism [512]. Numerous splice variants of the human RXFP1 and RXFP2 receptors have been identified, most of which do not bind relaxin family peptides [1340]. Splice variants of RXFP1 encoding the N‐terminal LDLa module act as antagonists of RXFP1 signalling [1685, 1687]. cAMP elevation appears to be a major signalling pathway for RXFP1 and RXFP2 [795, 796], but RXFP1 also activates MAP kinases, nitric oxide signalling, tyrosine kinase phosphorylation and relaxin can interact with glucocorticoid receptors [681]. RXFP1 signalling involves lipid rafts, residues in the C‐terminus of the receptor and activation of phosphatidylinositol‐3‐kinase [682]. More recent studies provide evidence that RXFP1 is pre‐assembled in signalosomes with other signalling proteins including G*α*_s_, G*β* *γ* and adenylyl cyclase 2 that display constitutive activity and are exquisitely sensitive to sub‐picomolar concentrations of relaxin [679]. The cyclic AMP signalling pattern is highly dependent on the cell type in which RXFP1 is expressed [680].

The receptor expression profiles suggested that RXFP3 was a neuropeptide receptor and RXFP4 a gut hormone receptor. Studies in rats and mice (including wildtype, and relaxin‐3 and RXFP3 gene‐deletion strains [671, 782, 1759, 1971] have revealed putative roles for the relaxin‐3/RXFP3 system in the modulation of feeding [564, 566, 714, 1706, 1760], anxiety [1618, 2114], and reward and motivated, goal‐directed behaviours [782, 1619, 1971], particularly in relation to the integration of stress and corticotrophin‐releasing factor signalling [1162], with implications for the therapeutic treatment of clinical anxiety, depression, eating disorders and addiction (see [565, 1761] for review). Relaxin‐3(*RLN3*, Q8WXF3) acts as an agonist at both RXFP3 and RXFP4 whereas INSL5(*INSL5*, Q9Y5Q6) is an agonist at RXFP4 and a weak antagonist at RXFP3. Unlike RXFP1 and RXFP2 both RXFP3 and RXFP4 are encoded by a single exon and therefore no splice variants exist. The rat RXFP3 sequence has two potential start codons that encode RXFP3L and RXFP3S with the longer variant having an additional 7 amino‐acids at the N‐terminus. It is not known which variant is expressed. Rat and dog RXFP4 sequences are pseudogenes [2027]. Recent studies suggest that INSL5 is an incretin secreted from enteroendocrine L cells and that the INSL5/RXFP4 system has roles in controlling food intake and glucose homeostasis [652]. RXFP3 couples to G_i/o_ and inhibits adenylyl cyclase [1119, 2144], and also causes Erk1/2 phosphorylation [2144]. Relatively little is known about RXFP4 signalling but like RXFP3 it couples to inhibitory G_i/o_ G‐proteins [1120]. Recent studies suggest that relaxin(*RLN2*, P04090) also interacts with RXFP3 to cause a pattern of activation of signalling pathways that are a subset of those activated by relaxin‐3(*RLN3*, Q8WXF3). The two patterns of signaling observed in several cell types expressing RXFP3 are strong inhibition of forskolin‐stimulated cyclic AMP accumulation, ERK1/2 activation and nuclear factor NF*κ*‐B reporter gene activation with relaxin‐3(*RLN3*, Q8WXF3), and weaker activity with relaxin(*RLN2*, P04090), porcine relaxin, or insulin‐like peptide 3 (INSL3(*INSL3*, P51460)) and a strong stimulation of activator protein (AP)‐1 reporter genes with relaxin(*RLN2*, P04090), and weaker activation with relaxin‐3(*RLN3*, Q8WXF3) or porcine relaxin[2144]. Thus at RXFP3, relaxin(*RLN2*, P04090) is a biased ligand compared to the cognate ligand relaxin‐3(*RLN3*, Q8WXF3). Two pharmacologically distinct ligand binding sites were also identified on RXFP3‐expressing cells using [^125^I]relaxin‐3‐B/INSL5 A chimera which binds with high affinity and displays competition by relaxin‐3(*RLN3*, Q8WXF3) or a relaxin‐3 (B chain) (*RLN3*, Q8WXF3) peptide, and [^125^I]relaxin (human) which displays competition by relaxin(*RLN2*, P04090), relaxin‐3(*RLN3*, Q8WXF3), or INSL3(*INSL3*, P51460) and weakly by porcine relaxin.

### Further Reading

Bathgate RA *et al*. (2013) Relaxin family peptides and their receptors. *Physiol. Rev.* **93**: 405‐80 [PMID:23303914]

Bathgate RA *et al*. (2006) International Union of Pharmacology LVII: recommendations for the nomenclature of receptors for relaxin family peptides. *Pharmacol Rev* **58**: 7‐31 [PMID:16507880]

Callander GE *et al*. (2010) Relaxin family peptide systems and the central nervous system. *Cell. Mol. Life Sci.* **67**: 2327‐41 [PMID:20213277]

Du XJ *et al*. (2010) Cardiovascular effects of relaxin: from basic science to clinical therapy. *Nat Rev Cardiol* **7**: 48‐58 [PMID:19935741]

Halls ML *et al*. (2015) International Union of Basic and Clinical Pharmacology. XCV. Recent advances in the understanding of the pharmacology and biological roles of relaxin family peptide receptors 1‐4, the receptors for relaxin family peptides. *Pharmacol. Rev.* **67**: 389‐440 [PMID:25761609]

Ivell R *et al*. (2011) Relaxin family peptides in the male reproductive system–a critical appraisal. *Mol. Hum. Reprod.* **17**: 71‐84 [PMID:20952422]

Kong RC *et al*. (2010) Membrane receptors: structure and function of the relaxin family peptide receptors. *Mol. Cell. Endocrinol.* **320**: 1‐15 [PMID:20138959]

van der Westhuizen ET *et al*. (2008) Relaxin family peptide receptors–from orphans to therapeutic targets. *Drug Discov. Today* **13**: 640‐51 [PMID:18675759]

## Somatostatin receptors

### Overview

Somatostatin (somatotropin release inhibiting factor) is an abundant neuropeptide, which acts on five subtypes of somatostatin receptor (sst_1_‐sst_5_; **nomenclature as agreed by the NC‐IUPHAR Subcommittee on Somatostatin Receptors [790]**). Activation of these receptors produces a wide range of physiological effects throughout the body including the inhibition of secretion of many hormones. The relationship of the cloned receptors to endogenously expressed receptors is not yet well established in some cases. Endogenous ligands for these receptors are somatostatin‐14 (SRIF‐14(*SST*, P61278)) and somatostatin‐28 (SRIF‐28(*SST*, P61278)). Cortistatin‐14{Mouse, Rat} has also been suggested to be an endogenous ligand for somatostatin receptors [404].

**Table:** 

Nomenclature	sst_1_ receptor	sst_2_ receptor	sst_3_ receptor	sst_4_ receptor	sst_5_ receptor
HGNC, UniProt	*SSTR1*, P30872	*SSTR2*, P30874	*SSTR3*, P32745	*SSTR4*, P31391	*SSTR5*, P35346
Agonists	pasireotide (pIC_50_ 8) [1669]	vapreotide (p*K*_i_ 8.3–10.1) [233, 1474], pasireotide (pIC_50_ 9) [1669]	pasireotide (pIC_50_ 8.8) [1669], vapreotide (p*K*_i_ 7.4–7.9) [233, 1474, 1738]	NNC269100 (p*K*_i_ 8.2) [1132]	pasireotide (pIC_50_ 9.8) [1669], vapreotide (p*K*_i_ 7.3–9.2) [233, 1265, 1474, 1736, 1737, 1738]
Selective agonists	L‐797,591 (p*K*_i_ 8.8) [1595], Des‐Ala^1,2,5^‐[D‐Trp^8^,IAmp^9^]SRIF (pIC_50_ 7.5) [484]	L‐054,522 (p*K*_i_ 11) [2084], BIM 23027 (pIC_50_ 10.9) [271], seglitide (p*K*_i_ 8.8–10.3) [233, 1474, 1736, 1737, 1738, 2084], octreotide (p*K*_i_ 8.7–9.9) [233, 1474, 1736, 1737, 1738, 2084]	L‐796,778 (p*K*_i_ 7.6) [1595]	L‐803,087 (p*K*_i_ 9.2) [1595]	BIM 23052 (p*K*_i_ 7.4–9.6) [1265, 1736, 1737, 1738], L‐817,818 (p*K*_i_ 9.4) [1595], BIM 23268 (p*K*_i_ 8.7) [1265]
Selective antagonists	SRA880 (p*K*_d_ 8–8.1) [792]	[D‐Tyr^8^]CYN 154806 (p*K*_d_ 8.1–8.9) [1412]	NVP ACQ090 (p*K*_i_ 7.9) [793]	–	–
Labelled ligands	–	[^125^I]Tyr^3^ SMS 201‐995 (Agonist) (p*K*_d_ 9.9) [1736, 1737], [^125^I]BIM23027 (Agonist) (pIC_50_ 9.7) [772] – Rat	–	–	[^125^I]Tyr^3^ SMS 201‐995 (Agonist) (p*K*_d_ 9.6) [1736, 1737]
Comments	–	–	Troxler *et al.* (2010) describe the identification of non‐peptidic, subtype‐selective sst_3_ receptor antagonists [1907].	–	–

### Comments

[^125^I]Tyr^11^‐SRIF‐14, [^125^I]LTT‐SRIF‐28, [^125^I]CGP 23996 and [^125^I]Tyr^10^‐CST14 may be used to label somatostatin receptors nonselectively. A number of nonpeptide subtype‐selective agonists have been synthesised [1595]. A novel peptide somatostatin analogue, somatoprim, has affinity for sst_2_, sst_4_ and sst_5_ receptors and is a potent inhibitor of GH secretion [1514, 1726].

### Further Reading

Ben‐Shlomo A *et al*. (2010) Pituitary somatostatin receptor signaling. *Trends Endocrinol. Metab.* **21**: 123‐33 [PMID:20149677]

Colao A *et al*. (2011) Resistance to somatostatin analogs in acromegaly. *Endocr. Rev.* **32**: 247‐71 [PMID:21123741]

Csaba Z *et al*. (2012) Molecular mechanisms of somatostatin receptor trafficking. *J. Mol. Endocrinol.* **48**: R1‐12 [PMID:22159161]

Hoyer D *et al*. (2000) Somatostatin receptors. *In The IUPHAR Compendium of Receptor Characterization and Classification, 2nd edn.* Edited by Watson SP, Girdlestone D: IUPHAR Media: 354‐364

Schulz S *et al*. (2014) Fine‐tuning somatostatin receptor signalling by agonist‐selective phosphorylation and dephosphorylation: IUPHAR Review 5. *Br. J. Pharmacol.* **171**: 1591‐9 [PMID:24328848]

Zatelli MC *et al*. (2009) The significance of new somatostatin analogs as therapeutic agents. *Curr Opin Investig Drugs* **10**: 1025‐31 [PMID:19777390]

## Succinate receptor

### Overview

**Nomenclature as recommended by NC‐IUPHAR[** **396** **]**.

**Table:** 

Nomenclature	succinate receptor
HGNC, UniProt	*SUCNR1*, Q9BXA5
Endogenous agonists	succinic acid (pEC_50_ 3.1–4.7) [728, 1785]

## Tachykinin receptors

### Overview

Tachykinin receptors (**provisional nomenclature as recommended by NC‐IUPHAR[** **530** **]**) are activated by the endogenous peptides substance P(*TAC1*, P20366) (SP), neurokinin A (*TAC1*, P20366) (NKA; previously known as substance K, neurokinin *α*, neuromedin L), neurokinin B (*TAC3*, Q9UHF0) (NKB; previously known as neurokinin *β*, neuromedin K), neuropeptide K(*TAC1*, P20366) and neuropeptide *γ*(*TAC1*, P20366) (N‐terminally extended forms of neurokinin A). The neurokinins (A and B) are mammalian members of the tachykinin family, which includes peptides of mammalian and nonmammalian origin containing the consensus sequence: Phe‐x‐Gly‐Leu‐Met. Marked species differences in *in vitro* pharmacology exist for all three receptors, in the context of nonpeptide ligands.

**Table:** 

Nomenclature	NK_1_ receptor	NK_2_ receptor	NK_3_ receptor
HGNC, UniProt	*TACR1*, P25103	*TACR2*, P21452	*TACR3*, P29371
Rank order of potency	substance P (*TAC1*, P20366) >neurokinin A (*TAC1*, P20366) >neurokinin B (*TAC3*, Q9UHF0)	neurokinin A (*TAC1*, P20366) >neurokinin B (*TAC3*, Q9UHF0) ≫substance P (*TAC1*, P20366)	neurokinin B (*TAC3*, Q9UHF0) >neurokinin A (*TAC1*, P20366) >substance P (*TAC1*, P20366)
Agonists	substance P‐OMe (pIC_50_ 7.4–7.5) [1882]	–	–
Selective agonists	[Sar^9^,Met(O_2_)^11^]SP (pIC_50_ 9.7–9.9) [1882], septide (p*K*_i_ 7–9.3) [125, 711], [Pro^9^]SP (pIC_50_ 8.6) [1896] – Rat	[Lys^5^,Me‐Leu^9^,Nle^10^]NKA‐(4‐10) (pIC_50_ 8.8–9.4) [1229] – Rat, GR64349 (pEC_50_ 8.4) [407] – Rat, [*β*Ala^8^]neurokinin A‐(4‐10) (p*K*_d_ 6) [477]	[Phe(Me)^7^]neurokinin B (p*K*_i_ 8.7–9.6) [1644, 1645], senktide (p*K*_i_ 7.1–8.6) [1644, 1645, 1882]
Selective antagonists	aprepitant (p*K*_i_ 10.1) [673, 674], lanepitant (p*K*_i_ 9.8–10) [613], lanepitant (pIC_50_ 9.8) [798], CP 99994 (p*K*_i_ 9.3–9.7) [50, 1645], casopitant (p*K*_i_ 9.4) [798, 1905], vestipitant (p*K*_i_ 9.4) [221, 418], nolpitantium (pIC_50_ 8.9–9) [1882], RP67580 (pIC_50_ 7.7) [528]	GR94800 (p*K*_i_ 9.8) [200], saredutant (p*K*_i_ 9.4–9.7) [50, 477, 1645], GR 159897 (p*K*_d_ 7.8–9.5) [133, 477, 1770], MEN10627 (p*K*_i_ 9.2) [603], nepadutant (p*K*_i_ 8.5–8.7) [272, 343]	osanetant (p*K*_i_ 8.4–9.7) [50, 110, 342, 476, 898, 1450, 1644, 1645, 1882], talnetant (p*K*_i_ 7.4–9) [129, 604, 1644, 1645], PD157672 (pIC_50_ 7.8–7.9) [165, 1882]
Labelled ligands	[^125^I]L703,606 (Antagonist) (p*K*_d_ 9.5) [537], [^125^I]BH‐[Sar^9^,Met(O_2_)^11^]SP (Agonist) (p*K*_d_ 9) [1901] – Rat, [^3^H]BH‐[Sar^9^,Met(O_2_)^11^]SP (Agonist) (p*K*_d_ 8.7) [1902] – Rat, [^3^H]SP (human, mouse, rat) (Agonist) (p*K*_d_ 8.6) [80], [^125^I]SP (human, mouse, rat) (Agonist), [^18^F]SPA‐RQ (Antagonist) [317]	[^3^H]saredutant (Antagonist) (p*K*_d_ 9.7) [649] – Rat, [^125^I]NKA (human, mouse, rat) (Agonist) (p*K*_d_ 9.3) [1990], [^3^H]GR100679 (Antagonist) (p*K*_d_ 9.2) [669]	[^3^H]osanetant (Antagonist) (p*K*_d_ 9.9), [^3^H]senktide (Agonist) (p*K*_d_ 8.1–8.7) [660] – Guinea pig, [^125^I][MePhe^7^]NKB (Agonist)

### Comments

The NK_1_ receptor has also been described to couple to other G proteins [1606]. The hexapeptide agonist septide appears to bind to an overlapping but non‐identical site to substance P(*TAC1*, P20366) on the NK_1_ receptor. There are suggestions for additional subtypes of tachykinin receptor; an orphan receptor (SwissProt P30098) with structural similarities to the NK_3_ receptor was found to respond to NKB when expressed in *Xenopus* oocytes or Chinese hamster ovary cells [433, 1004].

### Further Reading

Commons KG. (2010) Neuronal pathways linking substance P to drug addiction and stress. *Brain Res.* **1314**: 175‐82 [PMID:19913520]

Douglas SD *et al*. (2011) Neurokinin‐1 receptor: functional significance in the immune system in reference to selected infections and inflammation. *Ann. N. Y. Acad. Sci.* **1217**: 83‐95 [PMID:21091716]

Foord SM *et al*. (2005) International Union of Pharmacology. XLVI. G protein‐coupled receptor list. *Pharmacol Rev* **57**: 279‐288 [PMID:15914470]

Pantaleo N *et al*. (2010) The mammalian tachykinin ligand‐receptor system: an emerging target for central neurological disorders. *CNS Neurol Disord Drug Targets* **9**: 627‐35 [PMID:20632965]

Rance NE *et al*. (2010) Neurokinin B and the hypothalamic regulation of reproduction. *Brain Res.* **1364**: 116‐28 [PMID:20800582]

Rojas C *et al*. (2012) Pharmacological mechanisms of 5‐HT_3 and tachykinin NK_1 receptor antagonism to prevent chemotherapy‐induced nausea and vomiting. *Eur. J. Pharmacol.* **684**: 1‐7 [PMID:22425650]

Tuluc F *et al*. (2009) Neurokinin 1 receptor isoforms and the control of innate immunity. *Trends Immunol.* **30**: 271‐6 [PMID:19427266]

## Thyrotropin‐releasing hormone receptors

### Overview

Thyrotropin‐releasing hormone (TRH) receptors (**provisional nomenclature as recommended by NC‐IUPHAR[** **530** **]**) are activated by the endogenous tripeptide TRH(*TRH*, P20396) (pGlu‐His‐ProNH2). TRH(*TRH*, P20396) and TRH analogues fail to distinguish TRH_1_ and TRH_2_ receptors [1822]. [^3^H]TRH (human, mouse, rat) is able to label both TRH_1_ and TRH_2_ receptors with K_d_ values of 13 and 9 nM respectively.

**Table:** 

Nomenclature	TRH_1_ receptor	TRH_2_ receptor
HGNC, UniProt	*TRHR*, P34981	–
Antagonists	diazepam (p*K*_i_ 5.2) [444] – Rat	–
Selective antagonists	midazolam (p*K*_i_ 5.5) [444] – Rat, chlordiazepoxide (p*K*_i_ 4.8) [444] – Rat, chlordiazepoxide (p*K*_i_ 4.7) [1804] – Mouse	–
Comments	–	A class A G protein‐coupled receptor: not present in man

### Further Reading

Bílek R *et al*. (2011) TRH‐like peptides. *Physiol Res* **60**: 207‐15 [PMID:21114375]

Foord SM *et al*. (2005) International Union of Pharmacology. XLVI. G protein‐coupled receptor list. *Pharmacol Rev* **57**: 279‐288 [PMID:15914470]

Nillni EA. (2010) Regulation of the hypothalamic thyrotropin releasing hormone (TRH) neuron by neuronal and peripheral inputs. *Front Neuroendocrinol* **31**: 134‐56 [PMID:20074584]

## Trace amine receptor

### Overview

Trace amine‐associated receptors were initially discovered as a result of a search for novel 5‐HT receptors [185], where 15 mammalian orthologues were identified and divided into two families. The TA_1_ receptor (**nomenclature as agreed by the NC‐IUPHAR Subcommittee for the Trace amine receptor [** **1181** **]**) has been shown to have affinity for the endogenous trace amines tyramine, *β*‐phenylethylamine and octopamine in addition to the classical amine dopamine[185]. Emerging evidence suggests that TA_1_ is a modulator of monoaminergic activity in the brain [2062] with TA_1_ and dopamine D_2_ receptors shown to form constitutive heterodimers when co‐expressed [492]. In addition to trace amines, receptors can be activated by amphetamine‐like psychostimulants, and endogenous thyronamines such as thyronamine and 3‐iodothyronamine.

**Table:** 

Nomenclature	TA_1_ receptor
HGNC, UniProt	*TAAR1*, Q96RJ0
Rank order of potency	tyramine>*β*‐phenylethylamine>octopamine = dopamine [185]
Agonists	RO5166017 (pEC_50_ 7.3) [1574]
Antagonists	EPPTB (Inverse agonist) (pIC_50_ 5.1) [199]
Labelled ligands	[^3^H]tyramine (Agonist) (p*K*_d_ 7.7) [185]

### Comments

In addition to TA_1_, analysis has shown that in man there are up to 5 functional TAAR genes (TAAR2,5,6,8,9). See [185] for detailed discussion. The product of the gene TAAR2 (also known as GPR58) appears to respond to *β*‐phenylethylamine>tyramine and to couple through G_s_[185].

*TAAR3*, in some individuals, and *TAAR4* are pseudogenes in man, although functional in rodents. The signalling characteristics and pharmacology of TAA_5_(PNR, Putative Neurotransmitter Receptor: *TAAR5*, O14804), TAA_6_(Trace amine receptor 4, TaR‐4: *TAAR6*, 96RI8), TAA_8_(Trace amine receptor 5, GPR102: *TAAR8*, Q969N4) and TAA_9_(trace amine associated receptor 9: TAAR9, 96RI9) are lacking. The thyronamines, endogenous derivatives of thyroid hormone, have been shown to have affinity for rodent cloned trace amine receptors, including TA_1_[1657]. An antagonist EPPTB has recently been described that has a *p*K_i_ of 9.1 at the mouse TA_1_ but less than 5.3 for human TA_1_[1792].

### Further Reading

Jing L *et al*. (2015) Trace amine‐associated receptor 1: A promising target for the treatment of psychostimulant addiction. *Eur. J. Pharmacol.* [PMID:26092759]

Liberles SD. (2015) Trace amine‐associated receptors: ligands, neural circuits, and behaviors. *Curr. Opin. Neurobiol.* **34C**: 1‐7 [PMID:25616211]

Maguire JJ *et al*. (2009) International Union of Pharmacology. LXXII. Recommendations for trace amine receptor nomenclature. *Pharmacol. Rev.* **61**: 1‐8 [PMID:19325074]

Miller GM. (2011) The emerging role of trace amine‐associated receptor 1 in the functional regulation of monoamine transporters and dopaminergic activity. *J. Neurochem.* **116**: 164‐76 [PMID:21073468]

Sotnikova TD *et al*. (2009) Trace amine‐associated receptors as emerging therapeutic targets. *Mol. Pharmacol.* **76**: 229‐35 [PMID:19389919]

Zucchi R *et al*. (2006) Trace amine‐associated receptors and their ligands. *Br J Pharmacol* **149**: 967‐978 [PMID:17088868]

## Urotensin receptor

### Overview

The urotensin‐II (U‐II) receptor (UT, **nomenclature as agreed by the NC‐IUPHAR Subcommittee on the Urotensin receptor [439,** **530** **,** **1952** **]**) is activated by the endogenous dodecapeptide urotensin‐II(*UTS2*, O95399), originally isolated from the urophysis, the endocrine organ of the caudal neurosecretory system of teleost fish [134]. Several structural forms of U‐II exist in fish and amphibians. The Goby orthologue was used to identify U‐II as the cognate ligand for the predicted receptor encoded by the rat gene *gpr14* [375, 1130, 1327, 1410]. Human urotensin‐II(*UTS2*, O95399), an 11‐amino‐acid peptide [375], retains the cyclohexapeptide sequence of goby U‐II that is thought to be important in ligand binding [219, 957]. This sequence is also conserved in the deduced amino‐acid sequence of rat urotensin‐II {Rat} (14 amino‐acids) and mouse urotensin‐II{Mouse} (14 amino‐acids), although the N‐terminal is more divergent from the human sequence [374]. A second endogenous ligand for UT has been discovered in rat [1816]. This is the urotensin II‐related peptide (*UTS2B*, Q765I0), an octapeptide that is derived from a different gene, but shares the C‐terminal sequence (CFWKYCV) common to U‐II from other species. Identical sequences to rat urotensin II‐related peptide (*UTS2B*, Q765I0) are predicted for the mature mouse and human peptides.

**Table:** 

Nomenclature	UT receptor
HGNC, UniProt	*UTS2R*, Q9UKP6
Endogenous agonists	urotensin II‐related peptide (*UTS2B*, Q765I0) (p*K*_d_ 9.6) [1179], urotensin‐II (*UTS2*, O95399) (p*K*_i_ 8.6) [440, 475, 647]
Selective agonists	[Pen5]‐U (4‐11) (human) (p*K*_i_ 9.7) [647], U‐II‐(4‐11) (human) (p*K*_i_ 9.6) [647], FL104 (pEC_50_ 5.8–7.5) [1075, 1077], AC‐7954 (p*K*_i_ 6.6) [382, 1076]
Selective antagonists	urantide (p*K*_i_ 8.3) [1469], SB‐706375 (p*K*_i_ 8) [440], palosuran (pIC_50_ 7.1) [353], SB‐611812 (p*K*_i_ 6.6) [1550]
Labelled ligands	[^125^I]U‐II (human) (Agonist) (p*K*_d_ 9.4–9.6) [42, 1179]

### Comments

In human vasculature, human urotensin‐II(*UTS2*, O95399) elicits both vasoconstrictor (p*D*_2_ 9.3‐10.1, [1179]) and vasodilator (pIC_50_ 10.3‐10.4, [1800]) responses.

### Further Reading

Douglas SA Ohlstein EH. (2000) Urotensin receptors. *In The IUPHAR Receptor Compendium of Receptor Characterization and Classification.* Edited by Girdlestone D: IUPHAR Media Ltd: 365‐372

Foord SM *et al*. (2005) International Union of Pharmacology. XLVI. G protein‐coupled receptor list. *Pharmacol Rev* **57**: 279‐288 [PMID:15914470]

Guidolin D *et al*. (2010) Urotensin‐II as an angiogenic factor. *Peptides* **31**: 1219‐24 [PMID:20346384]

Hunt BD *et al*. (2010) A rat brain atlas of urotensin‐II receptor expression and a review of central urotensin‐II effects. *Naunyn Schmiedebergs Arch. Pharmacol.* **382**: 1‐31 [PMID:20422157]

Maryanoff BE *et al*. (2010) Urotensin‐II receptor modulators as potential drugs. *J. Med. Chem.* **53**: 2695‐708 [PMID:20043680]

Ross B *et al*. (2010) Role of urotensin II in health and disease. *Am. J. Physiol. Regul. Integr. Comp. Physiol.* **298**: R1156‐72 [PMID:20421634]

## Vasopressin and oxytocin receptors

### Overview

Vasopressin (AVP) and oxytocin (OT) receptors (**nomenclature as recommended by NC‐IUPHAR[** **530** **]**) are activated by the endogenous cyclic nonapeptides vasopressin(*AVP*, P01185) and oxytocin(*OXT*, P01178). These peptides are derived from precursors which also produce neurophysins (neurophysin I for oxytocin; neurophysin II for vasopressin).

**Table:** 

Nomenclature	V_1A_ receptor	V_1B_ receptor	V_2_ receptor	OT receptor
HGNC, UniProt	*AVPR1A*, P37288	*AVPR1B*, P47901	*AVPR2*, P30518	*OXTR*, P30559
Rank order of potency	vasopressin (*AVP*, P01185) >oxytocin (*OXT*, P01178)	vasopressin (*AVP*, P01185) >oxytocin (*OXT*, P01178)	vasopressin (*AVP*, P01185) >oxytocin (*OXT*, P01178)	oxytocin (*OXT*, P01178) >vasopressin (*AVP*, P01185)
Endogenous agonists	vasopressin (*AVP*, P01185) (p*K*_i_ 8.5–9.3) [24, 311, 369, 415, 1359, 1501, 1627, 1839, 1840, 1870, 1871, 2073]	vasopressin (*AVP*, P01185) (p*K*_i_ 9–9.5) [24, 311, 415, 648, 1359, 1627, 1839, 1840, 1871, 2073]	vasopressin (*AVP*, P01185) (p*K*_i_ 7.9–9.1) [24, 311, 319, 415, 1359, 1627, 1700, 1839, 1840, 1871, 2073]	oxytocin (*OXT*, P01178) (p*K*_i_ 8.2–9.6) [24, 319, 320, 345, 648, 853]
Selective agonists	F180 (p*K*_d_ 7.9–8.3) [49, 369]	d[Leu^4^]LVP (p*K*_i_ 9.8) [1485], d[Cha^4^]AVP (p*K*_i_ 9–9.7) [415, 648]	VNA932 (pIC_50_ 7.1) [501], OPC‐51803 (p*K*_i_ 7) [1359], d[Val^4^,DArg^8^]VP	[Thr^4^,Gly^7^]OT (p*K*_i_ 8.2–8.4) [320, 472, 853]
Antagonists	conivaptan (p*K*_i_ 8.2–8.4) [1839, 1840]	nelivaptan (p*K*_i_ 8.4–9.3) [644, 648, 1702]	–	L‐371,257 (p*K*_i_ 8.8) [648]
Selective antagonists	relcovaptan (p*K*_i_ 8.1–9.3) [24, 369, 648, 1501, 1700, 1839, 1870, 1871, 1910], d(CH_2_)_5_[Tyr(Me)^2^,Arg^8^]VP (p*K*_i_ 9)	–	conivaptan (p*K*_i_ 9.4) [381], tolvaptan (p*K*_i_ 9.4) [2073], satavaptan (p*K*_i_ 8.4–9.3) [24, 369, 370, 1699, 1700, 1839, 1910], lixivaptan (Inverse agonist) (p*K*_i_ 8.9–9.2) [33, 1700], d(CH_2_)_5_[D‐Ile^2^,Ile^4^]AVP (p*K*_i_ 6.9–8.4) [1700], mozavaptan (Inverse agonist) (p*K*_i_ 7.4–8.1) [370, 1700, 1839, 1871, 2073, 2074]	SSR126768A (p*K*_i_ 8.8–9.1) [1701], desGlyNH_2_‐d(CH_2_)_5_[Tyr(Me)^2^,Thr^4^,Orn^8^]OT (p*K*_i_ 8.5), L‐372662 (p*K*_i_ 8.4) [121]
Labelled ligands	[^125^I]OH‐LVA (Antagonist) (p*K*_d_ 10.3–10.4) [319, 369, 1501], [^3^H]AVP (human, mouse, rat) (Agonist) (p*K*_d_ 8.6–10.2) [208, 319, 369, 370, 1359, 1501, 1627, 1839, 1840, 1870, 1871, 1910, 2073], [^3^H]d(CH_2_)_5_[Tyr(Me)^2^]AVP (Antagonist) (p*K*_d_ 9)	[^3^H]AVP (human, mouse, rat) (Agonist) (p*K*_d_ 8.6–9.6) [208, 319, 369, 370, 1359, 1501, 1627, 1839, 1840, 1870, 1871, 1910, 2073]	[^3^H]AVP (human, mouse, rat) (Agonist) (p*K*_d_ 8.4–9.4) [319, 369, 370, 1359, 1627, 1839, 1840, 1871, 1910, 2073], [^3^H]dDAVP (Agonist) (p*K*_d_ 7.2–9.1) [319, 370, 1871], [^3^H]desGly‐NH_2_[D‐Ile^2^,Ile^4^]VP (p*K*_d_ 8.6)	[^125^I]d(CH_2_)_5_[Tyr(Me)^2^,Thr^4^,Orn^8^,Tyr‐NH_2_^9^]OVT (Antagonist) (p*K*_d_ 10), [^3^H]OT (human, mouse, rat) (Agonist) (p*K*_d_ 8.2–9.5) [319, 553, 853, 952], [^111^In]DOTA‐dLVT (p*K*_d_ 8.3) [318]

### Comments

The V_2_ receptor exhibits marked species differences, such that many ligands (d(CH_2_)_5_[D‐Ile^2^,Ile^4^]AVP and [^3^H]desGly‐NH_2_[D‐Ile^2^,Ile^4^]VP) exhibit low affinity at human V_2_ receptors [29]. Similarly, [^3^H]d[D‐Arg^8^]VP is V_2_ selective in the rat, not in the human [1627]. The gene encoding the V_2_ receptor is polymorphic in man, underlying nephrogenic diabetes insipidus [148]. D[Cha^4^]AVP is selective only for the human and bovine V_1b_ receptors [415], while d[Leu^4^]LVP has high affinity for the rat V_1b_ receptor [1485].

### Further Reading

Bartz JA *et al*. (2011) Social effects of oxytocin in humans: context and person matter. *Trends Cogn. Sci. (Regul. Ed.)* **15**: 301‐9 [PMID:21696997]

Knepper MA. (2012) Systems biology in physiology: the vasopressin signaling network in kidney. *Am. J. Physiol., Cell Physiol.* **303**: C1115‐24 [PMID:22932685]

Koshimizu TA *et al*. (2012) Vasopressin V1a and V1b receptors: from molecules to physiological systems. *Physiol. Rev.* **92**: 1813‐64 [PMID:23073632]

Manning M *et al*. (2012) Oxytocin and vasopressin agonists and antagonists as research tools and potential therapeutics. *J. Neuroendocrinol.* **24**: 609‐28 [PMID:22375852]

Meyer‐Lindenberg A *et al*. (2011) Oxytocin and vasopressin in the human brain: social neuropeptides for translational medicine. *Nat. Rev. Neurosci.* **12**: 524‐38 [PMID:21852800]

Neumann ID *et al*. (2012) Balance of brain oxytocin and vasopressin: implications for anxiety, depression, and social behaviors. *Trends Neurosci.* **35**: 649‐59 [PMID:22974560]

## VIP and PACAP receptors

### Overview

Vasoactive intestinal peptide (VIP) and pituitary adenylate cyclase‐activating peptide (PACAP) receptors (**nomenclature as agreed by the NC‐IUPHAR Subcommittee on Vasoactive Intestinal Peptide Receptors [** **704** **,** **705** **]**) are activated by the endogenous peptides VIP(*VIP*, P01282), PACAP‐38(*ADCYAP1*, P18509), PACAP‐27(*ADCYAP1*, P18509), peptide histidine isoleucineamide (PHI {Mouse, Rat}), peptide histidine methionineamide (PHM(*VIP*, P01282)) and peptide histidine valine (PHV(*VIP*, P01282)). VPAC_1_ and VPAC_2_ receptors display comparable affinity for the PACAP peptides, PACAP‐27(*ADCYAP1*, P18509) and PACAP‐38(*ADCYAP1*, P18509), and VIP(*VIP*, P01282), whereas PACAP‐27(*ADCYAP1*, P18509) and PACAP‐38(*ADCYAP1*, P18509) are >100 fold more potent than VIP(*VIP*, P01282) as agonists of most isoforms of the PAC_1_ receptor. However, one splice variant of the human PAC_1_ receptor has been reported to respond to PACAP‐38(*ADCYAP1*, P18509), PACAP‐27(*ADCYAP1*, P18509) and VIP(*VIP*, P01282) with comparable affinity [393]. PG 99‐465[1320] has been used as a selective VPAC_2_receptor antagonist in a number of physiological studies, but has been reported to have significant activity at VPAC_1_ and PAC_1_ receptors [422]. The selective PAC_1_ receptor agonist maxadilan, was extracted from the salivary glands of sand flies (*Lutzomyia longipalpis*) and has no sequence homology to VIP(*VIP*, P01282) or the PACAP peptides [1330]. Two deletion variants of maxadilan, M65[1918] and Max.d.4[1331] have been reported to be PAC_1_ receptor antagonists, but these peptides have not been extensively characterised.

**Table:** 

Nomenclature	PAC_1_ receptor	VPAC_1_ receptor	VPAC_2_ receptor
HGNC, UniProt	*ADCYAP1R1*, P41586	*VIPR1*, P32241	*VIPR2*, P41587
Rank order of potency	PACAP‐27 (*ADCYAP1*, P18509), PACAP‐38 (*ADCYAP1*, P18509) ≫VIP (*VIP*, P01282)	VIP (*VIP*, P01282), PACAP‐27 (*ADCYAP1*, P18509), PACAP‐38 (*ADCYAP1*, P18509) ≫GHRH (*GHRH*, P01286), PHI {Pig}, secretin (*SCT*, P09683)	VIP (*VIP*, P01282), PACAP‐38 (*ADCYAP1*, P18509), PACAP‐27 (*ADCYAP1*, P18509) >PHI {Pig} ≫GHRH (*GHRH*, P01286), secretin (*SCT*, P09683)
Selective agonists	maxadilan (pEC_50_ 10.3) [422], maxadilan (pEC_50_ 6.2) [422]	[Lys^15^,Arg^16^,Leu^27^]VIP‐(1‐7)/GRF‐(8‐27)‐NH_2_ (pEC_50_ 8.3) [1315], [Ala^11,22,28^]VIP (p*K*_i_ 8.1) [1393]	Ro 25‐1553 (pIC_50_ 7.8–9.5) [634, 887, 1315], Ro 25‐1392 (p*K*_i_ 8) [2056]
Selective antagonists	–	PG 97‐269 (pIC_50_ 8.7) [633, 887]	–
Labelled ligands	[^125^I]PACAP‐27 (Agonist) (p*K*_d_ 9.1) [1509]	[^125^I]VIP (human, mouse, rat) (Agonist) (p*K*_d_ 9.4) [1393], [^125^I]PACAP‐27 (Agonist)	[^125^I]VIP (human, mouse, rat) (Agonist) (p*K*_d_ 9.2) [1393], [^125^I]PACAP‐27 (Agonist)

### Comments

Subtypes of PAC_1_ receptors have been proposed based on tissue differences in the potencies of PACAP‐27(*ADCYAP1*, P18509) and PACAP‐38(*ADCYAP1*, P18509); these might result from differences in G protein coupling and second messenger mechanisms [1939], or from alternative splicing of PAC_1_ receptor mRNA [1788].

### Further Reading

Harmar AJ *et al*. (1998) International Union of Pharmacology. XVIII. Nomenclature of receptors for vasoactive intestinal peptide and pituitary adenylate cyclase‐activating polypeptide. *Pharmacol Rev* **50**: 265‐270 [PMID:9647867]

Harmar AJ *et al*. (2012) Pharmacology and functions of receptors for vasoactive intestinal peptide and pituitary adenylate cyclase‐activating polypeptide: IUPHAR review 1. *Br. J. Pharmacol.* **166**: 4‐17 [PMID:22289055]

Reglodi D *et al*. (2012) Effects of pituitary adenylate cyclase activating polypeptide in the urinary system, with special emphasis on its protective effects in the kidney. *Neuropeptides* **46**: 61‐70 [PMID:21621841]

Smith CB *et al*. (2012) Is PACAP the major neurotransmitter for stress transduction at the adrenomedullary synapse? *J. Mol. Neurosci.* **48**: 403‐12 [PMID:22610912]
